# Supplementary figures and images for: miR-381-3p contribution in mouse spontaneous abortion via targeting VEGFA (part 1 of 2)
Source: PeerJ. 2025 Jun 24;13:e19568. doi: 10.7717/peerj.19568 (PMC12204090; doi:10.7717/peerj.19568)

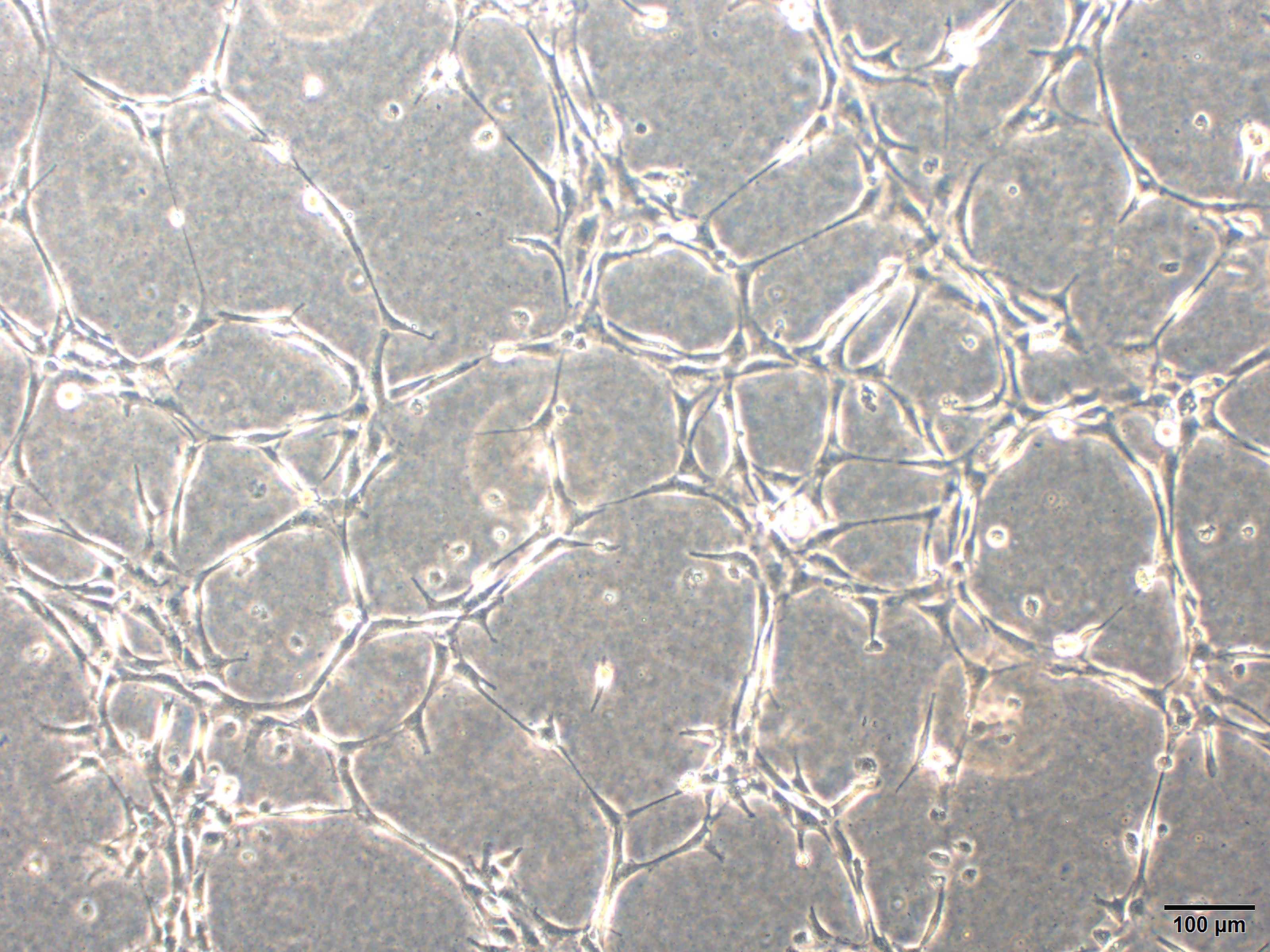

Supplement: Supplemental Information 1 [file peerj-13-19568-s001.zip › Figure 2A and 4C (angiogenesis)/MiRNA inhibitor (1).jpg]

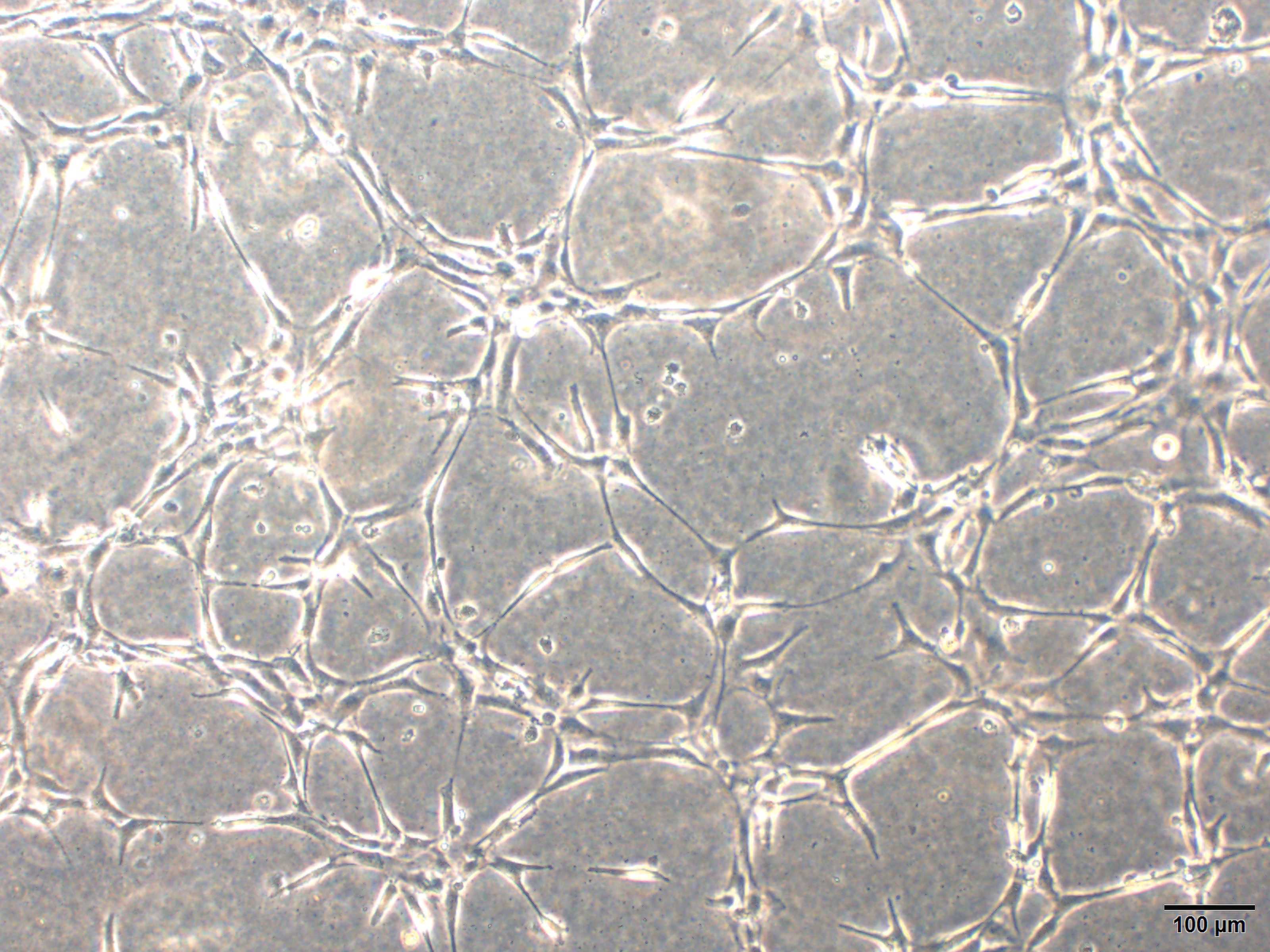

Supplement: Supplemental Information 1 [file peerj-13-19568-s001.zip › Figure 2A and 4C (angiogenesis)/MiRNA inhibitor (2).jpg]

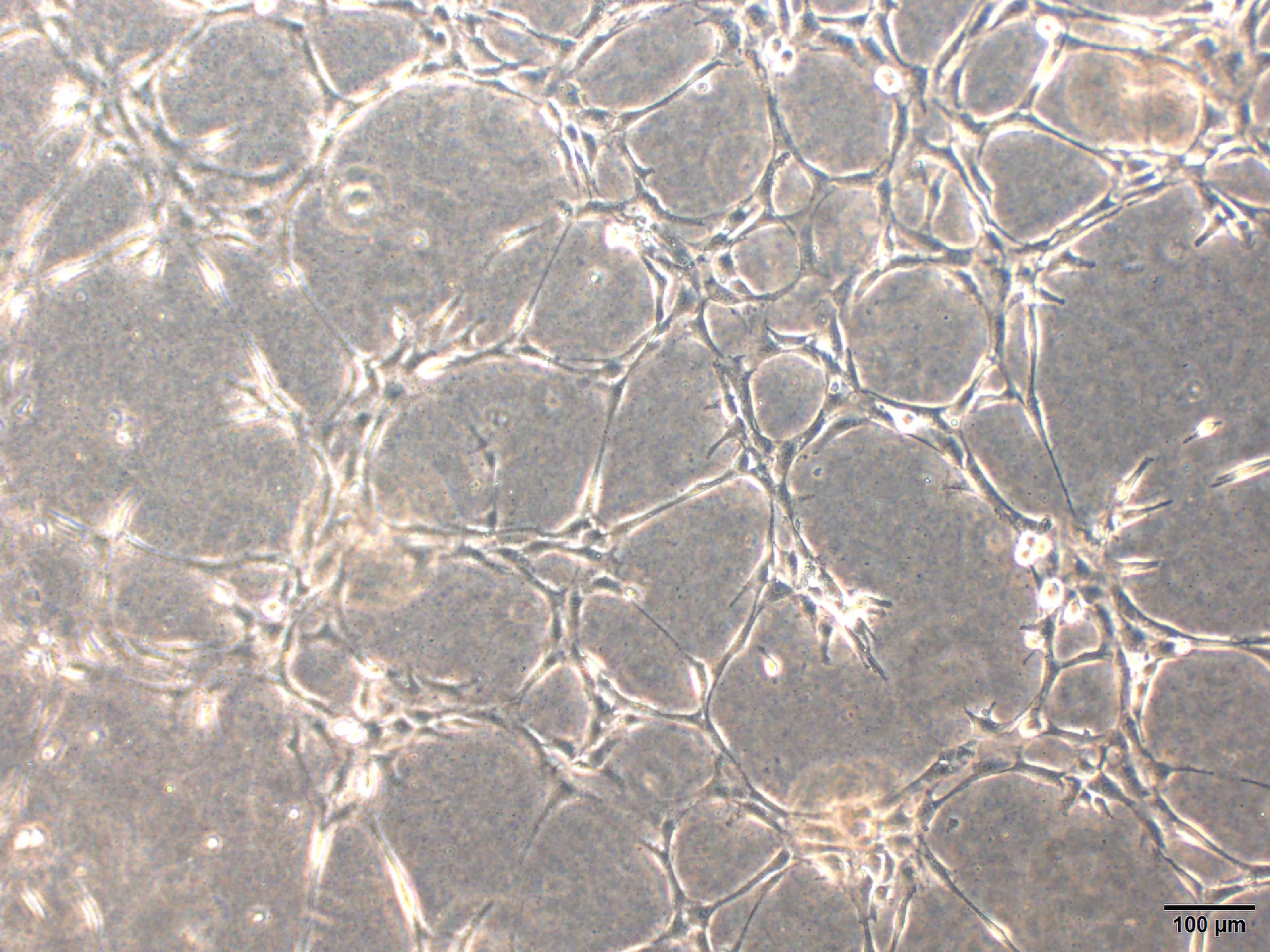

Supplement: Supplemental Information 1 [file peerj-13-19568-s001.zip › Figure 2A and 4C (angiogenesis)/MiRNA inhibitor (3).jpg]

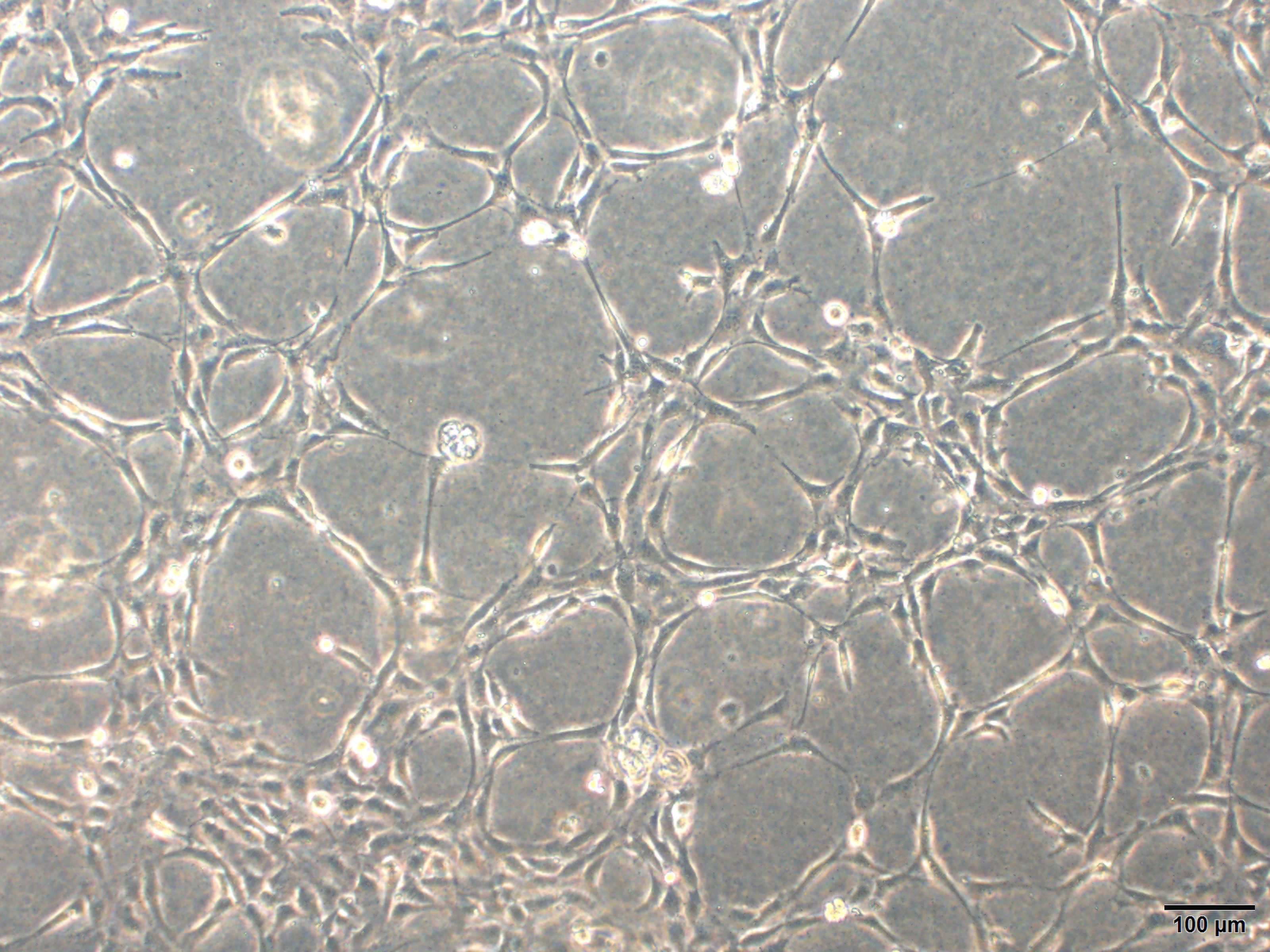

Supplement: Supplemental Information 1 [file peerj-13-19568-s001.zip › Figure 2A and 4C (angiogenesis)/MiRNA inhibitor +si-NC (1).jpg]

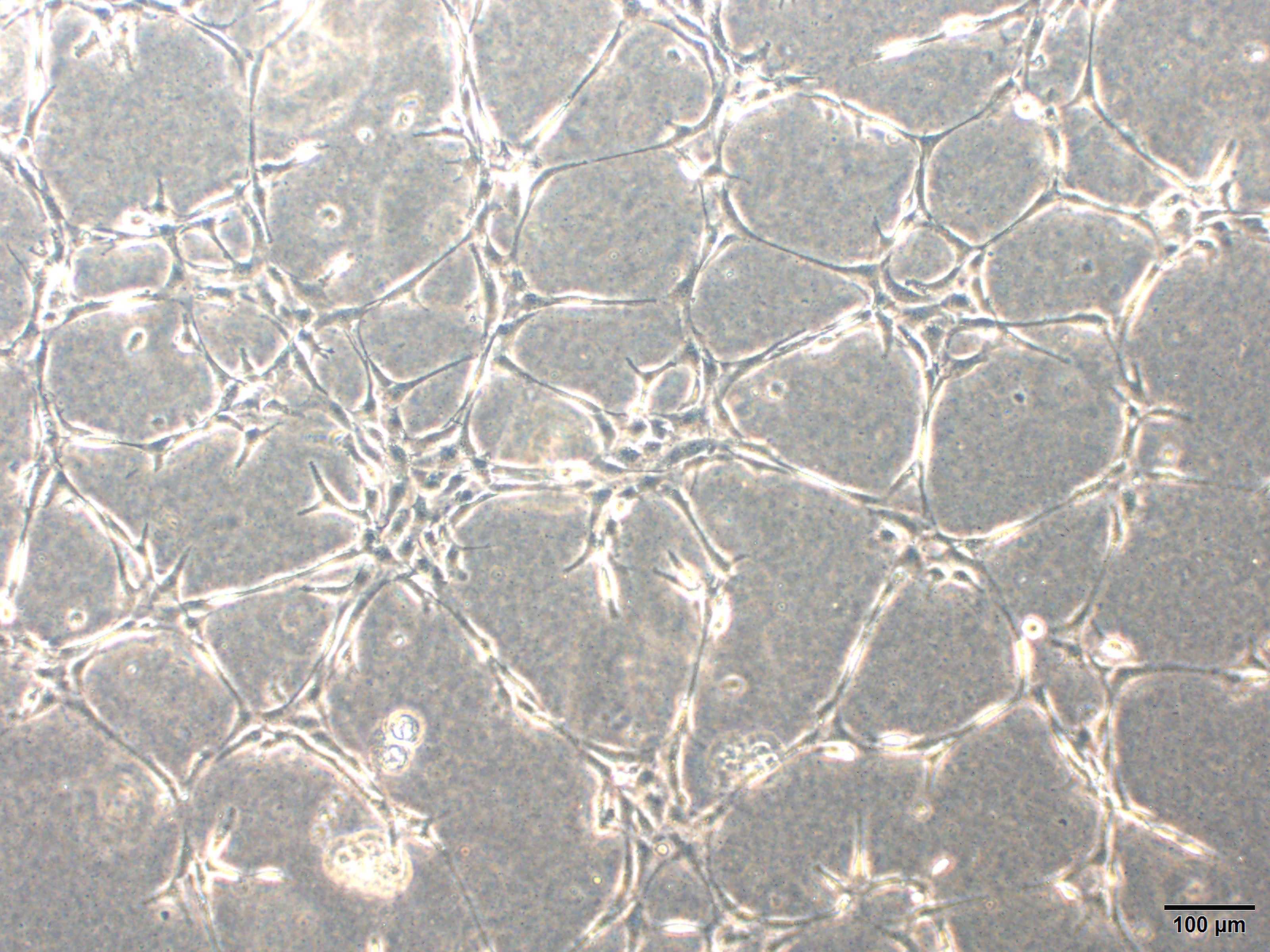

Supplement: Supplemental Information 1 [file peerj-13-19568-s001.zip › Figure 2A and 4C (angiogenesis)/MiRNA inhibitor +si-NC (2).jpg]

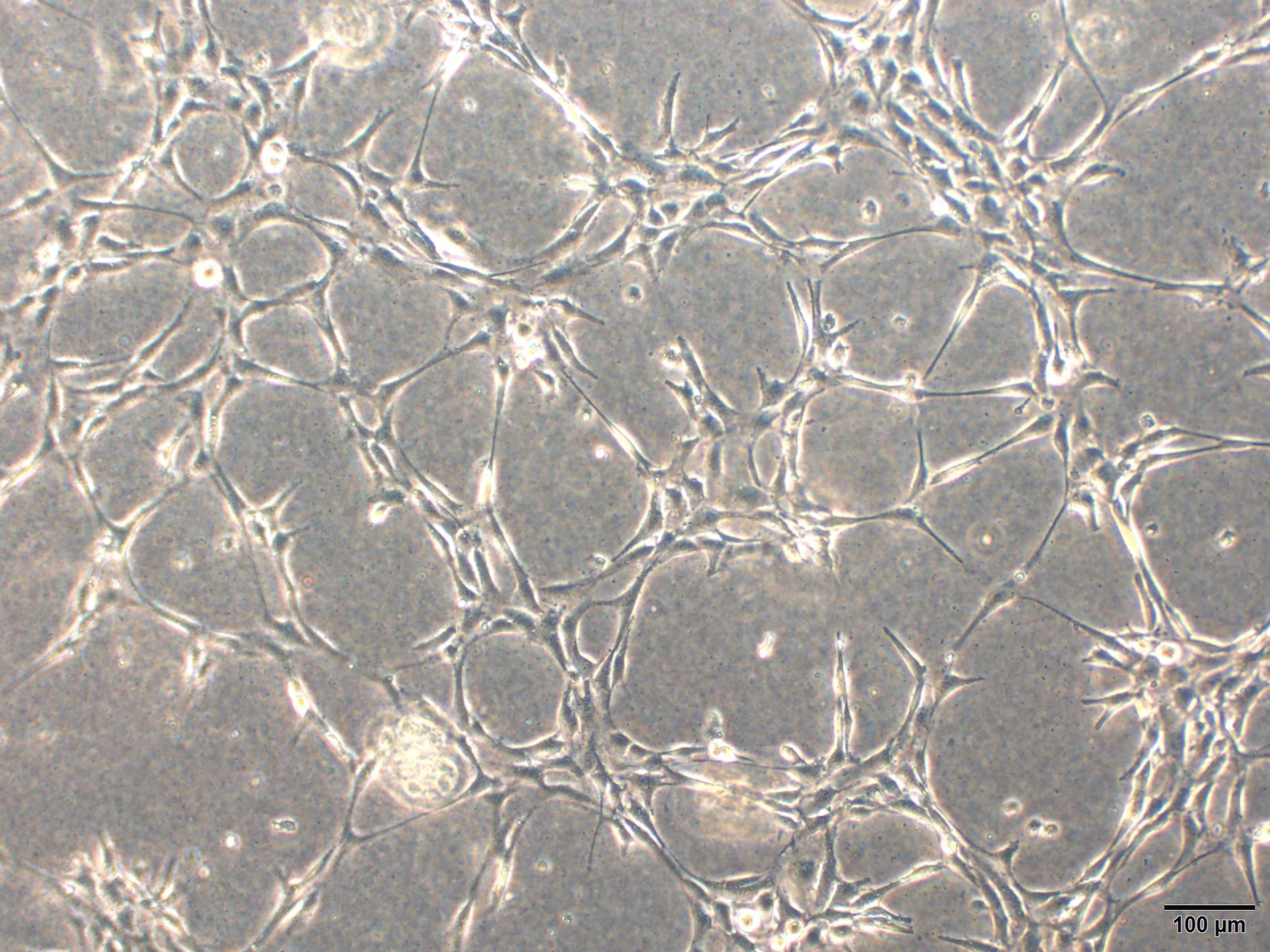

Supplement: Supplemental Information 1 [file peerj-13-19568-s001.zip › Figure 2A and 4C (angiogenesis)/MiRNA inhibitor +si-NC (3).jpg]

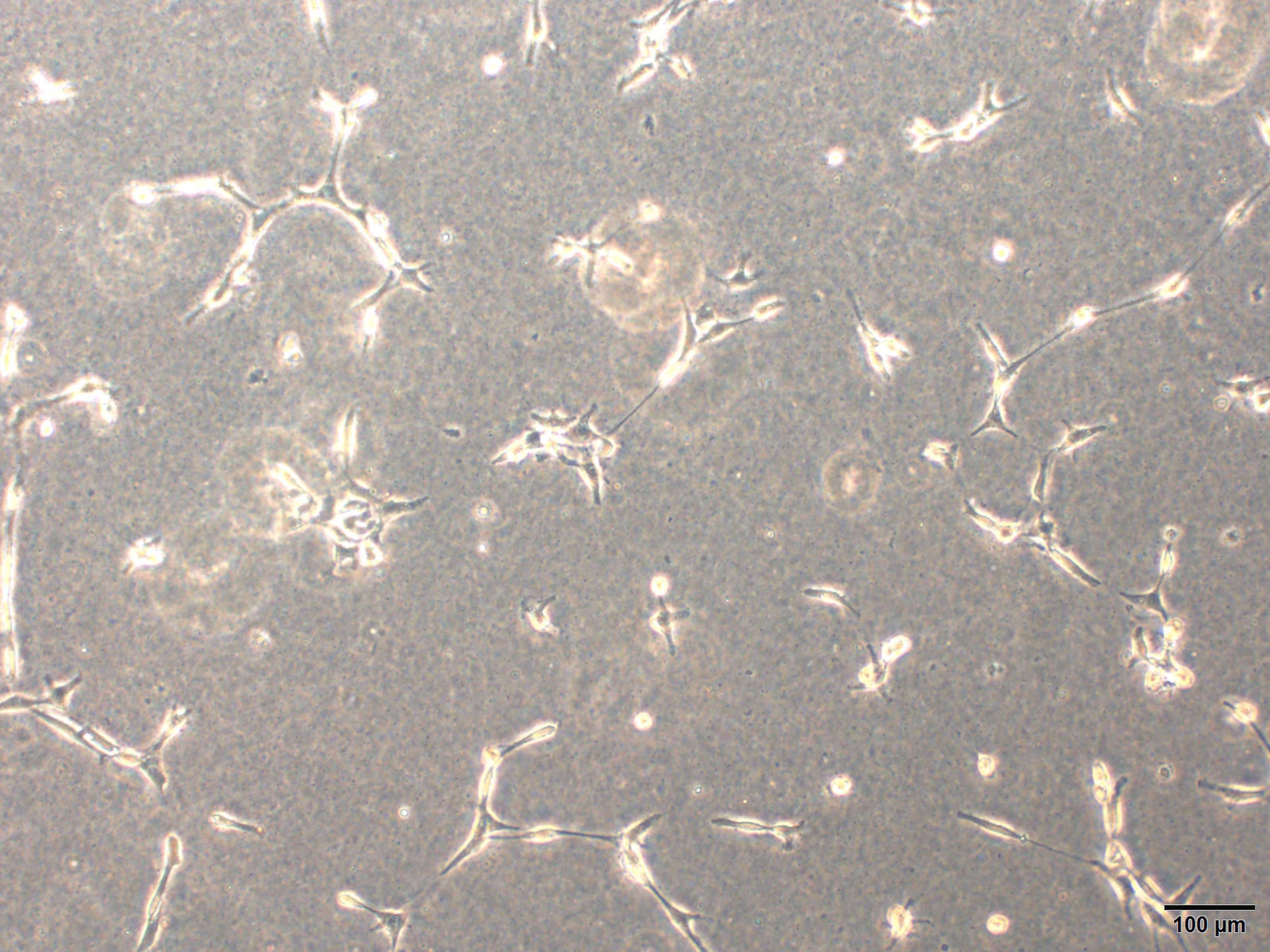

Supplement: Supplemental Information 1 [file peerj-13-19568-s001.zip › Figure 2A and 4C (angiogenesis)/MiRNA inhibitors+si-VEGF (1).jpg]

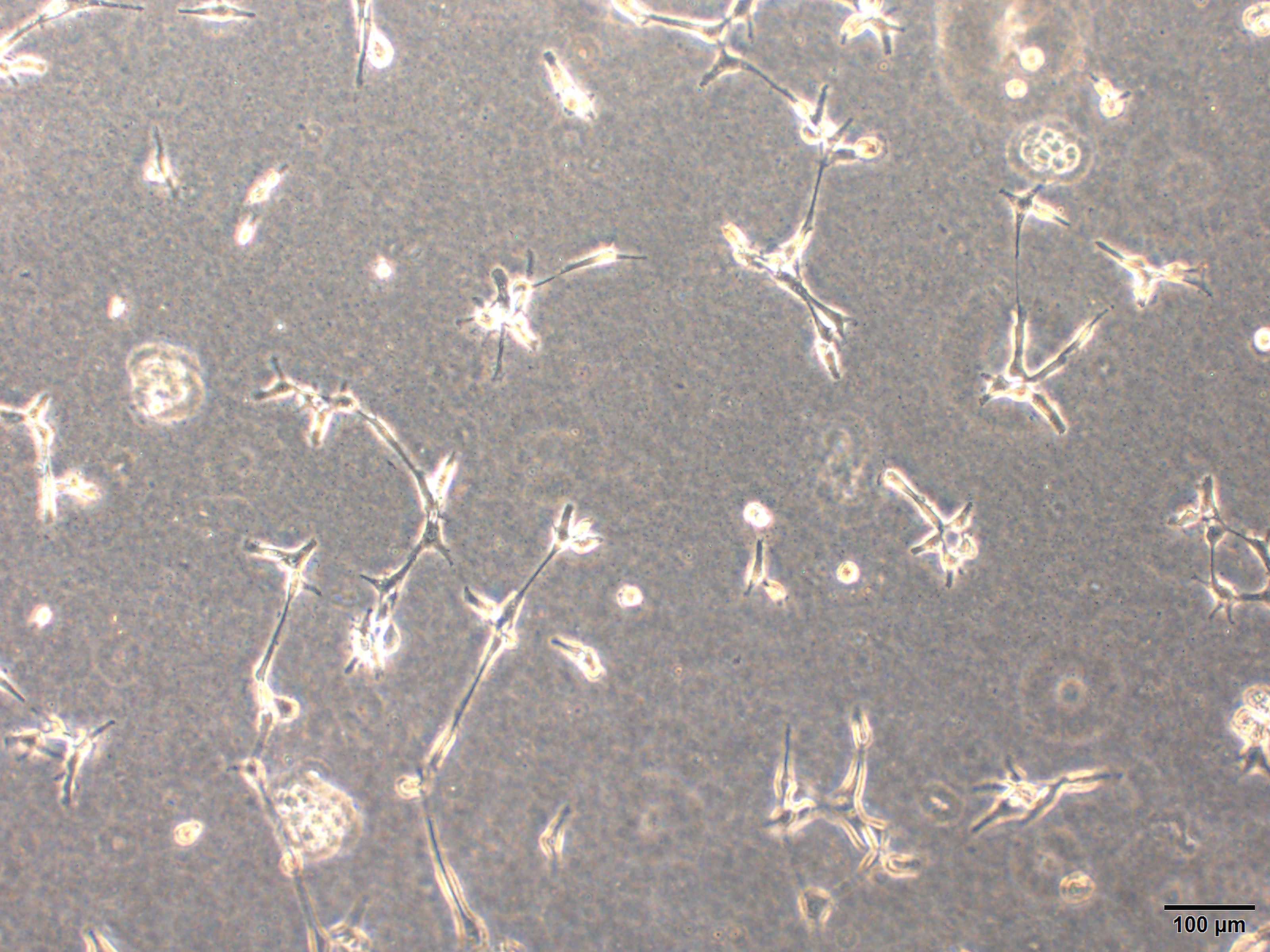

Supplement: Supplemental Information 1 [file peerj-13-19568-s001.zip › Figure 2A and 4C (angiogenesis)/MiRNA inhibitors+si-VEGF (2).jpg]

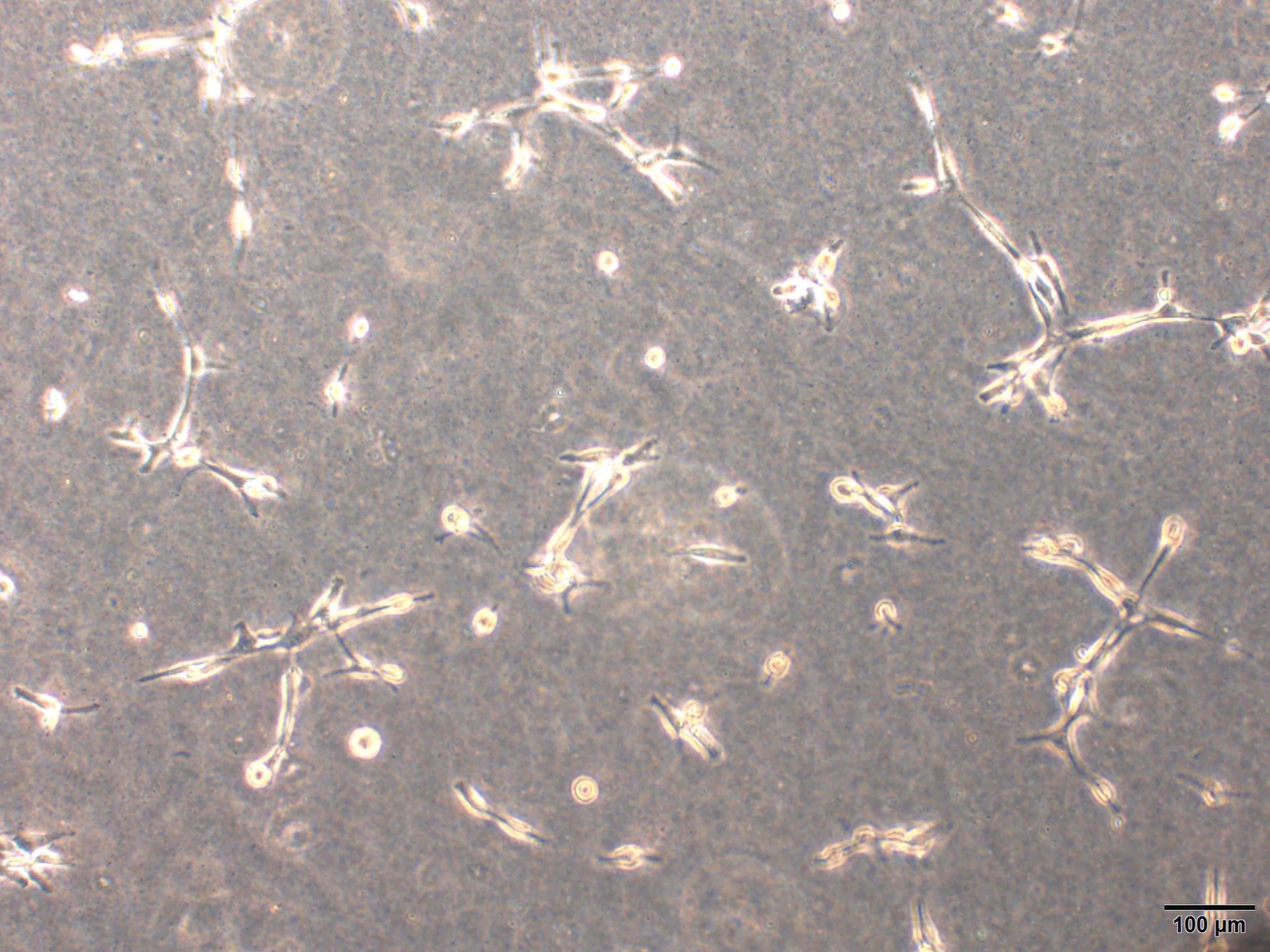

Supplement: Supplemental Information 1 [file peerj-13-19568-s001.zip › Figure 2A and 4C (angiogenesis)/MiRNA inhibitors+si-VEGF (3).jpg]

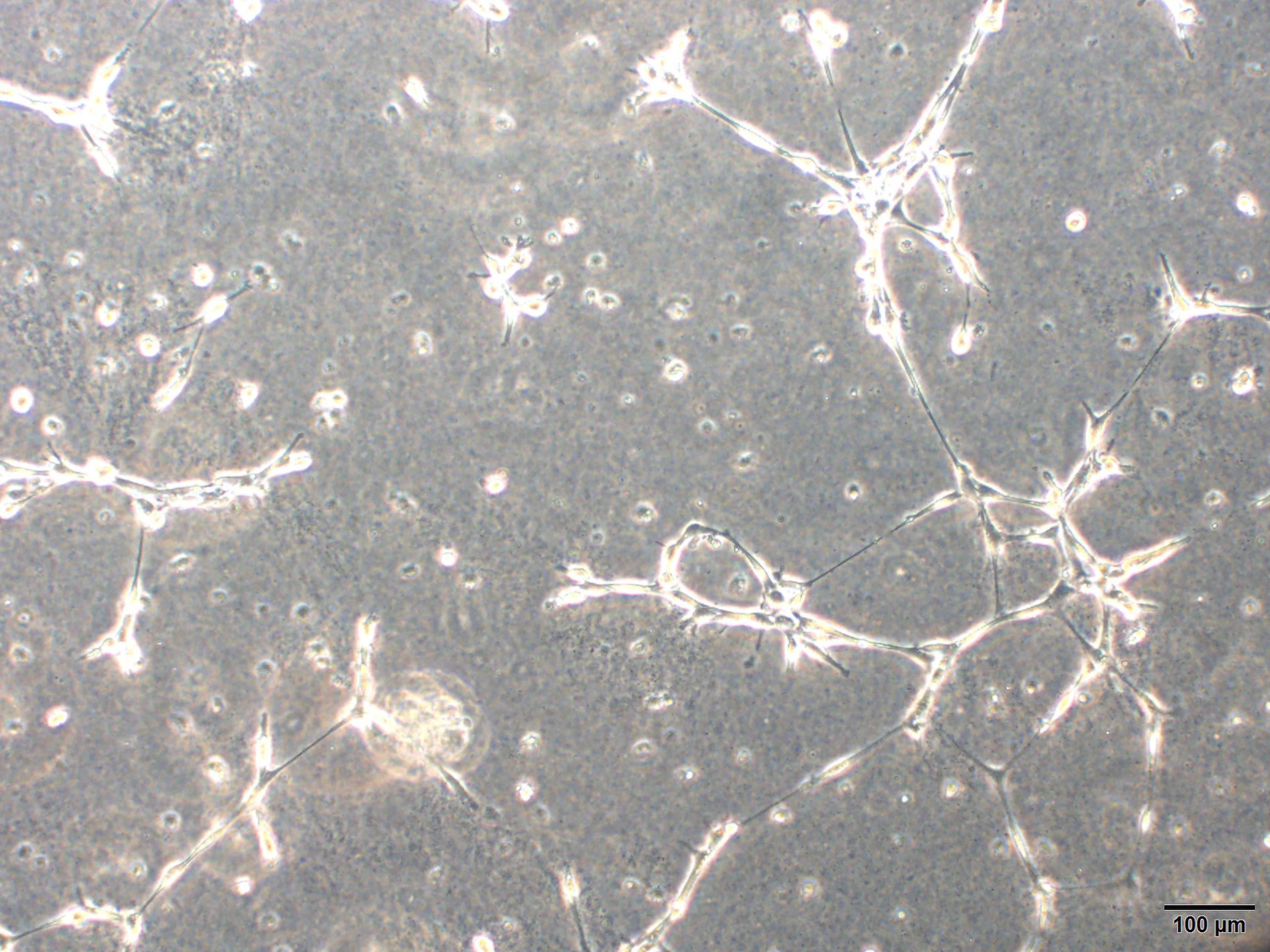

Supplement: Supplemental Information 1 [file peerj-13-19568-s001.zip › Figure 2A and 4C (angiogenesis)/MiRNA mimics (1).jpg]

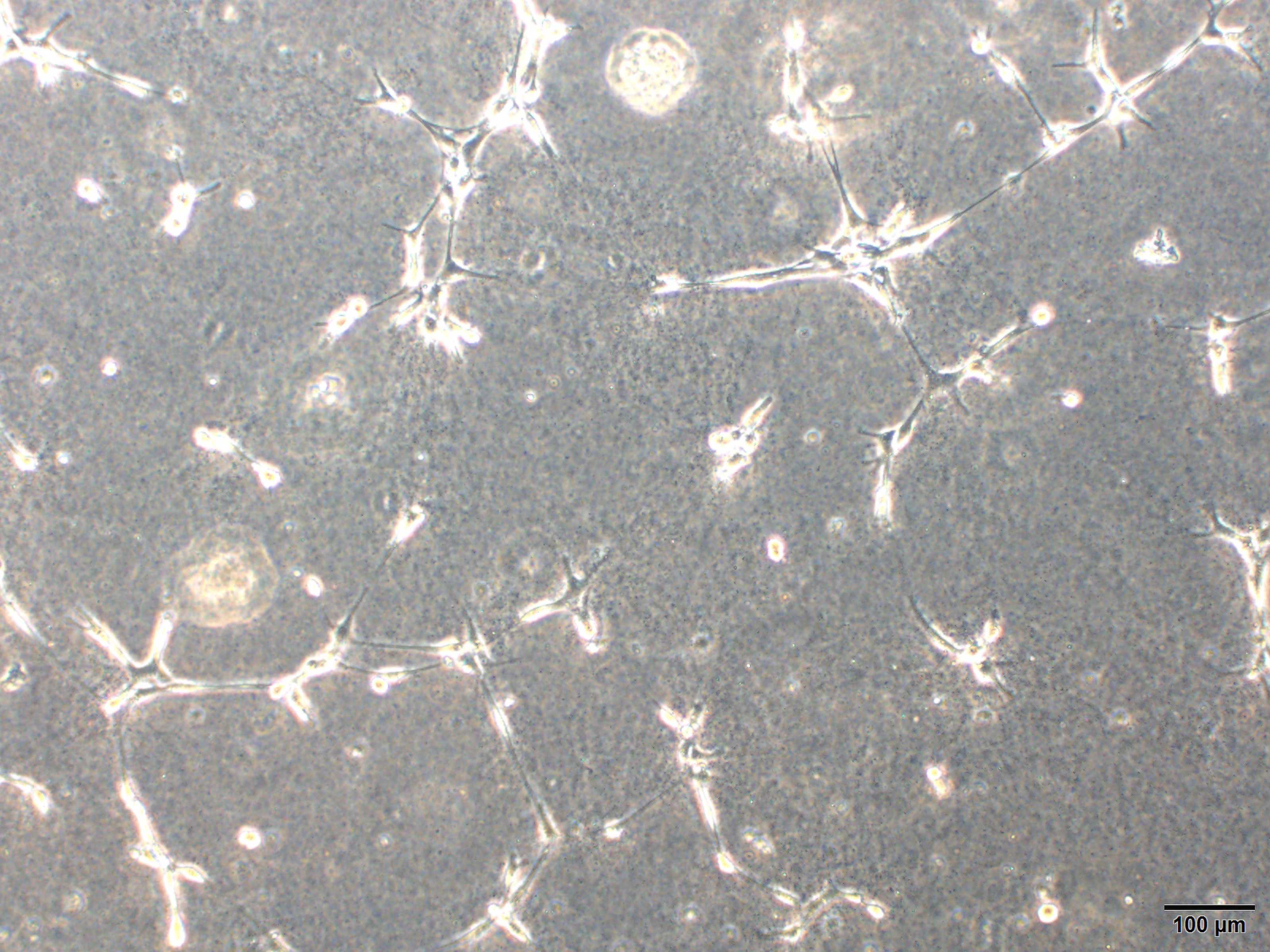

Supplement: Supplemental Information 1 [file peerj-13-19568-s001.zip › Figure 2A and 4C (angiogenesis)/MiRNA mimics (2).jpg]

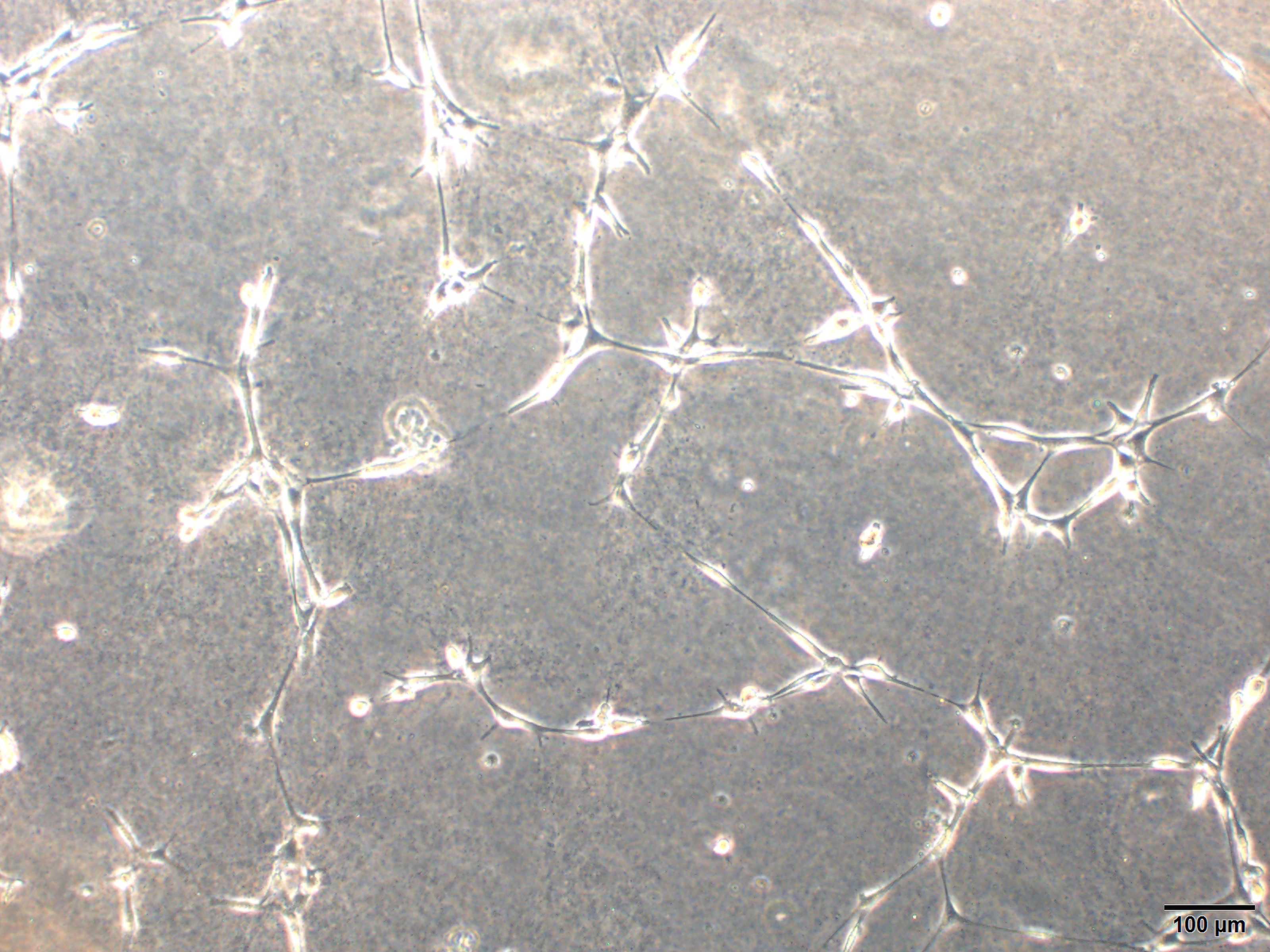

Supplement: Supplemental Information 1 [file peerj-13-19568-s001.zip › Figure 2A and 4C (angiogenesis)/MiRNA mimics (3).jpg]

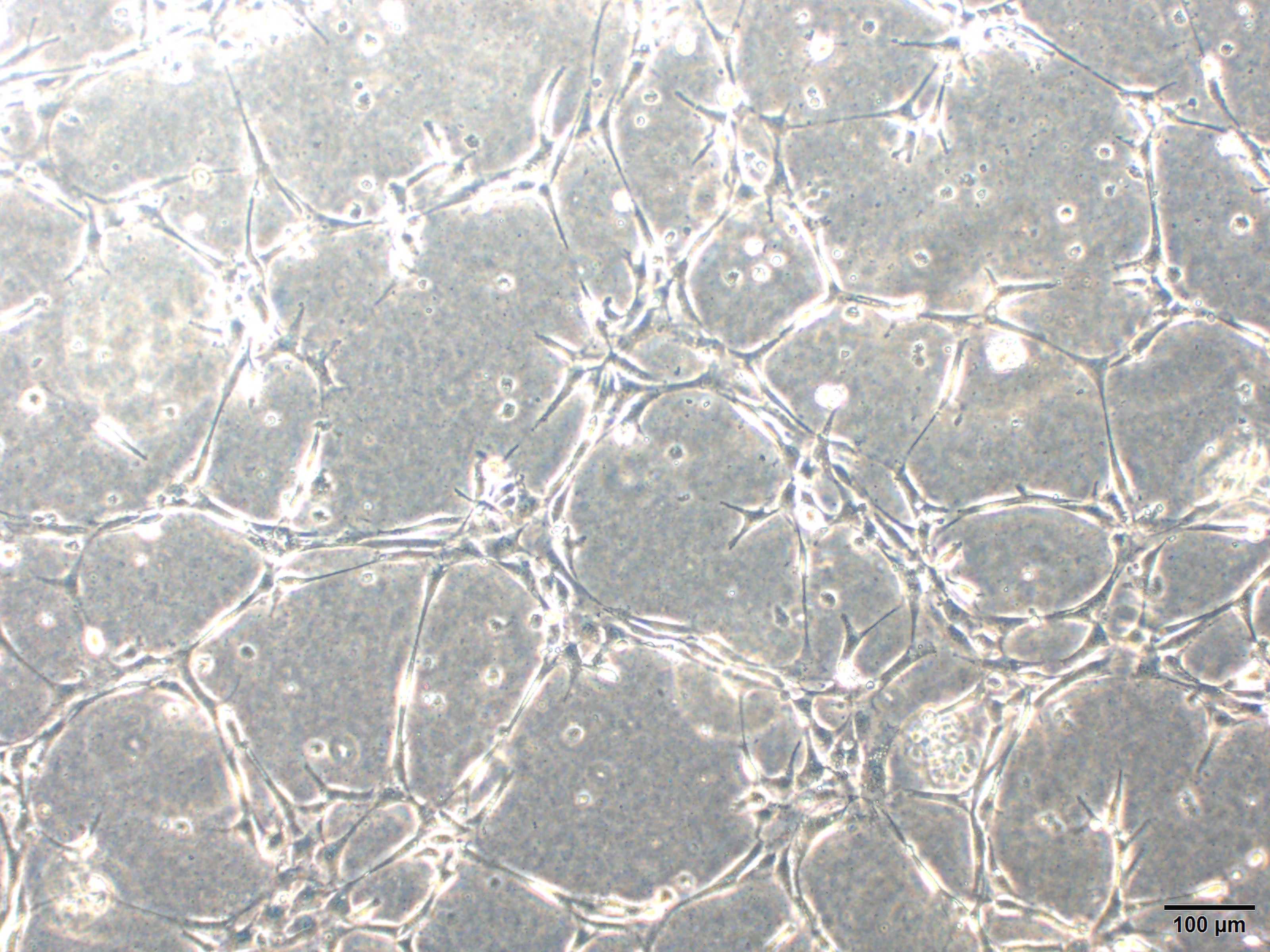

Supplement: Supplemental Information 1 [file peerj-13-19568-s001.zip › Figure 2A and 4C (angiogenesis)/control (1).jpg]

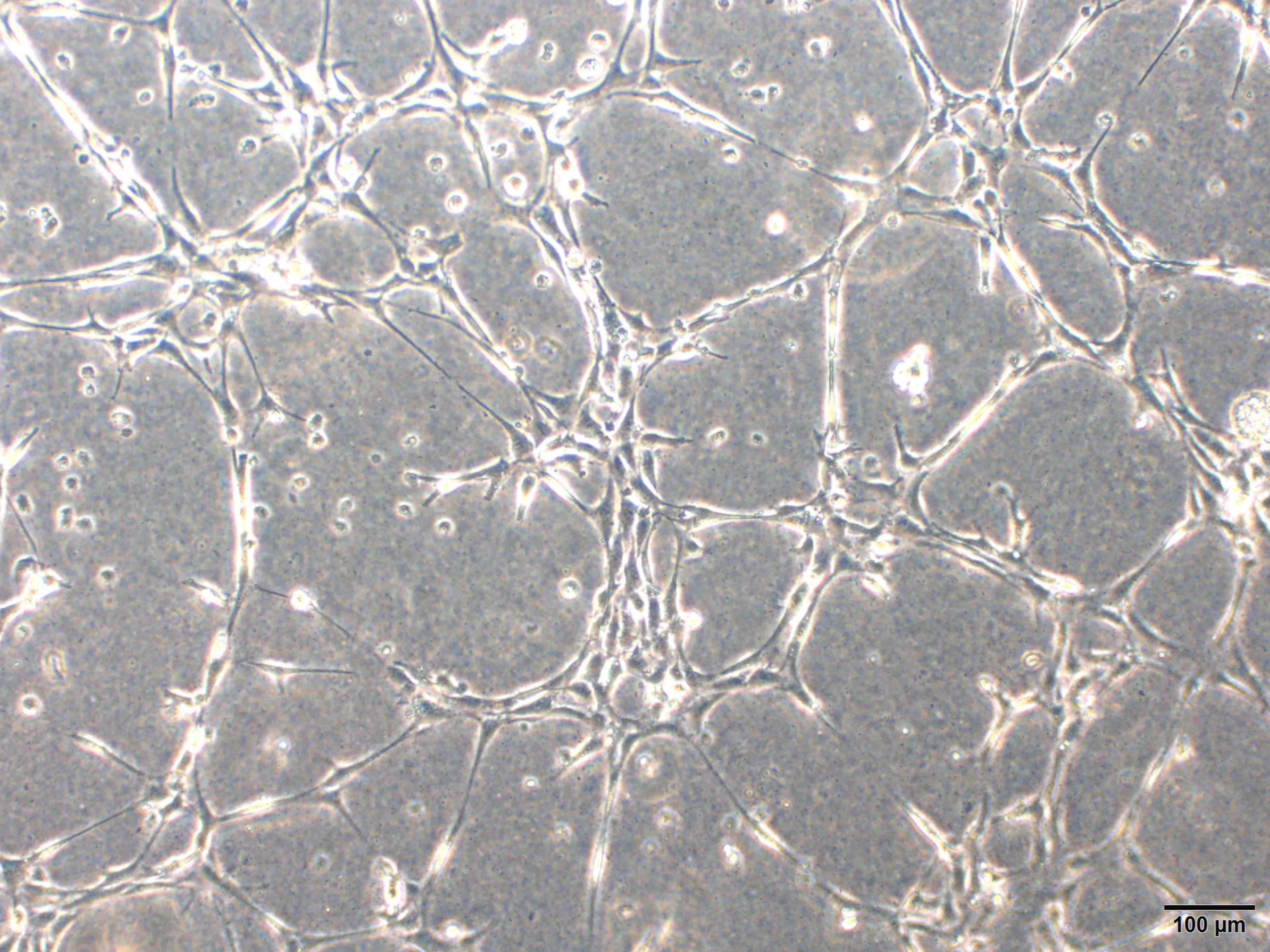

Supplement: Supplemental Information 1 [file peerj-13-19568-s001.zip › Figure 2A and 4C (angiogenesis)/control (2).jpg]

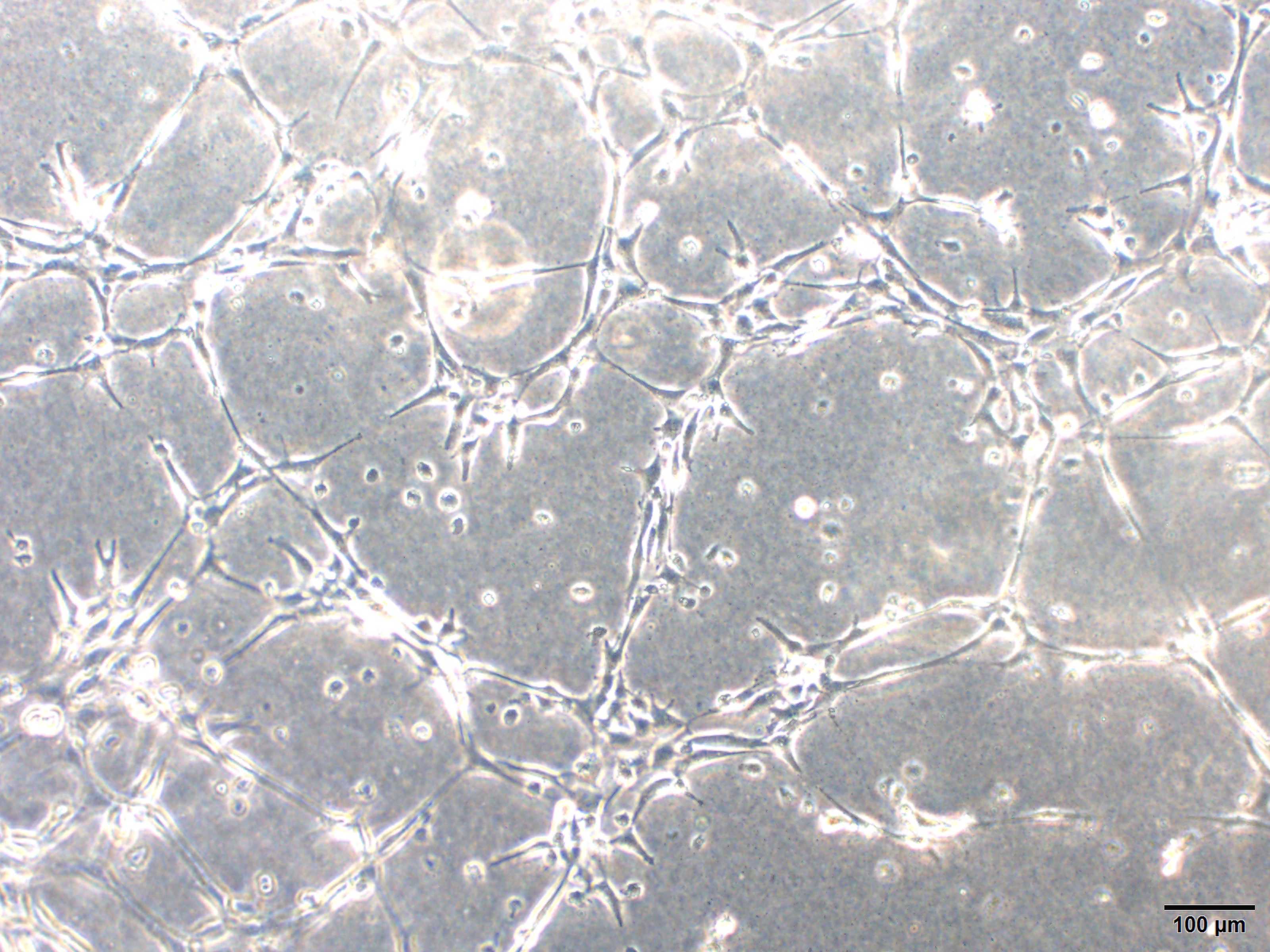

Supplement: Supplemental Information 1 [file peerj-13-19568-s001.zip › Figure 2A and 4C (angiogenesis)/control (3).jpg]

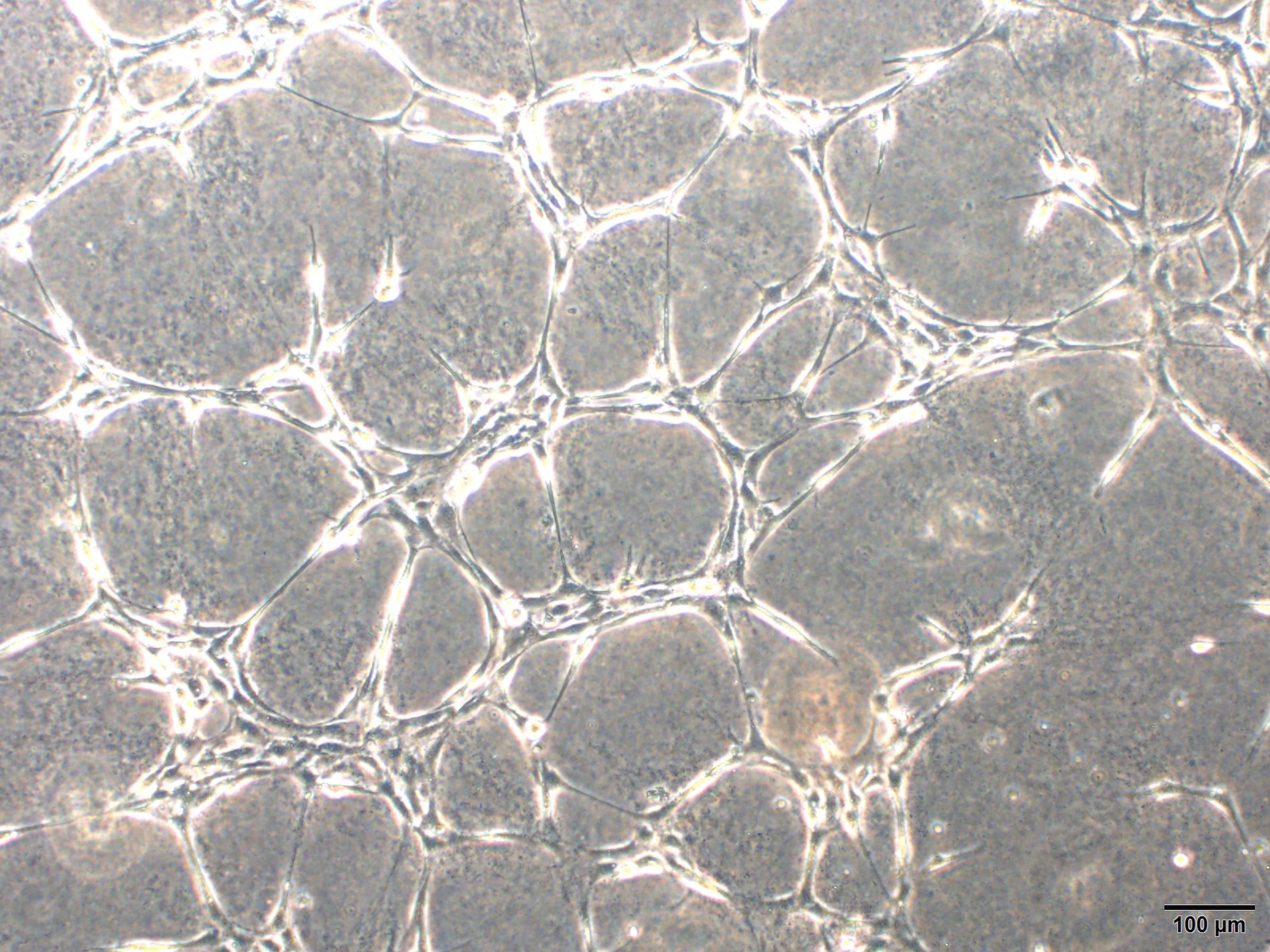

Supplement: Supplemental Information 1 [file peerj-13-19568-s001.zip › Figure 2A and 4C (angiogenesis)/miRNA inhibitors NC (1).jpg]

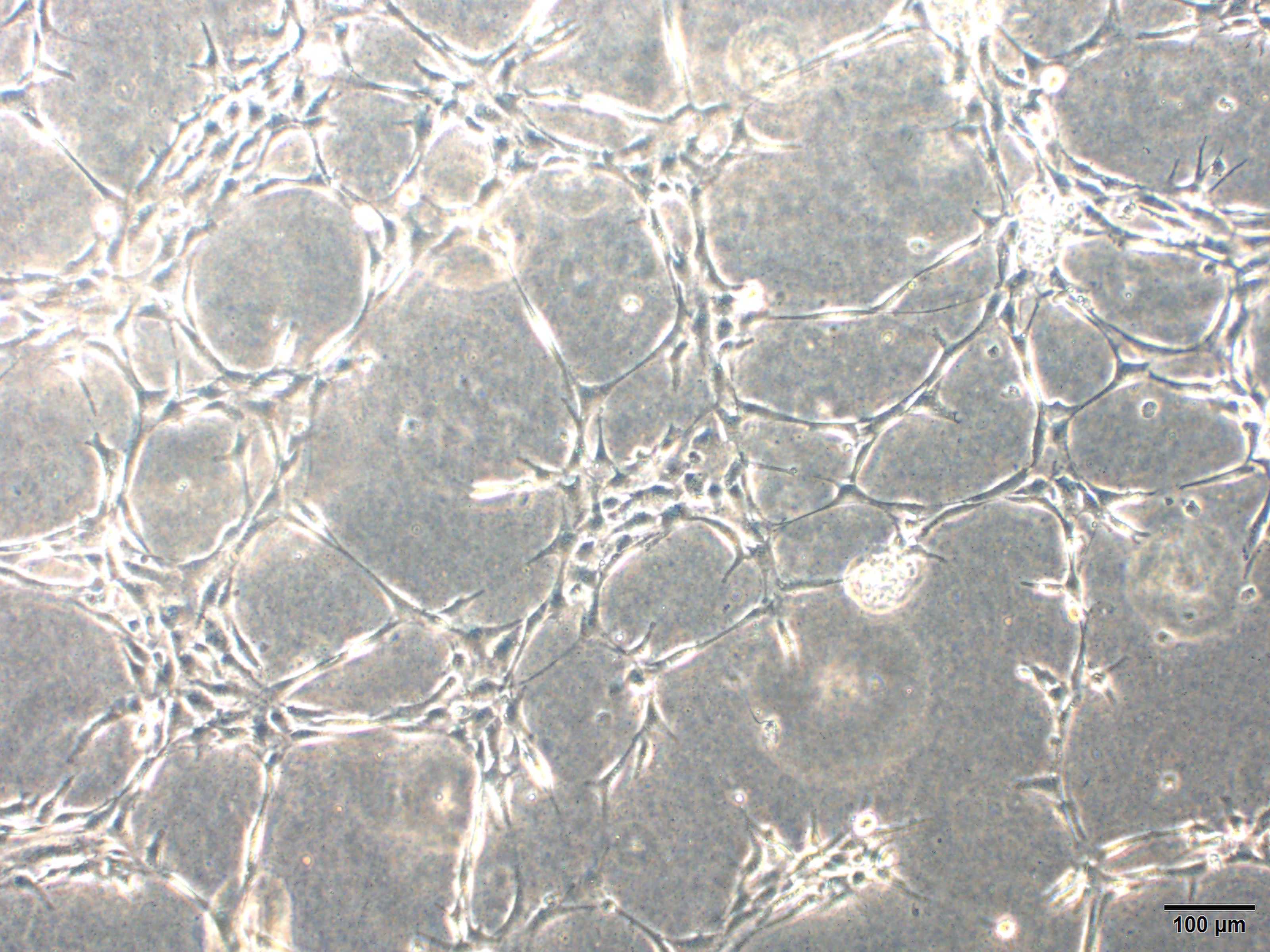

Supplement: Supplemental Information 1 [file peerj-13-19568-s001.zip › Figure 2A and 4C (angiogenesis)/miRNA inhibitors NC (2).jpg]

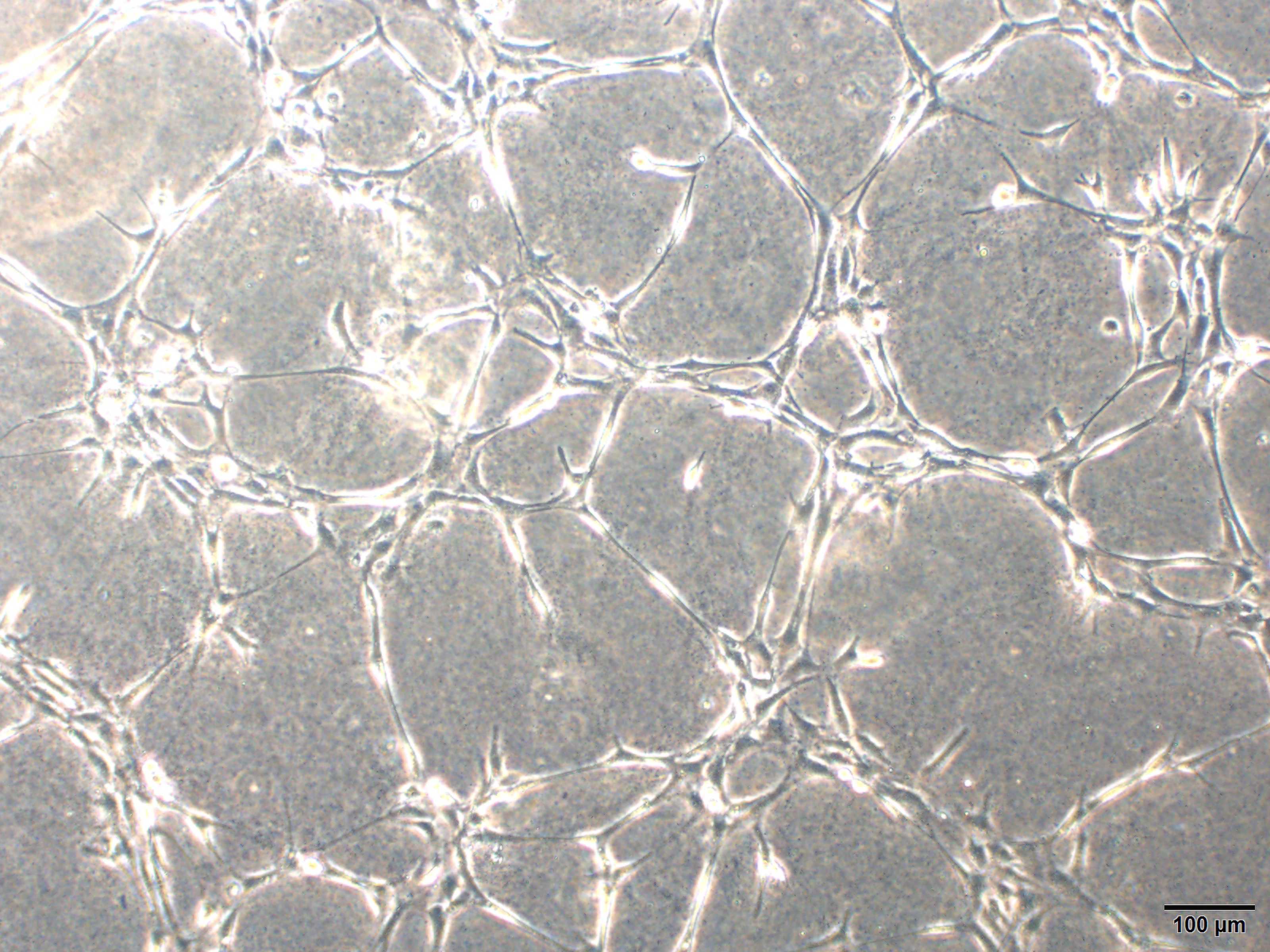

Supplement: Supplemental Information 1 [file peerj-13-19568-s001.zip › Figure 2A and 4C (angiogenesis)/miRNA inhibitors NC (3).jpg]

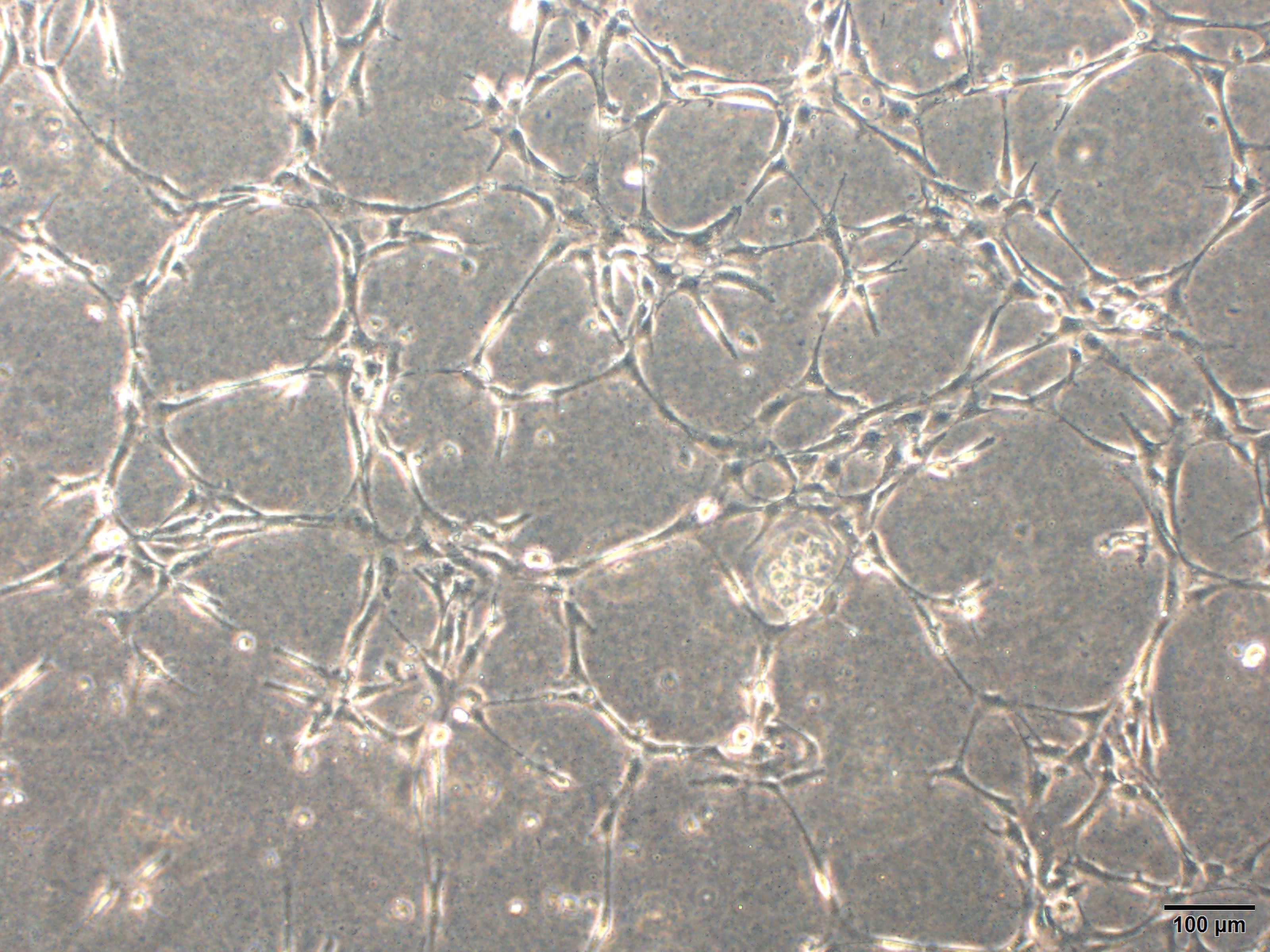

Supplement: Supplemental Information 1 [file peerj-13-19568-s001.zip › Figure 2A and 4C (angiogenesis)/miRNA mimics nc (1).jpg]

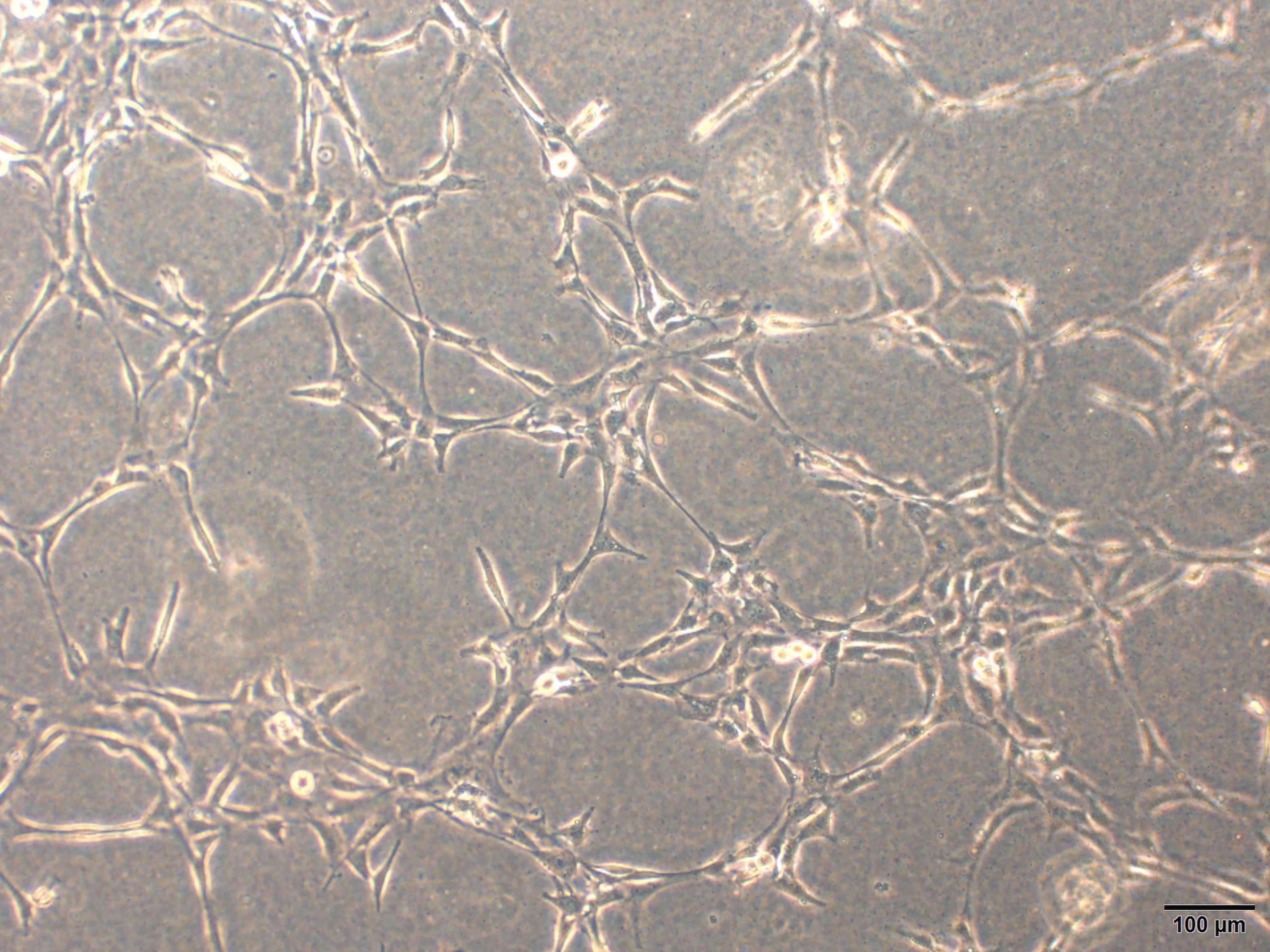

Supplement: Supplemental Information 1 [file peerj-13-19568-s001.zip › Figure 2A and 4C (angiogenesis)/miRNA mimics nc (2).jpg]

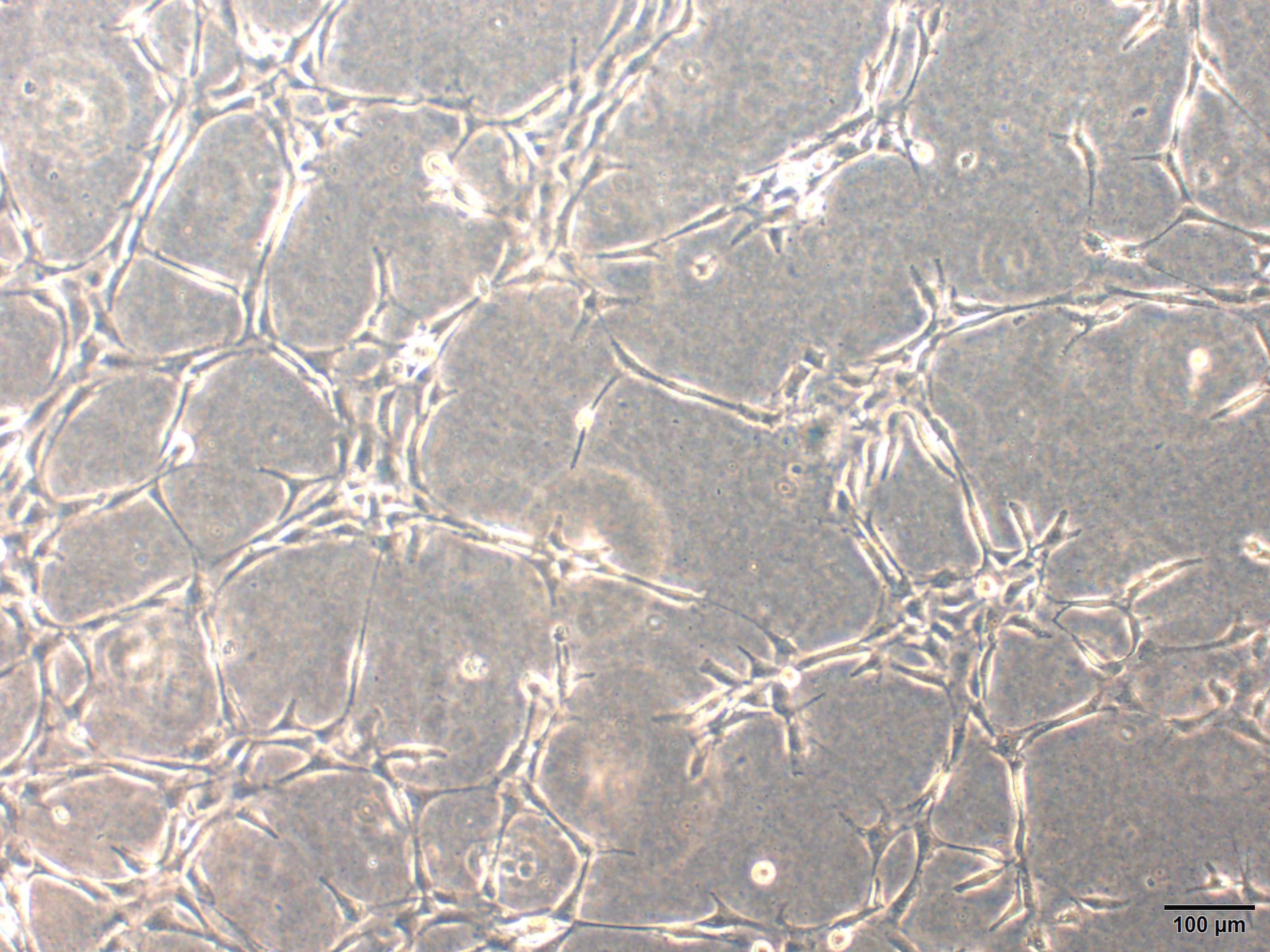

Supplement: Supplemental Information 1 [file peerj-13-19568-s001.zip › Figure 2A and 4C (angiogenesis)/miRNA mimics nc (3).jpg]

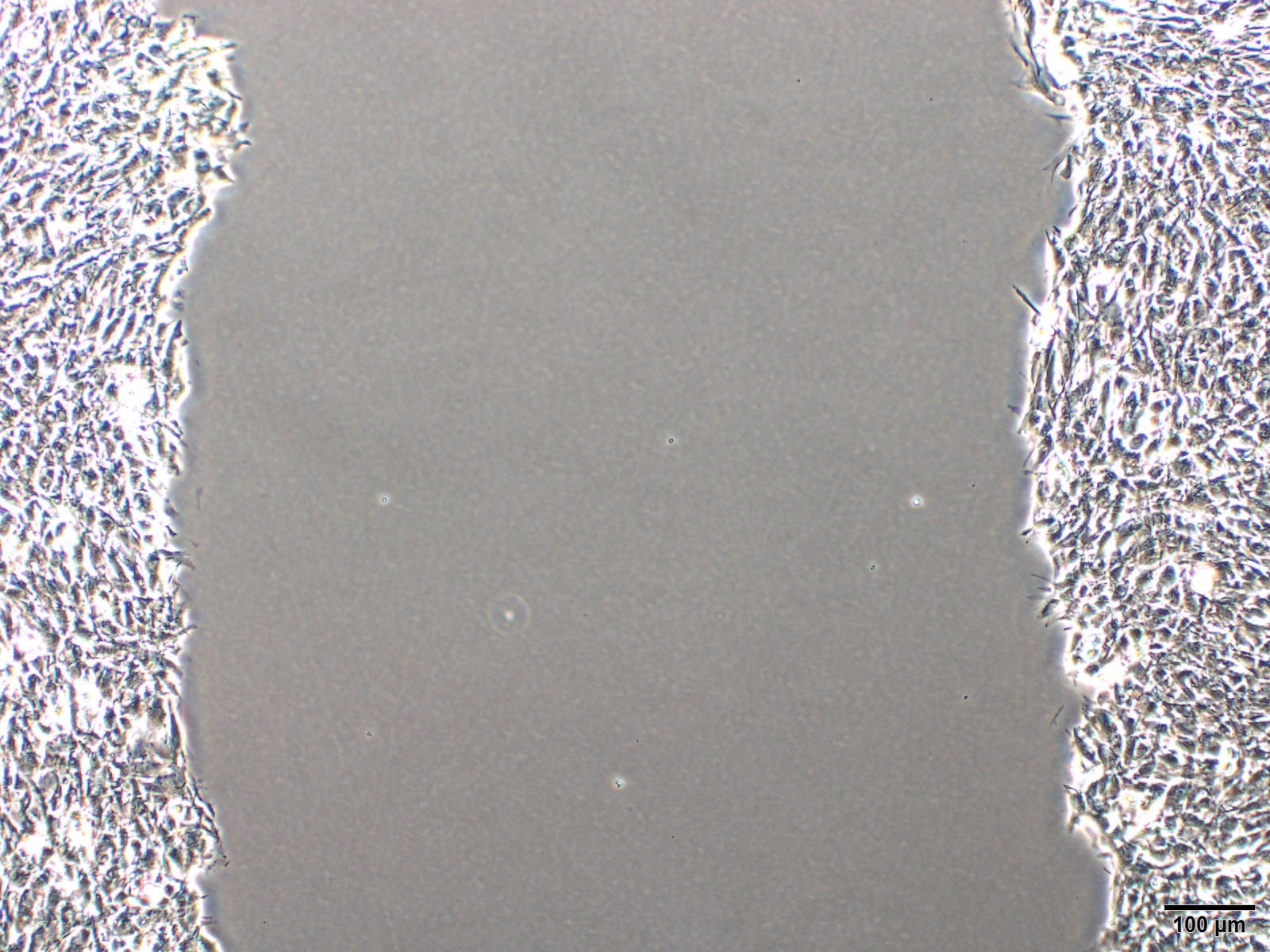

Supplement: Supplemental Information 2 [file peerj-13-19568-s002.zip › Figure 2A and 4C (Wound healing)/0h/control (1).jpg]

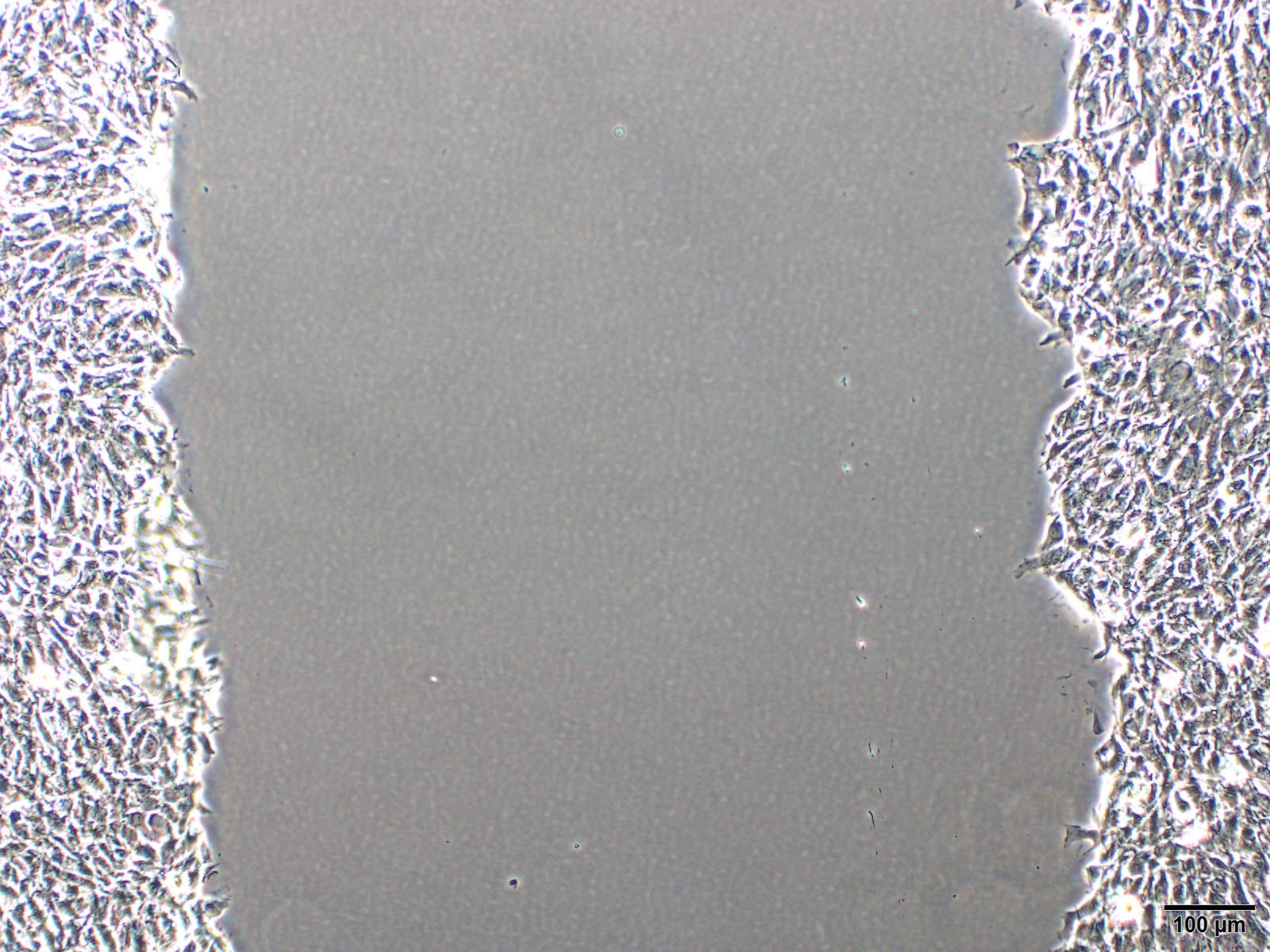

Supplement: Supplemental Information 2 [file peerj-13-19568-s002.zip › Figure 2A and 4C (Wound healing)/0h/control (2).jpg]

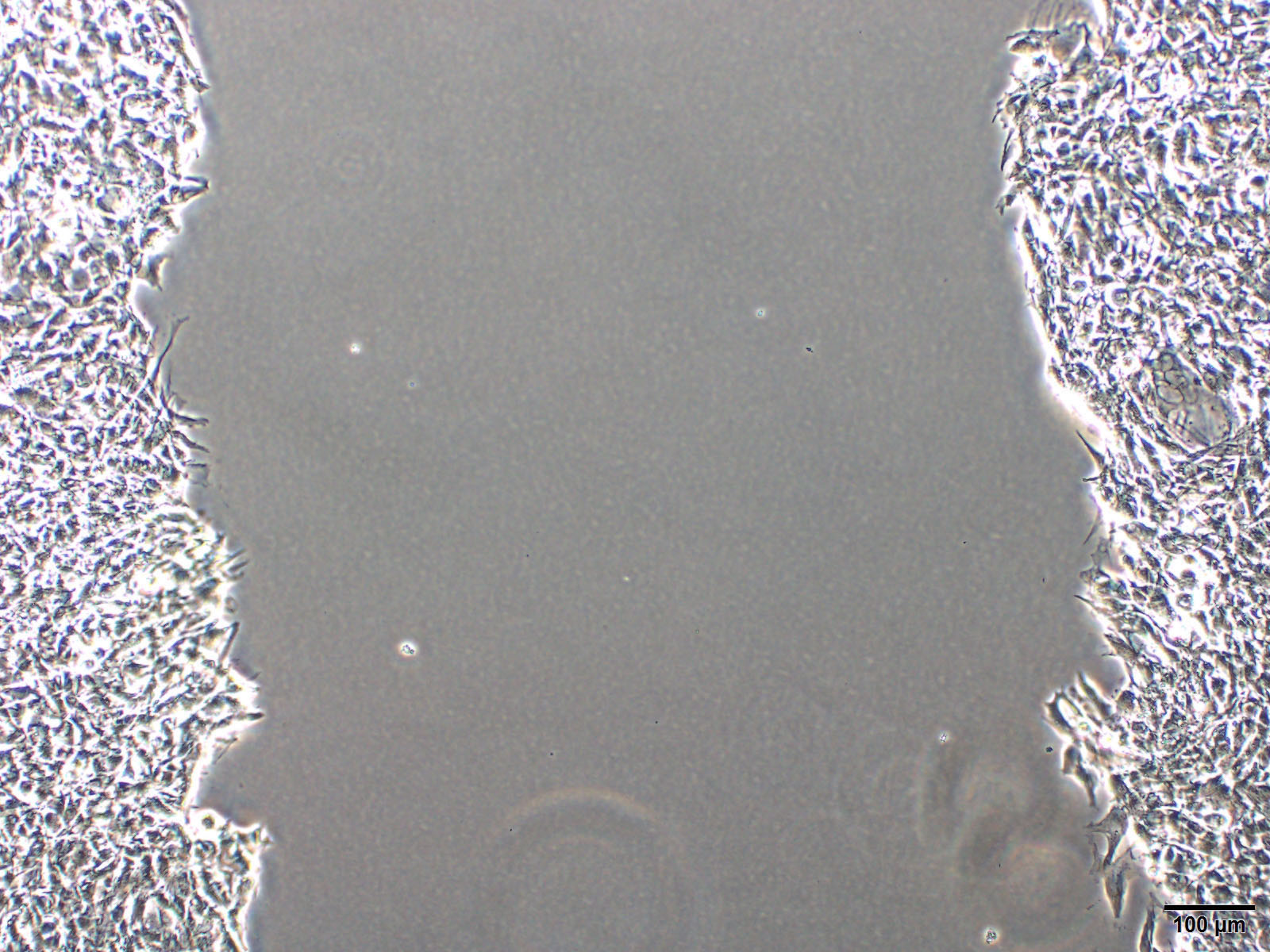

Supplement: Supplemental Information 2 [file peerj-13-19568-s002.zip › Figure 2A and 4C (Wound healing)/0h/control (3).jpg]

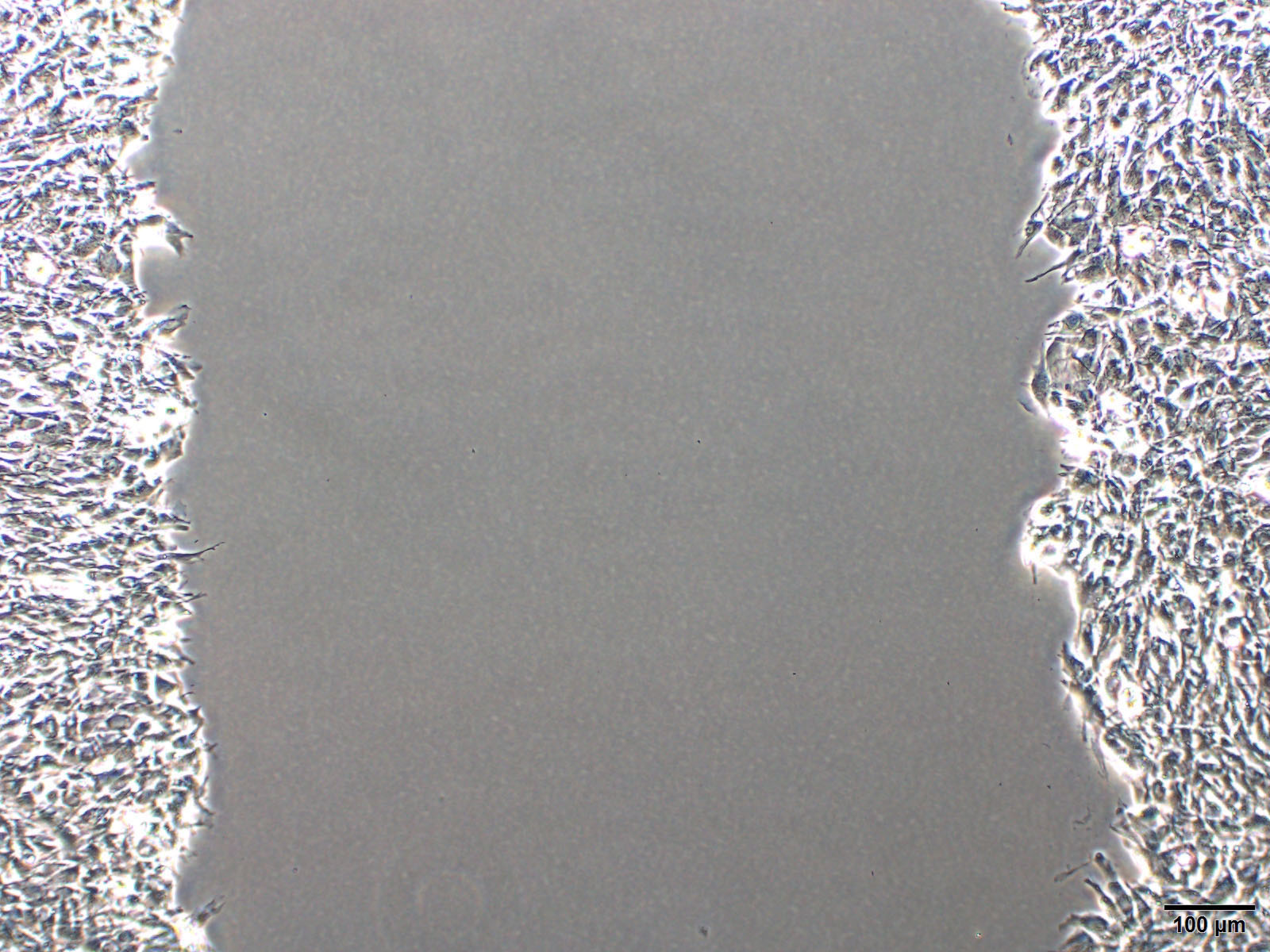

Supplement: Supplemental Information 2 [file peerj-13-19568-s002.zip › Figure 2A and 4C (Wound healing)/0h/miRNA inhibitor NC (1).jpg]

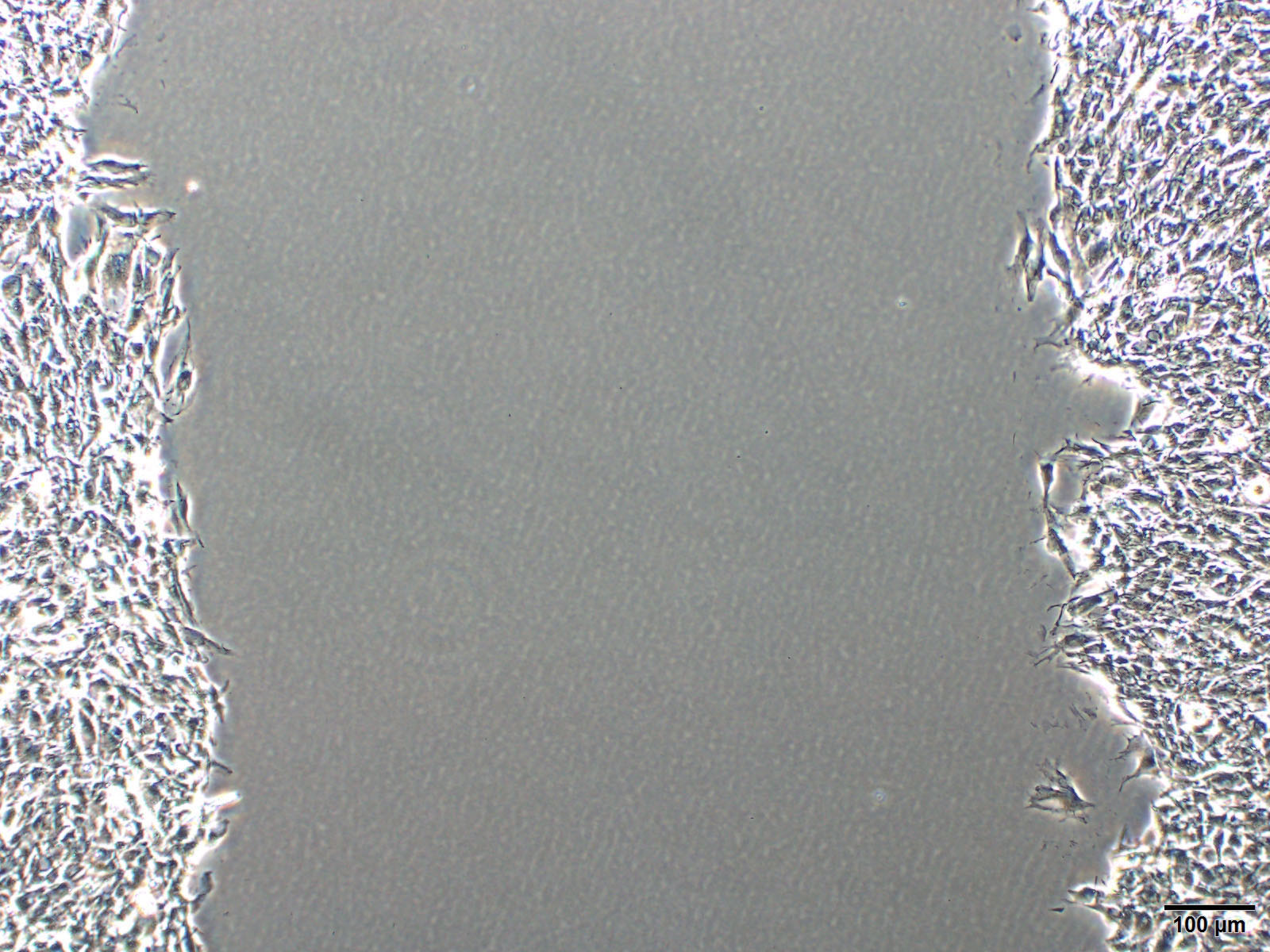

Supplement: Supplemental Information 2 [file peerj-13-19568-s002.zip › Figure 2A and 4C (Wound healing)/0h/miRNA inhibitor NC (2).jpg]

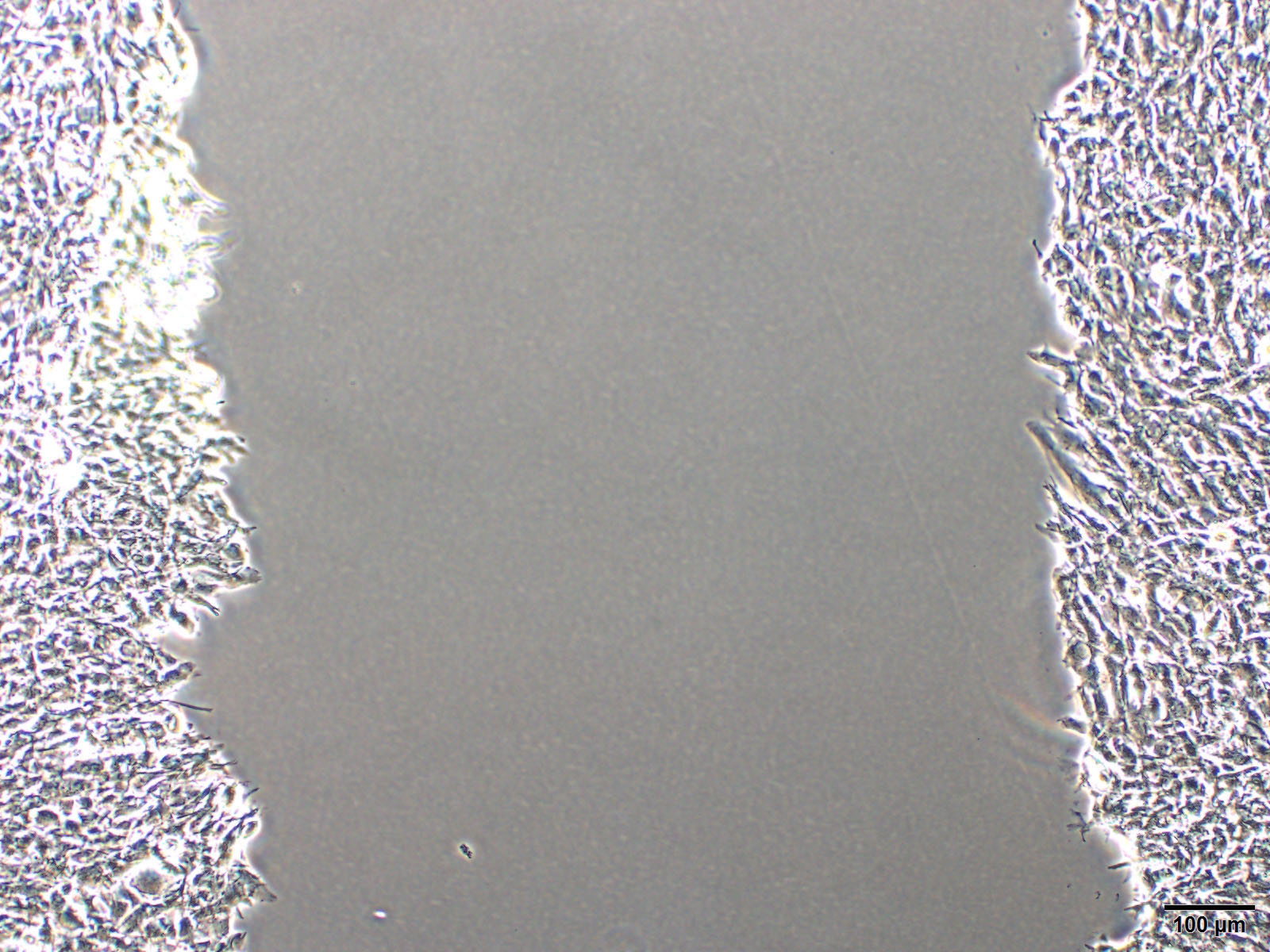

Supplement: Supplemental Information 2 [file peerj-13-19568-s002.zip › Figure 2A and 4C (Wound healing)/0h/miRNA inhibitor NC (3).jpg]

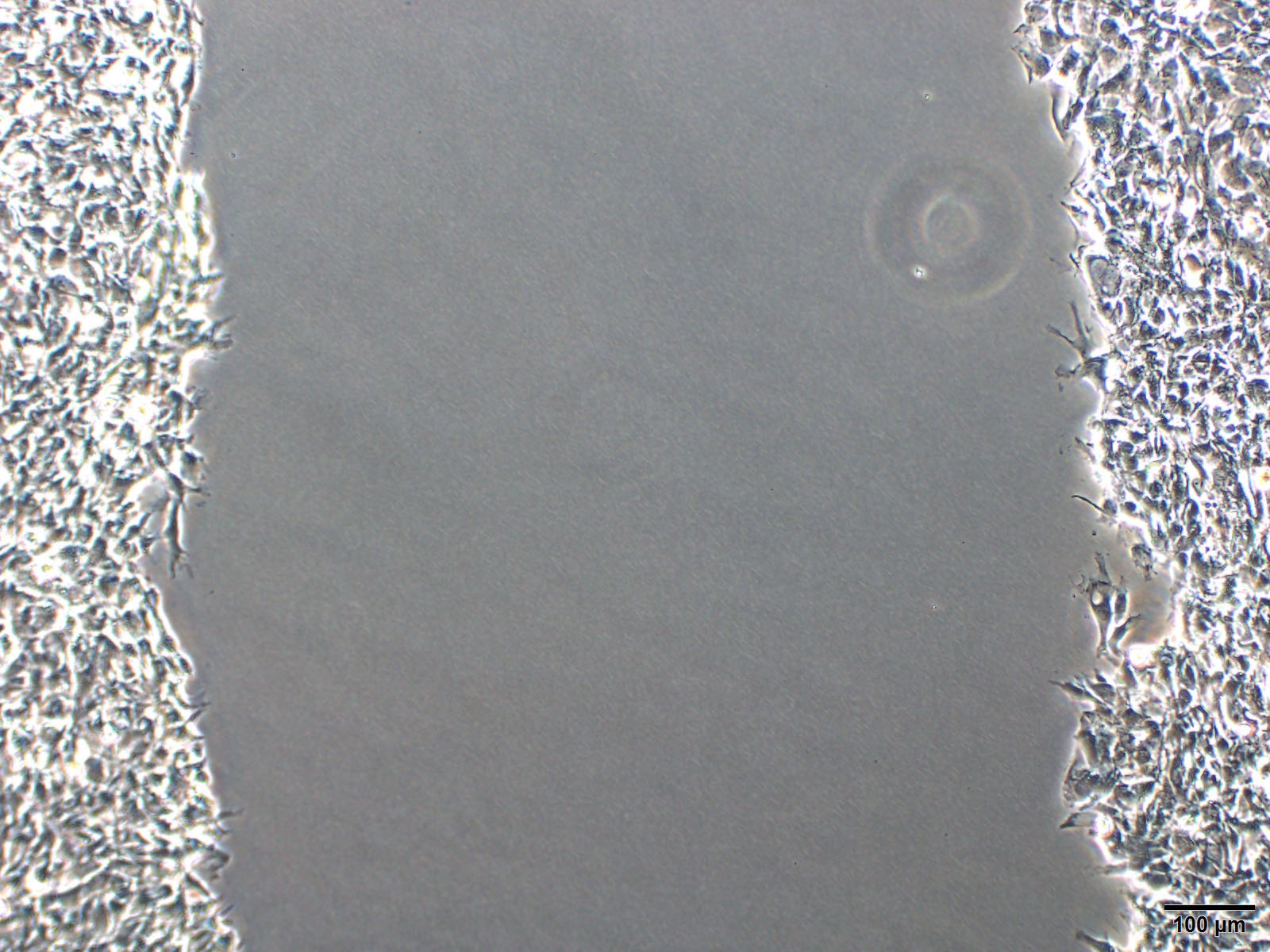

Supplement: Supplemental Information 2 [file peerj-13-19568-s002.zip › Figure 2A and 4C (Wound healing)/0h/miRNA inhibitors (1).jpg]

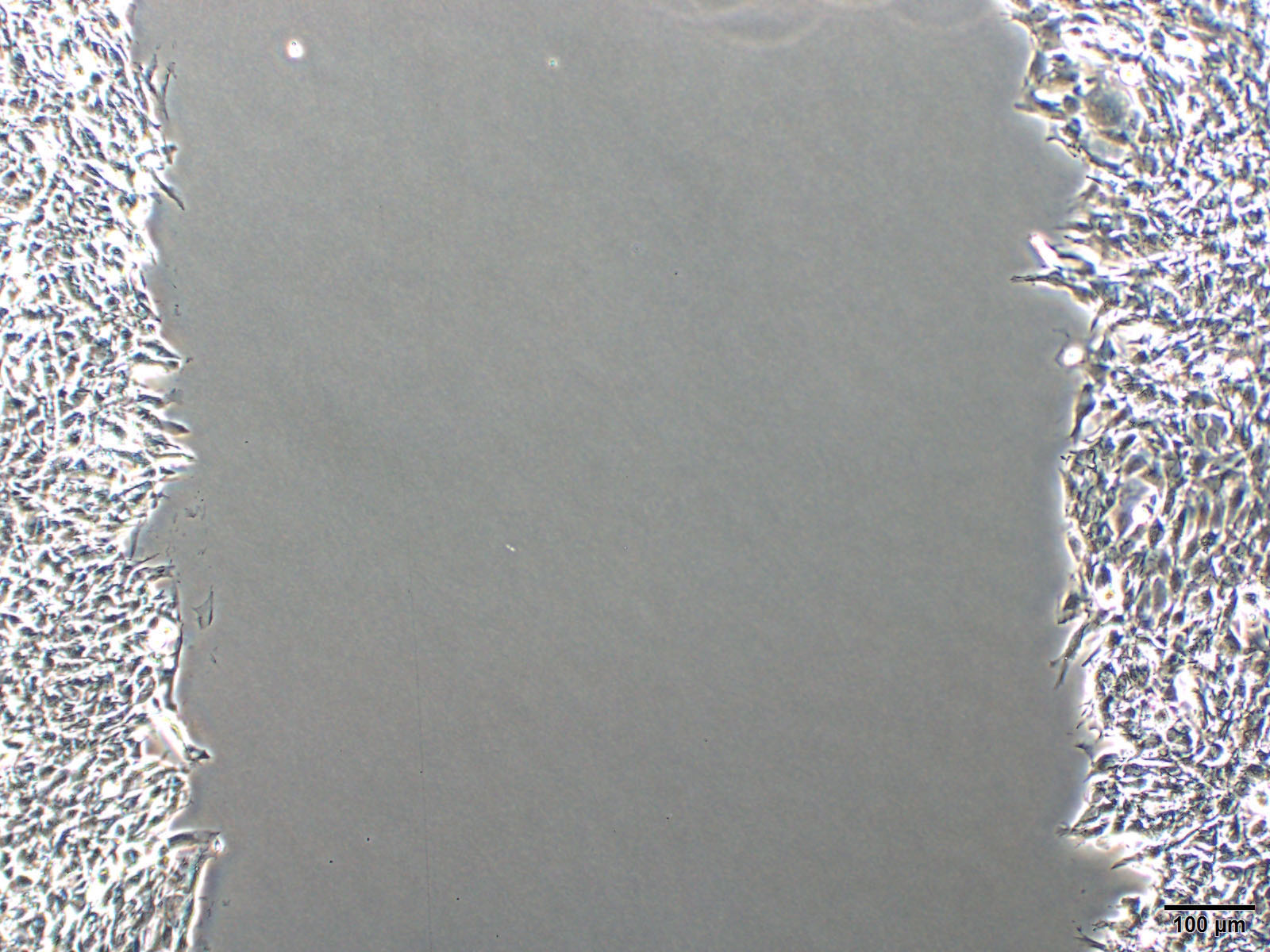

Supplement: Supplemental Information 2 [file peerj-13-19568-s002.zip › Figure 2A and 4C (Wound healing)/0h/miRNA inhibitors (2).jpg]

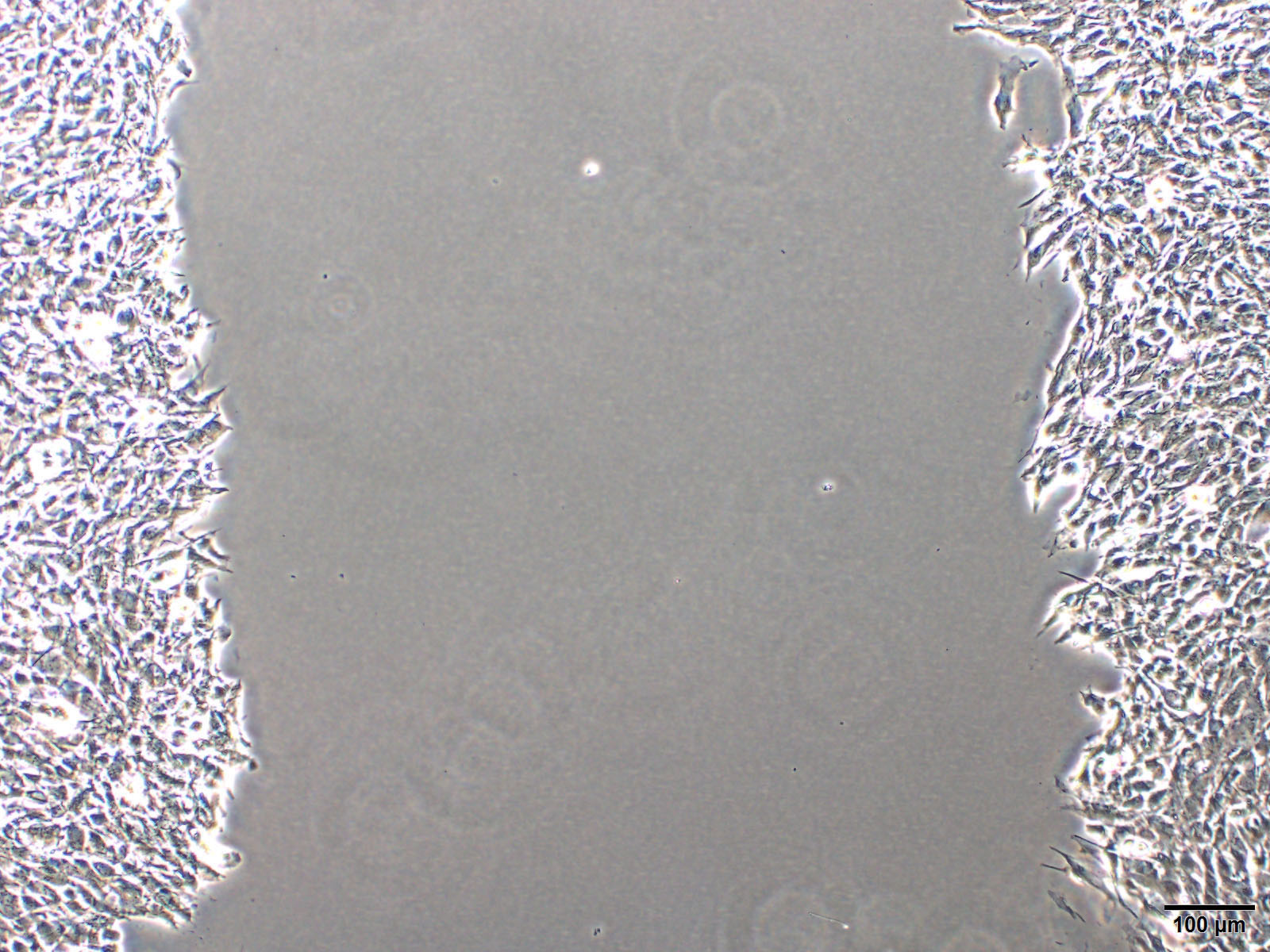

Supplement: Supplemental Information 2 [file peerj-13-19568-s002.zip › Figure 2A and 4C (Wound healing)/0h/miRNA inhibitors (3).jpg]

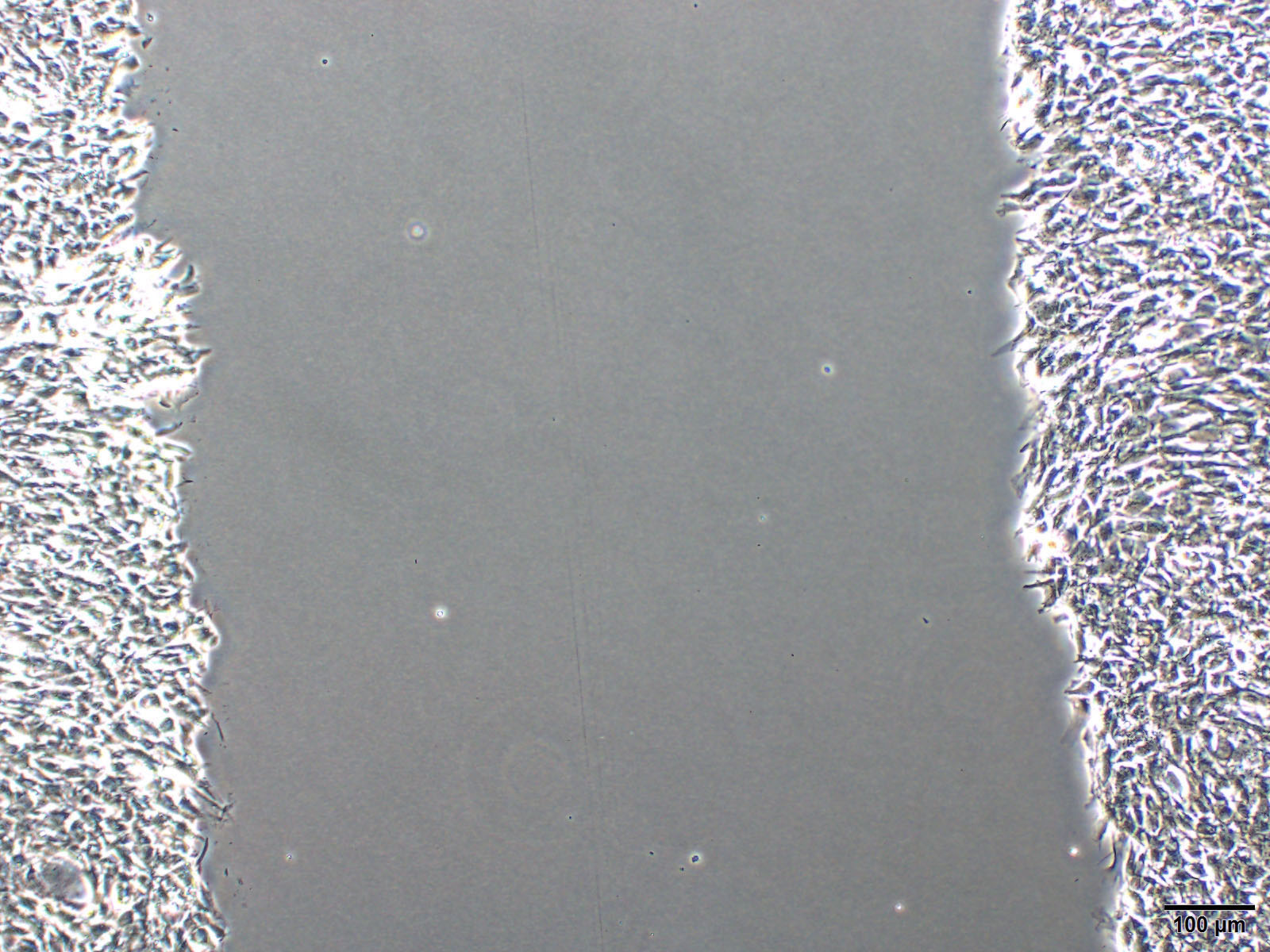

Supplement: Supplemental Information 2 [file peerj-13-19568-s002.zip › Figure 2A and 4C (Wound healing)/0h/miRNA inhibitors +si-NC (1).jpg]

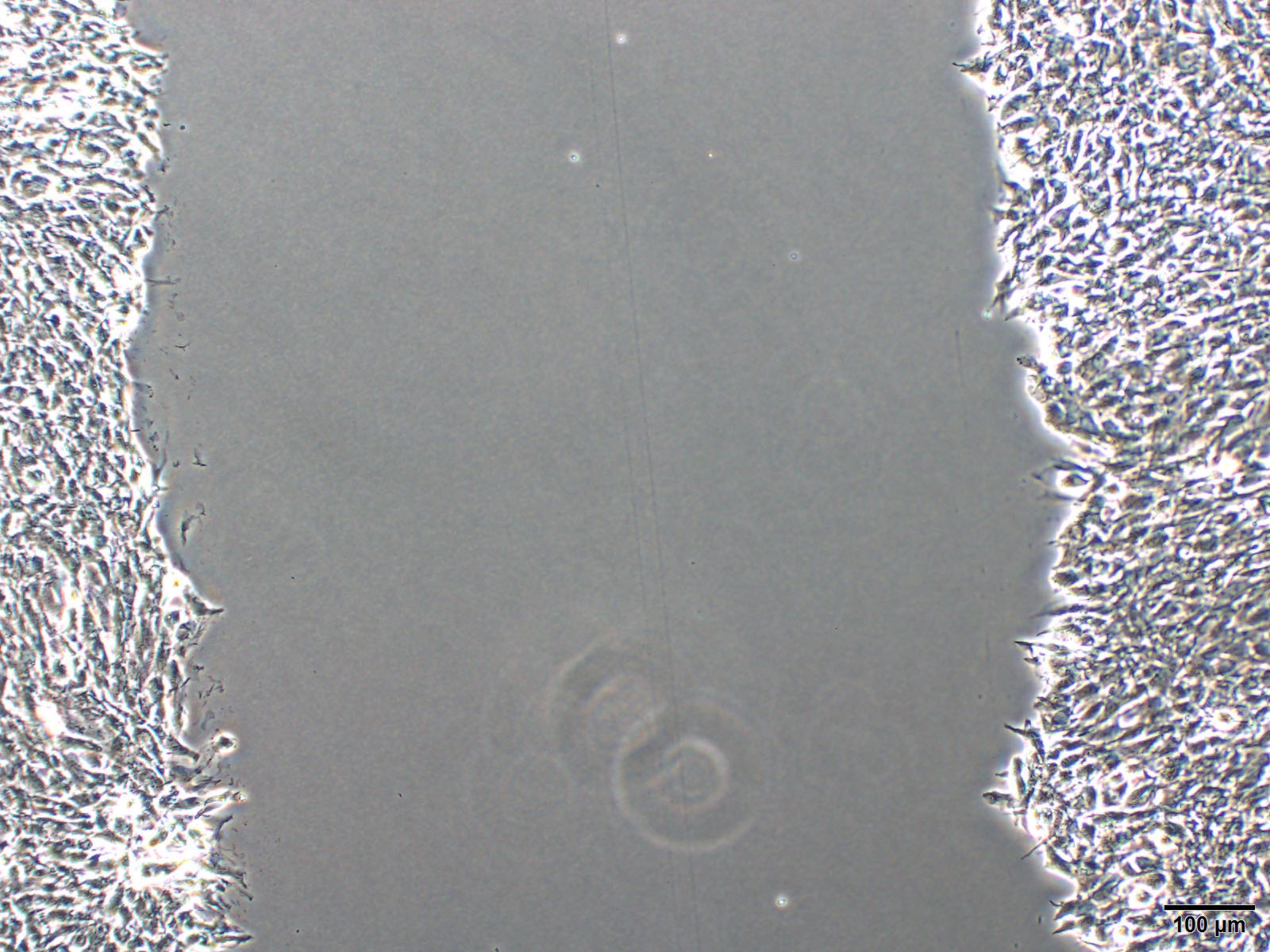

Supplement: Supplemental Information 2 [file peerj-13-19568-s002.zip › Figure 2A and 4C (Wound healing)/0h/miRNA inhibitors +si-NC (2).jpg]

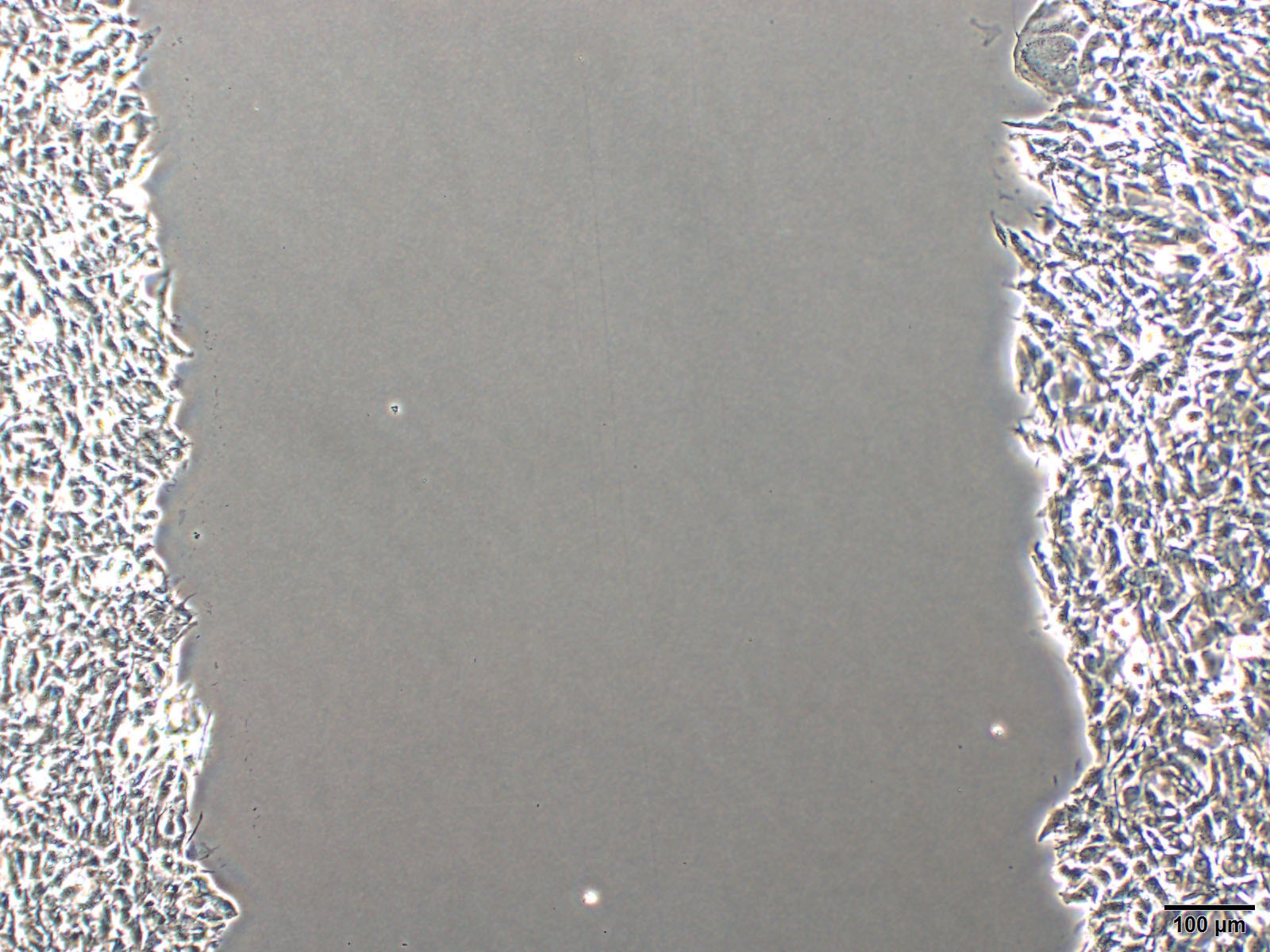

Supplement: Supplemental Information 2 [file peerj-13-19568-s002.zip › Figure 2A and 4C (Wound healing)/0h/miRNA inhibitors +si-NC (3).jpg]

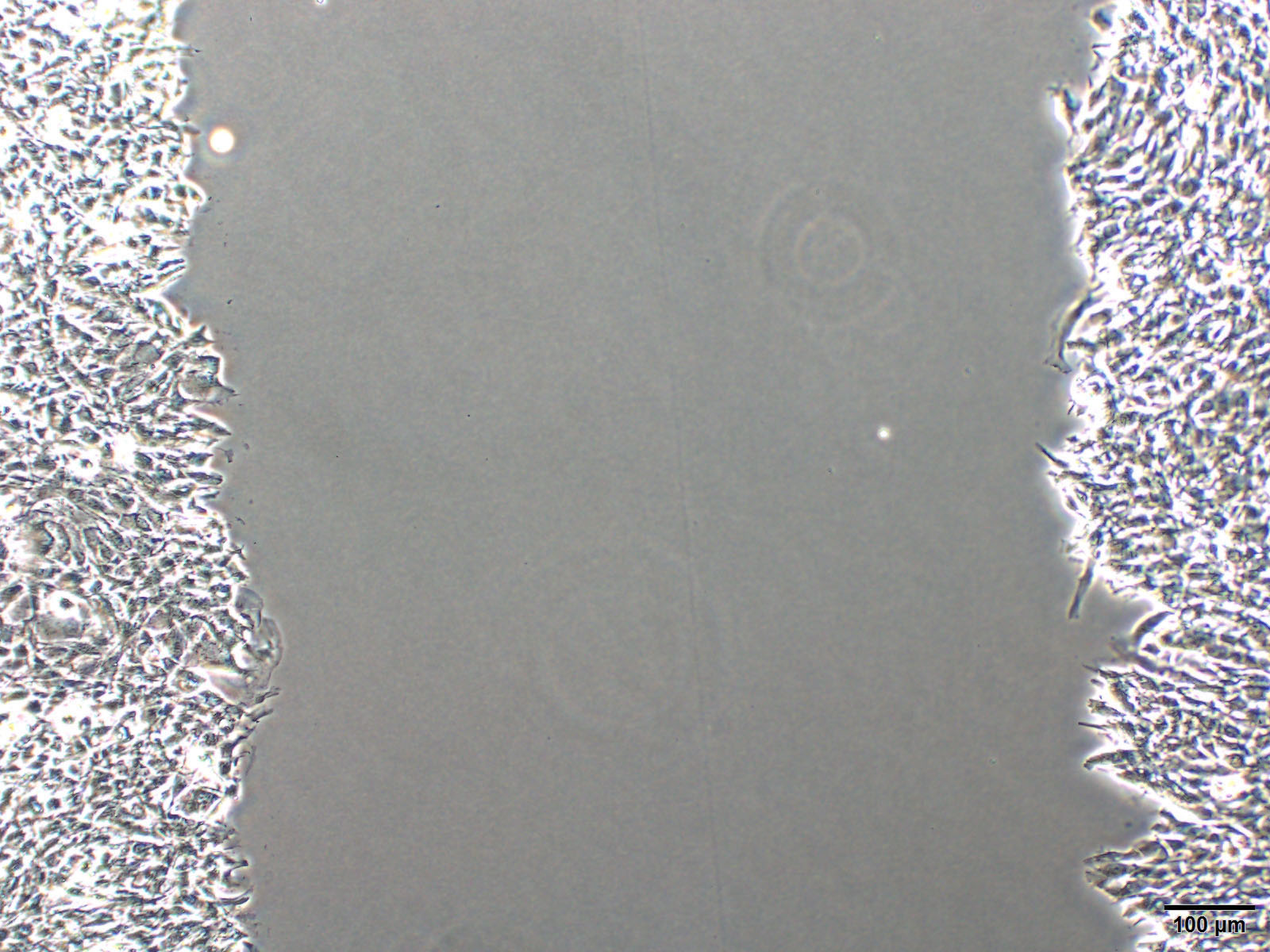

Supplement: Supplemental Information 2 [file peerj-13-19568-s002.zip › Figure 2A and 4C (Wound healing)/0h/miRNA inhibitors +si-VEGF (1).jpg]

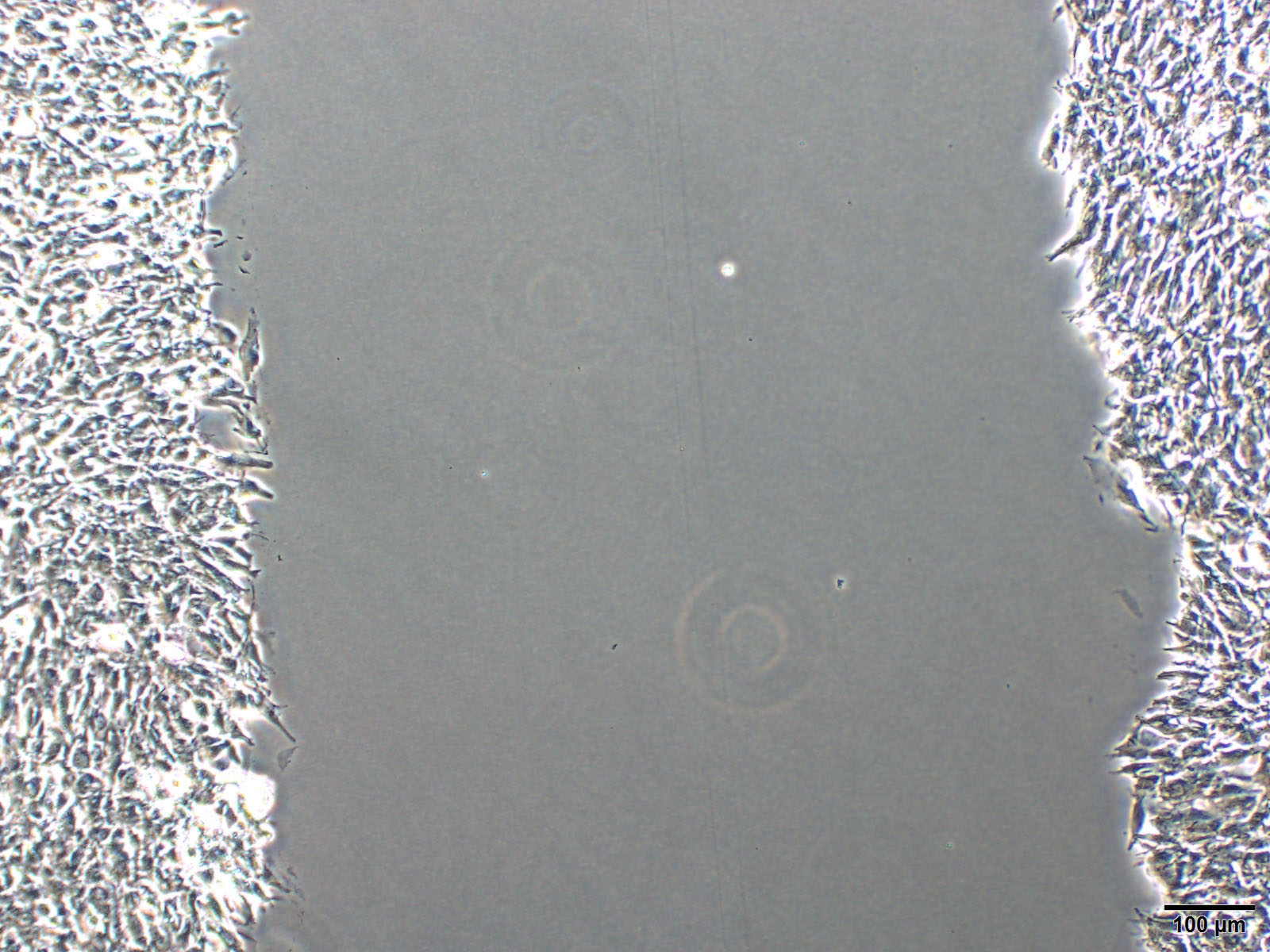

Supplement: Supplemental Information 2 [file peerj-13-19568-s002.zip › Figure 2A and 4C (Wound healing)/0h/miRNA inhibitors +si-VEGF (2).jpg]

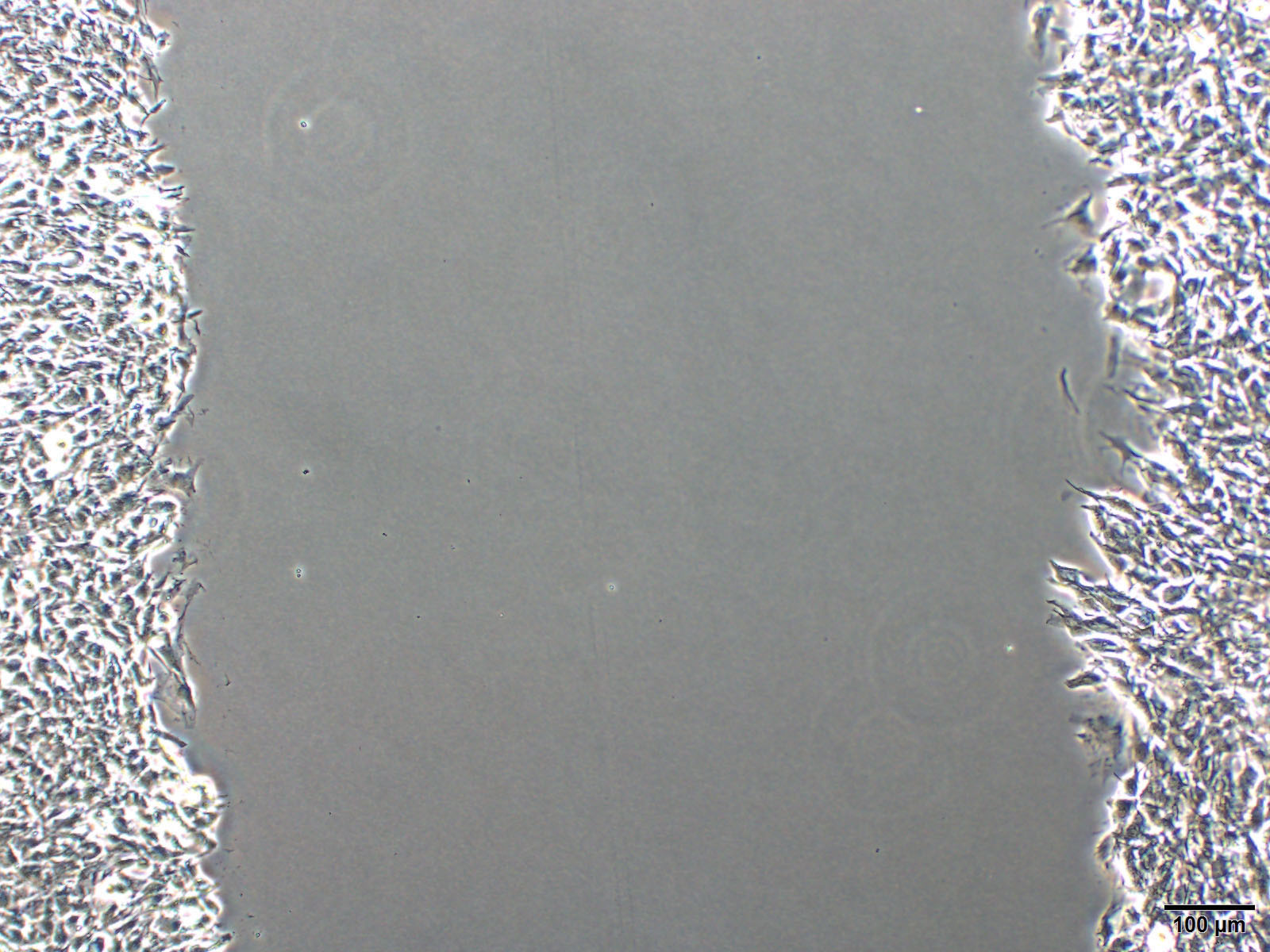

Supplement: Supplemental Information 2 [file peerj-13-19568-s002.zip › Figure 2A and 4C (Wound healing)/0h/miRNA inhibitors +si-VEGF (3).jpg]

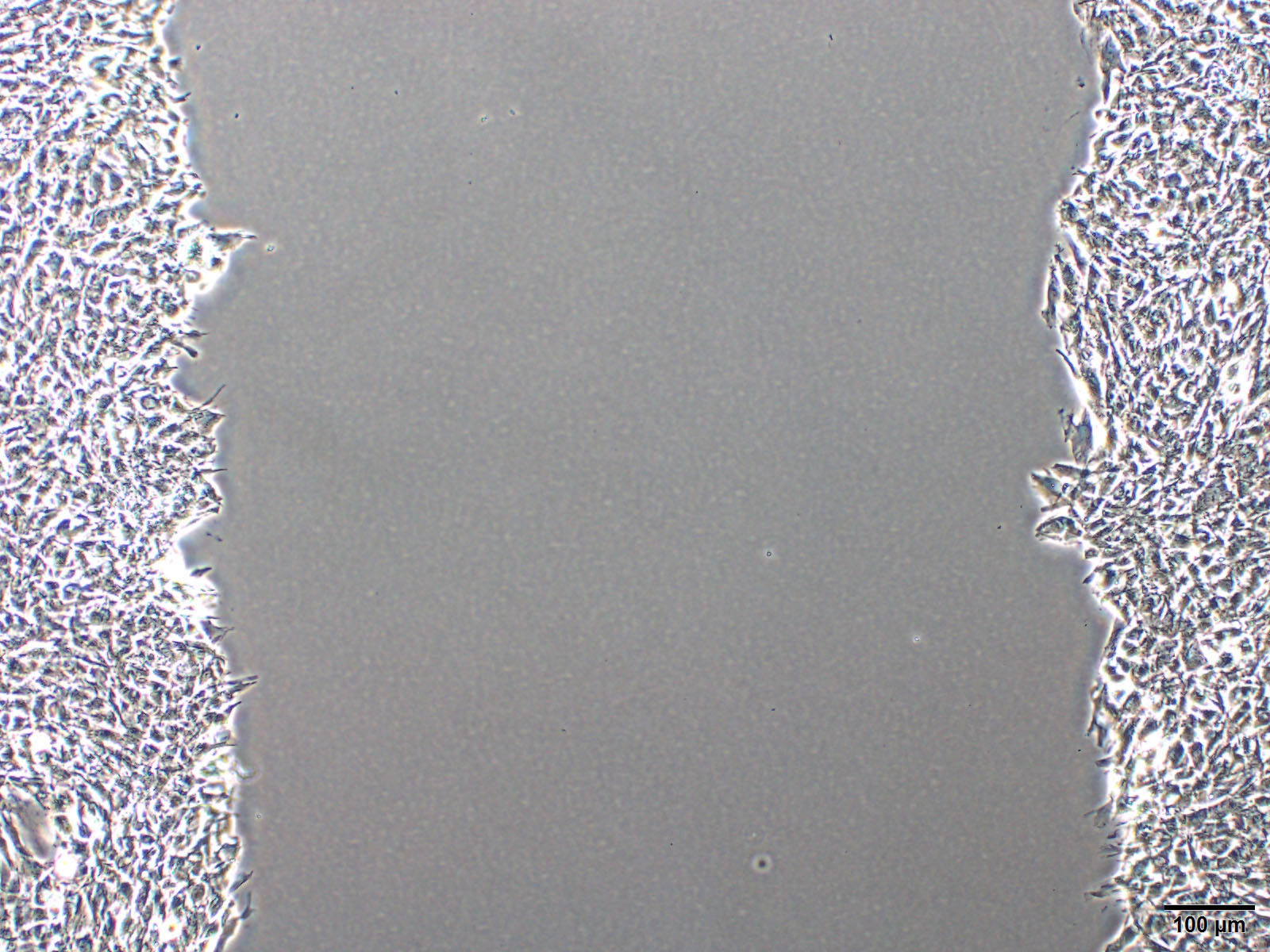

Supplement: Supplemental Information 2 [file peerj-13-19568-s002.zip › Figure 2A and 4C (Wound healing)/0h/miRNA mimics (1).jpg]

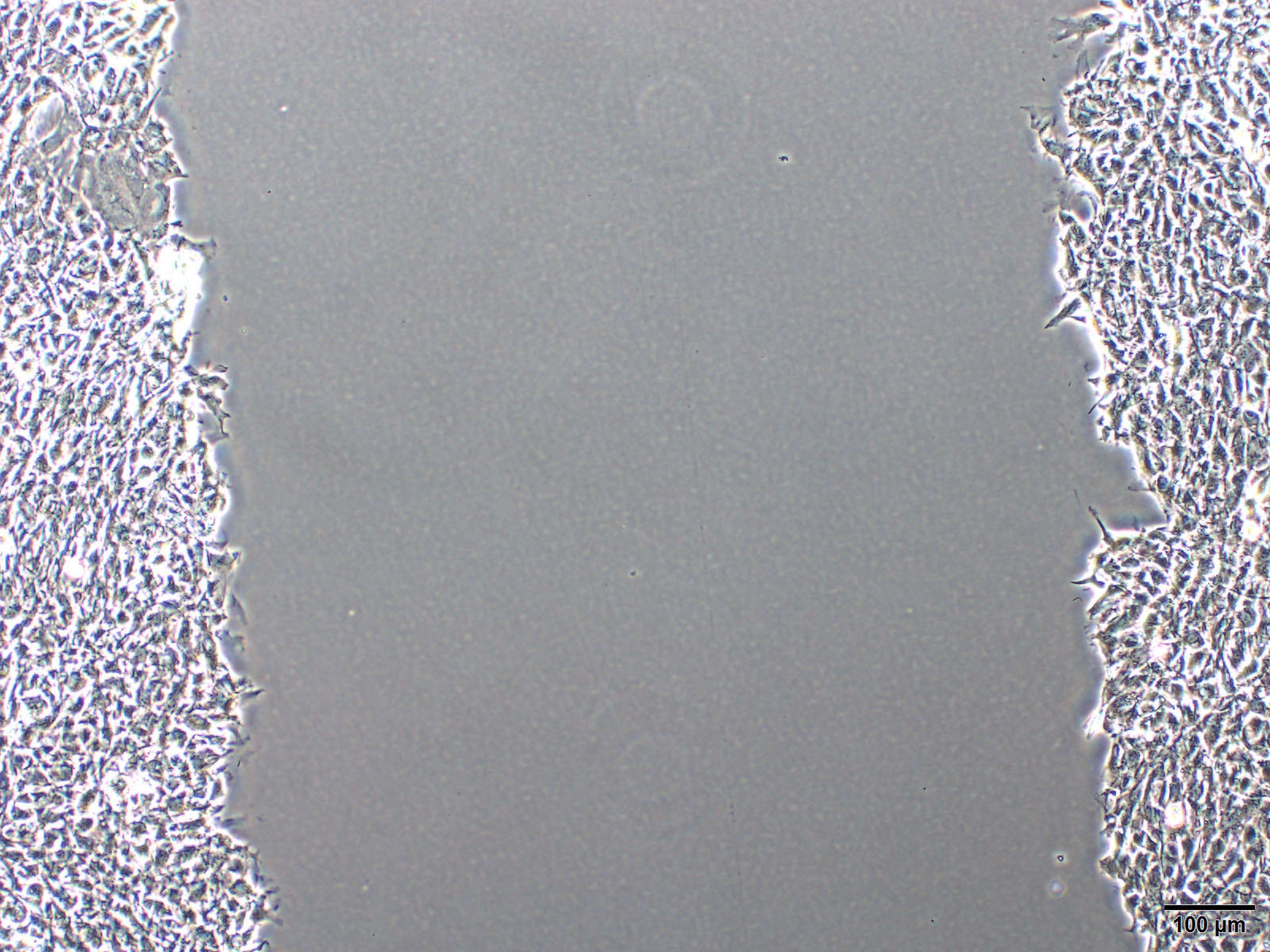

Supplement: Supplemental Information 2 [file peerj-13-19568-s002.zip › Figure 2A and 4C (Wound healing)/0h/miRNA mimics (2).jpg]

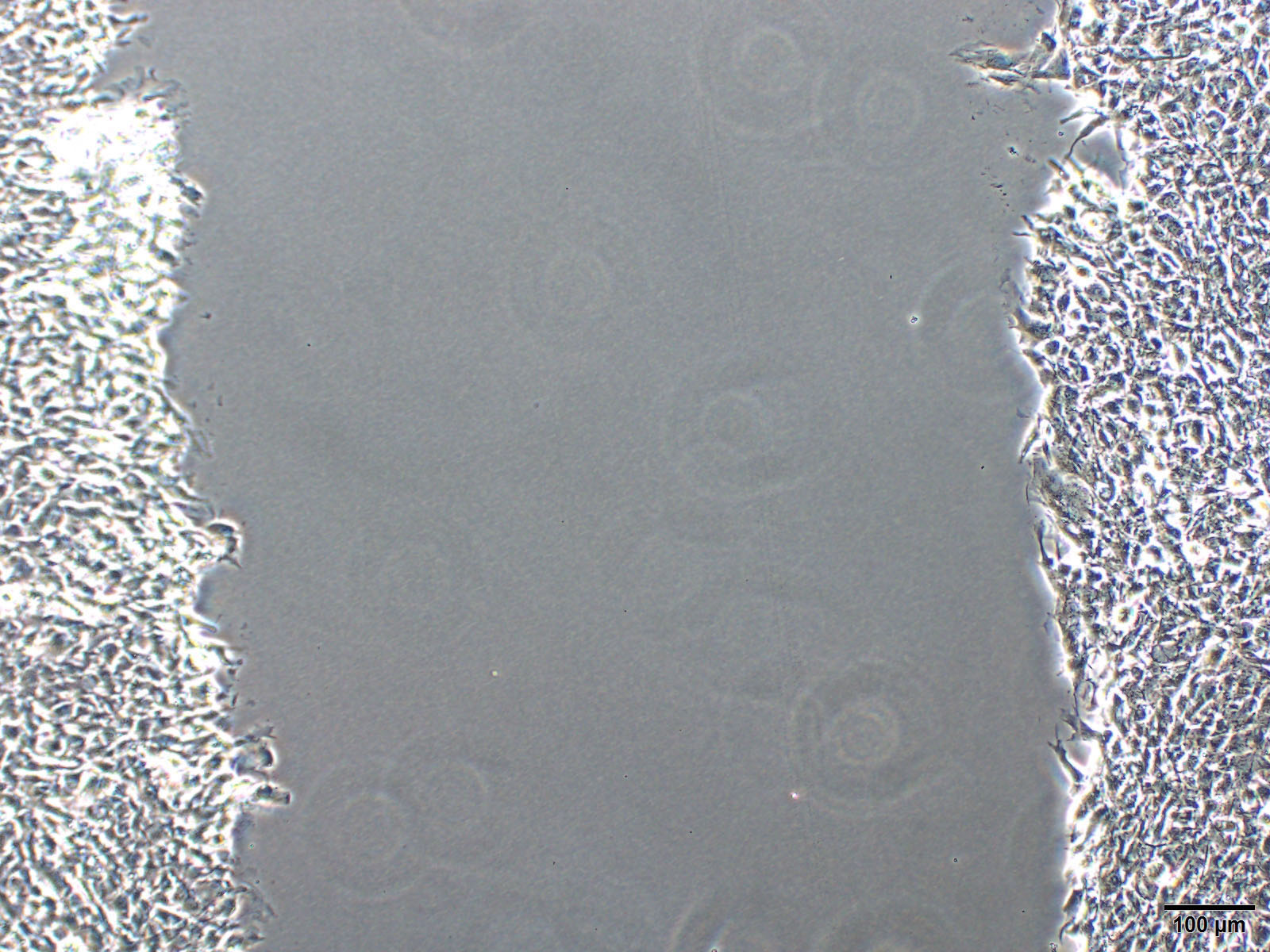

Supplement: Supplemental Information 2 [file peerj-13-19568-s002.zip › Figure 2A and 4C (Wound healing)/0h/miRNA mimics (3).jpg]

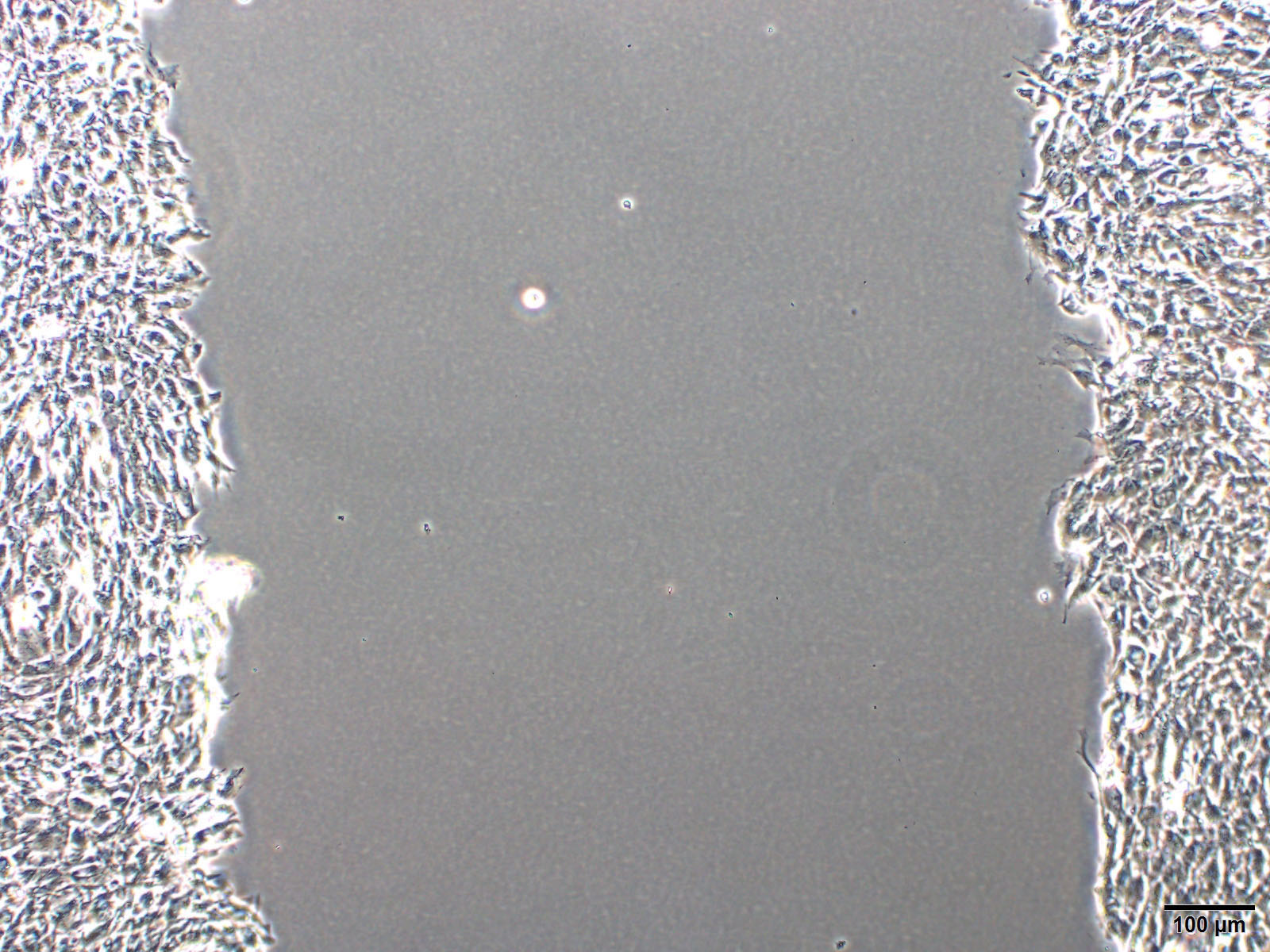

Supplement: Supplemental Information 2 [file peerj-13-19568-s002.zip › Figure 2A and 4C (Wound healing)/0h/miRNA mimics NC (1).jpg]

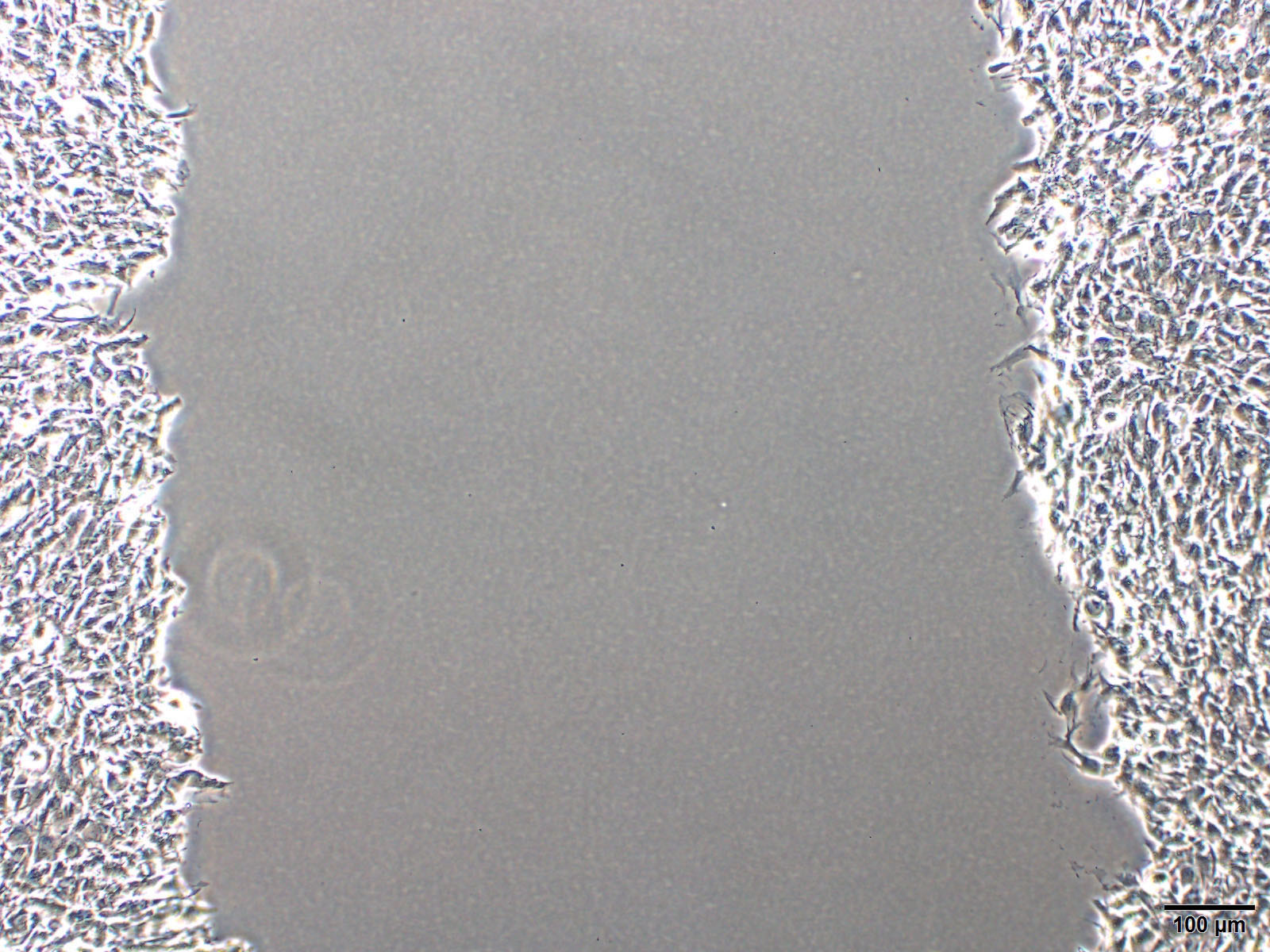

Supplement: Supplemental Information 2 [file peerj-13-19568-s002.zip › Figure 2A and 4C (Wound healing)/0h/miRNA mimics NC (2).jpg]

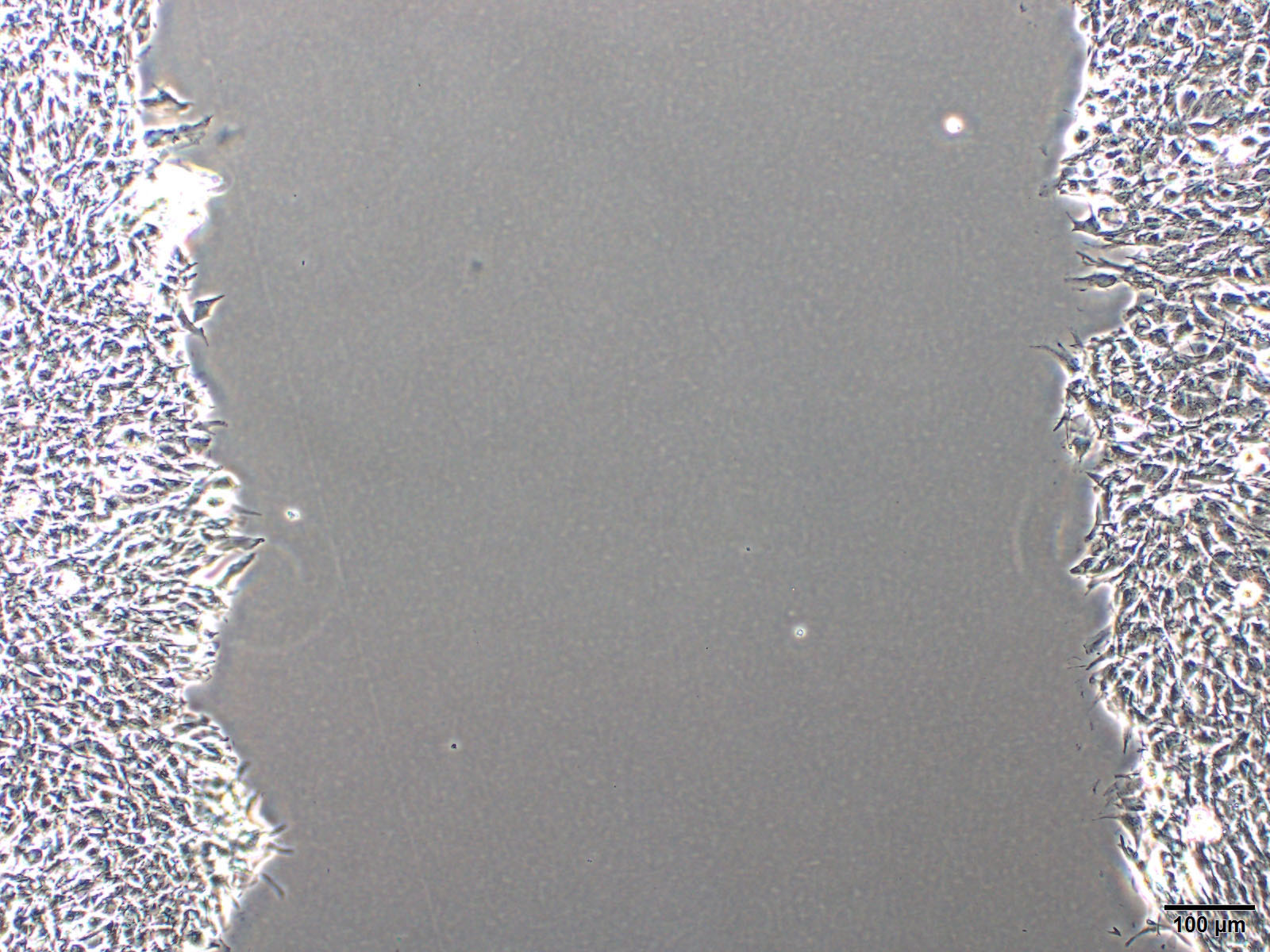

Supplement: Supplemental Information 2 [file peerj-13-19568-s002.zip › Figure 2A and 4C (Wound healing)/0h/miRNA mimics NC (3).jpg]

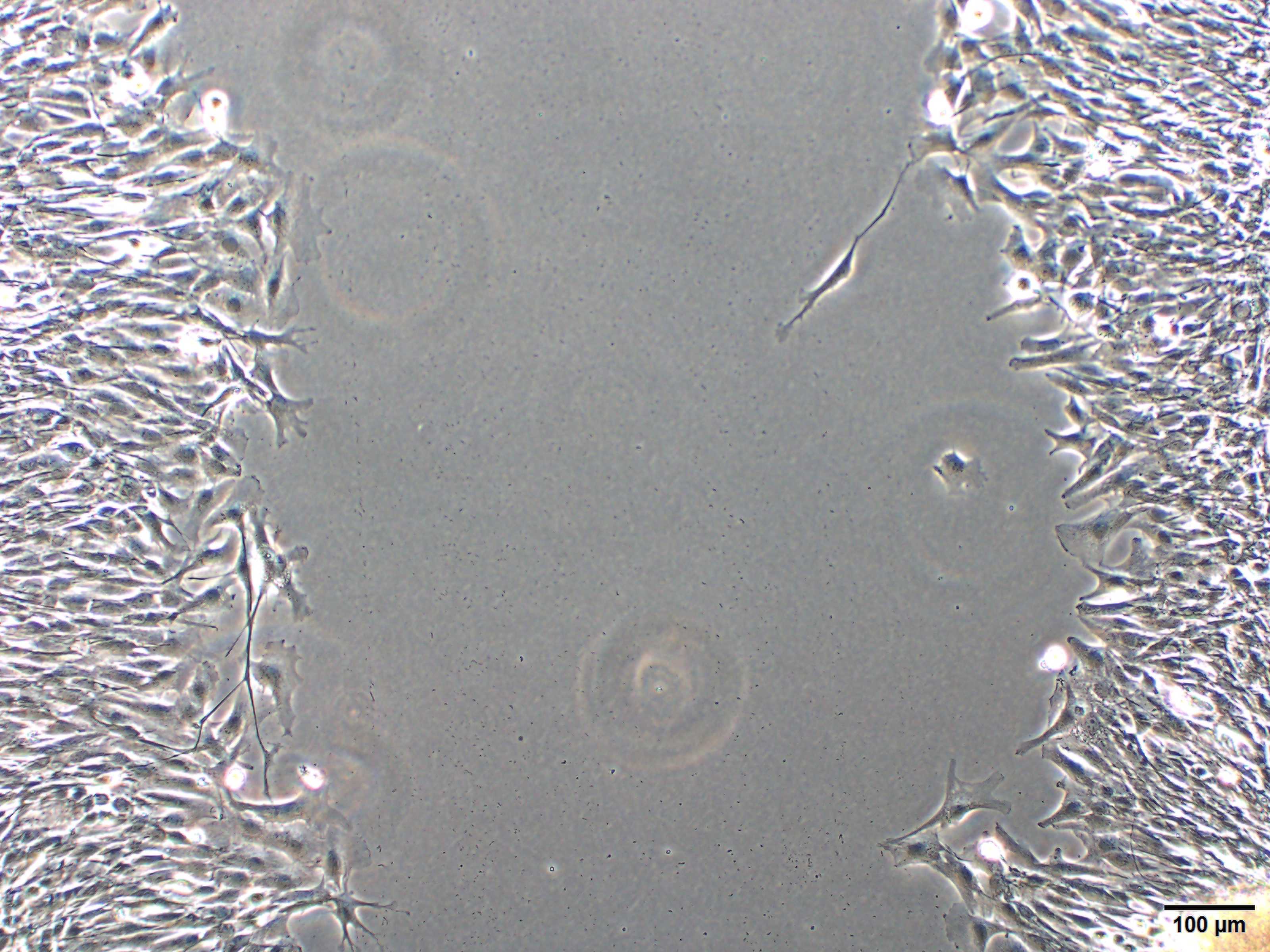

Supplement: Supplemental Information 2 [file peerj-13-19568-s002.zip › Figure 2A and 4C (Wound healing)/24h/MiRNA inhibitors+si-VEGF (1).jpg]

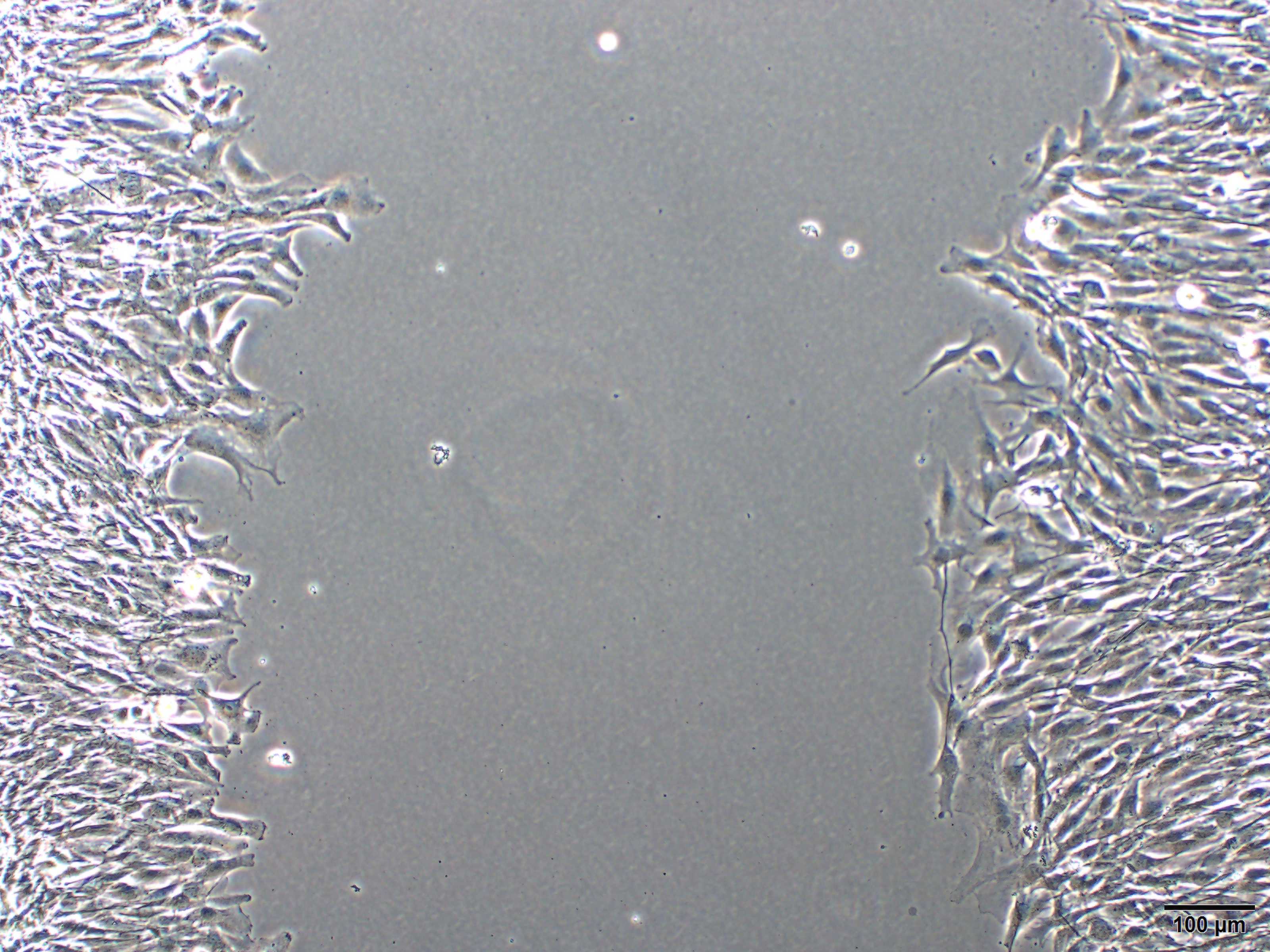

Supplement: Supplemental Information 2 [file peerj-13-19568-s002.zip › Figure 2A and 4C (Wound healing)/24h/MiRNA inhibitors+si-VEGF (2).jpg]

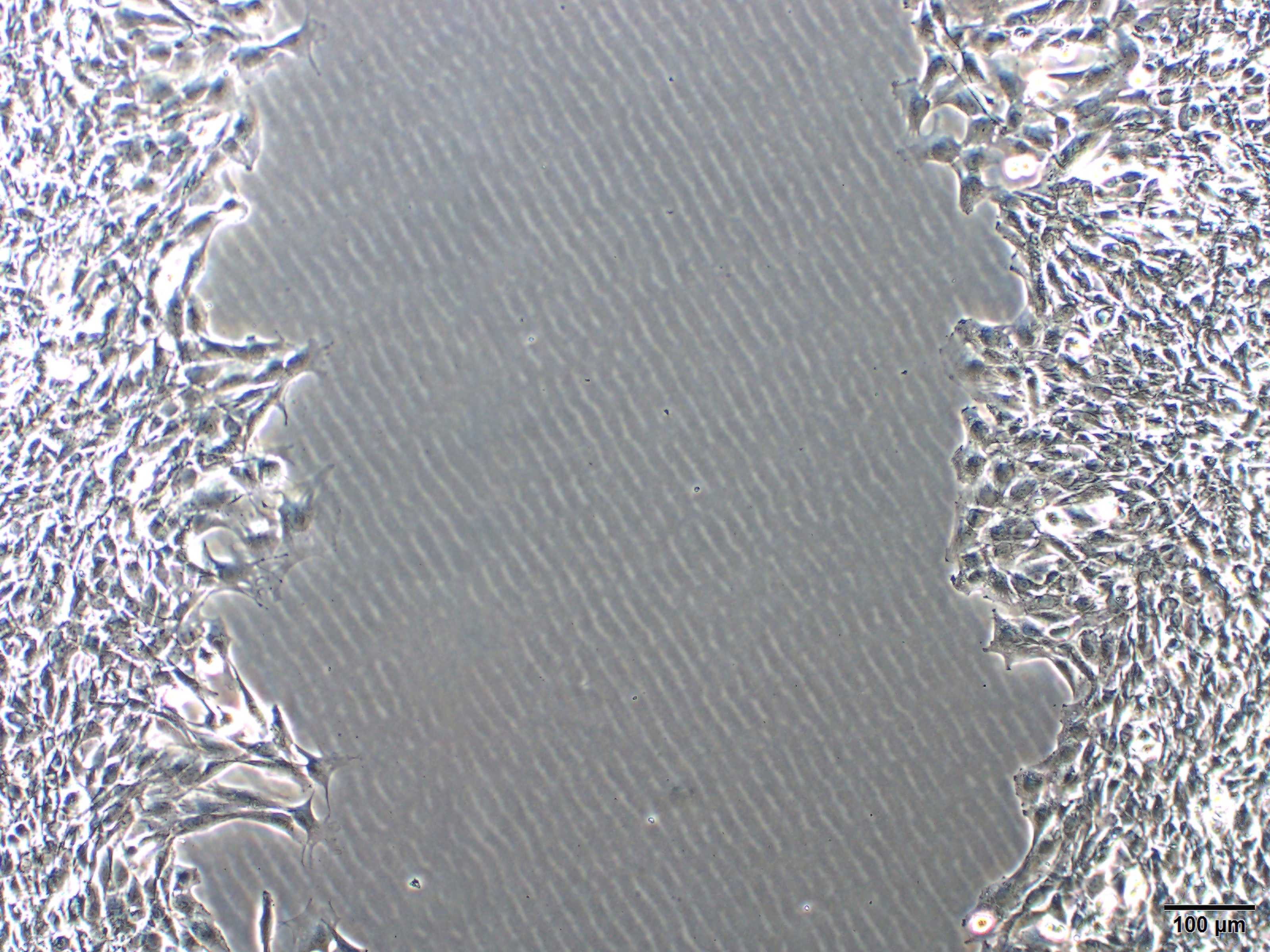

Supplement: Supplemental Information 2 [file peerj-13-19568-s002.zip › Figure 2A and 4C (Wound healing)/24h/MiRNA inhibitors+si-VEGF (3).jpg]

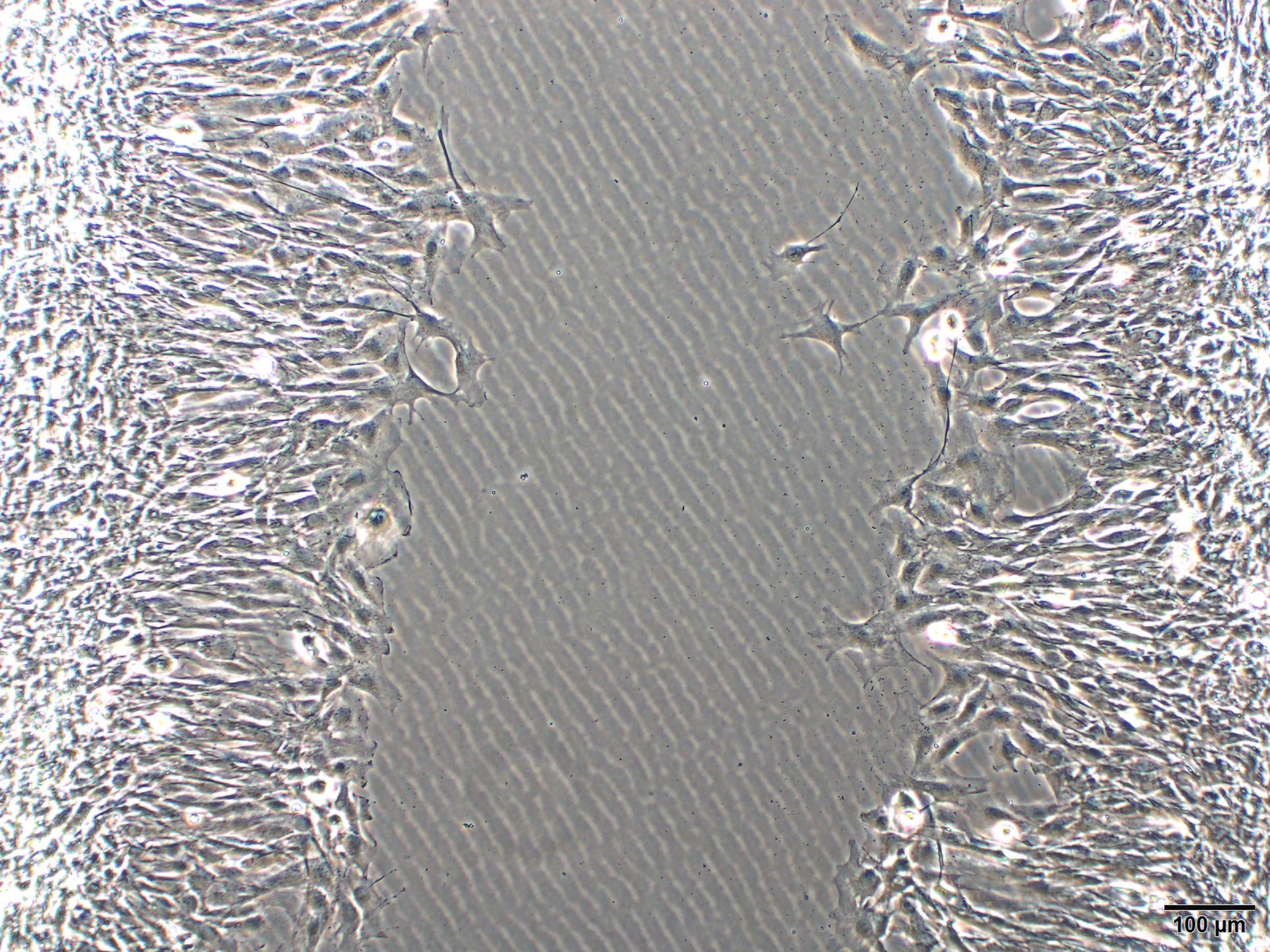

Supplement: Supplemental Information 2 [file peerj-13-19568-s002.zip › Figure 2A and 4C (Wound healing)/24h/control (1).jpg]

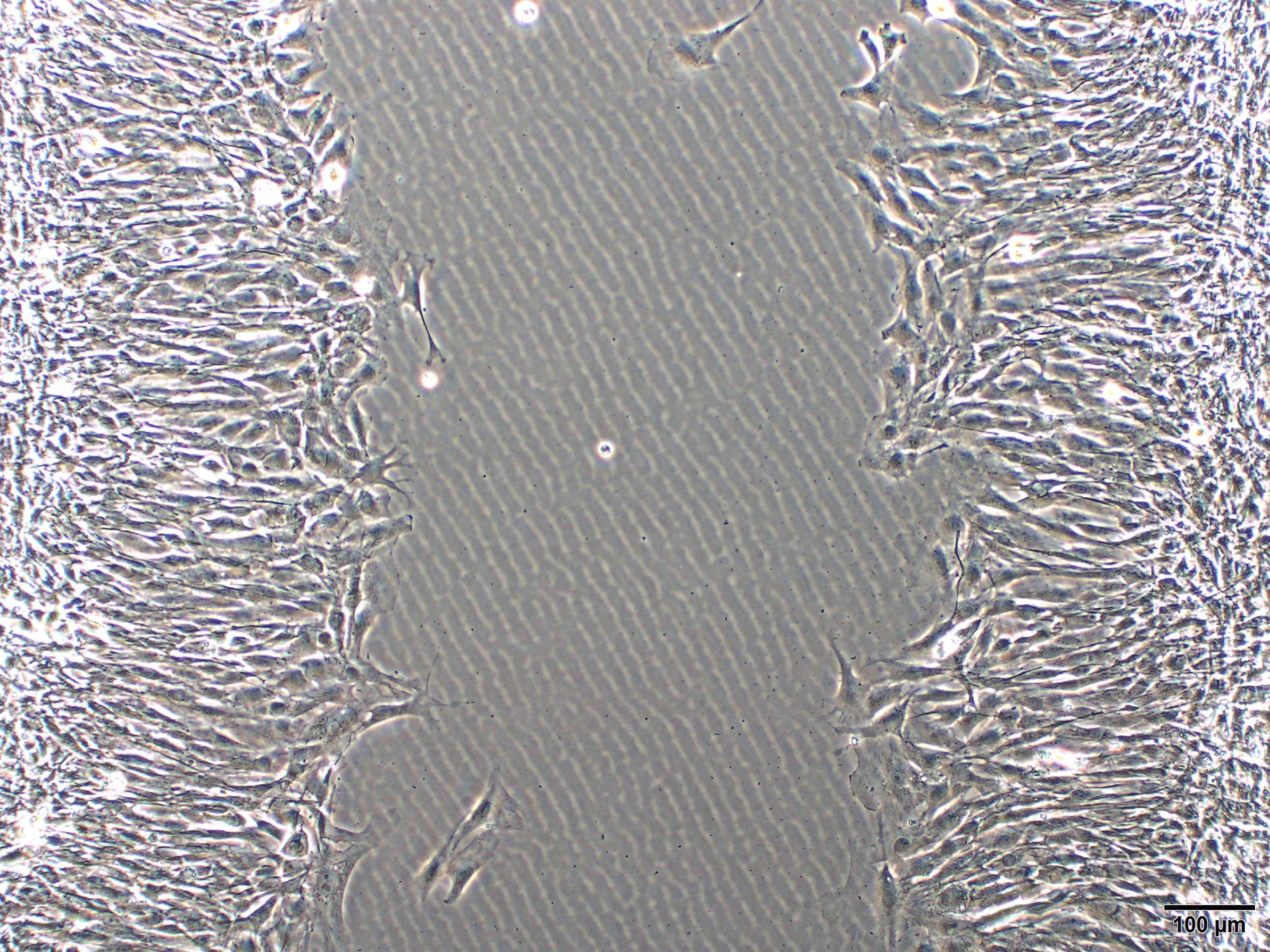

Supplement: Supplemental Information 2 [file peerj-13-19568-s002.zip › Figure 2A and 4C (Wound healing)/24h/control (2).jpg]

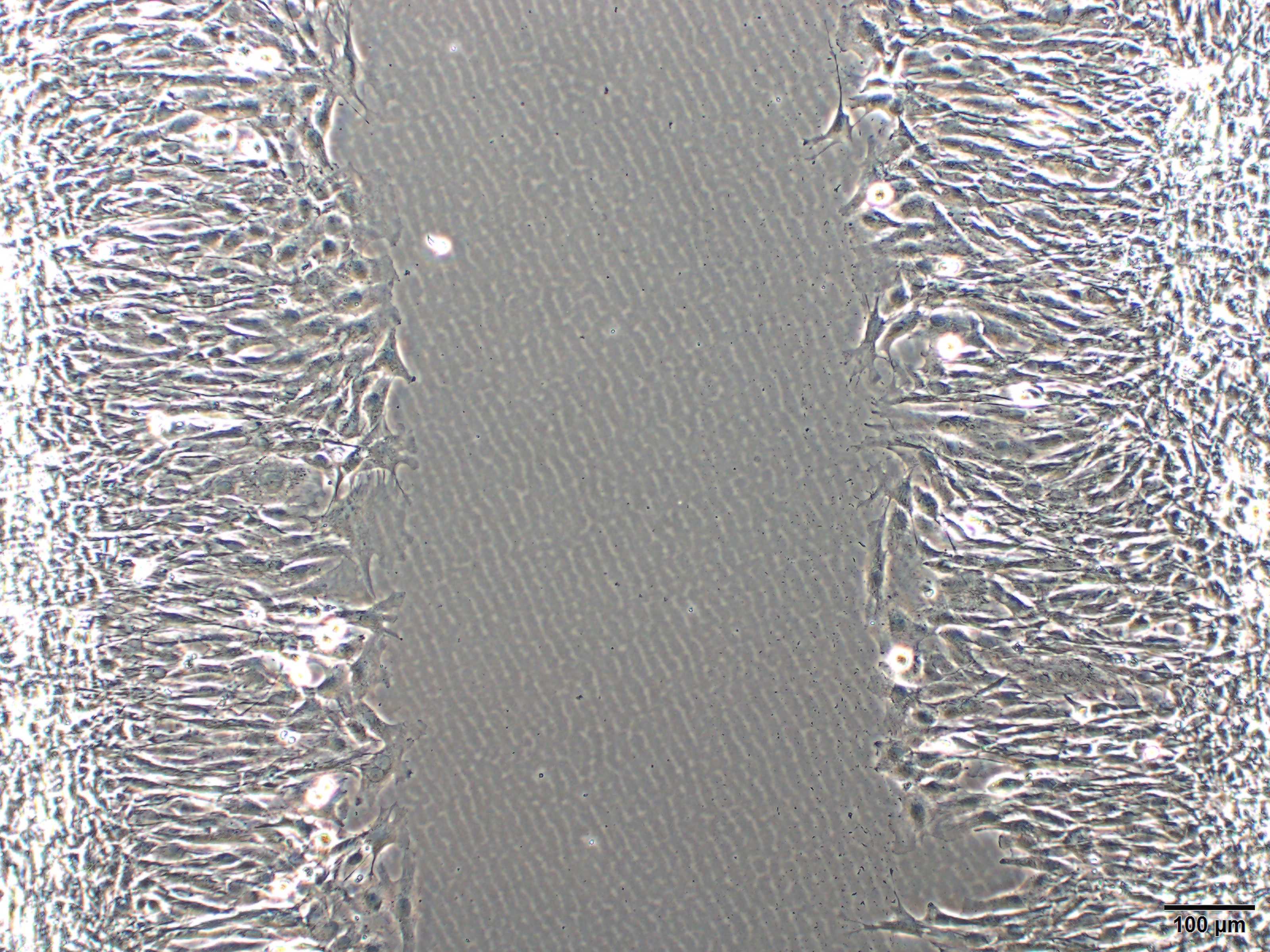

Supplement: Supplemental Information 2 [file peerj-13-19568-s002.zip › Figure 2A and 4C (Wound healing)/24h/control (3).jpg]

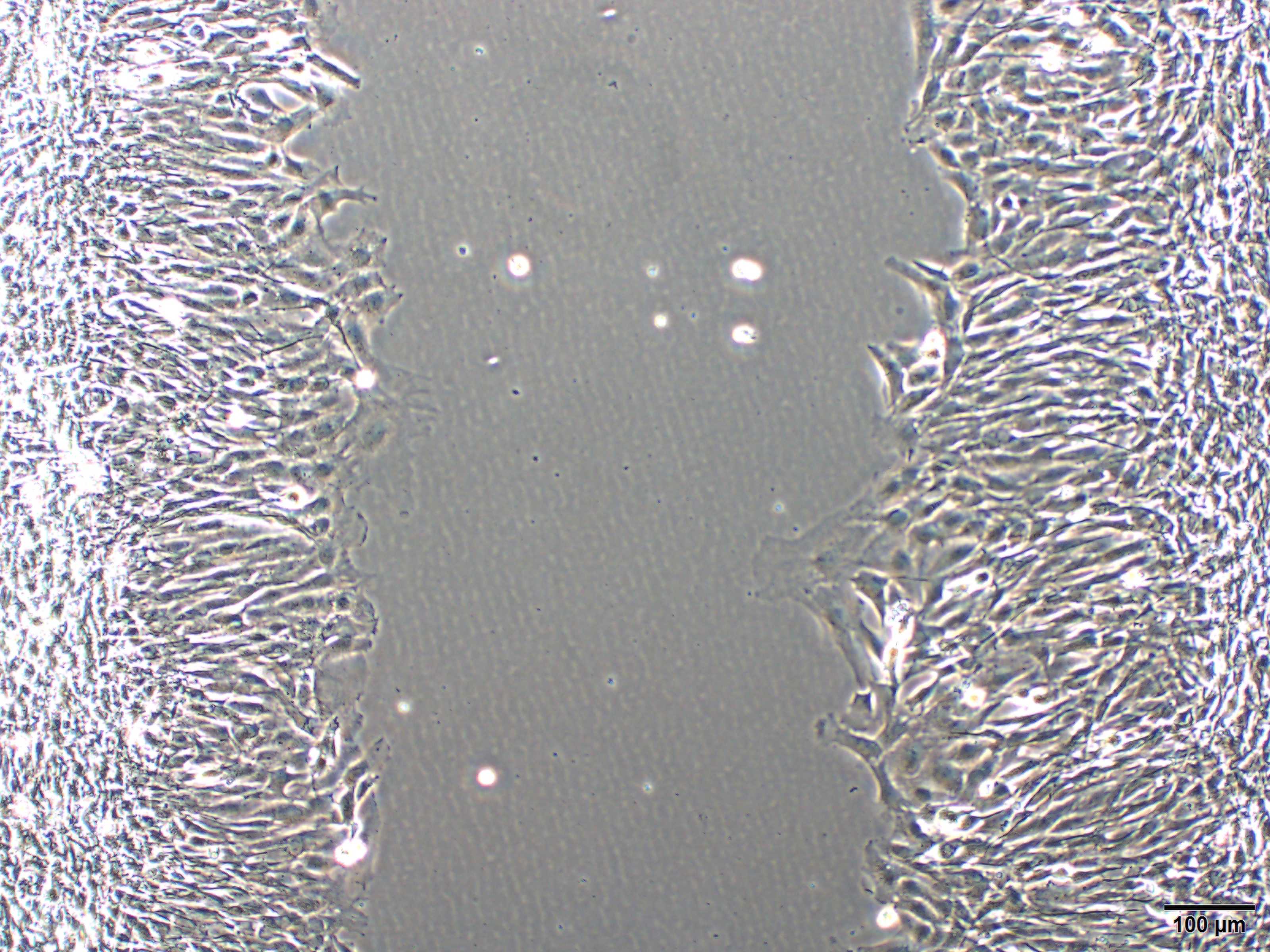

Supplement: Supplemental Information 2 [file peerj-13-19568-s002.zip › Figure 2A and 4C (Wound healing)/24h/miRNA inhibitor NC (1).jpg]

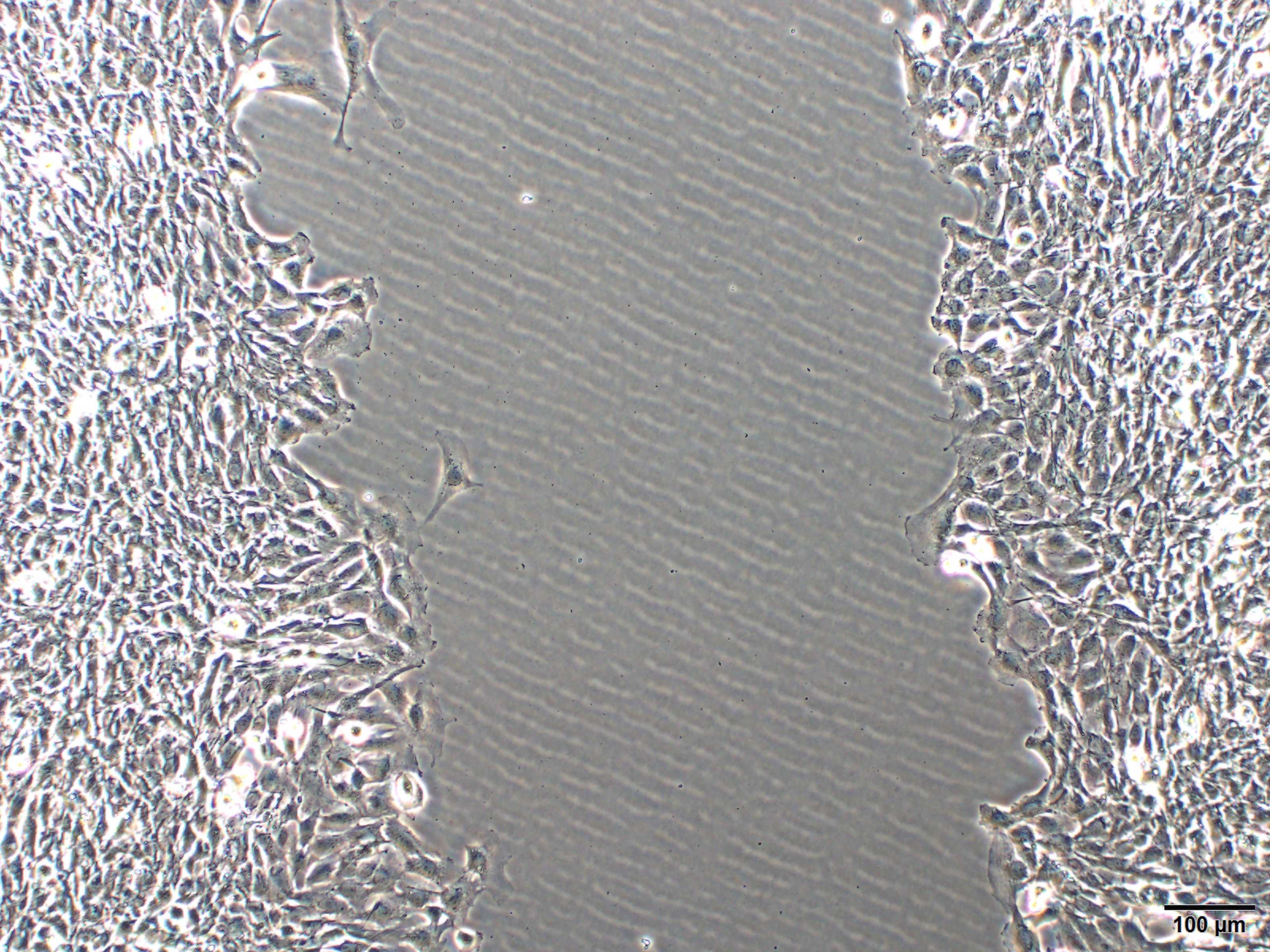

Supplement: Supplemental Information 2 [file peerj-13-19568-s002.zip › Figure 2A and 4C (Wound healing)/24h/miRNA inhibitor NC (2).jpg]

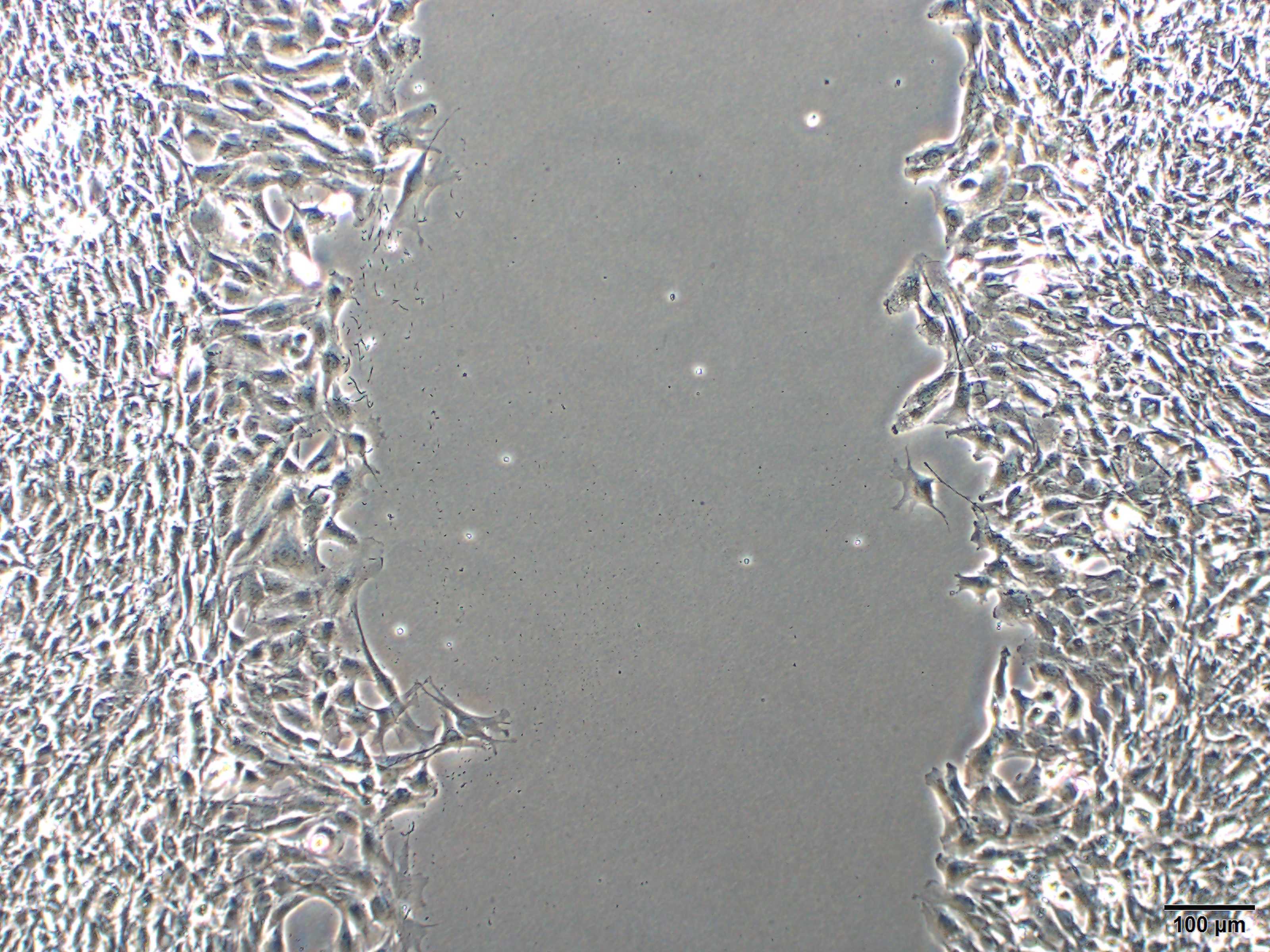

Supplement: Supplemental Information 2 [file peerj-13-19568-s002.zip › Figure 2A and 4C (Wound healing)/24h/miRNA inhibitor NC (3).jpg]

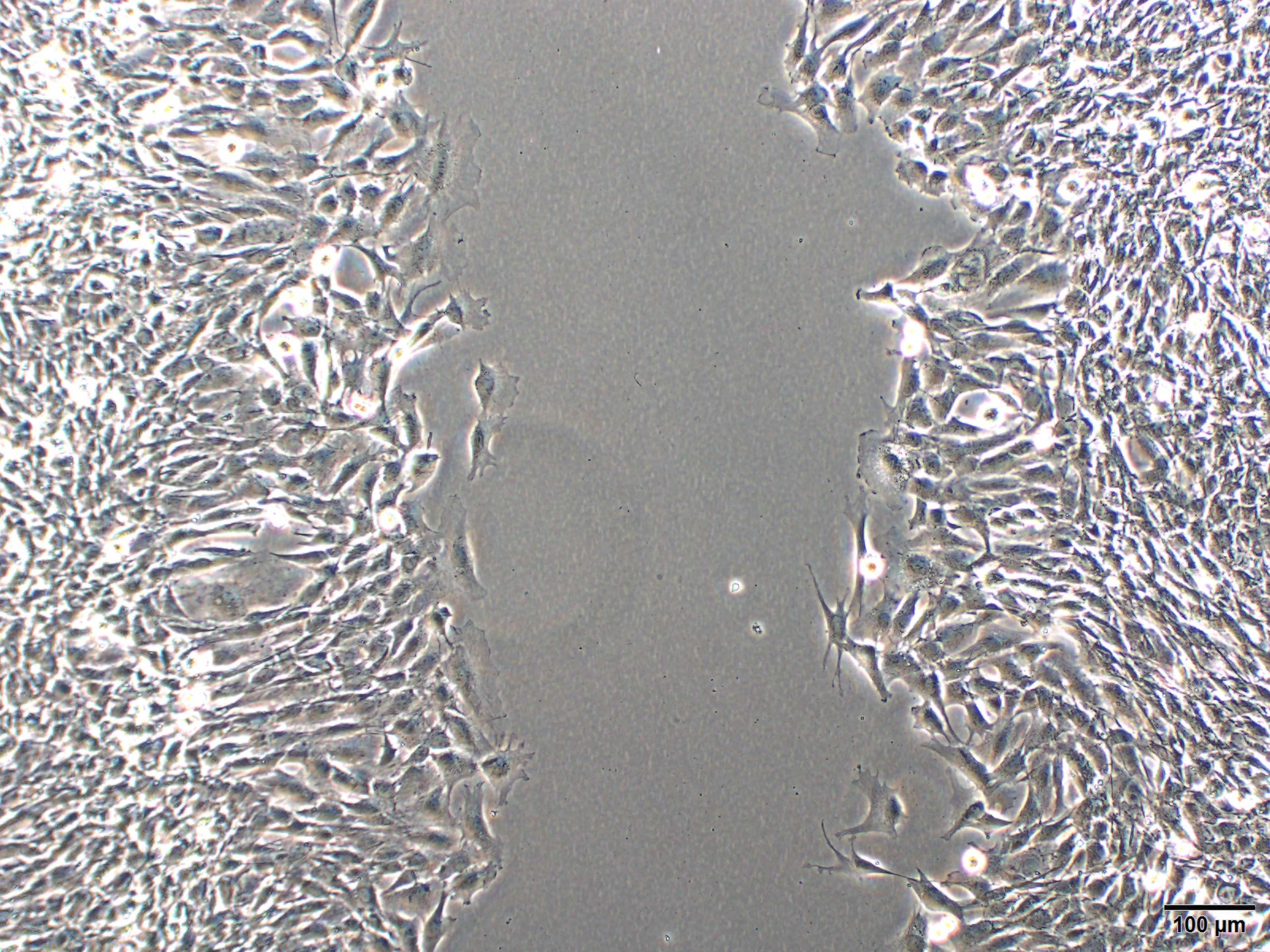

Supplement: Supplemental Information 2 [file peerj-13-19568-s002.zip › Figure 2A and 4C (Wound healing)/24h/miRNA inhibitors (1).jpg]

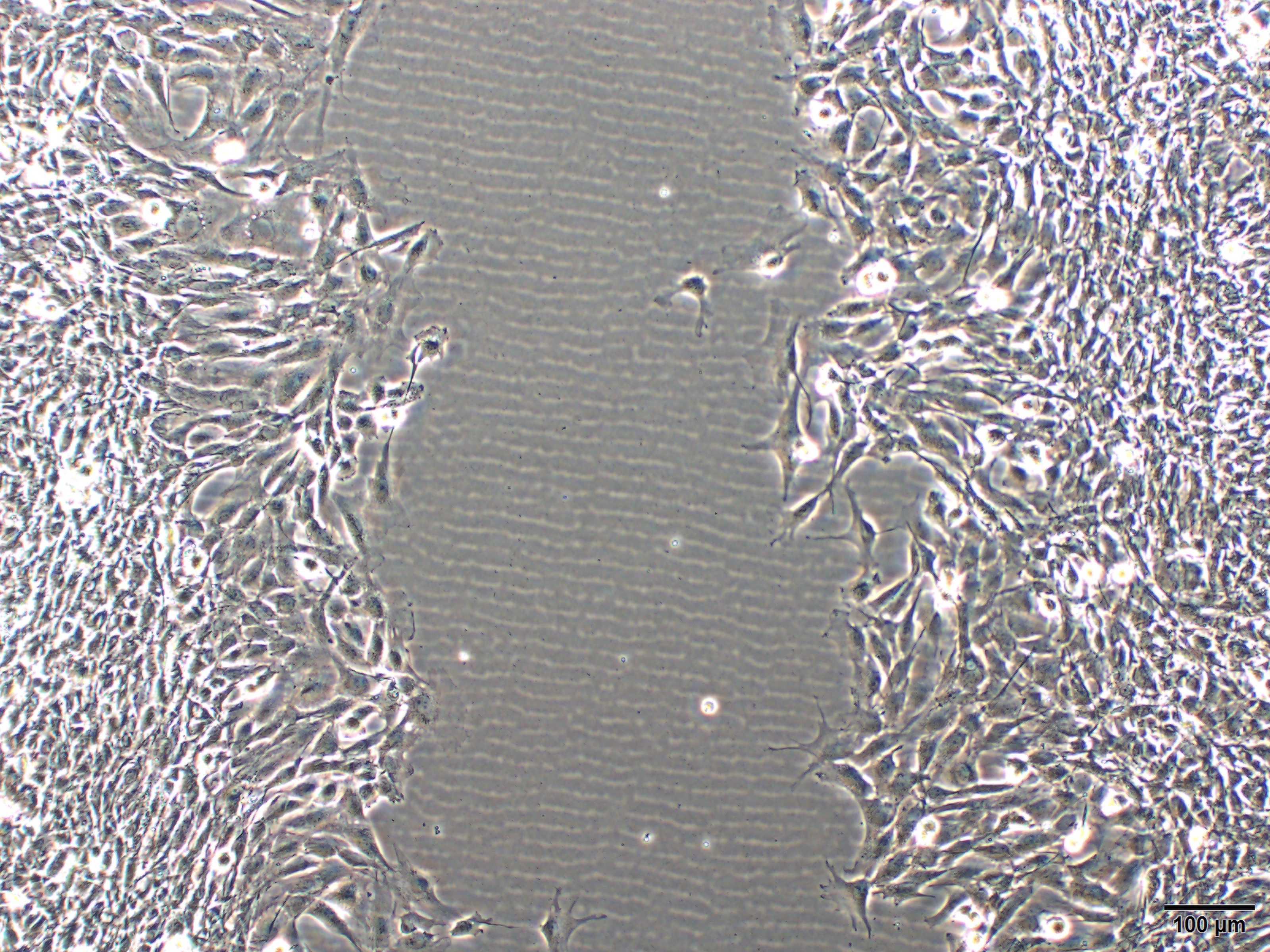

Supplement: Supplemental Information 2 [file peerj-13-19568-s002.zip › Figure 2A and 4C (Wound healing)/24h/miRNA inhibitors (2).jpg]

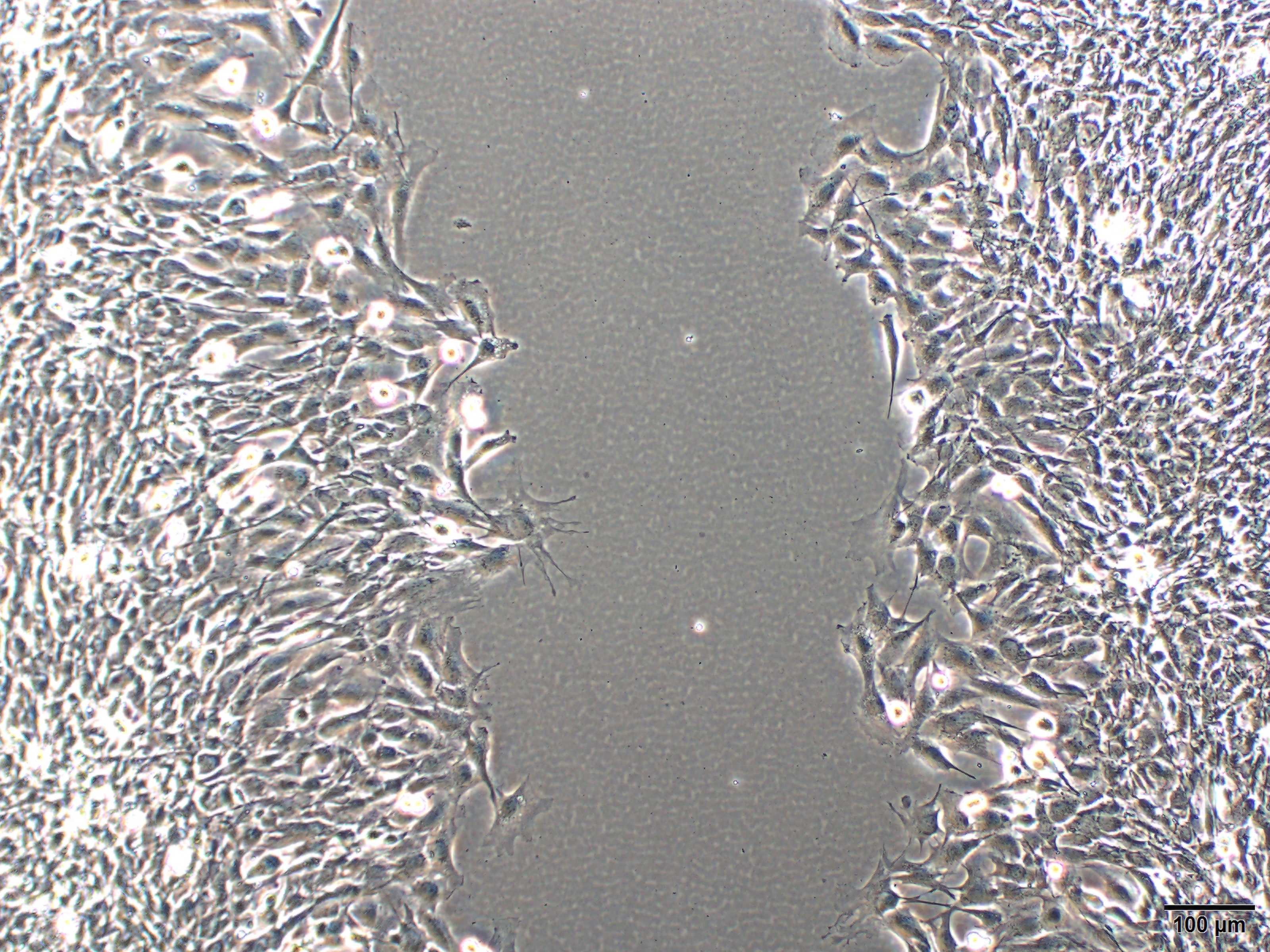

Supplement: Supplemental Information 2 [file peerj-13-19568-s002.zip › Figure 2A and 4C (Wound healing)/24h/miRNA inhibitors (3).jpg]

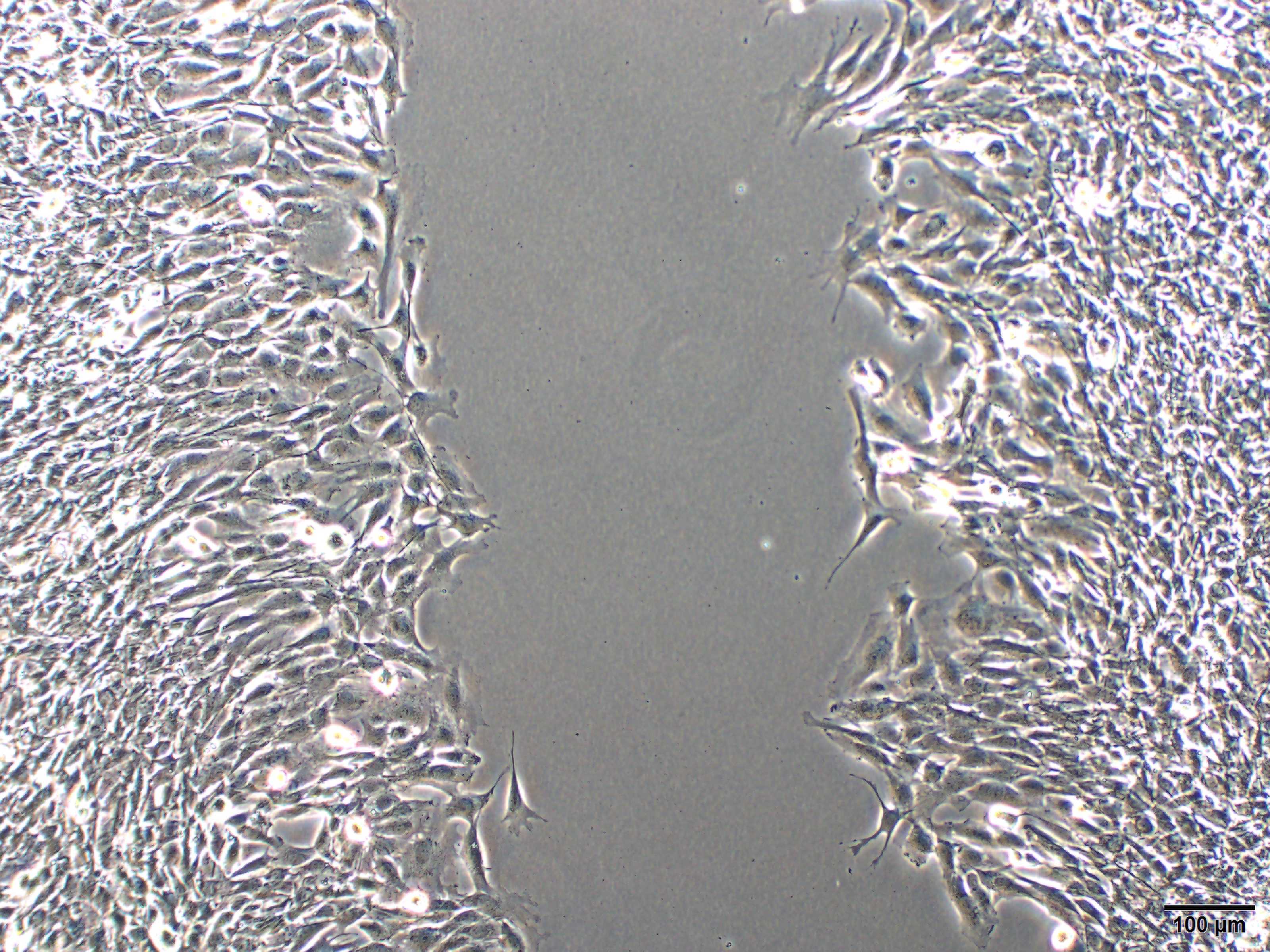

Supplement: Supplemental Information 2 [file peerj-13-19568-s002.zip › Figure 2A and 4C (Wound healing)/24h/miRNA inhibitors +si-NC (1).jpg]

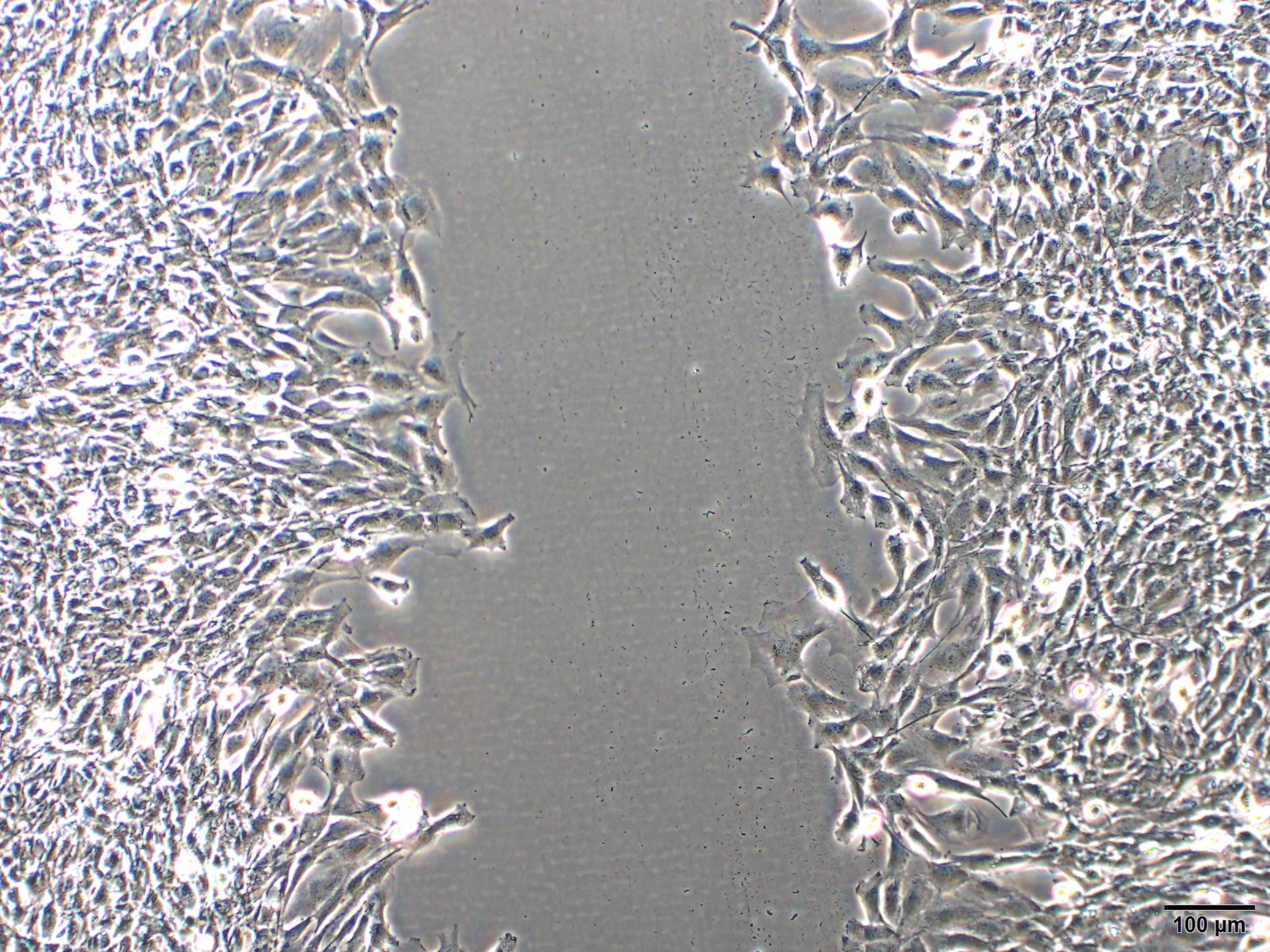

Supplement: Supplemental Information 2 [file peerj-13-19568-s002.zip › Figure 2A and 4C (Wound healing)/24h/miRNA inhibitors +si-NC (2).jpg]

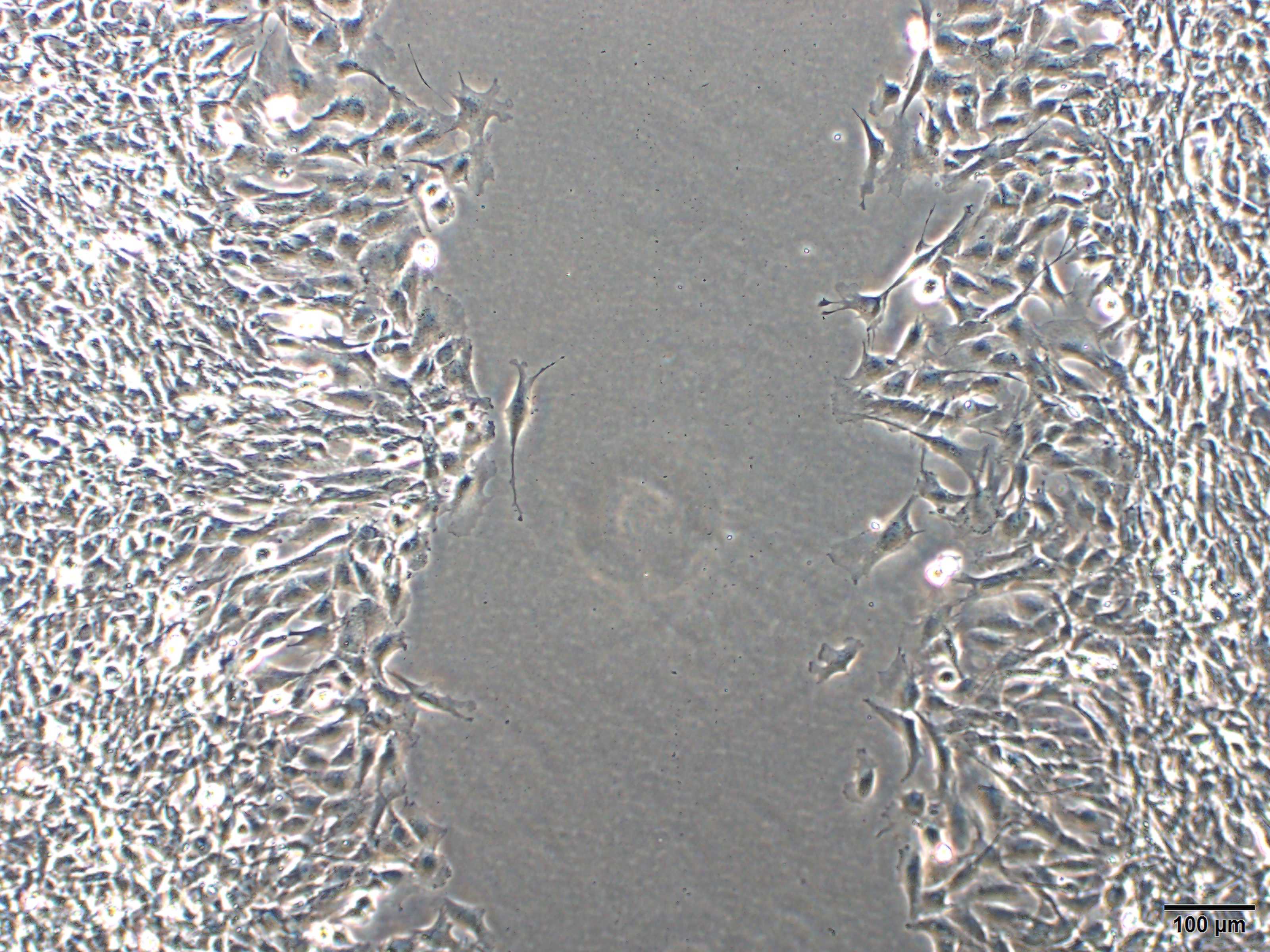

Supplement: Supplemental Information 2 [file peerj-13-19568-s002.zip › Figure 2A and 4C (Wound healing)/24h/miRNA inhibitors +si-NC (3).jpg]

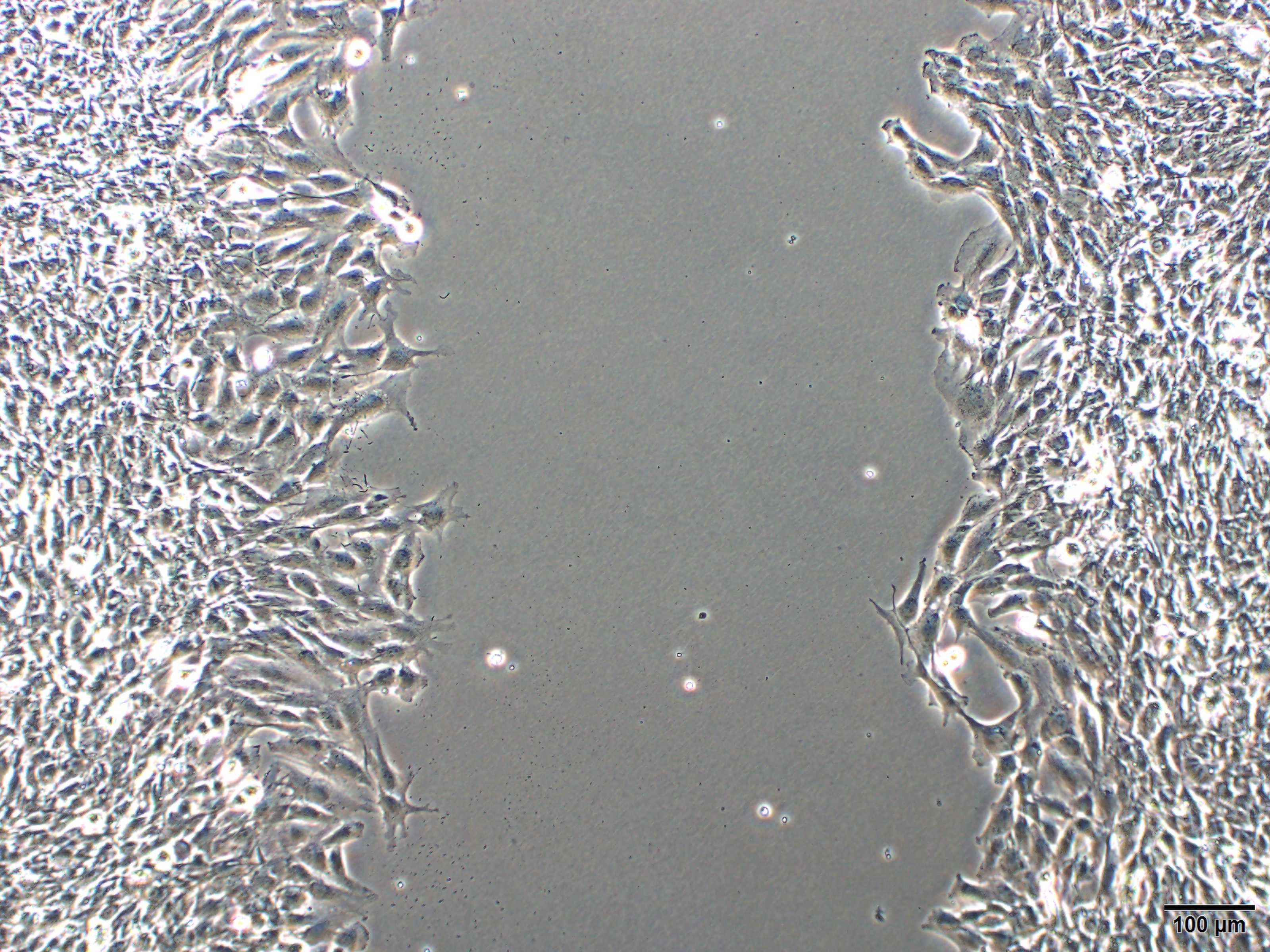

Supplement: Supplemental Information 2 [file peerj-13-19568-s002.zip › Figure 2A and 4C (Wound healing)/24h/miRNA mimics NC (1).jpg]

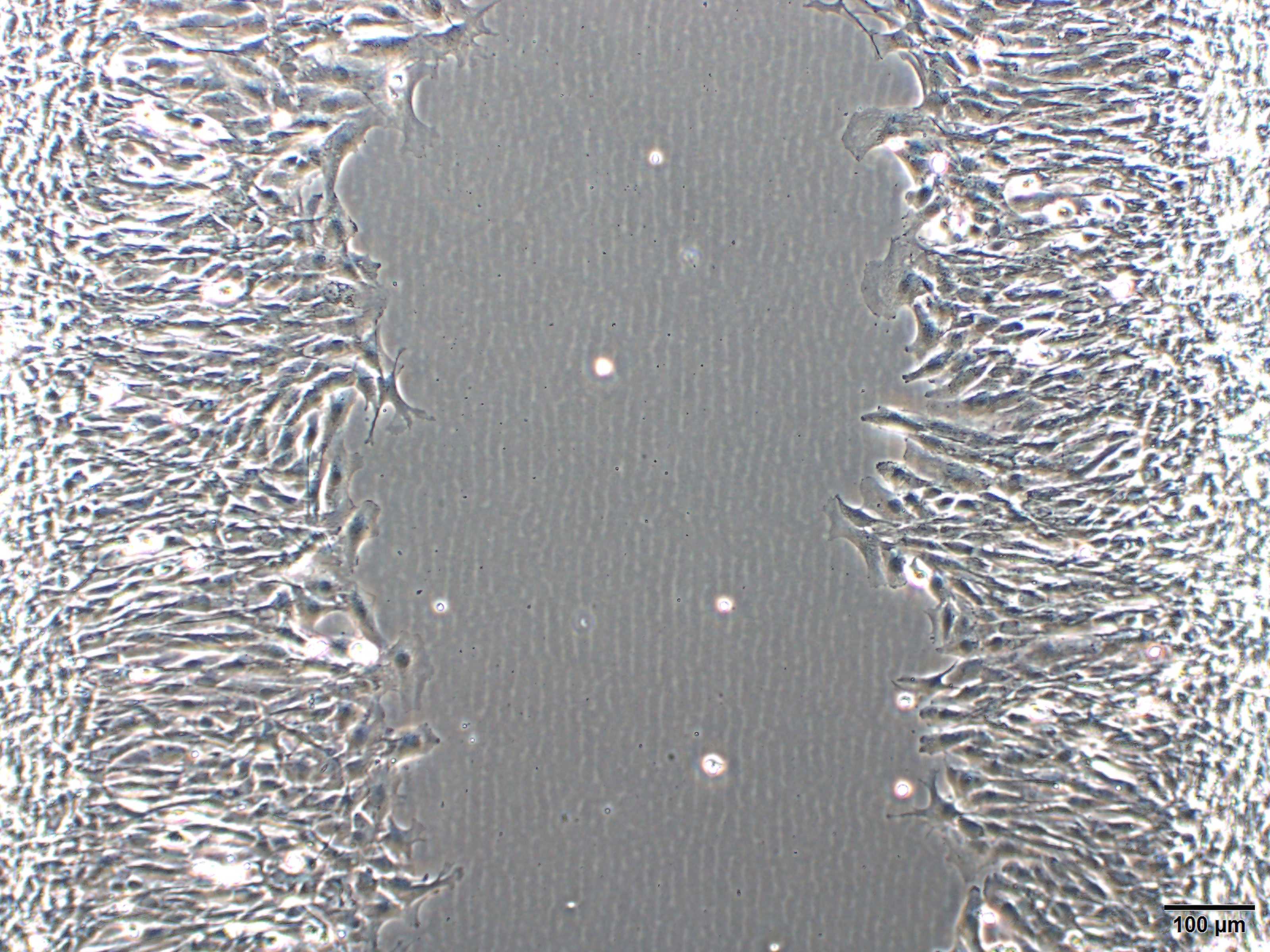

Supplement: Supplemental Information 2 [file peerj-13-19568-s002.zip › Figure 2A and 4C (Wound healing)/24h/miRNA mimics NC (2).jpg]

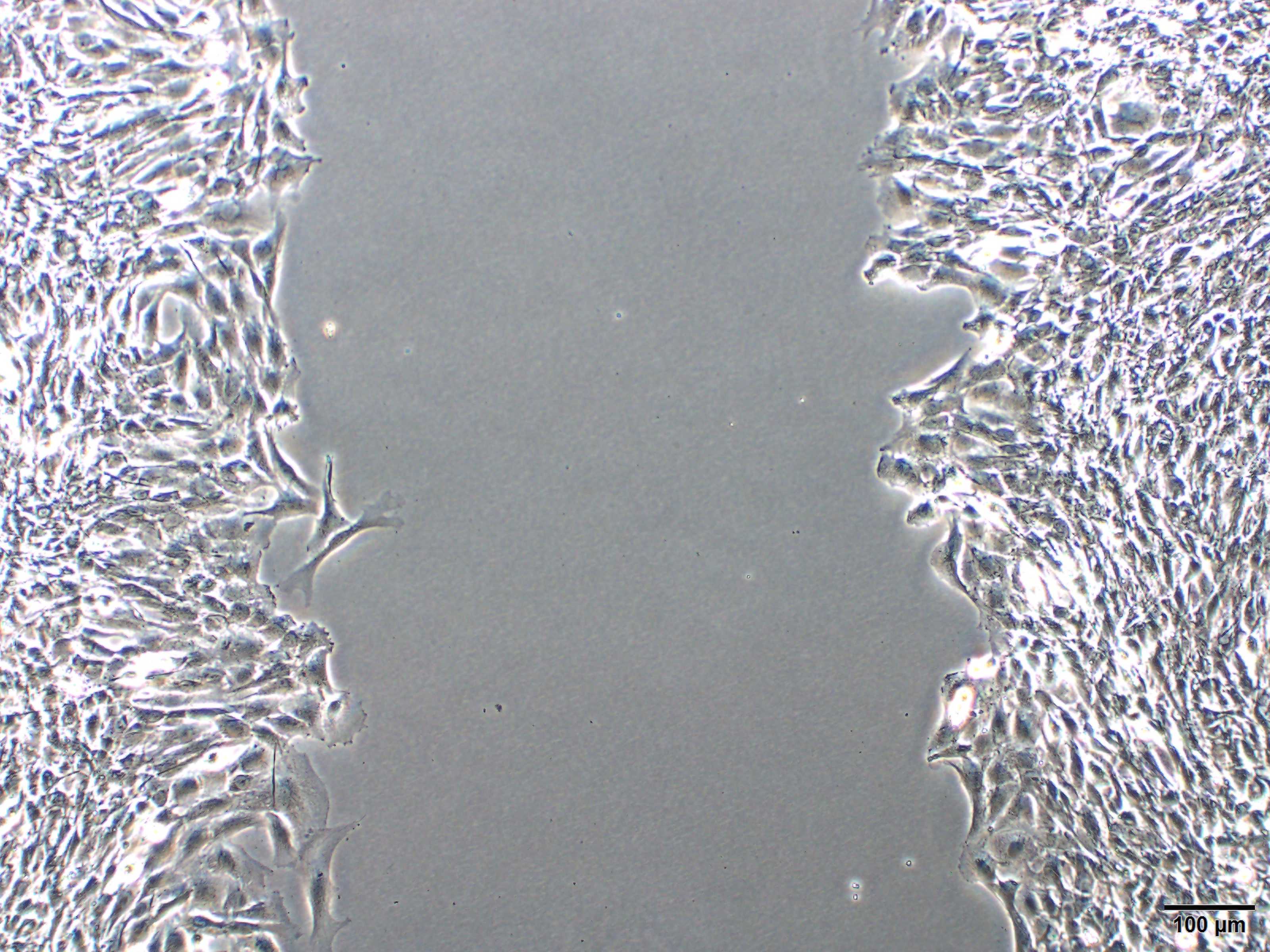

Supplement: Supplemental Information 2 [file peerj-13-19568-s002.zip › Figure 2A and 4C (Wound healing)/24h/miRNA mimics NC (3).jpg]

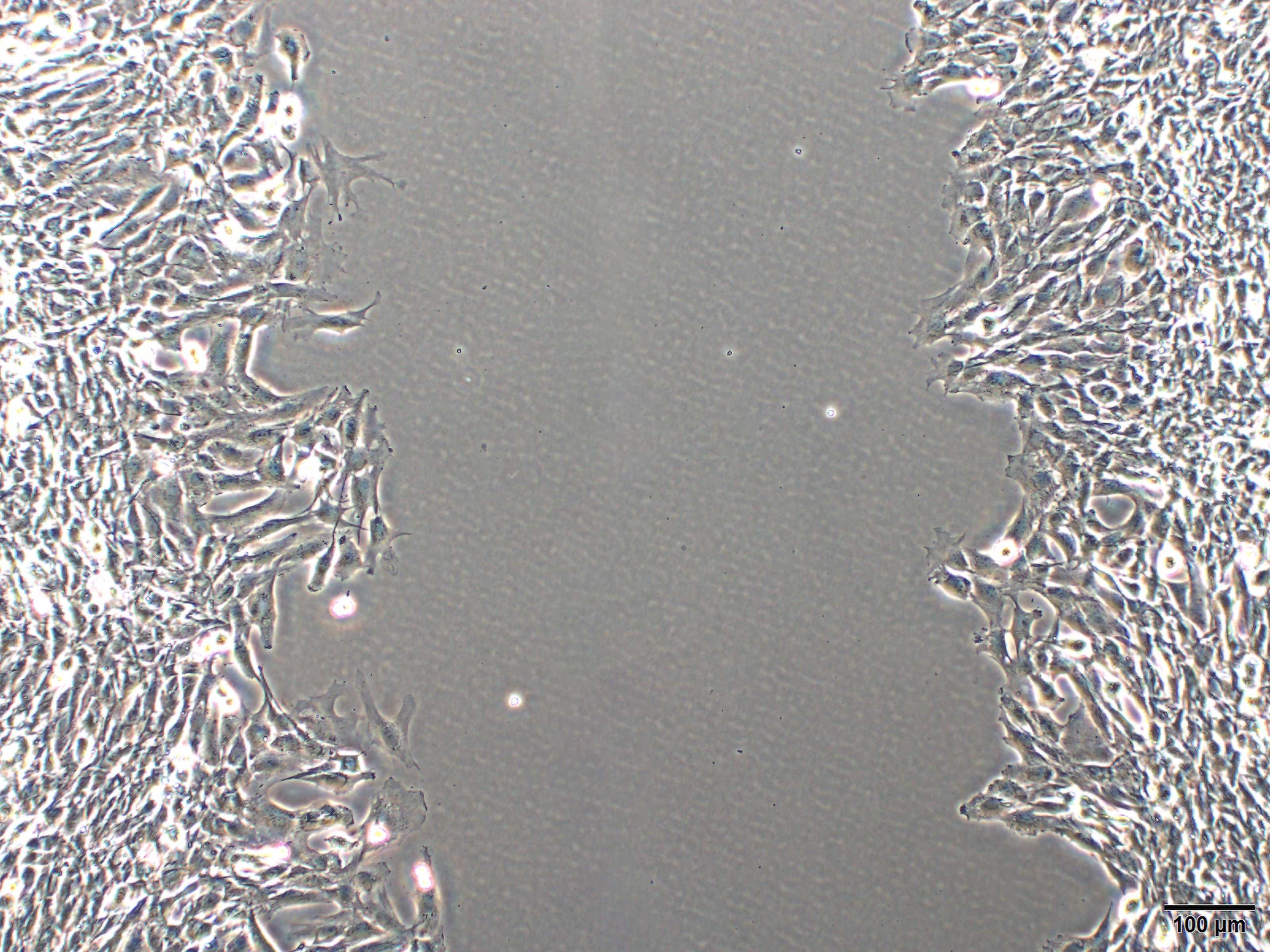

Supplement: Supplemental Information 2 [file peerj-13-19568-s002.zip › Figure 2A and 4C (Wound healing)/24h/miRNA mimics1 (1).jpg]

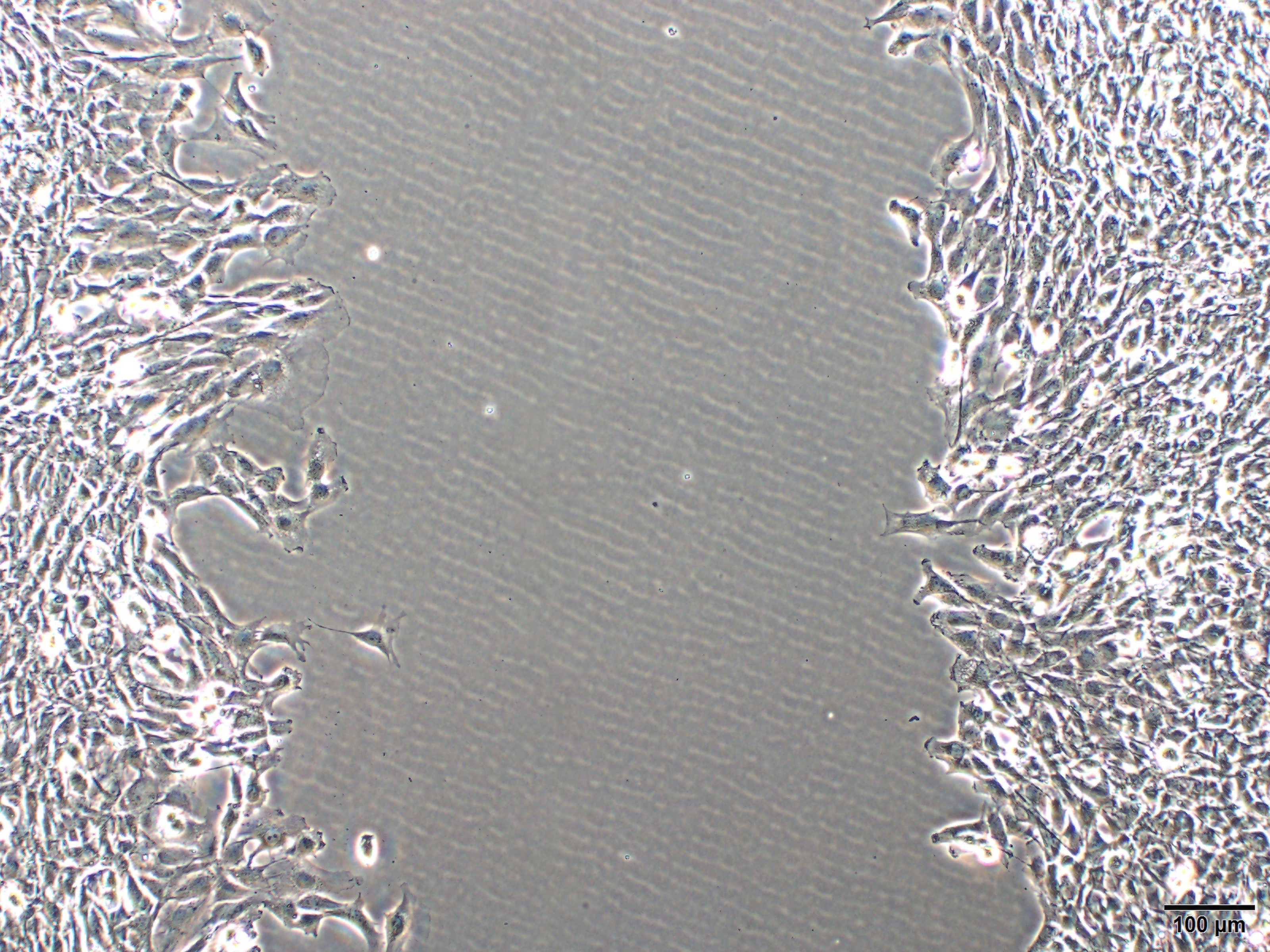

Supplement: Supplemental Information 2 [file peerj-13-19568-s002.zip › Figure 2A and 4C (Wound healing)/24h/miRNA mimics1 (2).jpg]

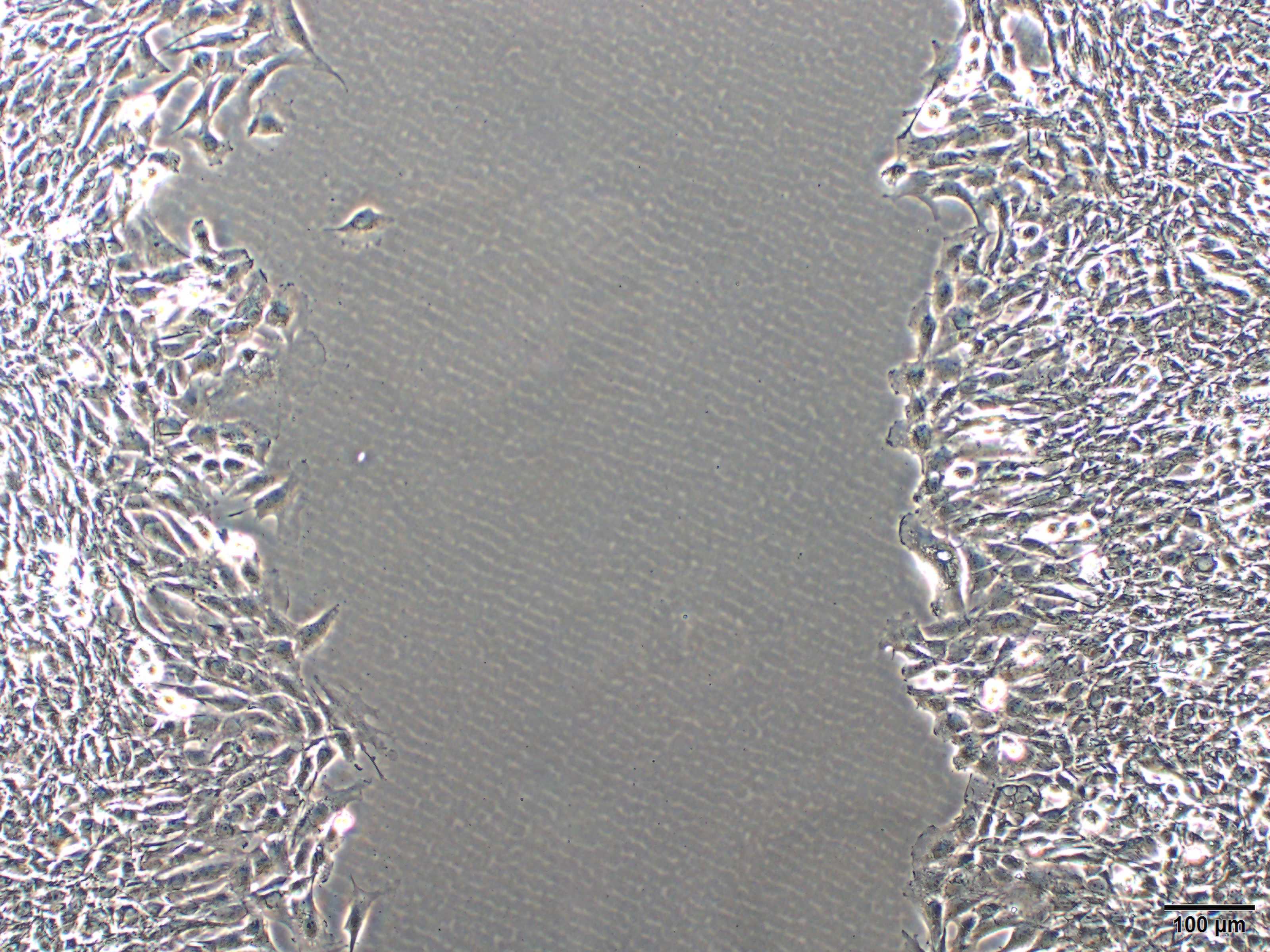

Supplement: Supplemental Information 2 [file peerj-13-19568-s002.zip › Figure 2A and 4C (Wound healing)/24h/miRNA mimics1 (3).jpg]

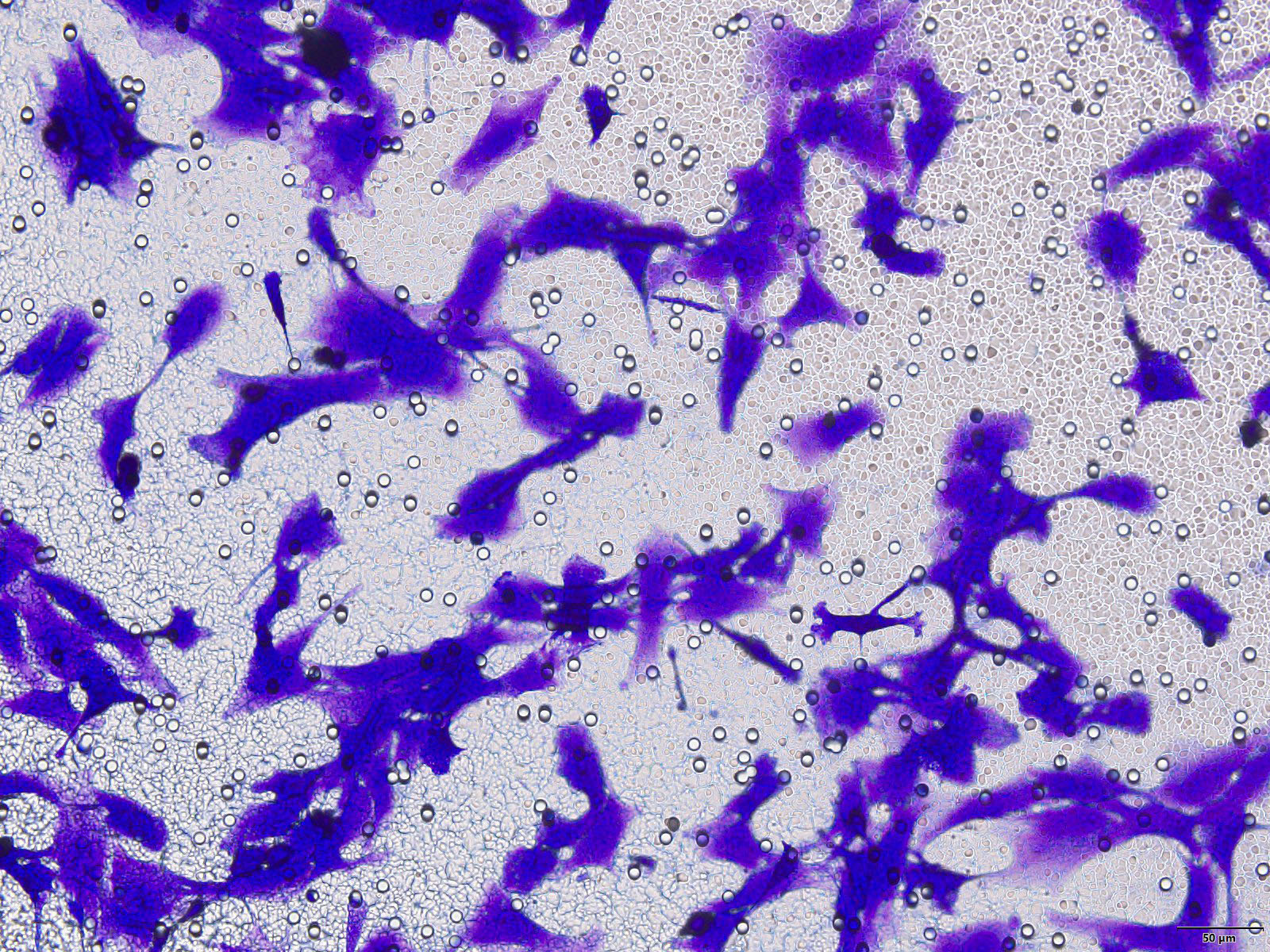

Supplement: Supplemental Information 3 [file peerj-13-19568-s003.zip › Figure 2A and 4C (Transwell)/control (1).jpg]

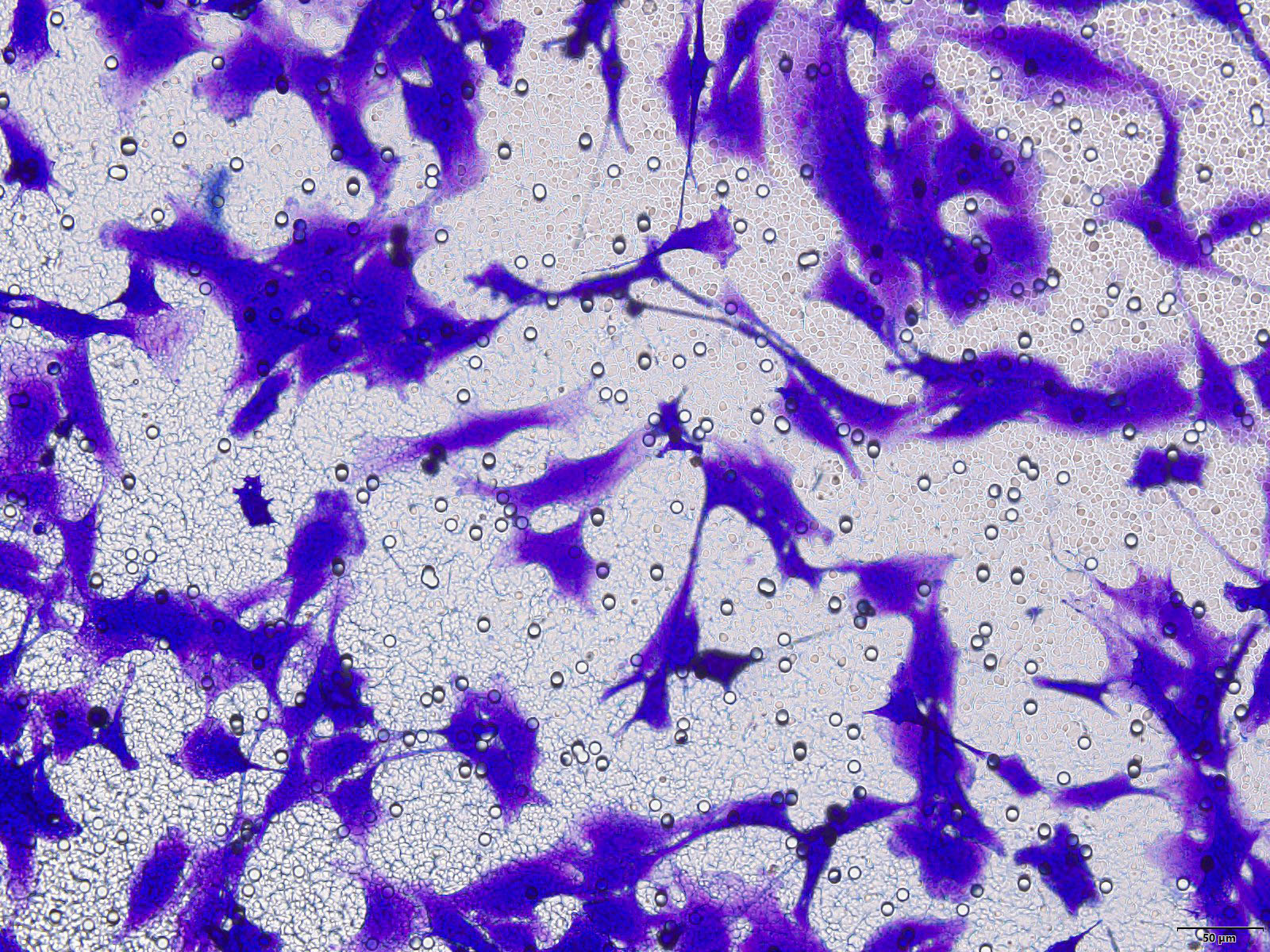

Supplement: Supplemental Information 3 [file peerj-13-19568-s003.zip › Figure 2A and 4C (Transwell)/control (2).jpg]

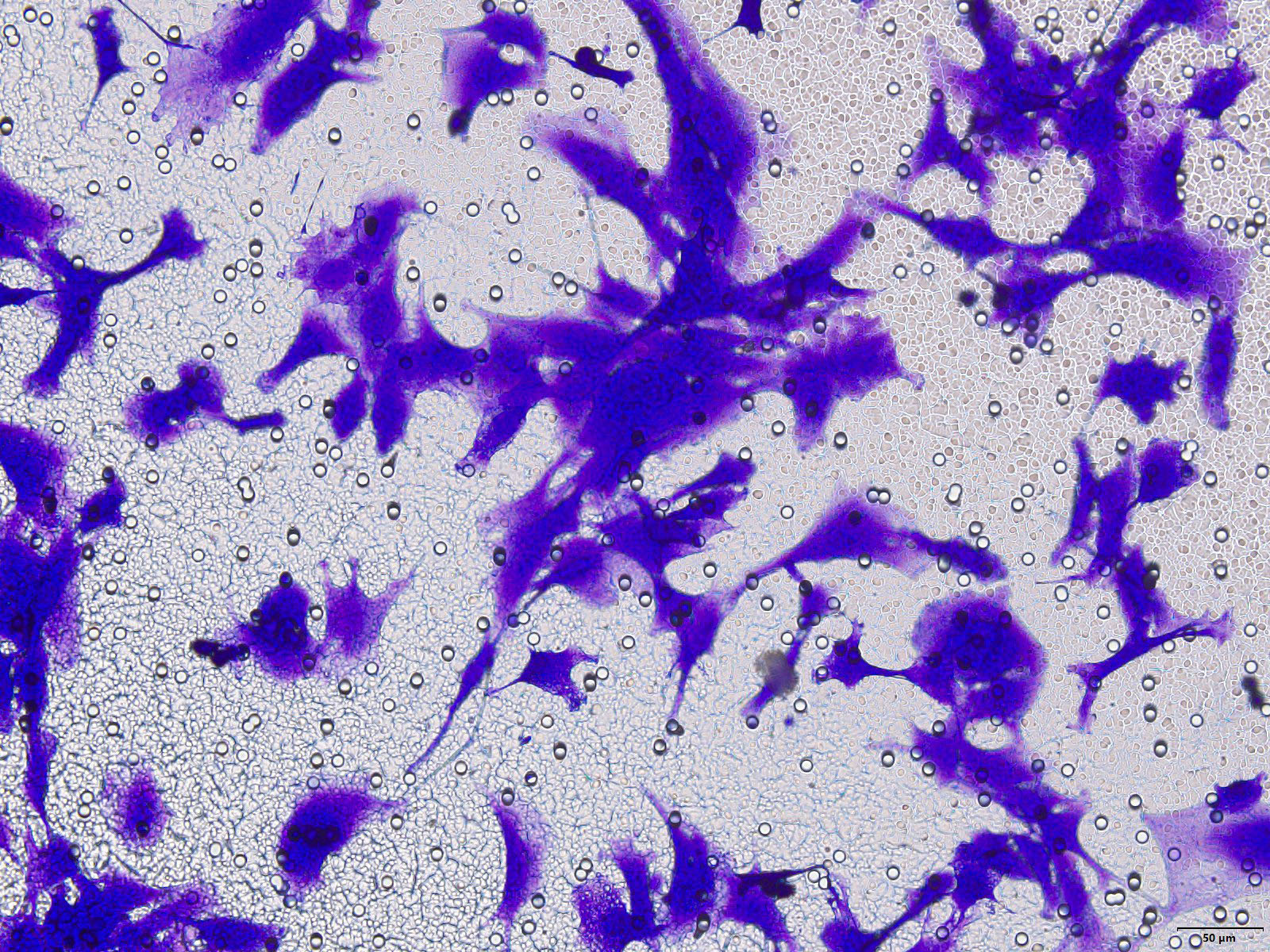

Supplement: Supplemental Information 3 [file peerj-13-19568-s003.zip › Figure 2A and 4C (Transwell)/control (3).jpg]

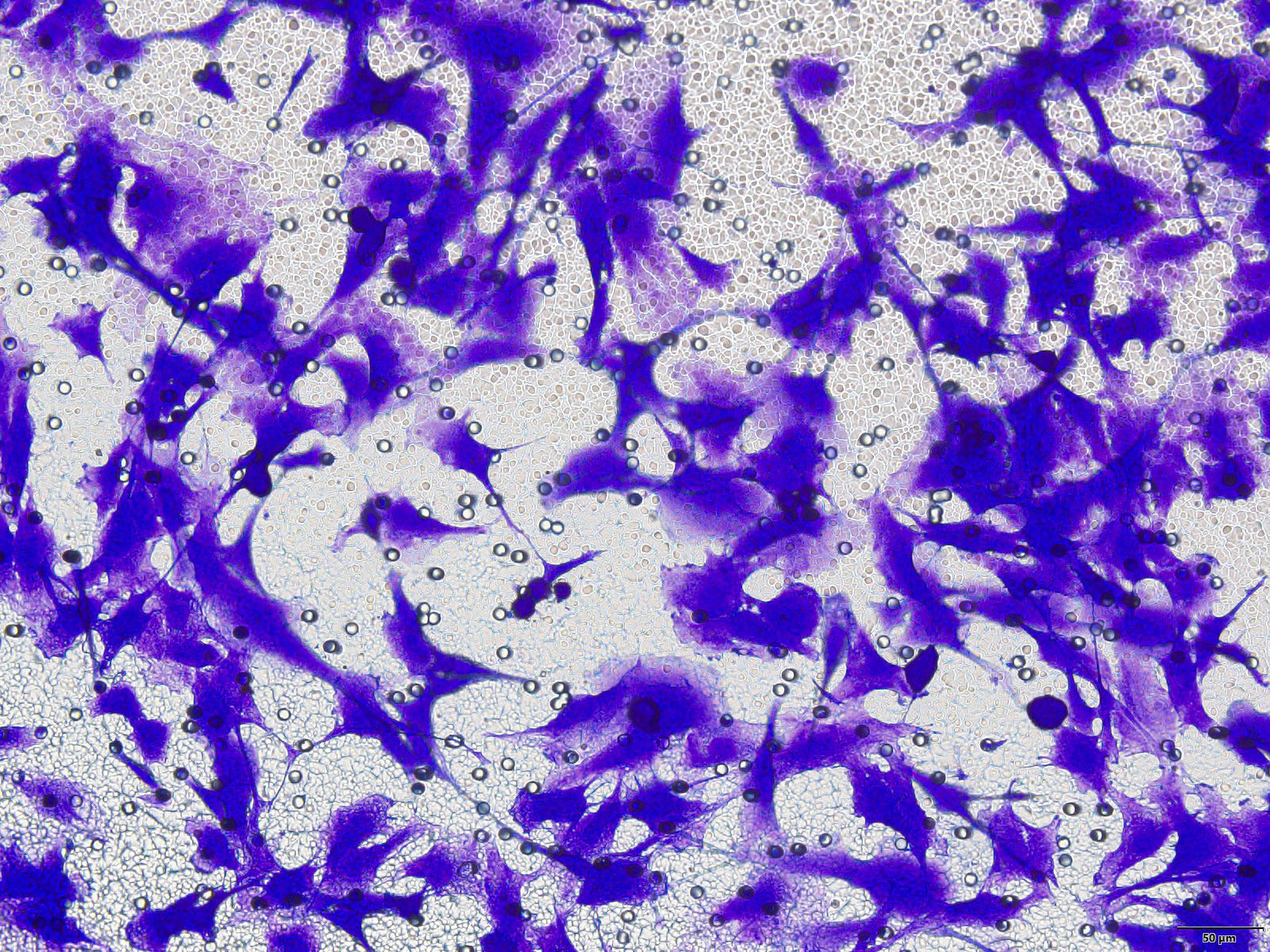

Supplement: Supplemental Information 3 [file peerj-13-19568-s003.zip › Figure 2A and 4C (Transwell)/miRNA inhibitor (1).jpg]

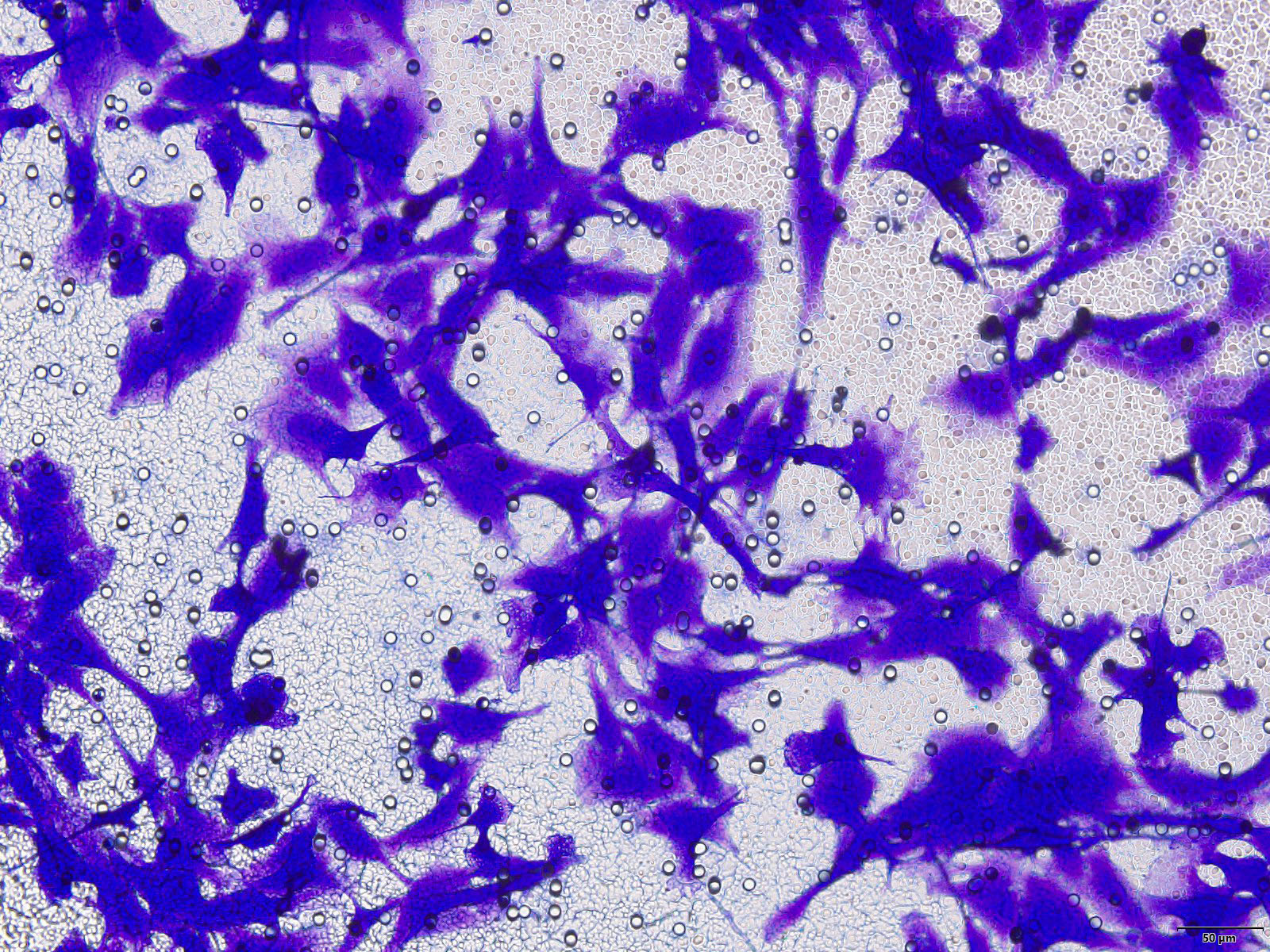

Supplement: Supplemental Information 3 [file peerj-13-19568-s003.zip › Figure 2A and 4C (Transwell)/miRNA inhibitor (2).jpg]

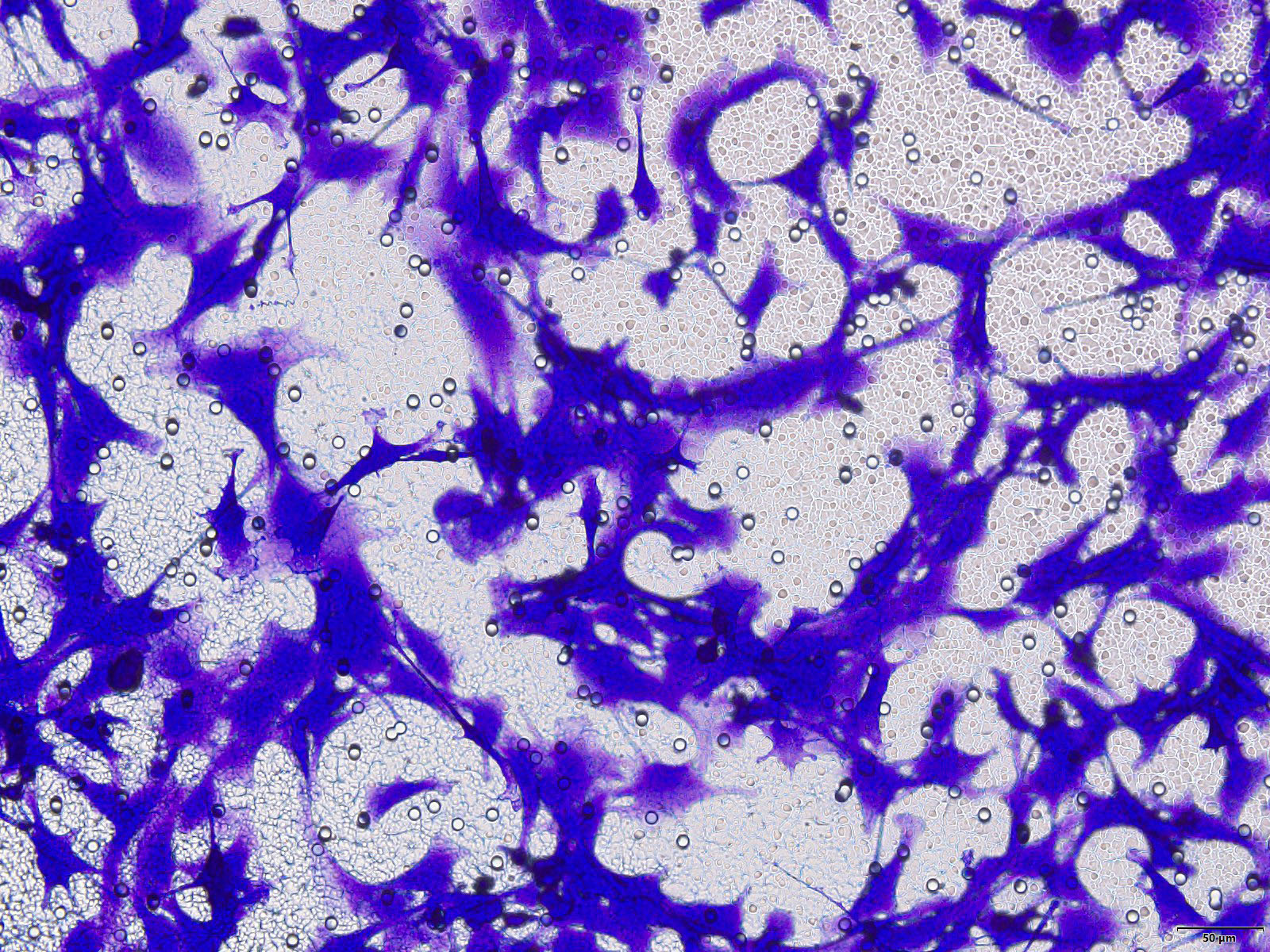

Supplement: Supplemental Information 3 [file peerj-13-19568-s003.zip › Figure 2A and 4C (Transwell)/miRNA inhibitor (3).jpg]

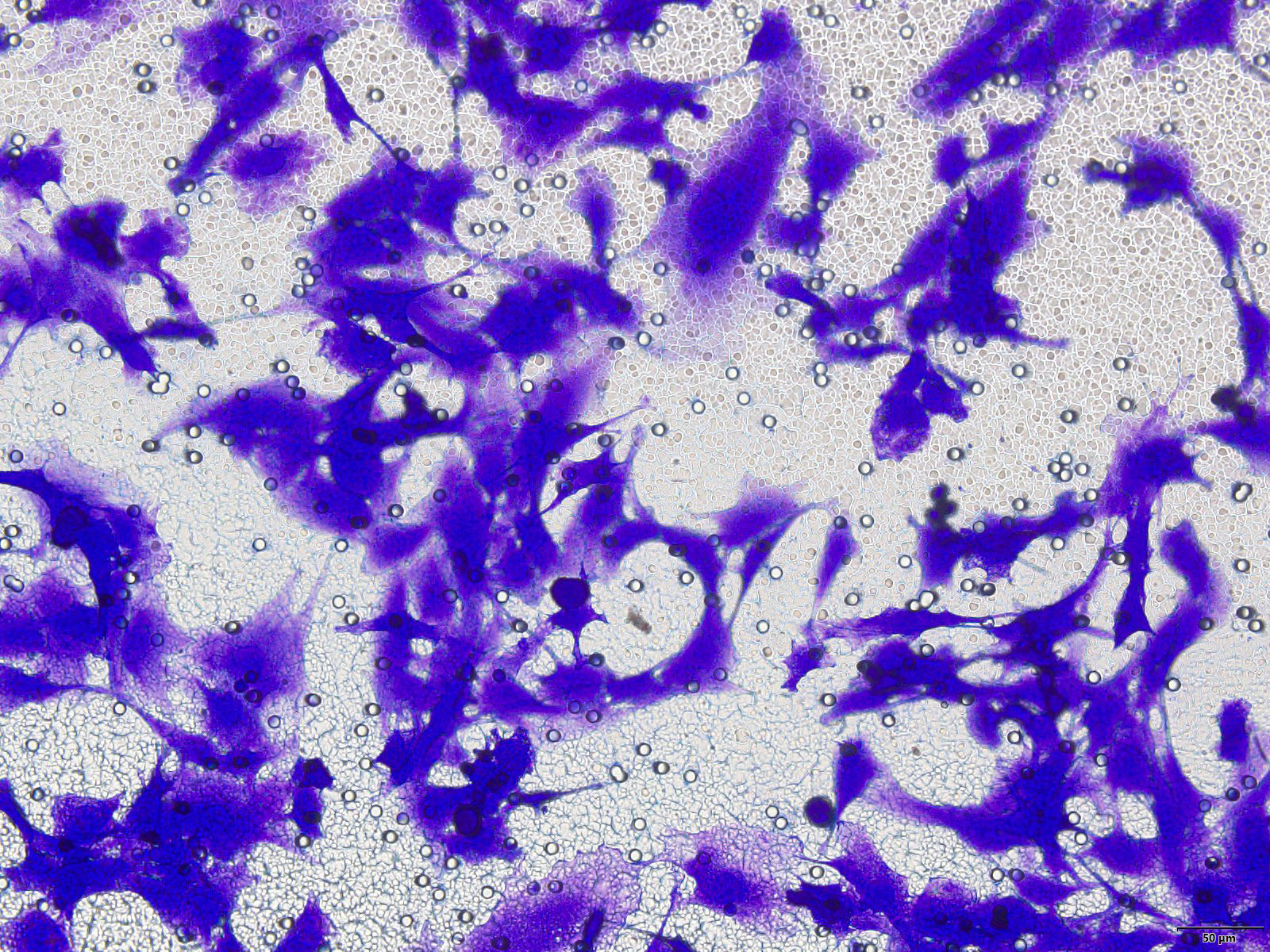

Supplement: Supplemental Information 3 [file peerj-13-19568-s003.zip › Figure 2A and 4C (Transwell)/miRNA inhibitors +si-NC (1).jpg]

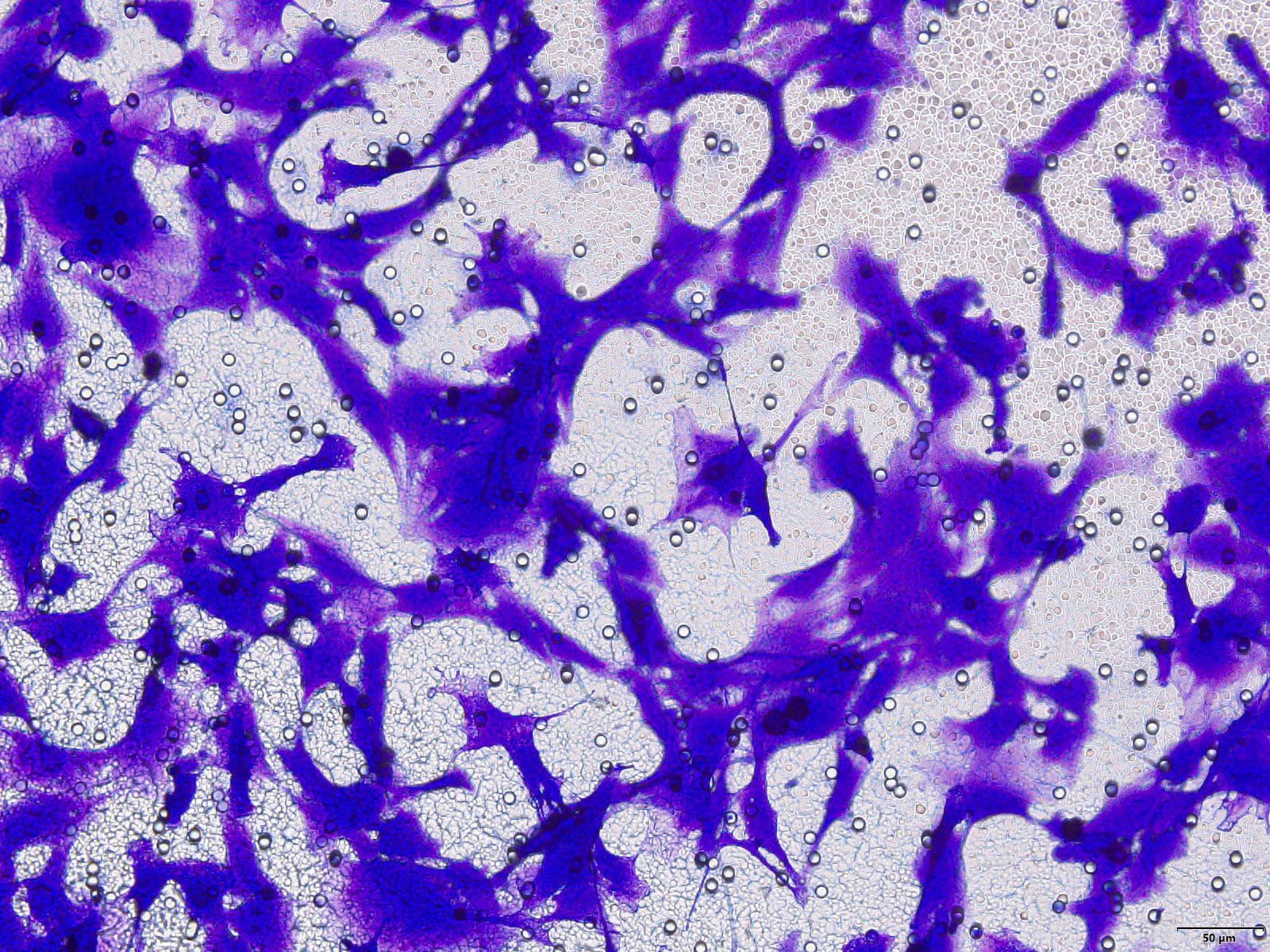

Supplement: Supplemental Information 3 [file peerj-13-19568-s003.zip › Figure 2A and 4C (Transwell)/miRNA inhibitors +si-NC (2).jpg]

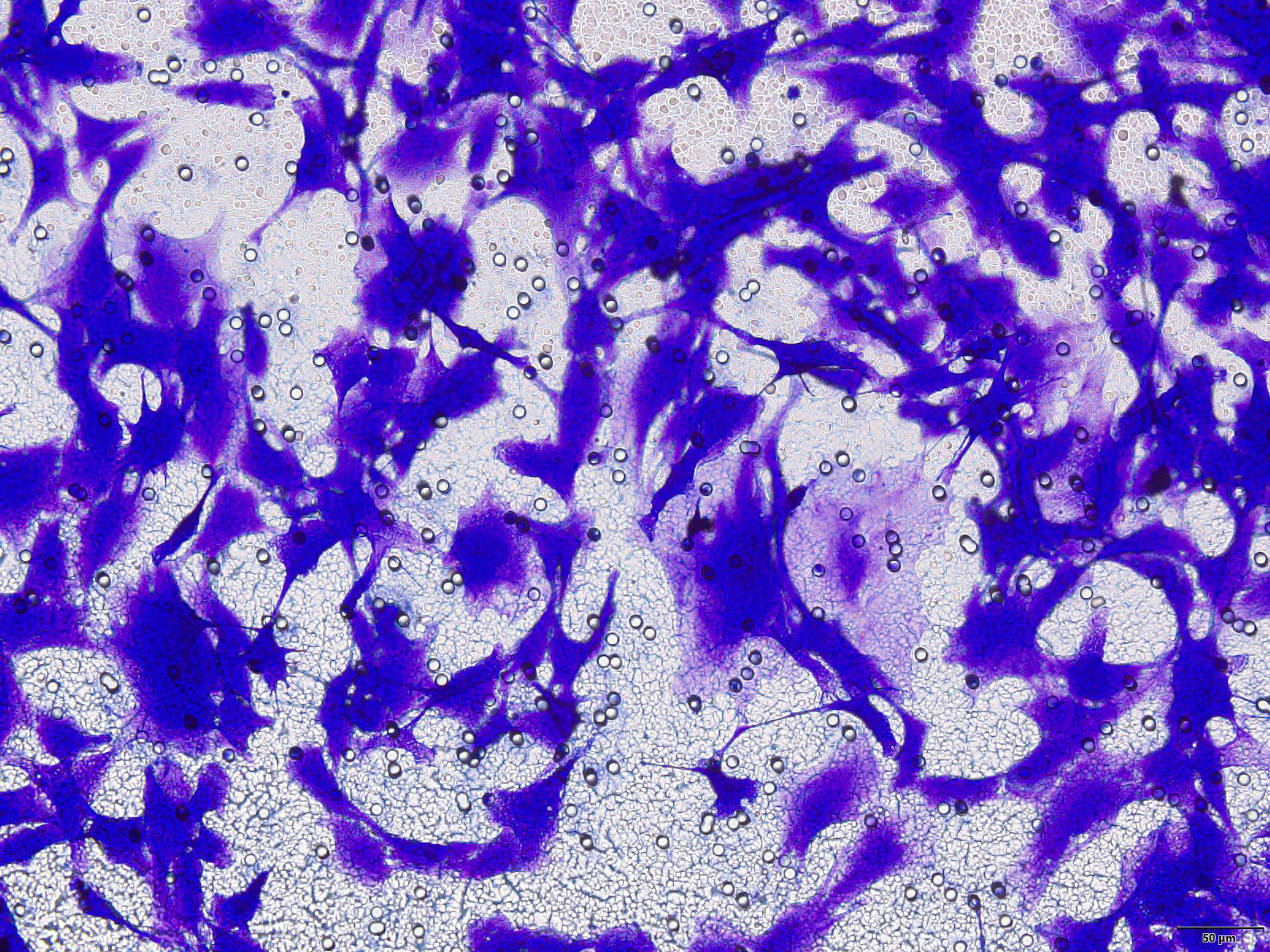

Supplement: Supplemental Information 3 [file peerj-13-19568-s003.zip › Figure 2A and 4C (Transwell)/miRNA inhibitors +si-NC (3).jpg]

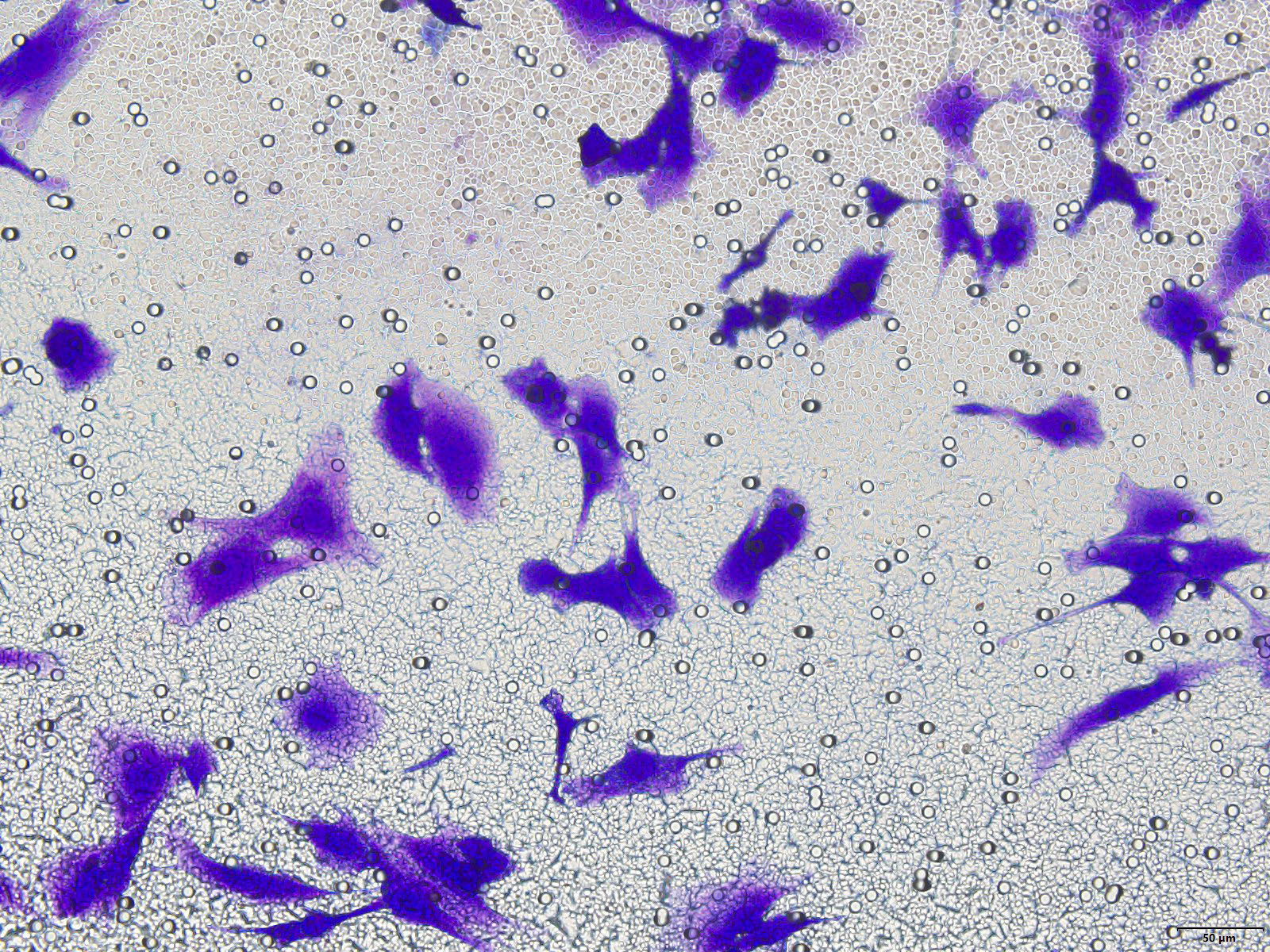

Supplement: Supplemental Information 3 [file peerj-13-19568-s003.zip › Figure 2A and 4C (Transwell)/miRNA inhibitors +si-vegf (1).jpg]

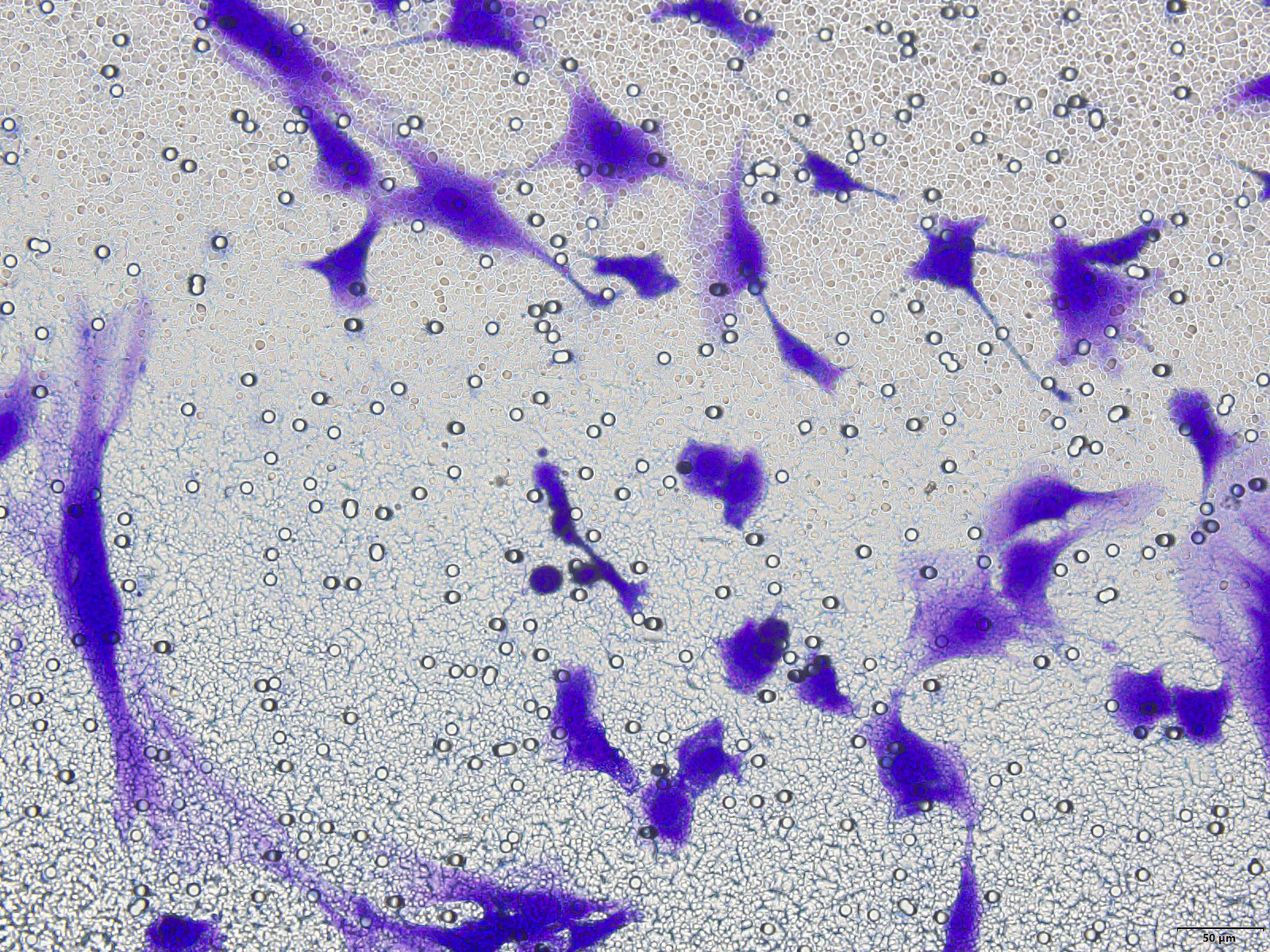

Supplement: Supplemental Information 3 [file peerj-13-19568-s003.zip › Figure 2A and 4C (Transwell)/miRNA inhibitors +si-vegf (2).jpg]

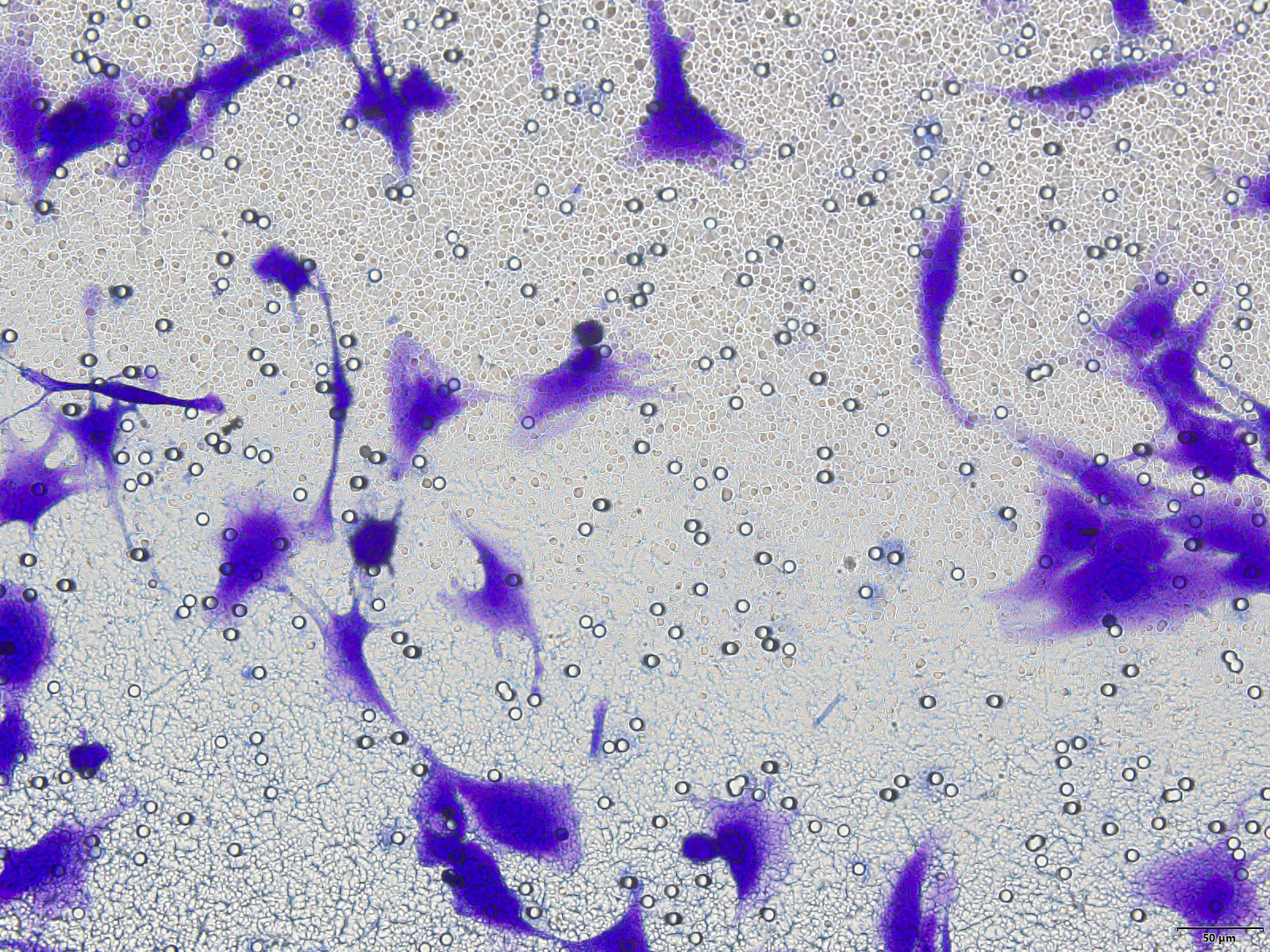

Supplement: Supplemental Information 3 [file peerj-13-19568-s003.zip › Figure 2A and 4C (Transwell)/miRNA inhibitors +si-vegf (3).jpg]

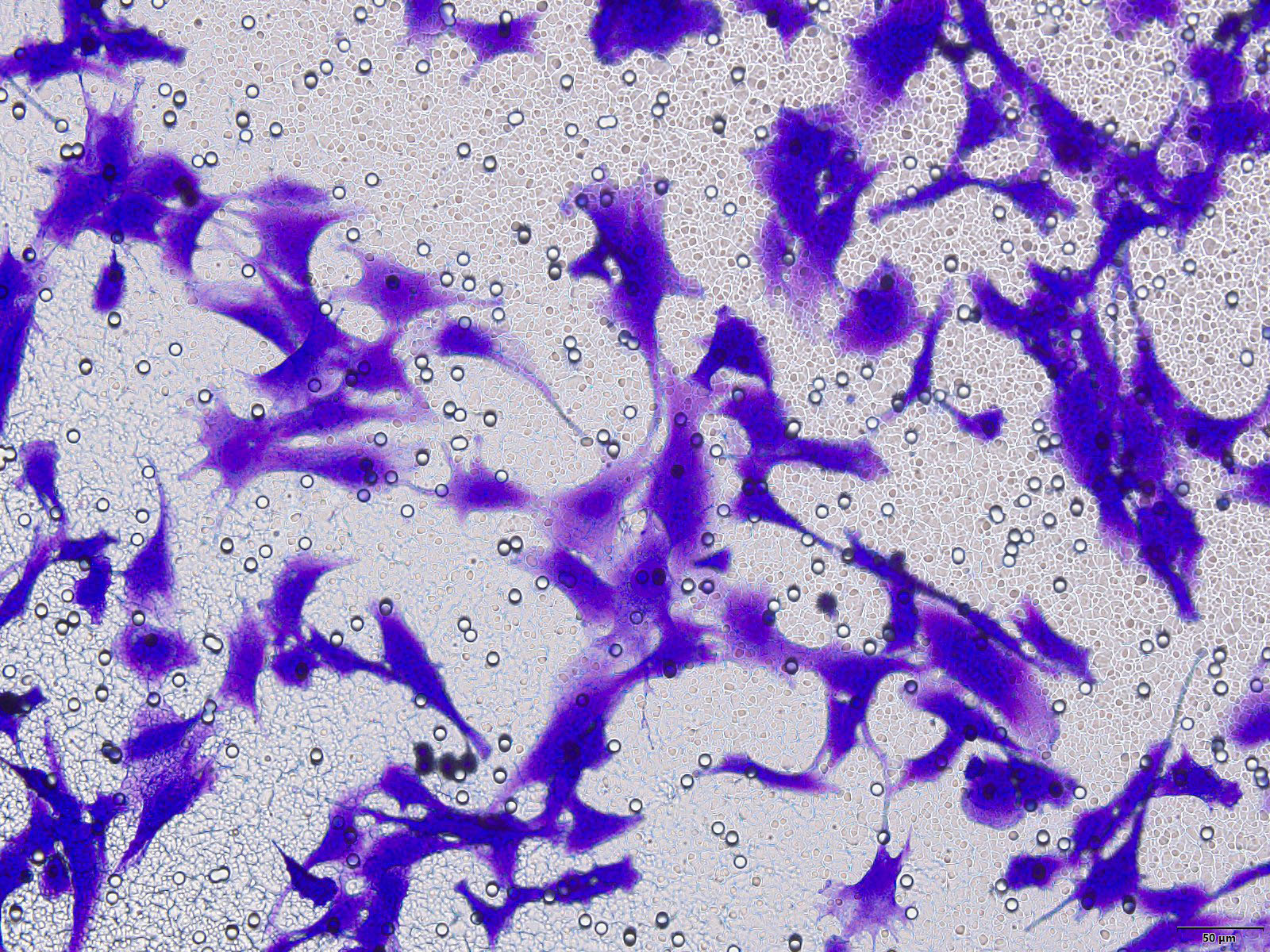

Supplement: Supplemental Information 3 [file peerj-13-19568-s003.zip › Figure 2A and 4C (Transwell)/miRNA inhibitors NC (1).jpg]

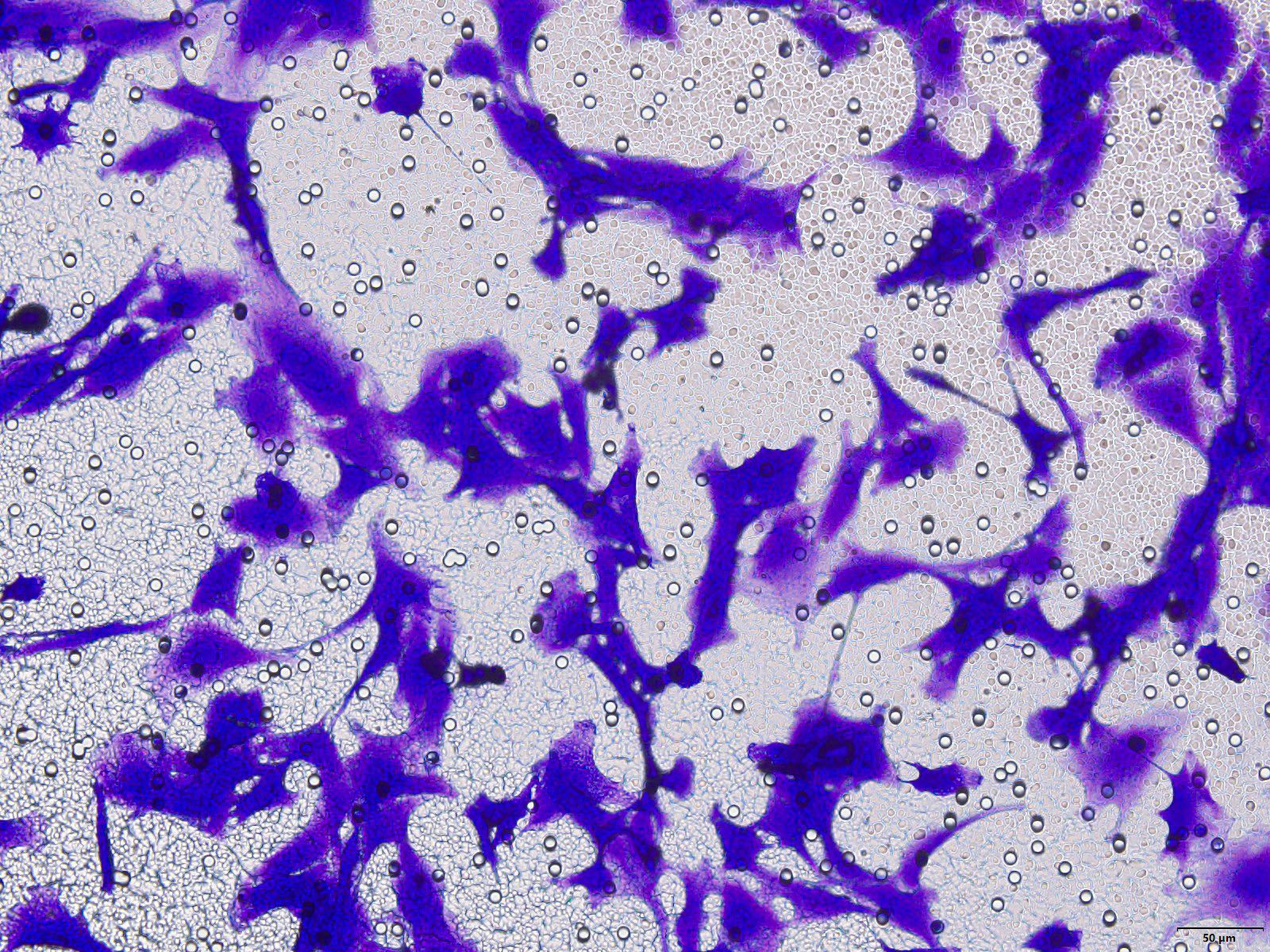

Supplement: Supplemental Information 3 [file peerj-13-19568-s003.zip › Figure 2A and 4C (Transwell)/miRNA inhibitors NC (2).jpg]

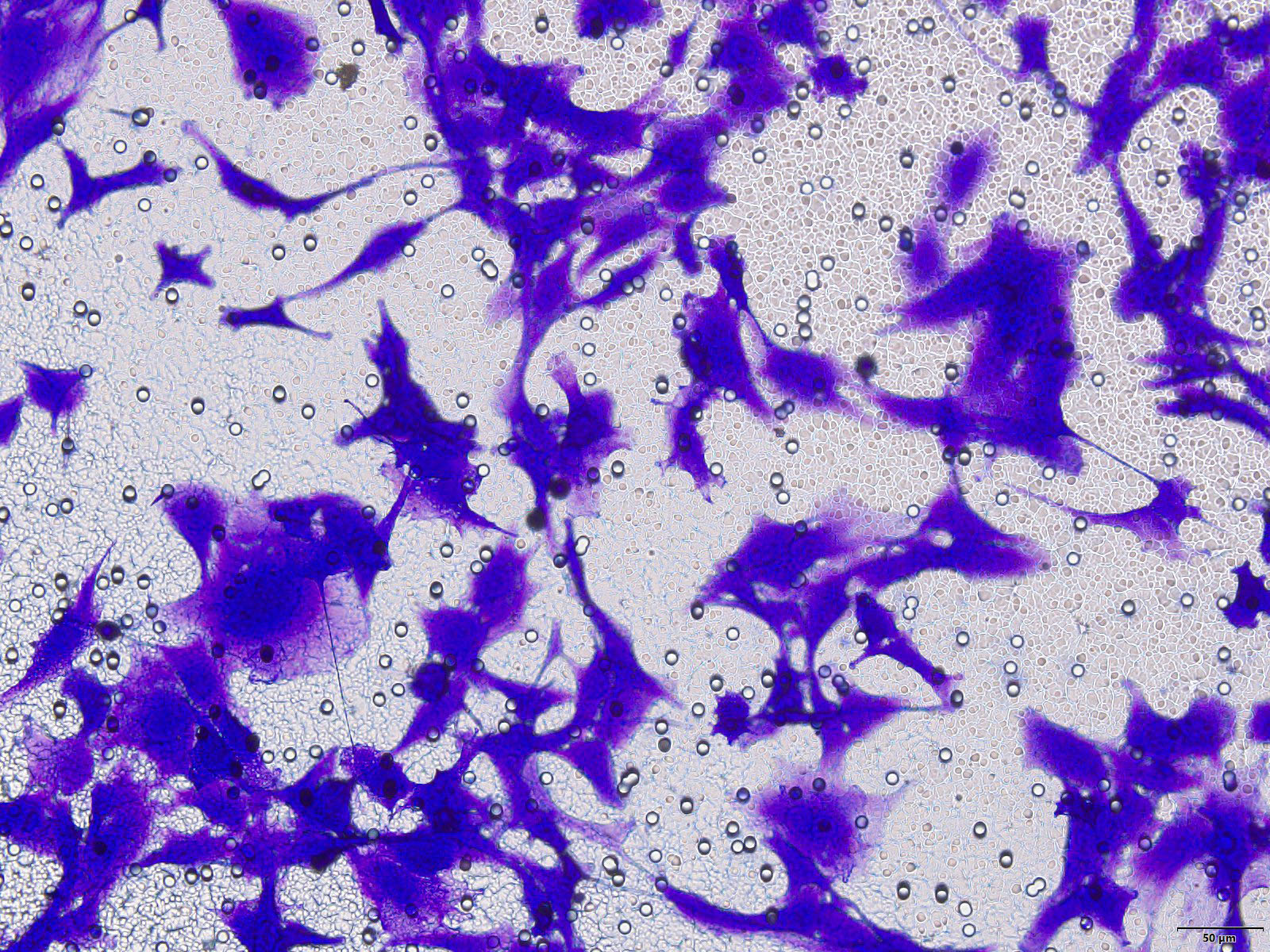

Supplement: Supplemental Information 3 [file peerj-13-19568-s003.zip › Figure 2A and 4C (Transwell)/miRNA inhibitors NC (3).jpg]

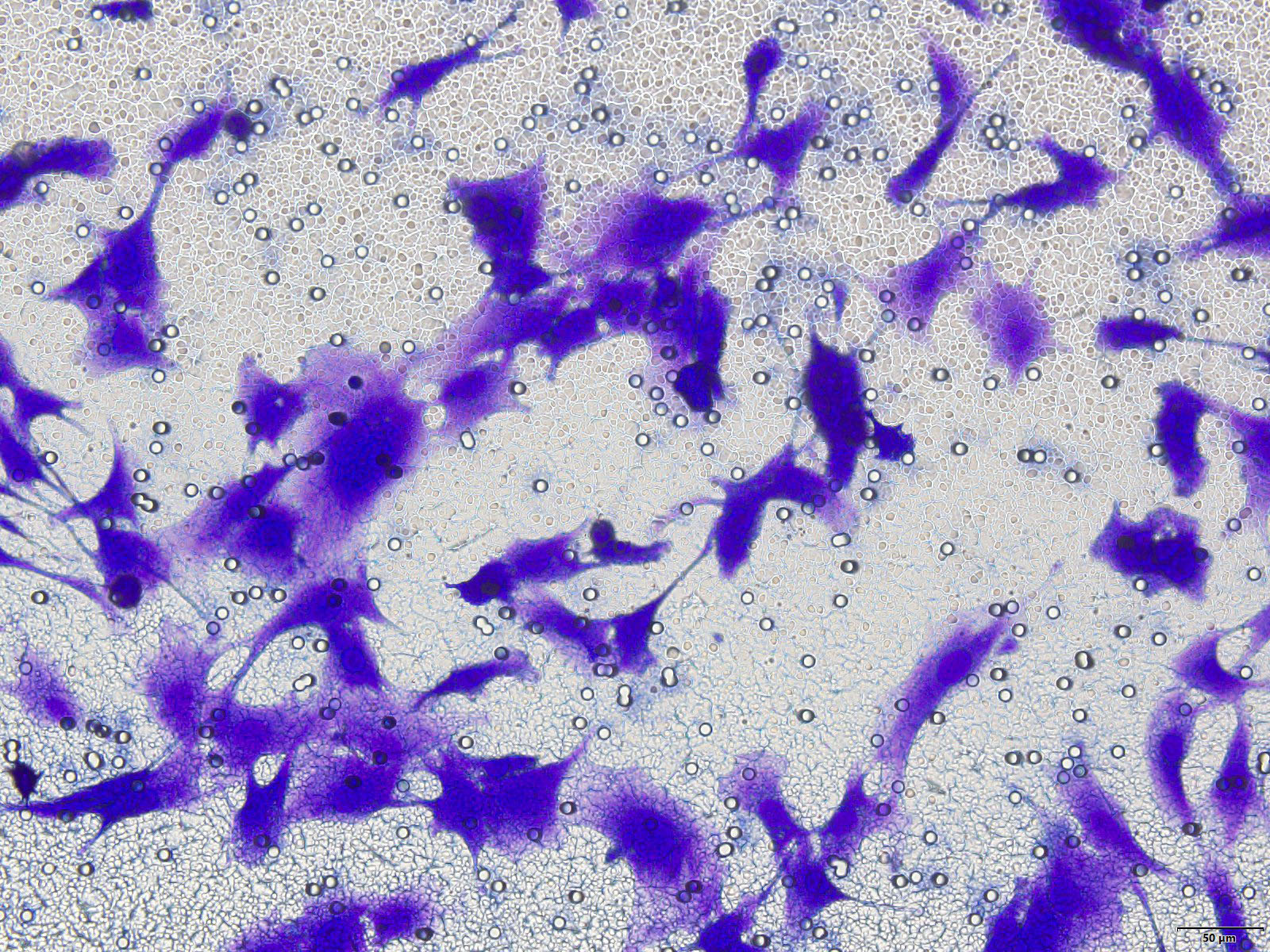

Supplement: Supplemental Information 3 [file peerj-13-19568-s003.zip › Figure 2A and 4C (Transwell)/miRNA mimics (1).jpg]

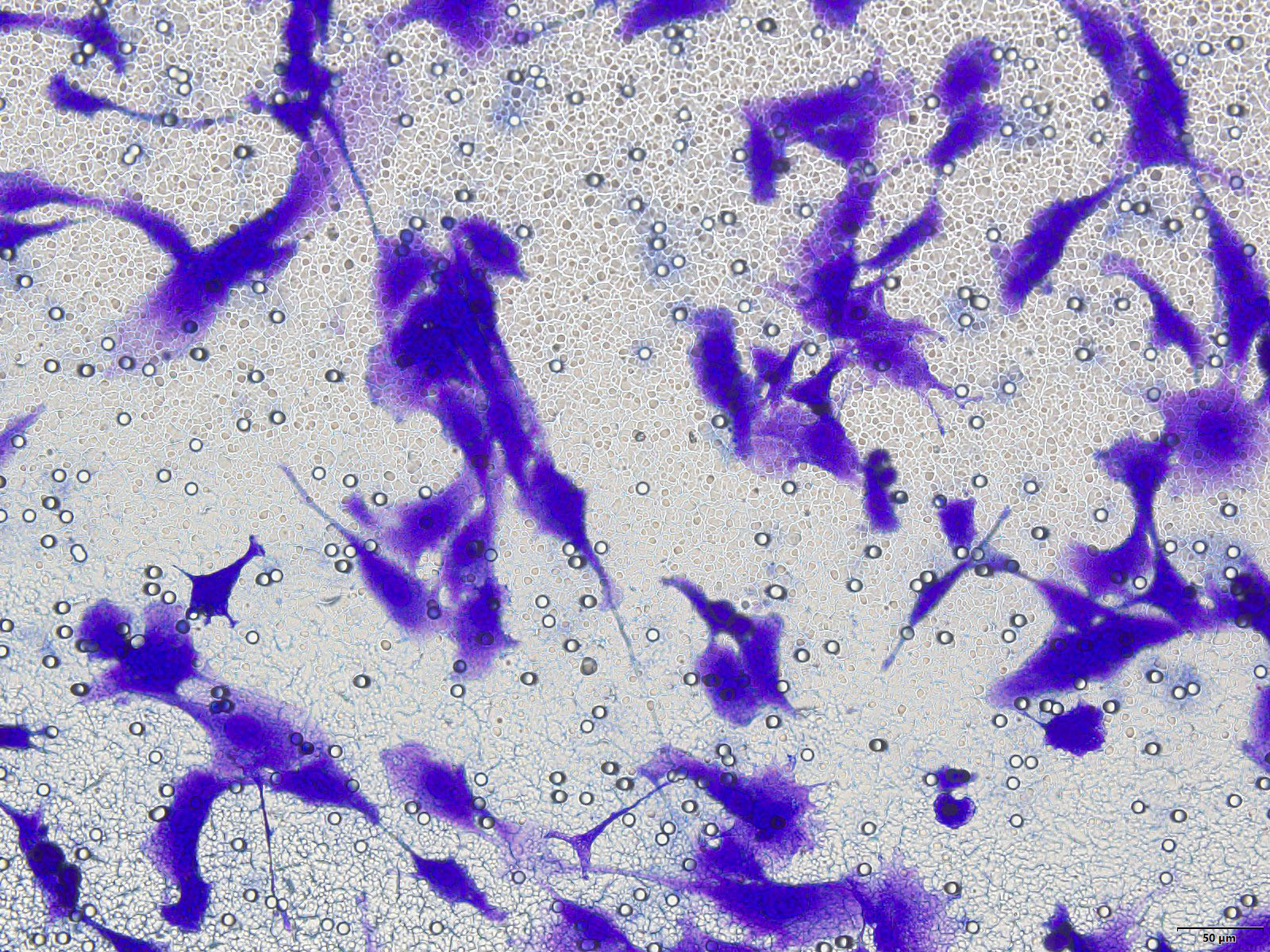

Supplement: Supplemental Information 3 [file peerj-13-19568-s003.zip › Figure 2A and 4C (Transwell)/miRNA mimics (2).jpg]

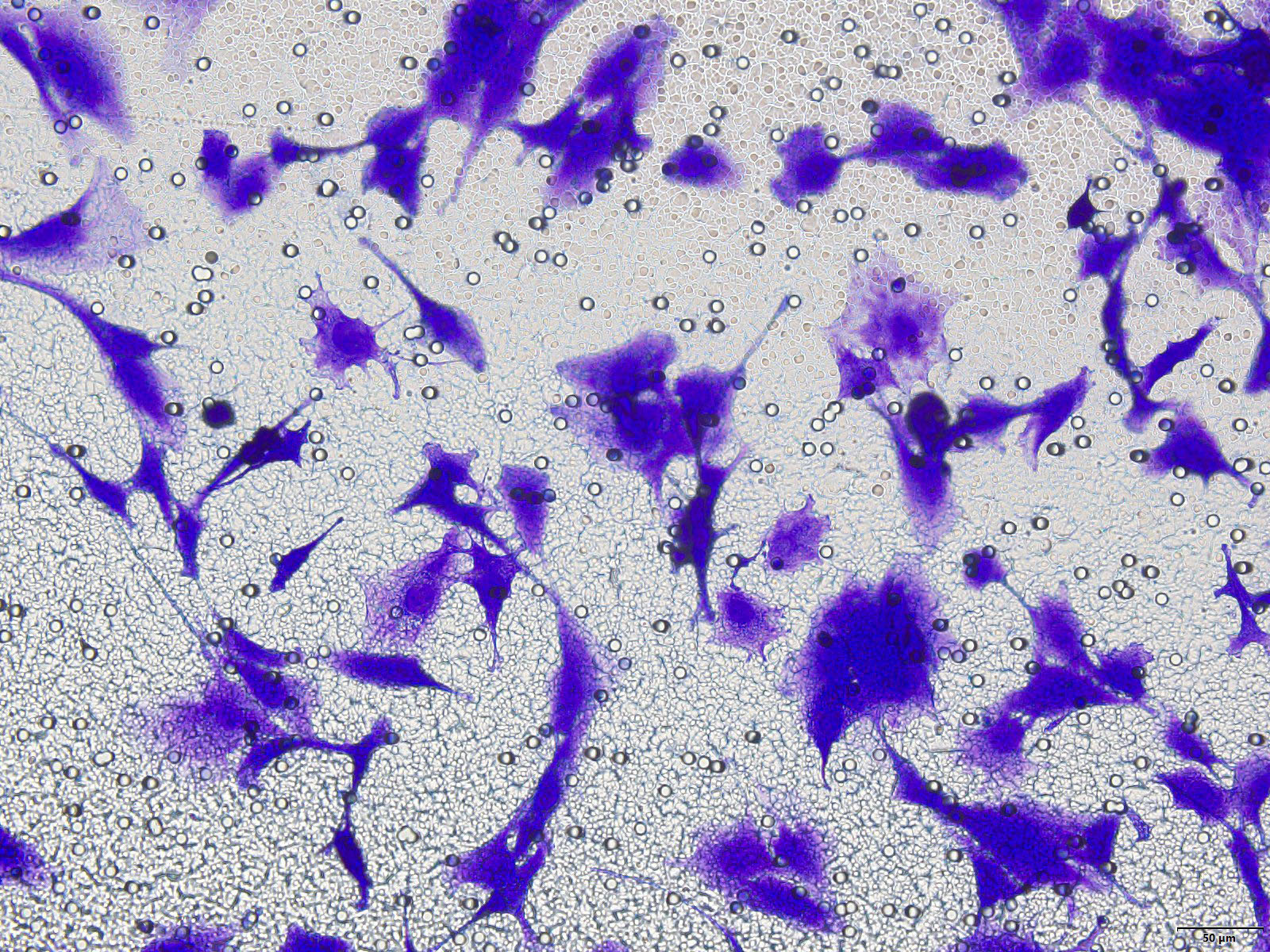

Supplement: Supplemental Information 3 [file peerj-13-19568-s003.zip › Figure 2A and 4C (Transwell)/miRNA mimics (3).jpg]

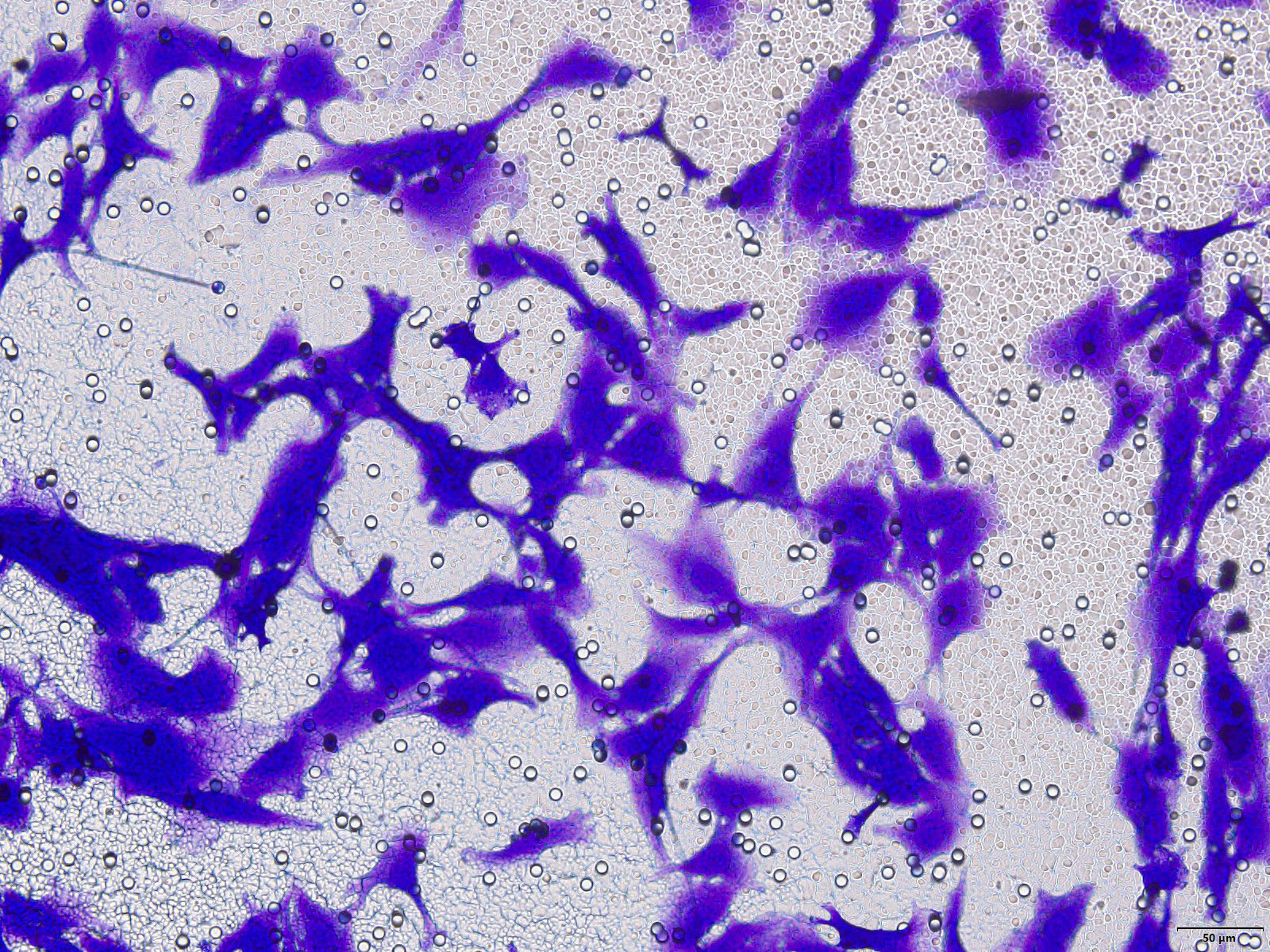

Supplement: Supplemental Information 3 [file peerj-13-19568-s003.zip › Figure 2A and 4C (Transwell)/miRNA mimics NC (1).jpg]

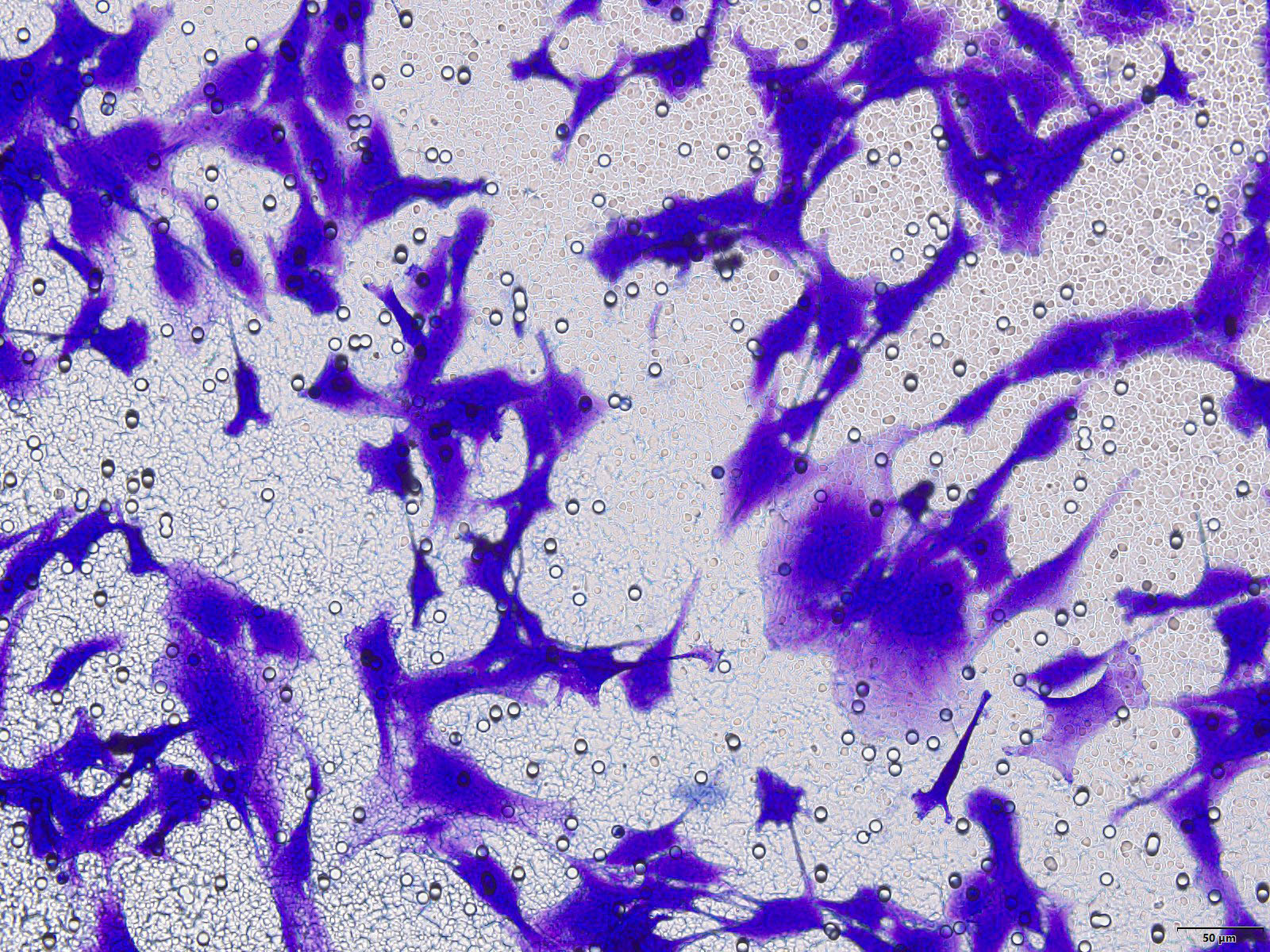

Supplement: Supplemental Information 3 [file peerj-13-19568-s003.zip › Figure 2A and 4C (Transwell)/miRNA mimics NC (2).jpg]

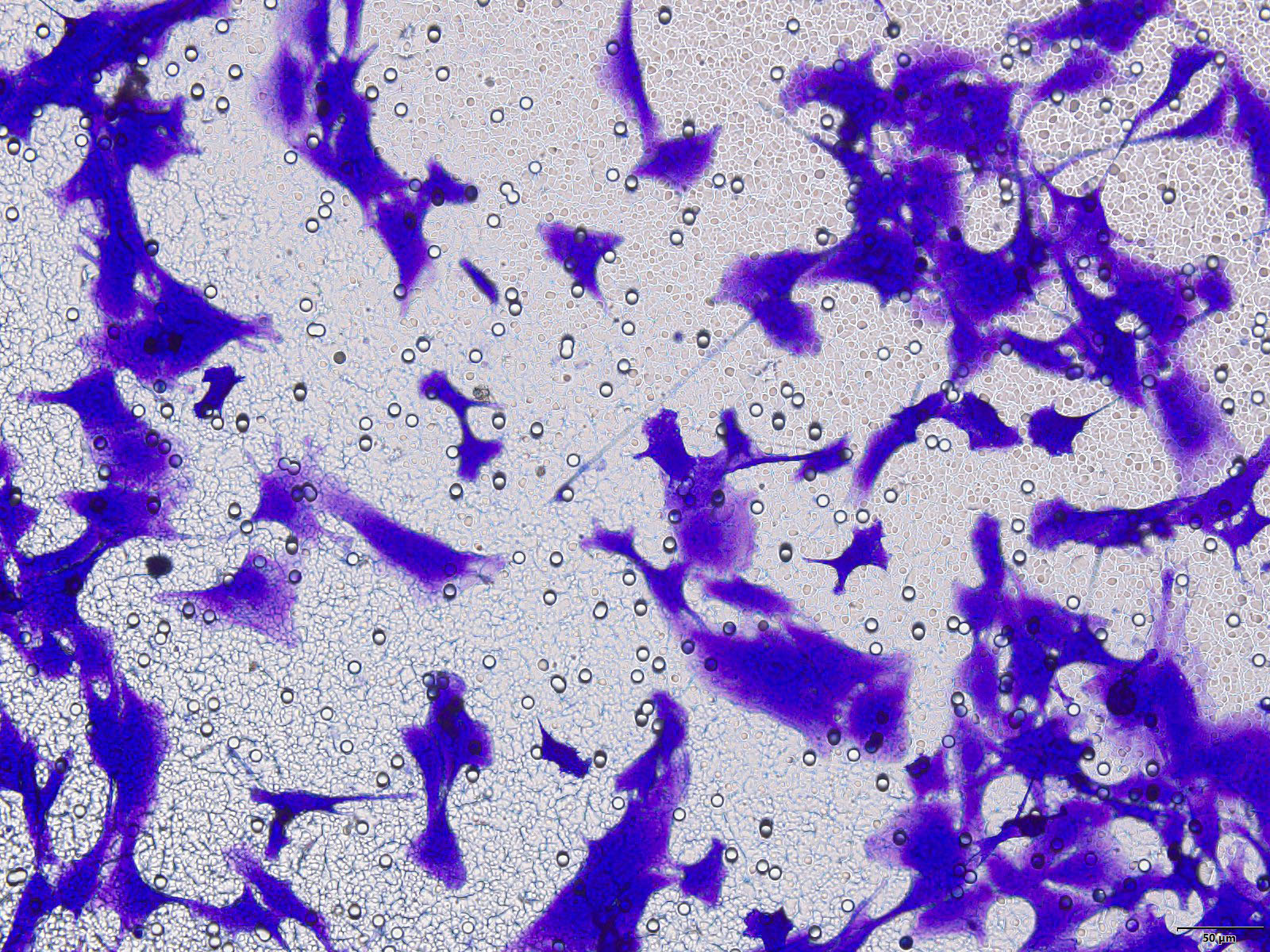

Supplement: Supplemental Information 3 [file peerj-13-19568-s003.zip › Figure 2A and 4C (Transwell)/miRNA mimics NC (3).jpg]

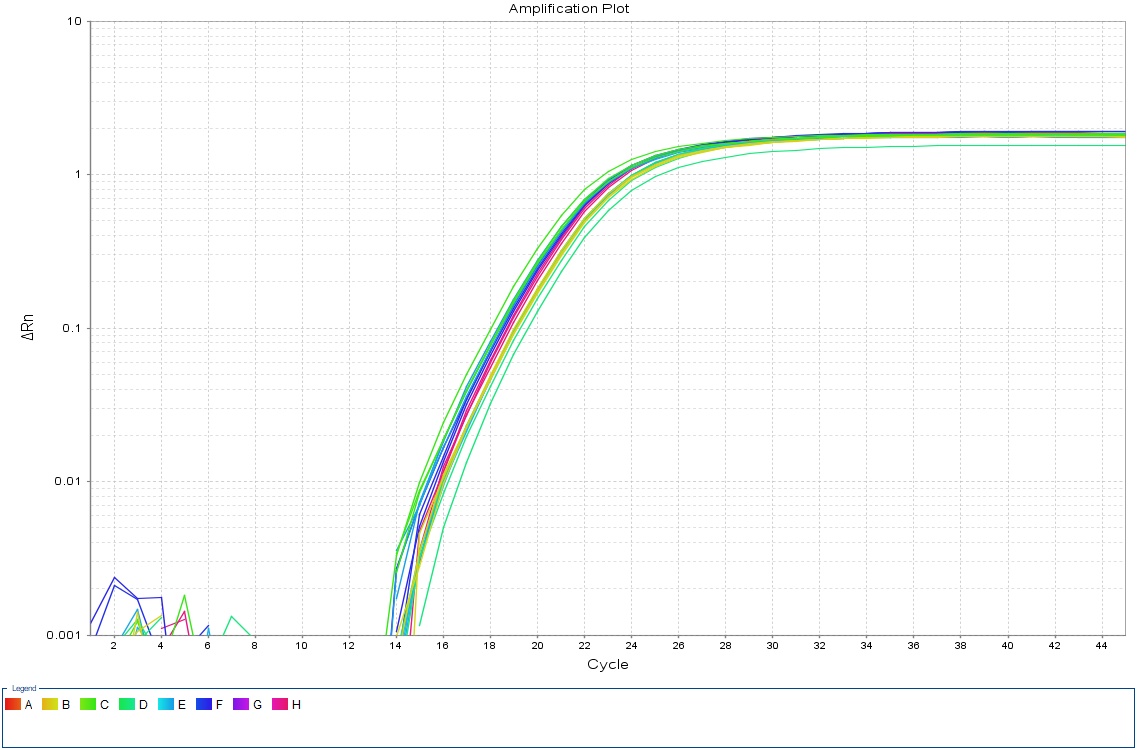

Supplement: Supplemental Information 4 [file peerj-13-19568-s004.zip › Figure 3A/curve/U6/Amplification Plot(LOG).jpg]

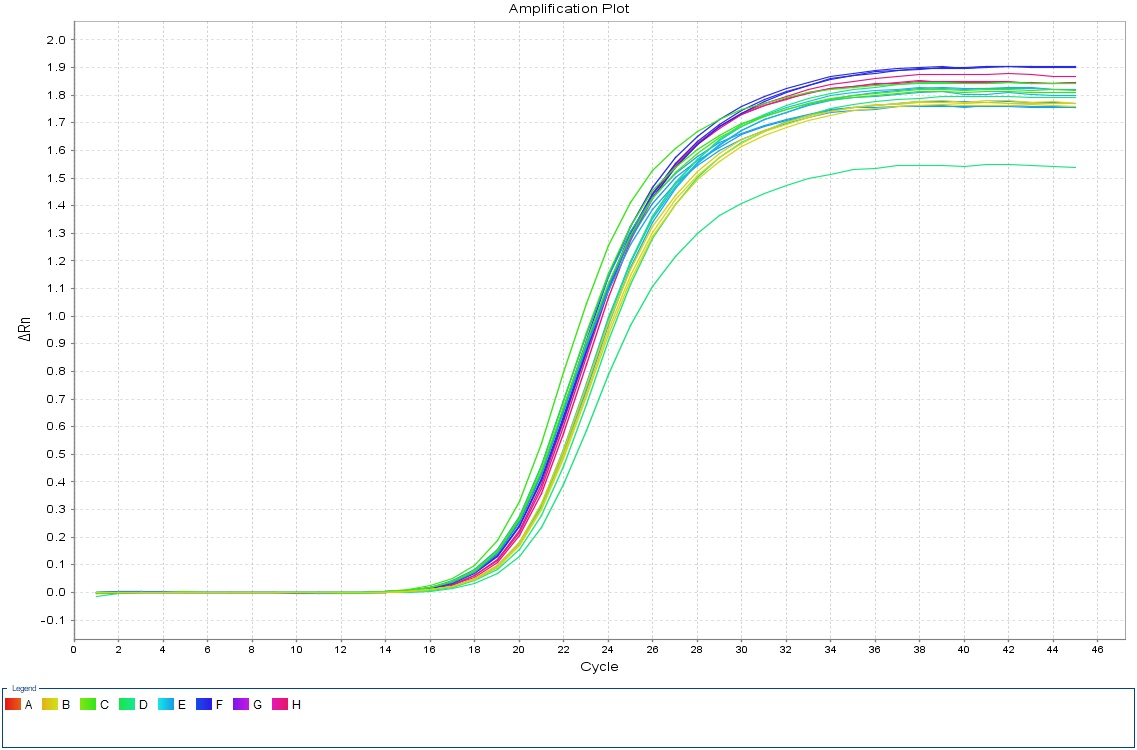

Supplement: Supplemental Information 4 [file peerj-13-19568-s004.zip › Figure 3A/curve/U6/Amplification Plot(linear).jpg]

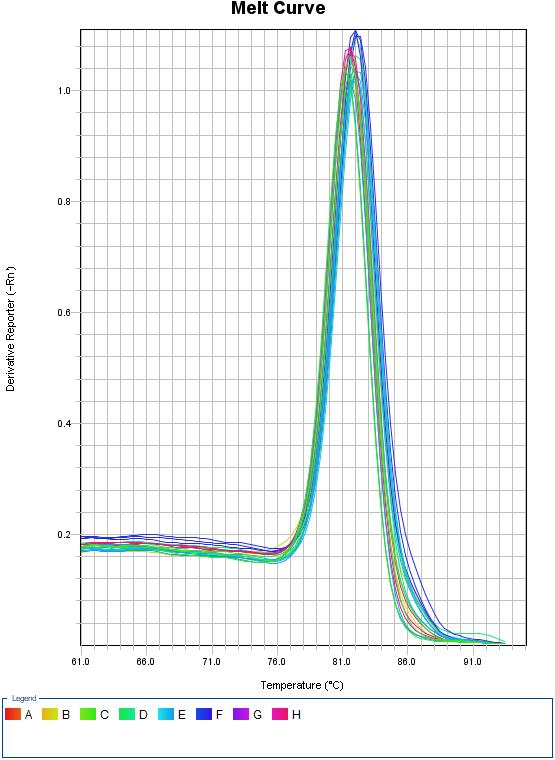

Supplement: Supplemental Information 4 [file peerj-13-19568-s004.zip › Figure 3A/curve/U6/Melt Curve.jpg]

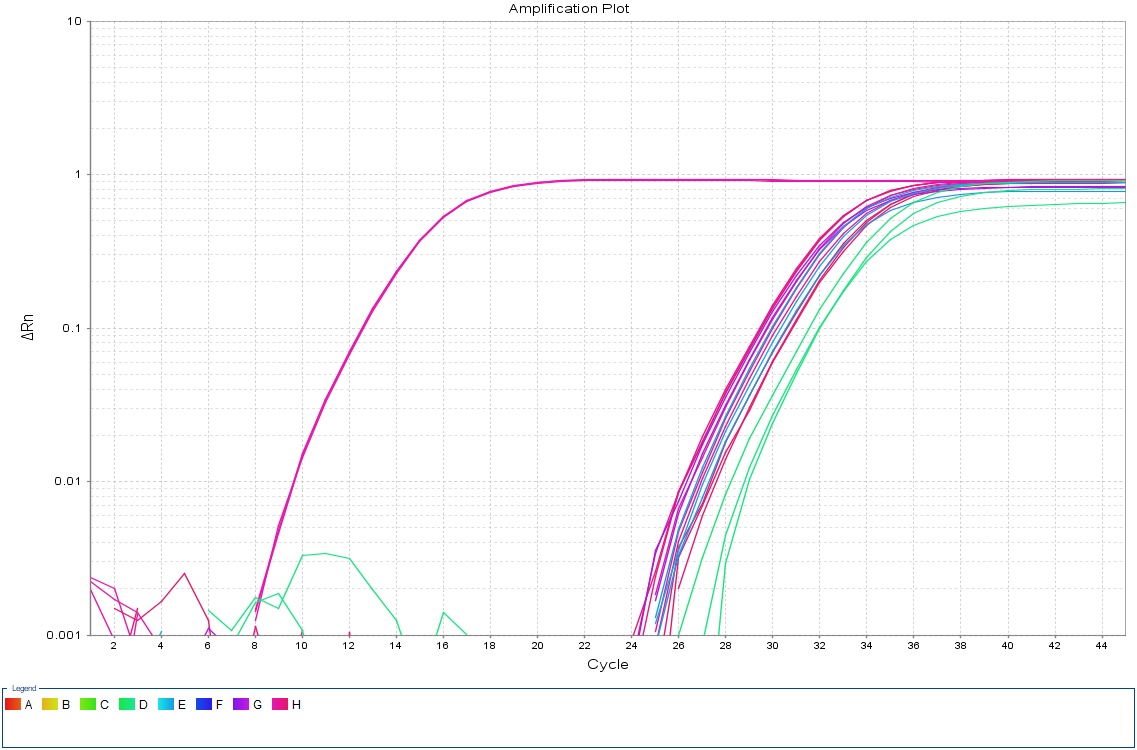

Supplement: Supplemental Information 4 [file peerj-13-19568-s004.zip › Figure 3A/curve/miRNA -381-3p/Amplification Plot(LOG).jpg]

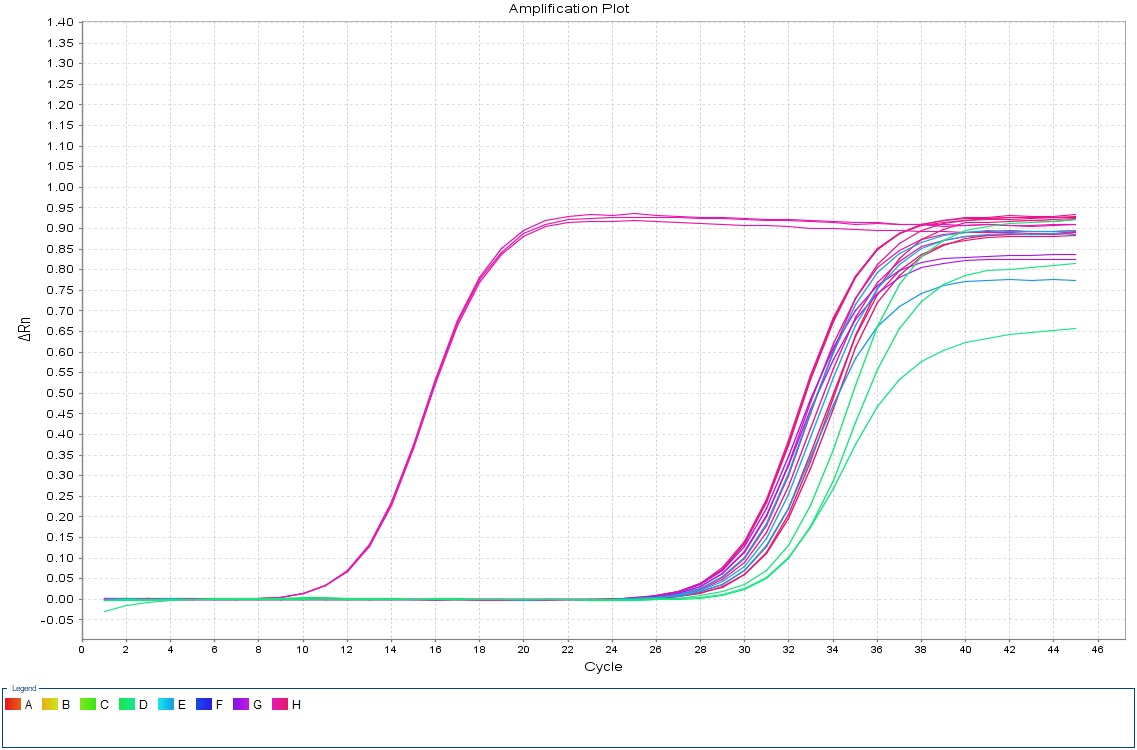

Supplement: Supplemental Information 4 [file peerj-13-19568-s004.zip › Figure 3A/curve/miRNA -381-3p/Amplification Plot(linear).jpg]

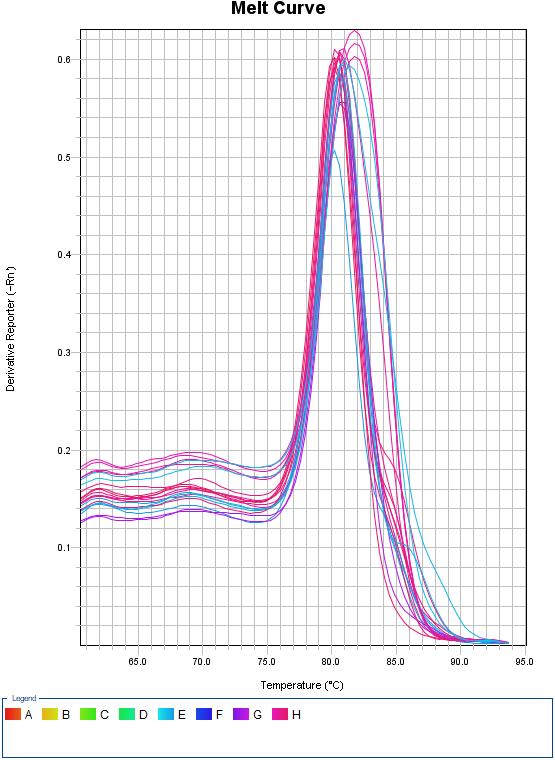

Supplement: Supplemental Information 4 [file peerj-13-19568-s004.zip › Figure 3A/curve/miRNA -381-3p/Melt Curve.jpg]

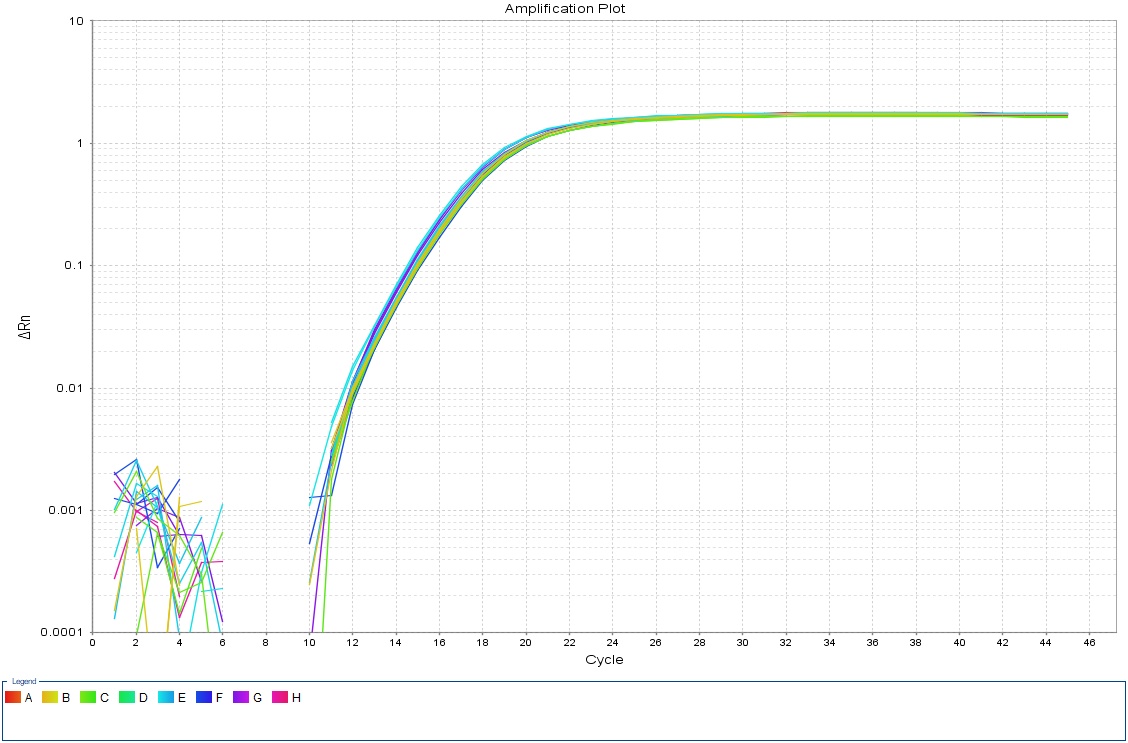

Supplement: Supplemental Information 5 [file peerj-13-19568-s005.zip › Figure 3B/CURVE/GAPDH/Amplification Plot(LOG).jpg]

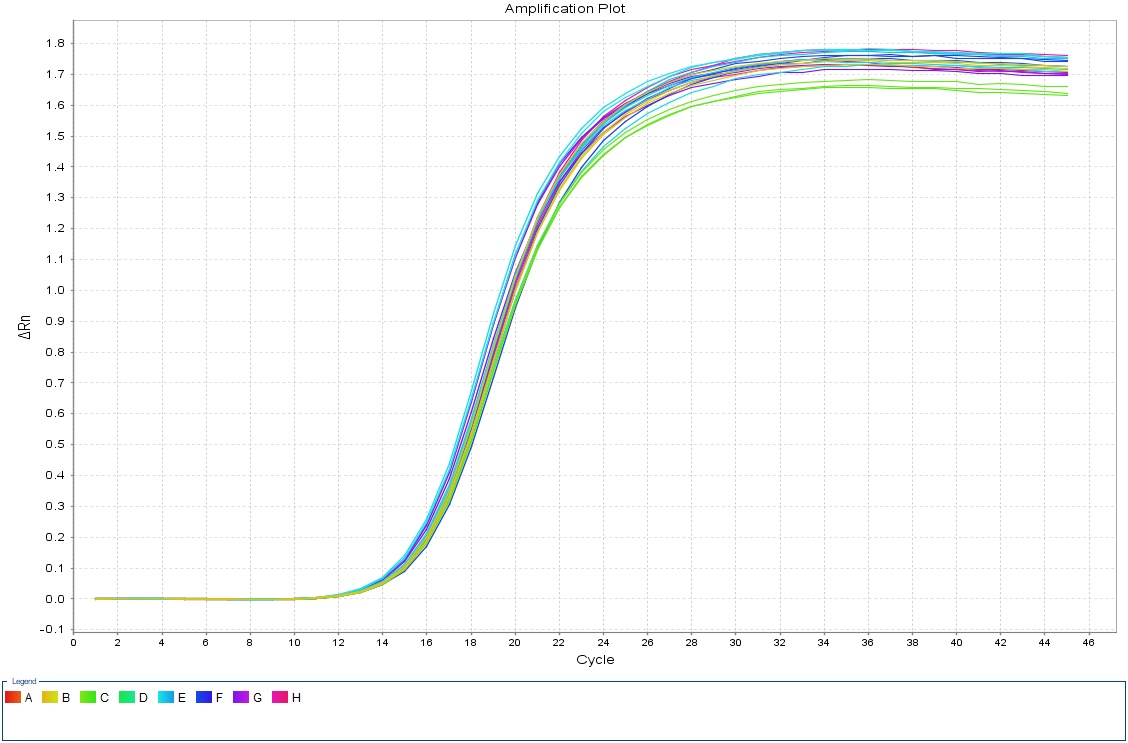

Supplement: Supplemental Information 5 [file peerj-13-19568-s005.zip › Figure 3B/CURVE/GAPDH/Amplification Plot(linear).jpg]

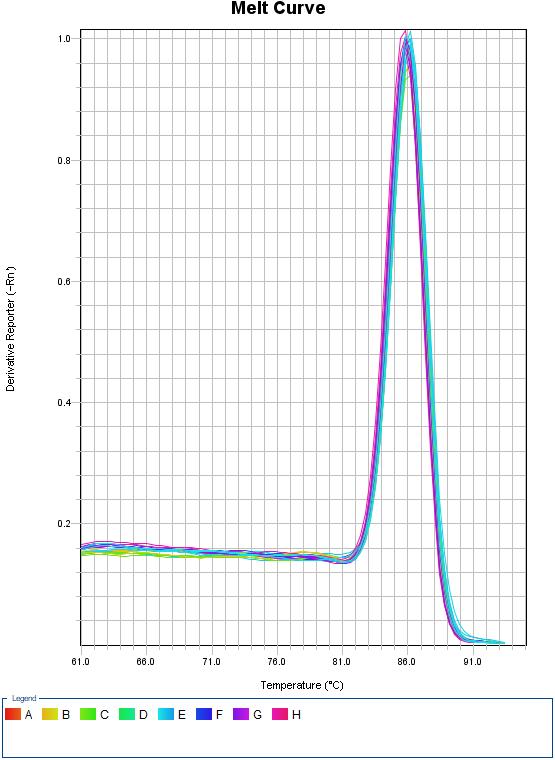

Supplement: Supplemental Information 5 [file peerj-13-19568-s005.zip › Figure 3B/CURVE/GAPDH/Melt Curve.jpg]

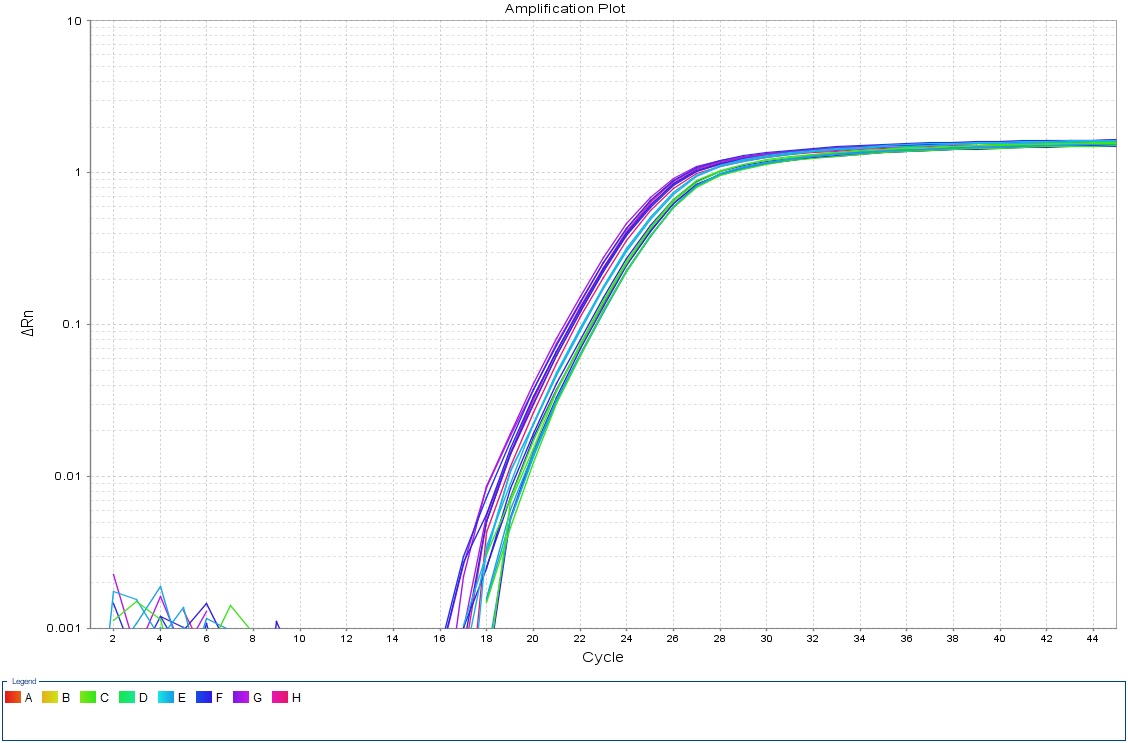

Supplement: Supplemental Information 5 [file peerj-13-19568-s005.zip › Figure 3B/CURVE/VEGFA/Amplification Plot(LOG).jpg]

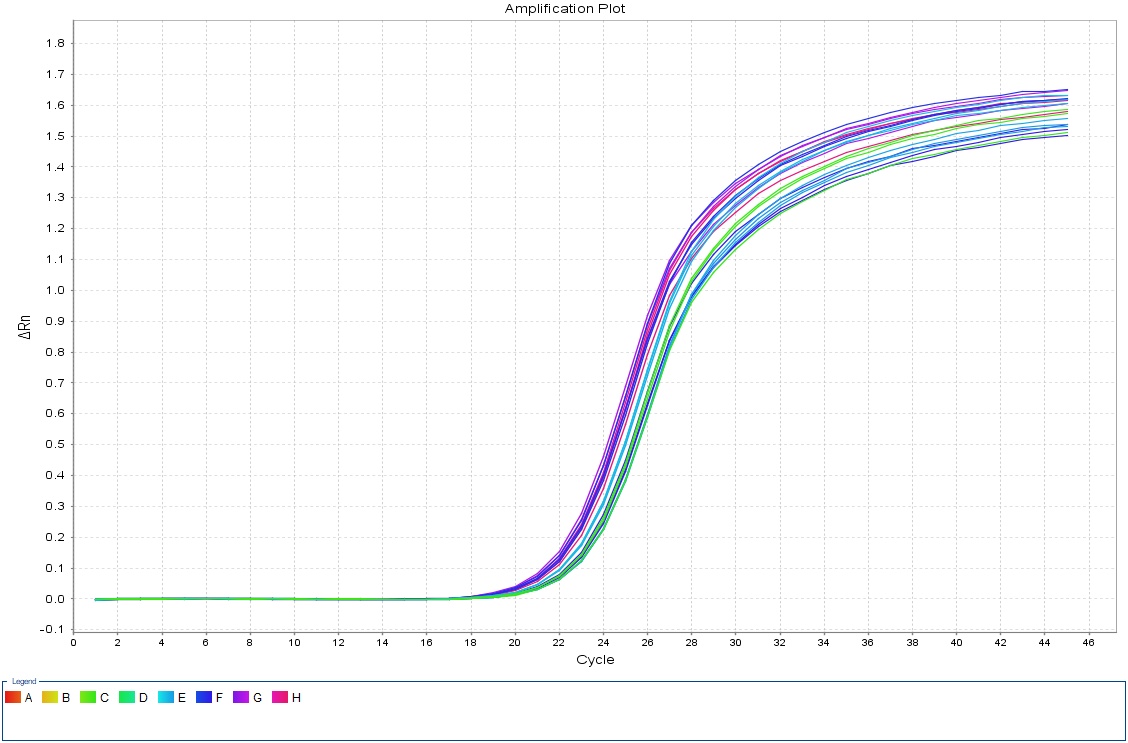

Supplement: Supplemental Information 5 [file peerj-13-19568-s005.zip › Figure 3B/CURVE/VEGFA/Amplification Plot(linear).jpg]

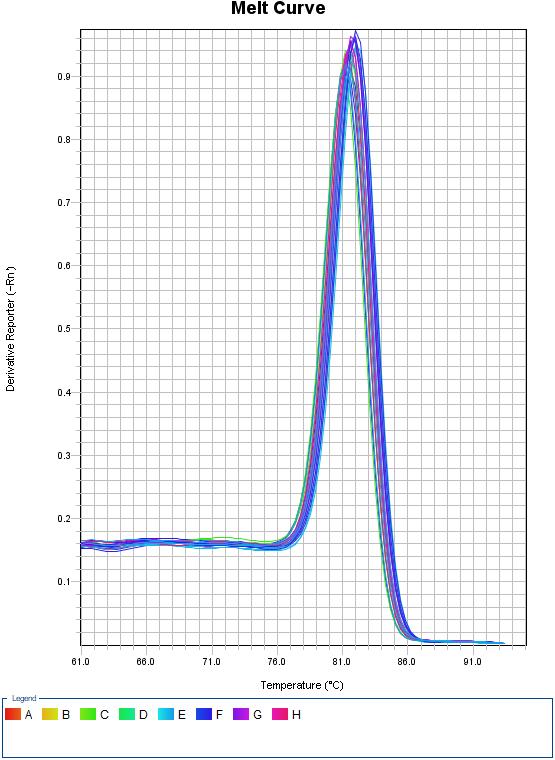

Supplement: Supplemental Information 5 [file peerj-13-19568-s005.zip › Figure 3B/CURVE/VEGFA/Melt Curve.jpg]

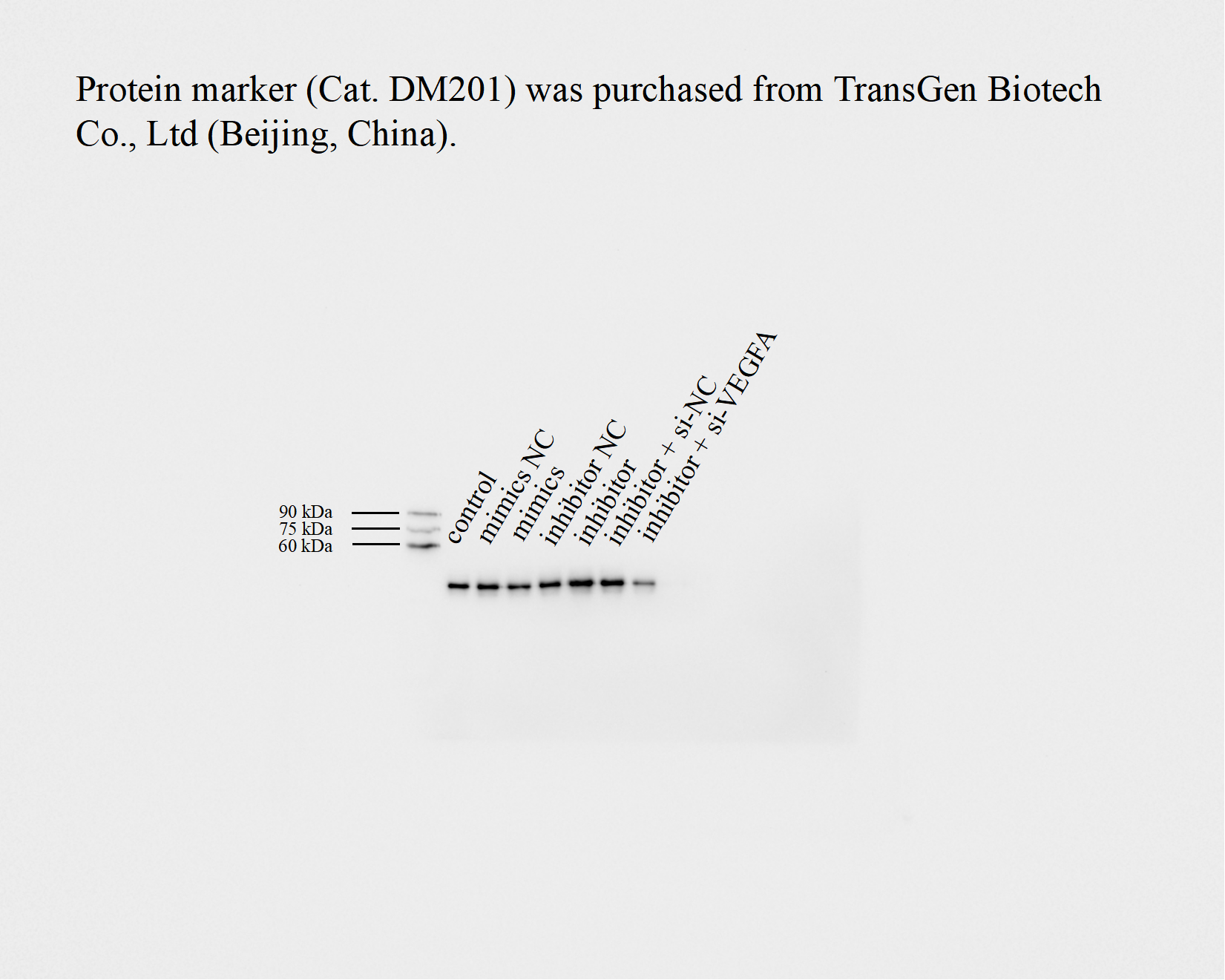

Supplement: Supplemental Information 6 [file peerj-13-19568-s006.zip › Figure 3C/Description.png]

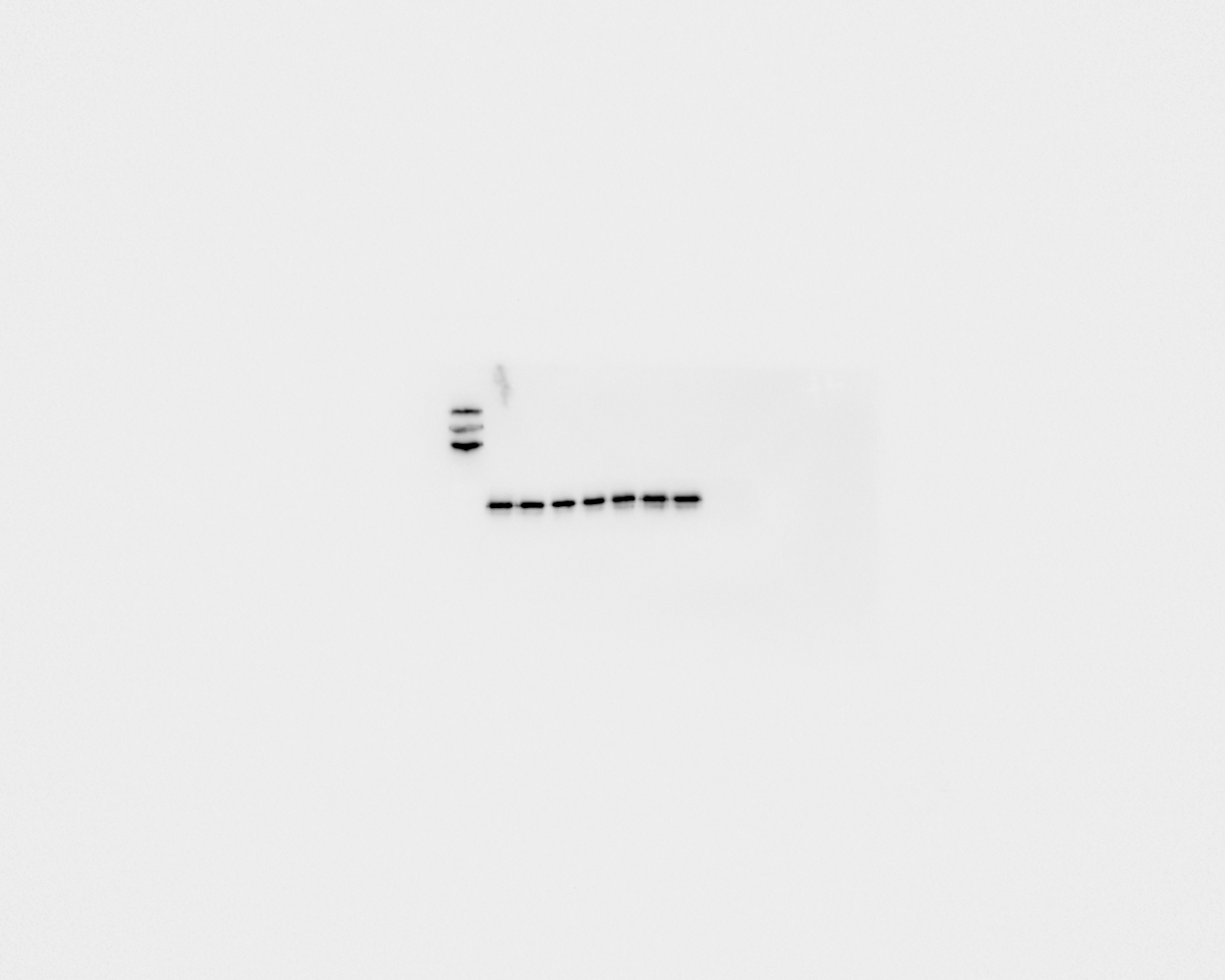

Supplement: Supplemental Information 6 [file peerj-13-19568-s006.zip › Figure 3C/GAPDH.tif]

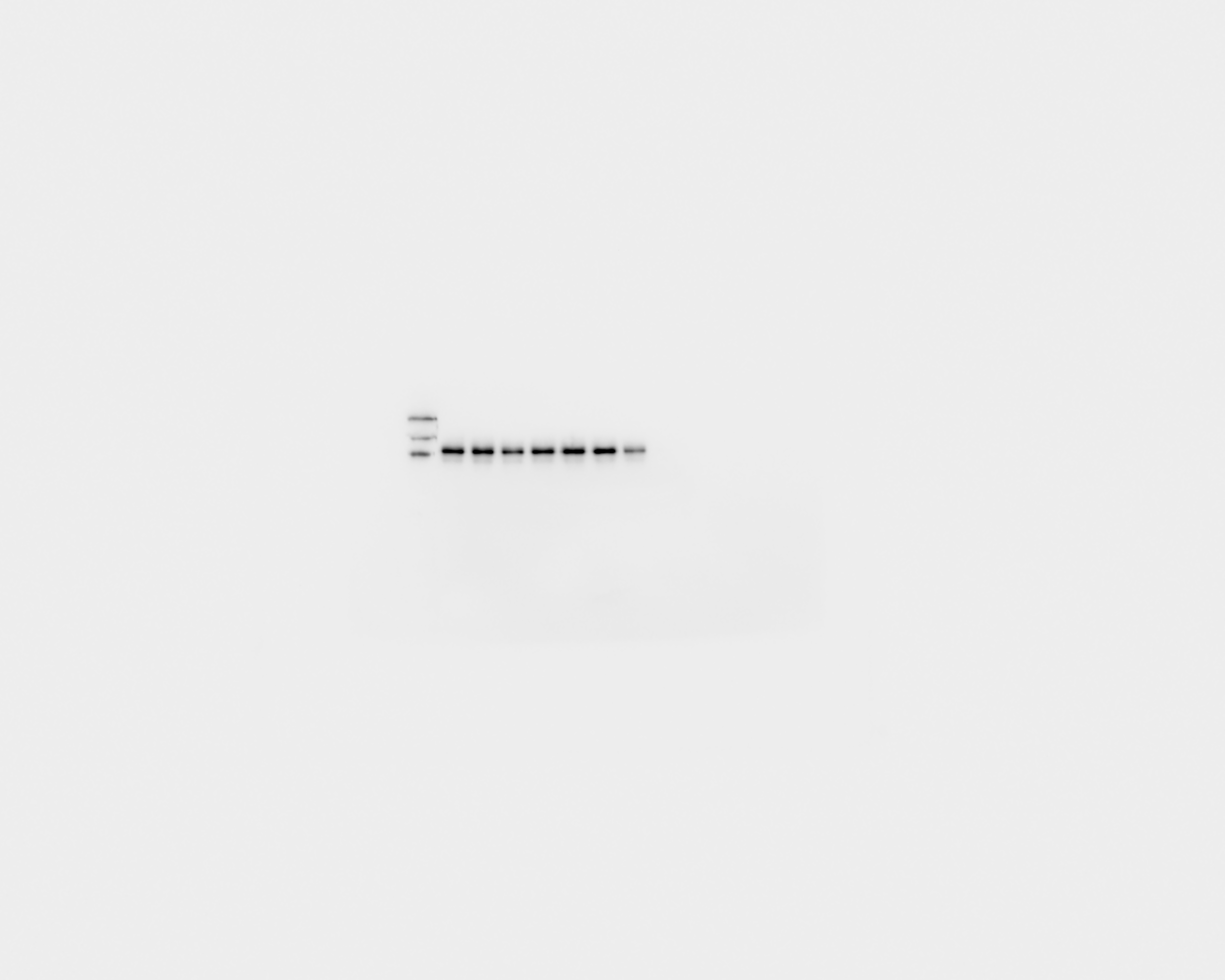

Supplement: Supplemental Information 6 [file peerj-13-19568-s006.zip › Figure 3C/P-P65.tif]

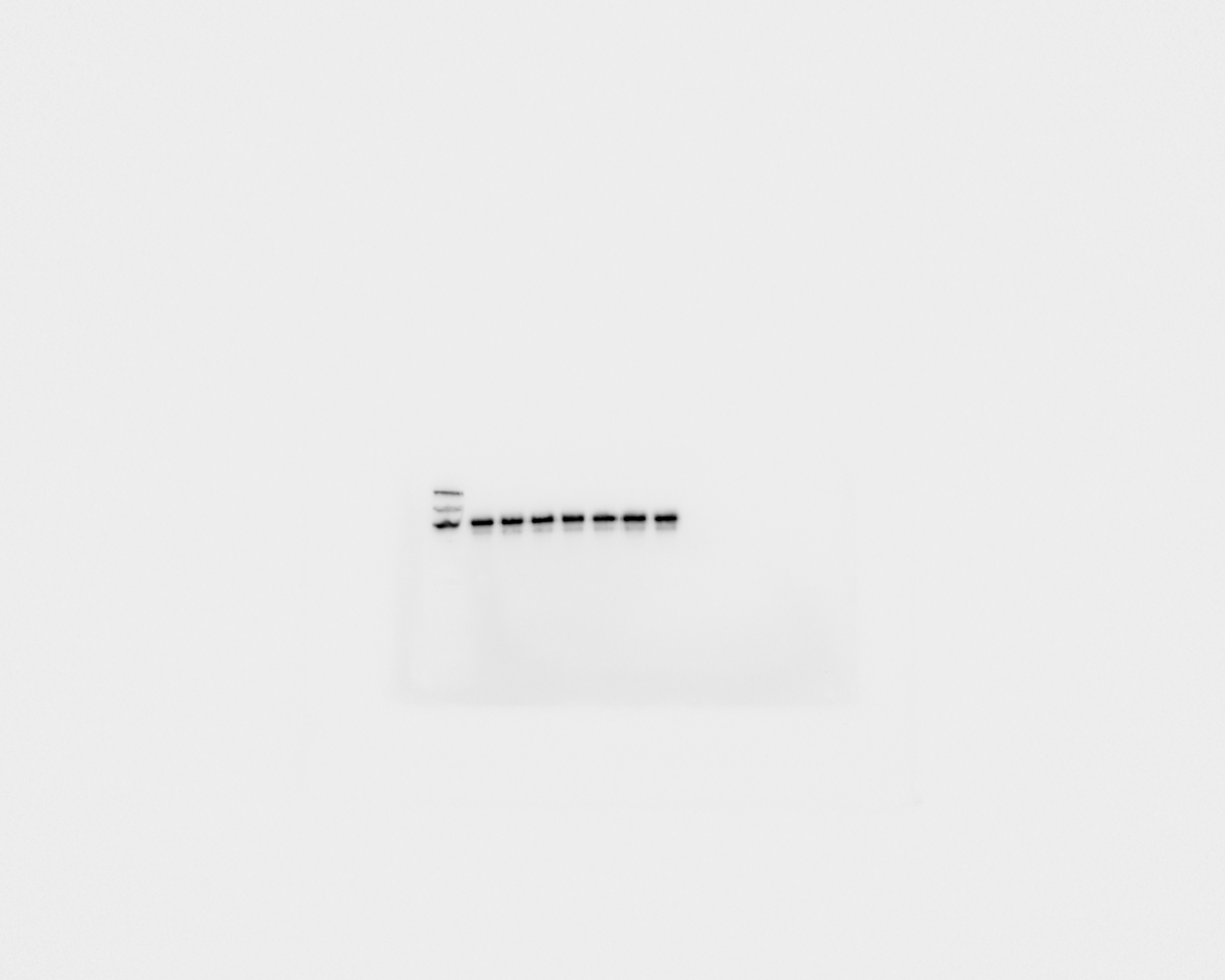

Supplement: Supplemental Information 6 [file peerj-13-19568-s006.zip › Figure 3C/P65.tif]
