# Supplementary material for: miR-381-3p contribution in mouse spontaneous abortion via targeting VEGFA
Source: PeerJ. 2025 Jun 24;13:e19568. doi: 10.7717/peerj.19568 (PMC12204090; doi:10.7717/peerj.19568)
Supplement: Supplemental Information 4 [file peerj-13-19568-s004.zip › Figure 3A/primer/U6/U6.docx]

U6 (94bp)

Forward：CTCGCTTCGGCAGCACA

Reverse：AACGCTTCACGAATTTGCGT

AAGGTCGGGCAGGAAGAGGGCCTATTTCCCATGATTCCTTCATATTTGCATATACGATACAAGGCTGTTAGAGAGATAATTAGAATTAATTTGACTGTAAACACAAAGATATTAGTACAAAATACGTGACGTAGAAAGTAATAATTTCTTGGGTAGTTTGCAGTTTTTAAAATTATGTTTTAAAATGGACTATCATATGCTTACCGTAACTTGAAAGTATTTCGATTTCTTGGCTTTATATATCTTGTGGAAAGGACGAAACACCGTGCTCGCTTCGGCAGCACATATACTAAAATTGGAACGATACAGAGAAGATTAGCATGGCCCCTGCGCAAGGATGACACGCAAATTCGTGAAGCGTTCCATATTTTTACATCAGGTTGTTTTTCTGTTTTTACATCAGGTTGTTTTTCTGTTTGGTTTTTTTTTTACACCACGTTTATACGCCGGTGCACGGTTTACCA
