# Supplementary material for: miR-381-3p contribution in mouse spontaneous abortion via targeting VEGFA
Source: PeerJ. 2025 Jun 24;13:e19568. doi: 10.7717/peerj.19568 (PMC12204090; doi:10.7717/peerj.19568)
Supplement: Supplemental Information 5 [file peerj-13-19568-s005.zip › Figure 3B/RT-qPCR details.docx]

**Main reagents and instruments**

TRIzol（Cat. 15596-026, Invitrogen, USA）; Chloroform (Nanjing Chemical Reagent Co., Ltd., Nanjing, China); isopropyl alcohol (Nanjing Chemical Reagent Co., Ltd.); absolute ethyl alcohol (Nanjing Chemical Reagent Co., Ltd.); DEPC Water (Cat. KGF2201, Jiangsu KeyGEN Biotechnology Co., Ltd., China); PrimeScript^TM^ RT Mix (Cat. RR036B, TaKaRa); SYBR Green qPCR Mix (Cat. RR820A, TaKaRa).

Ultraviolet-visible spectrophotometer (UV-2450, SHIMADZU); Veriti 96 well Thermal cycler PCR system (ABI, USA); 7500 Real time-PCR system (ABI).

**RNA extraction**

Total RNA was extracted using TRIzol Reagent (Invitrogen, USA) according to the manufacturer’s instructions.

**Determination of** **RNA concentration and purity**

Total RNA concentration and purity were measured by ultraviolet-visible spectrophotometer (UV-2450, SHIMADZU). The optical density (OD) was presented at 260 nm and 280 nm. RNA concentration = OD_260_ × dilution folds × 0.04 μg/μL. OD_260_/OD_280_ in the range of 1.8-2.1 was considered as high purity RNA.

**RT-qPCR reaction system**

| Composition | Volume (μL) |
| --- | --- |
| 2**×** Realltime PCR Master Mix(SYBR Green) | 10 |
| cDNA (10 x dilution) | 1 |
| Forward primer (10 μM) | 1 |
| Reverse primer (10 μM) | 1 |
| RNase-free water | 7 |
