# Supplementary figures and images for: miR-381-3p contribution in mouse spontaneous abortion via targeting VEGFA (part 2 of 2)
Source: PeerJ. 2025 Jun 24;13:e19568. doi: 10.7717/peerj.19568 (PMC12204090; doi:10.7717/peerj.19568)

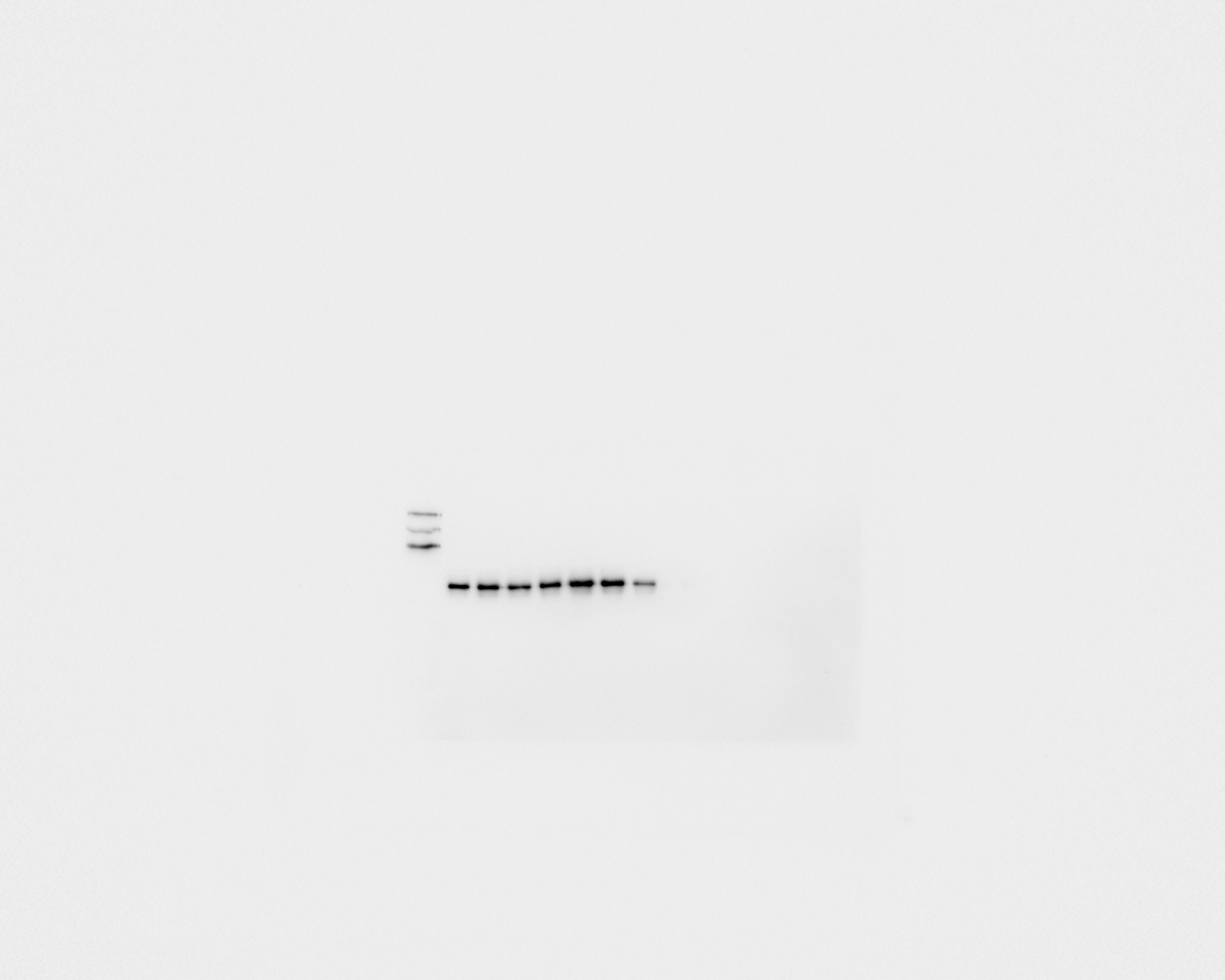

Supplement: Supplemental Information 6 [file peerj-13-19568-s006.zip › Figure 3C/VEGF.tif]

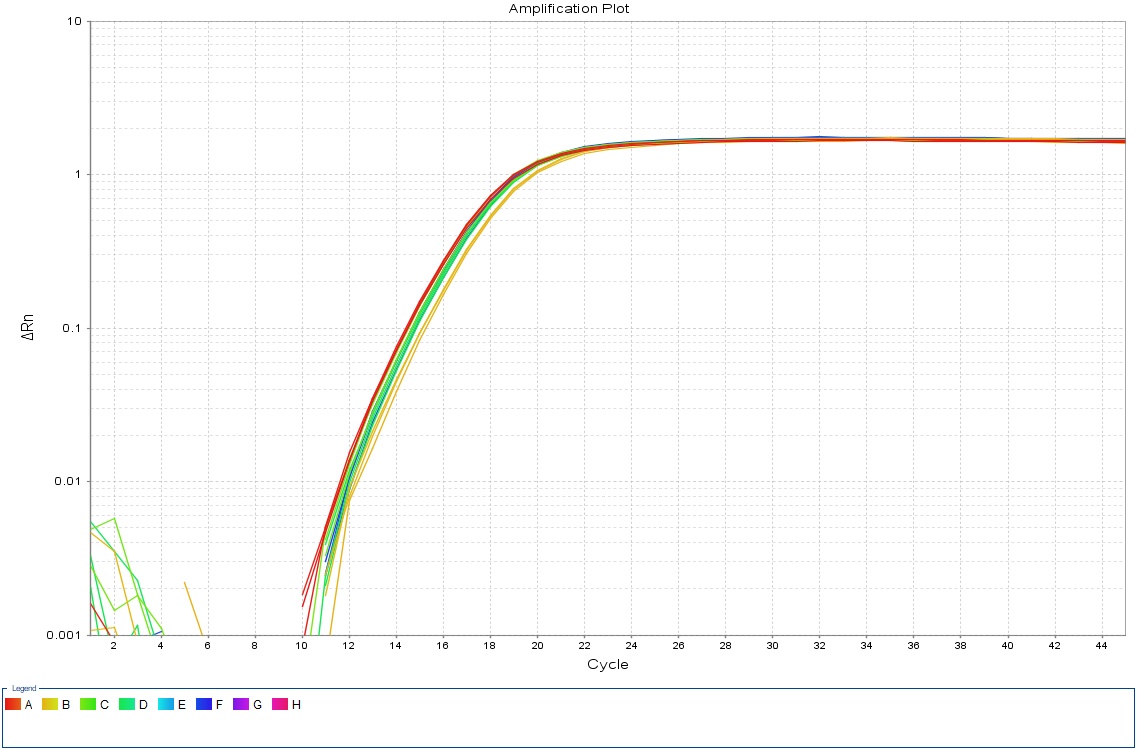

Supplement: Supplemental Information 7 [file peerj-13-19568-s007.zip › Figure 4B/Curve/GAPDH/Amplification Plot(LOG).jpg]

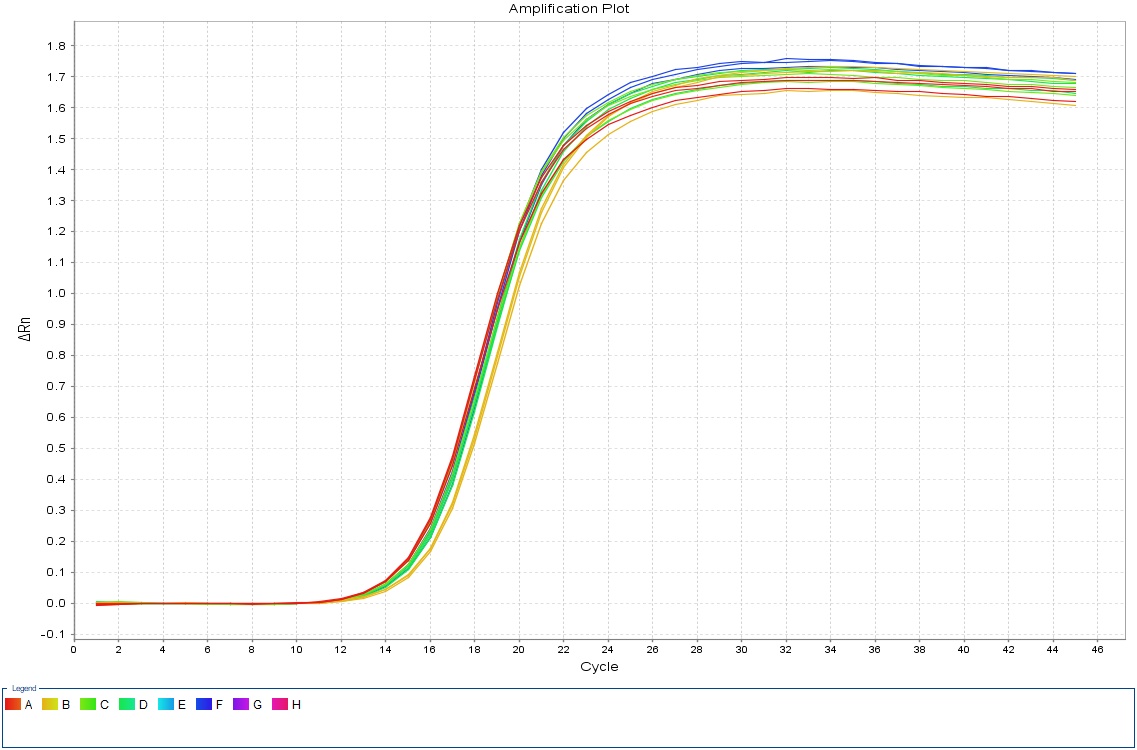

Supplement: Supplemental Information 7 [file peerj-13-19568-s007.zip › Figure 4B/Curve/GAPDH/Amplification Plot(linear).jpg]

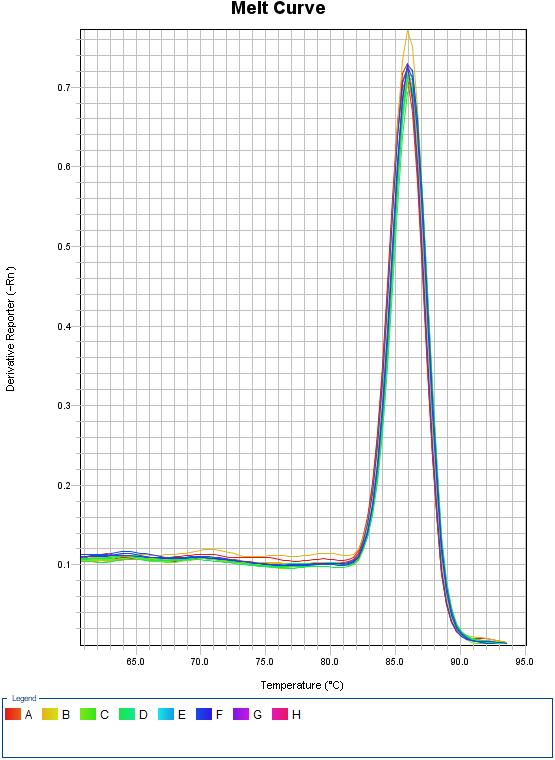

Supplement: Supplemental Information 7 [file peerj-13-19568-s007.zip › Figure 4B/Curve/GAPDH/Melt Curve.jpg]

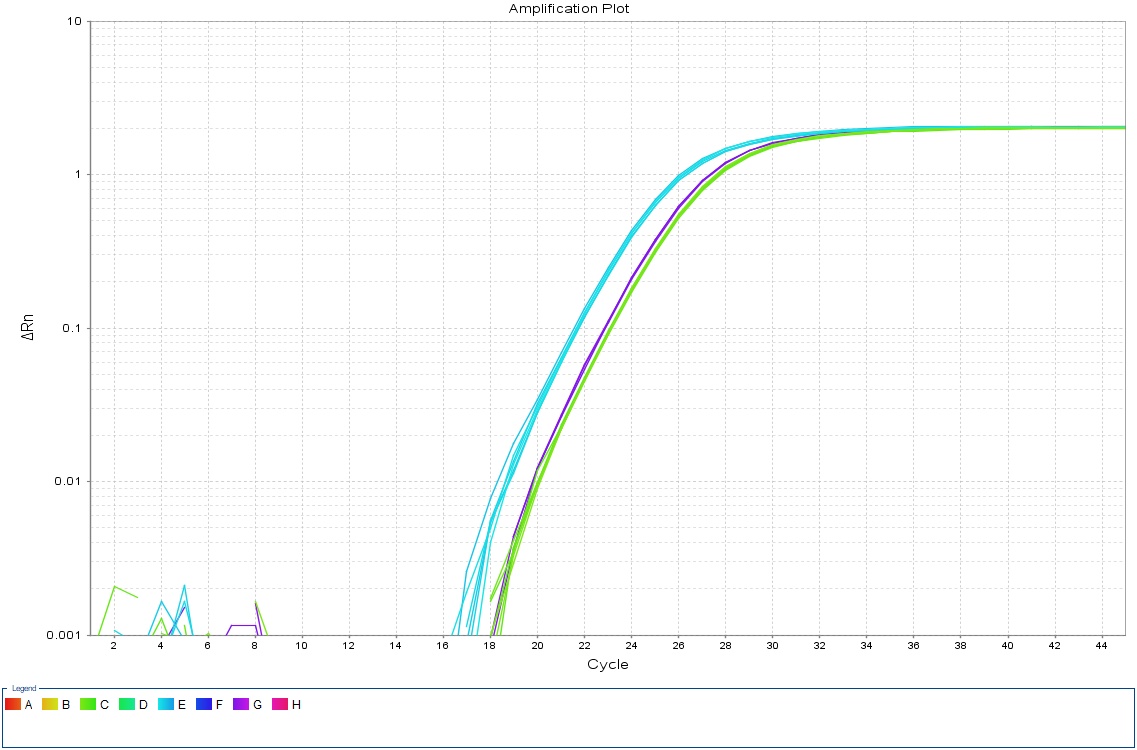

Supplement: Supplemental Information 7 [file peerj-13-19568-s007.zip › Figure 4B/Curve/VEGFA/Amplification Plot(LOG).jpg]

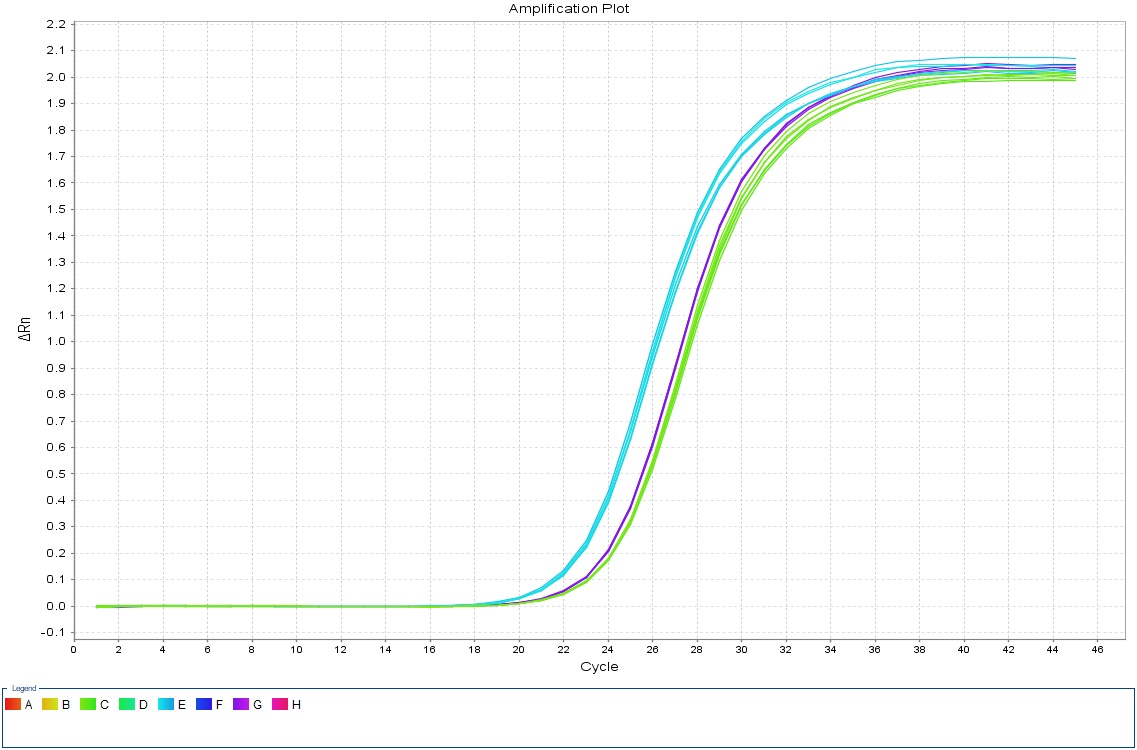

Supplement: Supplemental Information 7 [file peerj-13-19568-s007.zip › Figure 4B/Curve/VEGFA/Amplification Plot(linear).jpg]

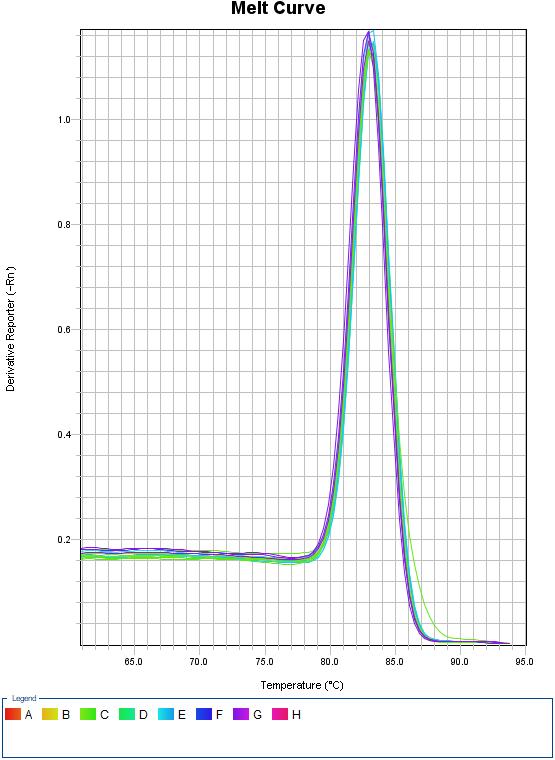

Supplement: Supplemental Information 7 [file peerj-13-19568-s007.zip › Figure 4B/Curve/VEGFA/Melt Curve.jpg]

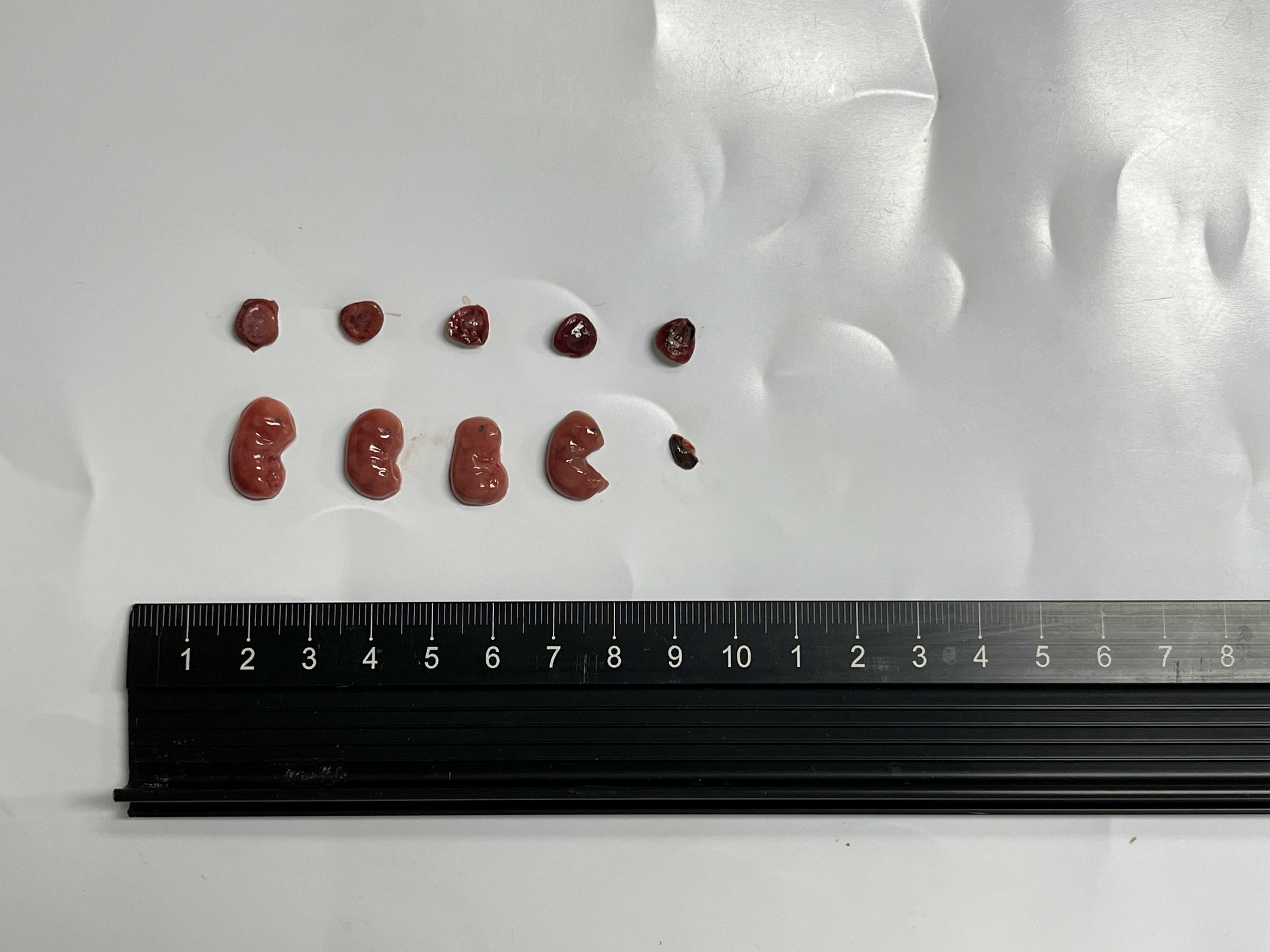

Supplement: Supplemental Information 8 [file peerj-13-19568-s008.zip › Figure 5A/abortion/AAV-NC -1.jpg]

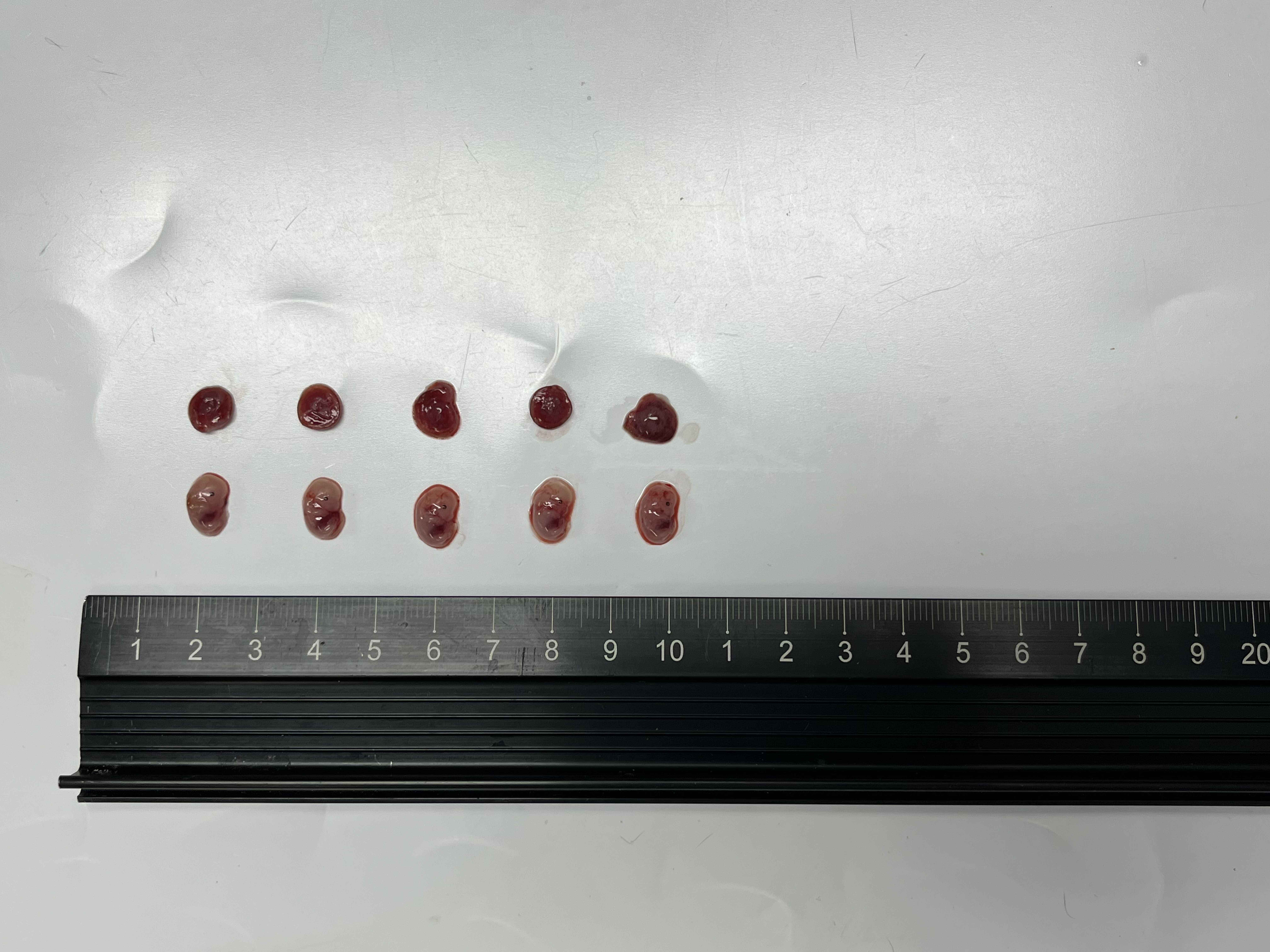

Supplement: Supplemental Information 8 [file peerj-13-19568-s008.zip › Figure 5A/abortion/AAV-NC -3.jpg]

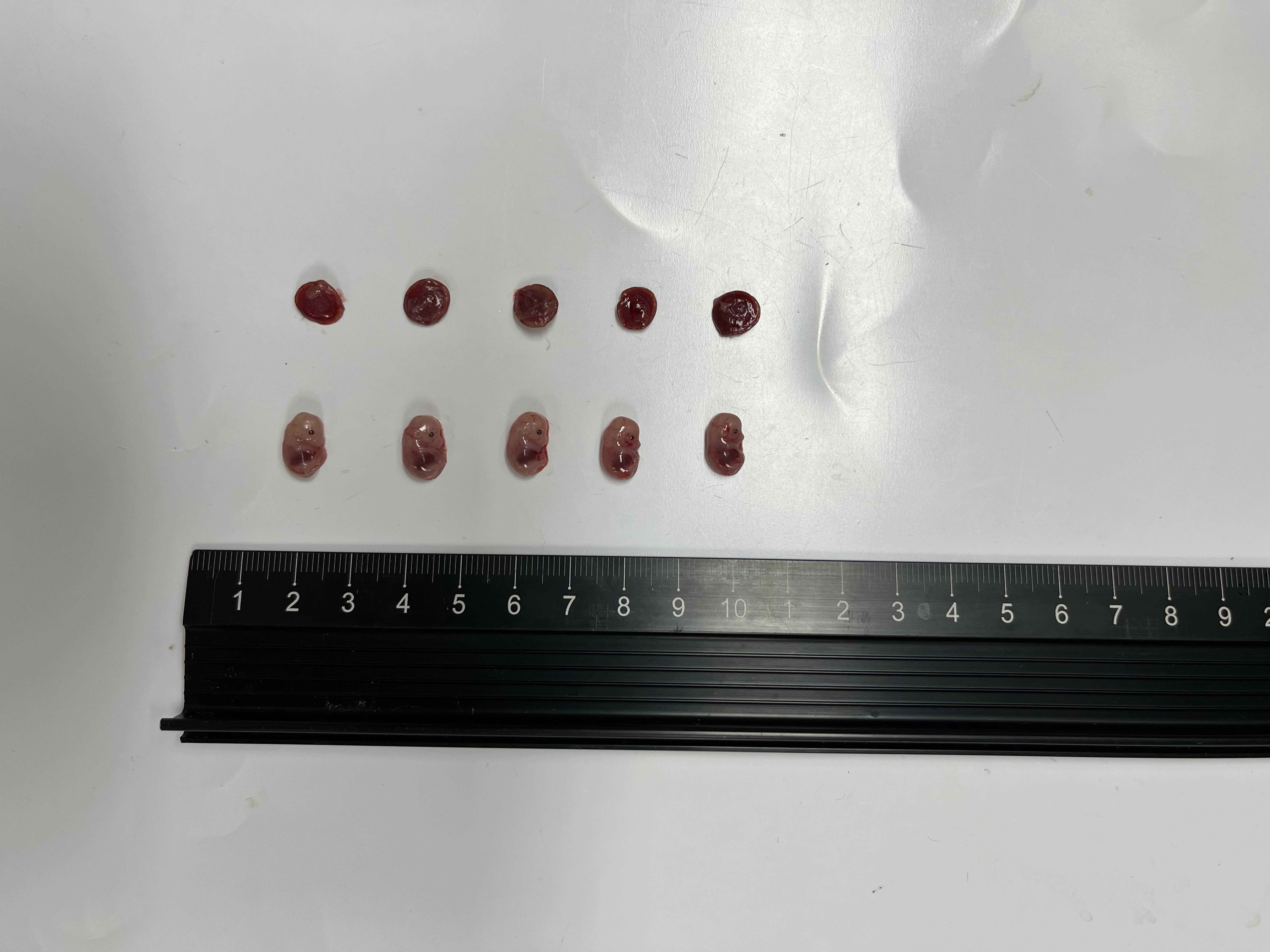

Supplement: Supplemental Information 8 [file peerj-13-19568-s008.zip › Figure 5A/abortion/AAV-NC -4.jpg]

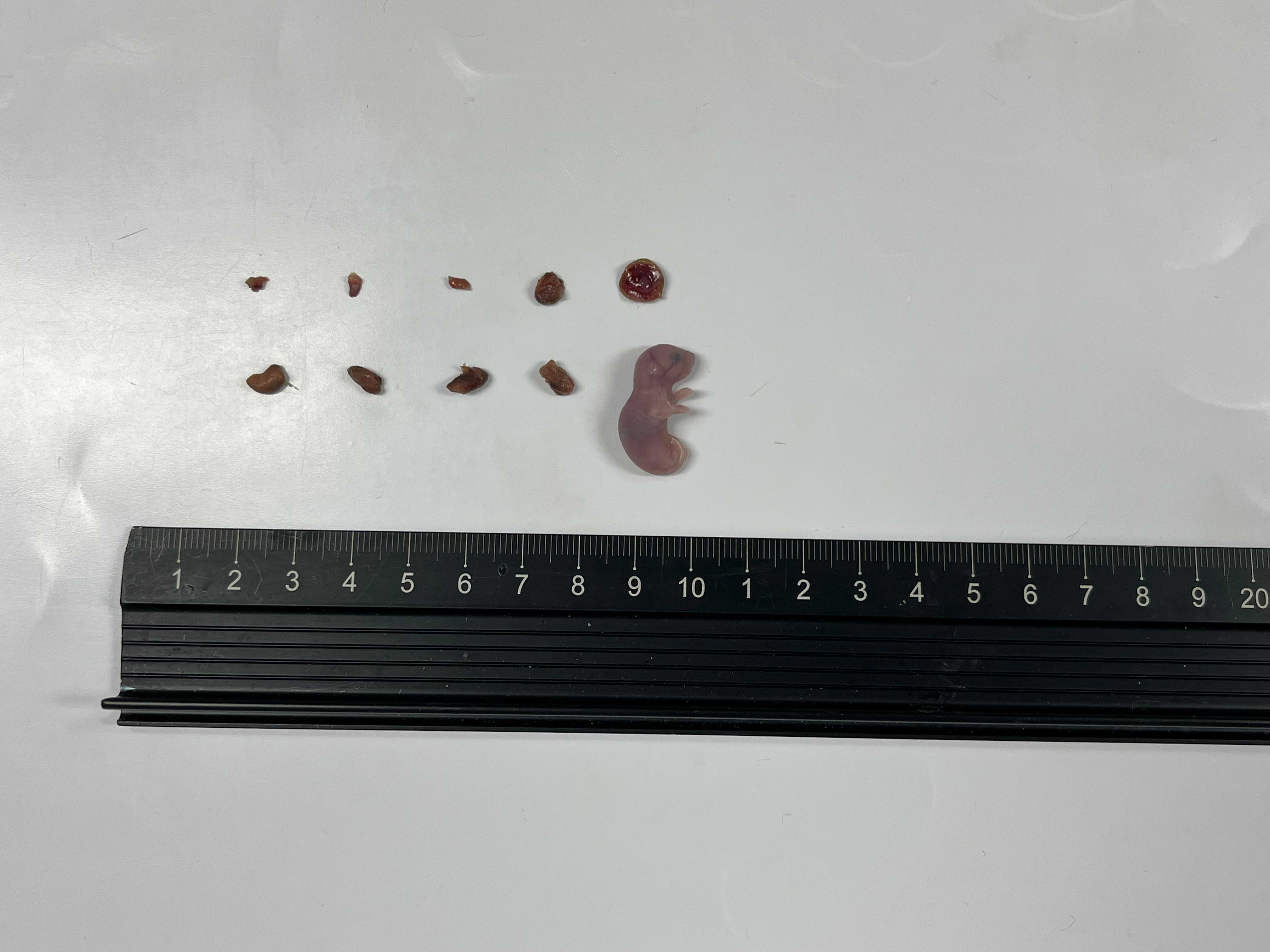

Supplement: Supplemental Information 8 [file peerj-13-19568-s008.zip › Figure 5A/abortion/AAV-NC -5.jpg]

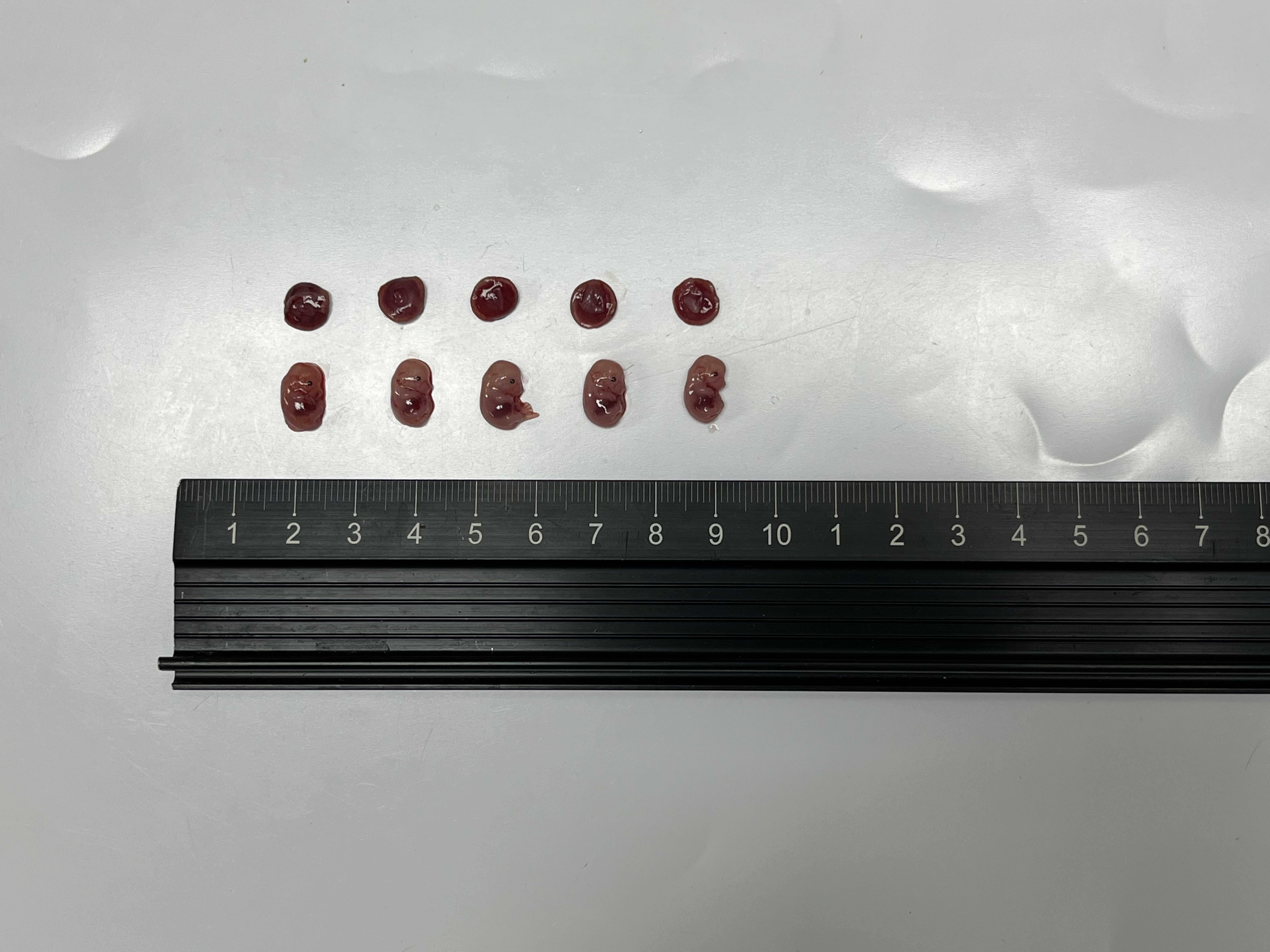

Supplement: Supplemental Information 8 [file peerj-13-19568-s008.zip › Figure 5A/abortion/AVV-NC-2.jpg]

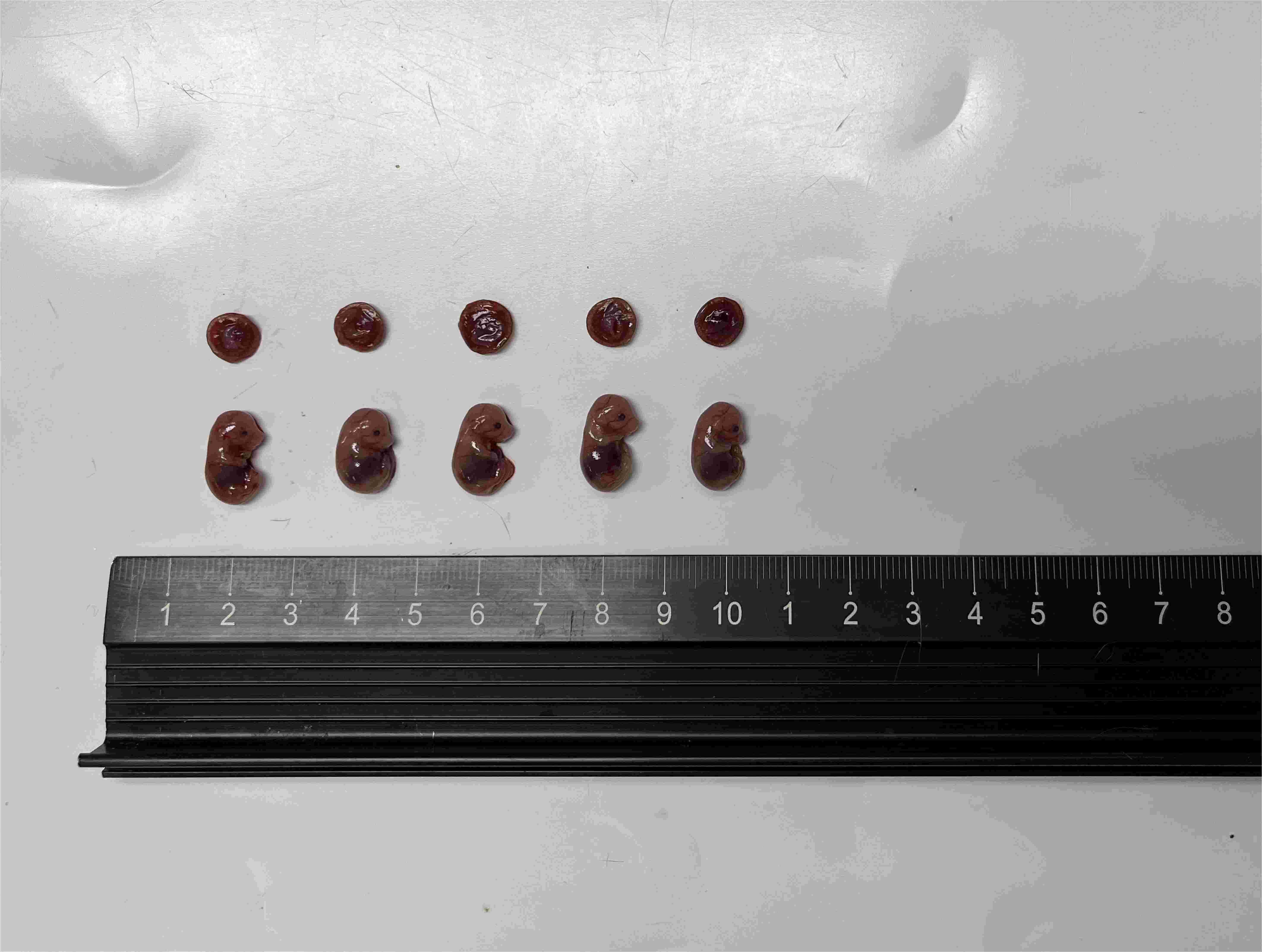

Supplement: Supplemental Information 8 [file peerj-13-19568-s008.zip › Figure 5A/abortion+shmiR-391-3p/AVV-mirna-1.jpg]

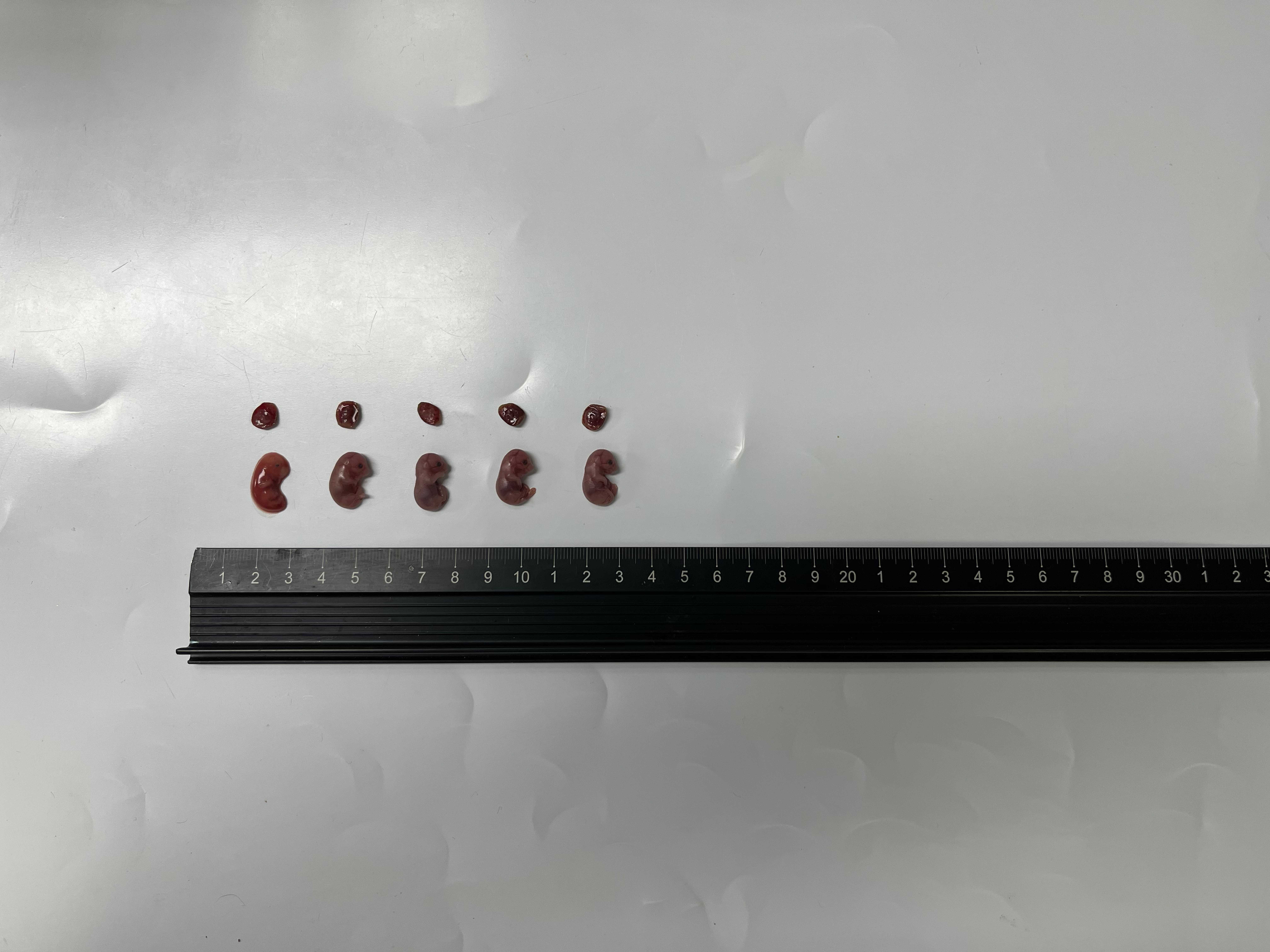

Supplement: Supplemental Information 8 [file peerj-13-19568-s008.zip › Figure 5A/abortion+shmiR-391-3p/AVV-mirna-2.jpg]

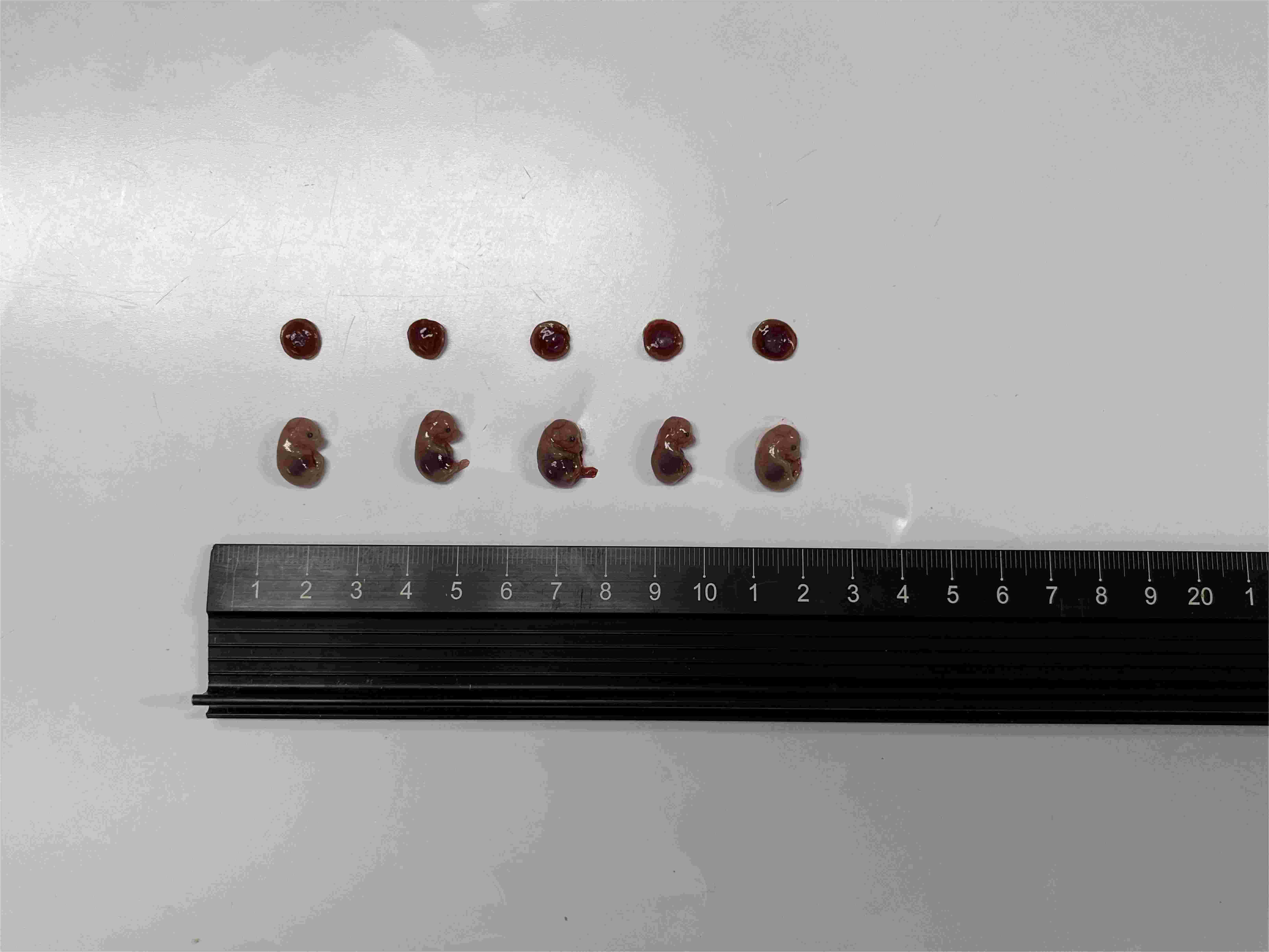

Supplement: Supplemental Information 8 [file peerj-13-19568-s008.zip › Figure 5A/abortion+shmiR-391-3p/AVV-mirna-3.jpg]

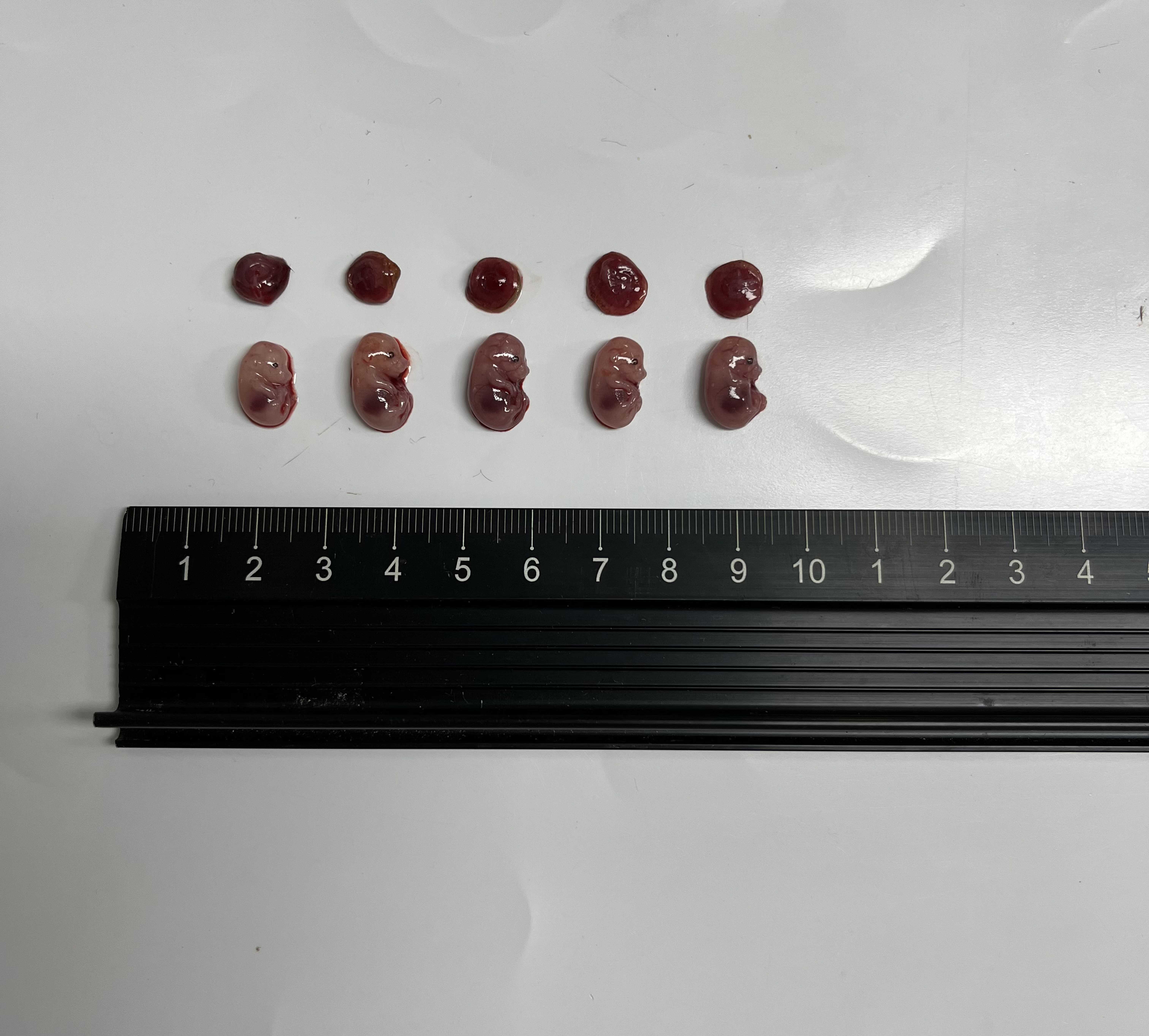

Supplement: Supplemental Information 8 [file peerj-13-19568-s008.zip › Figure 5A/abortion+shmiR-391-3p/AVV-mirna-4.jpg]

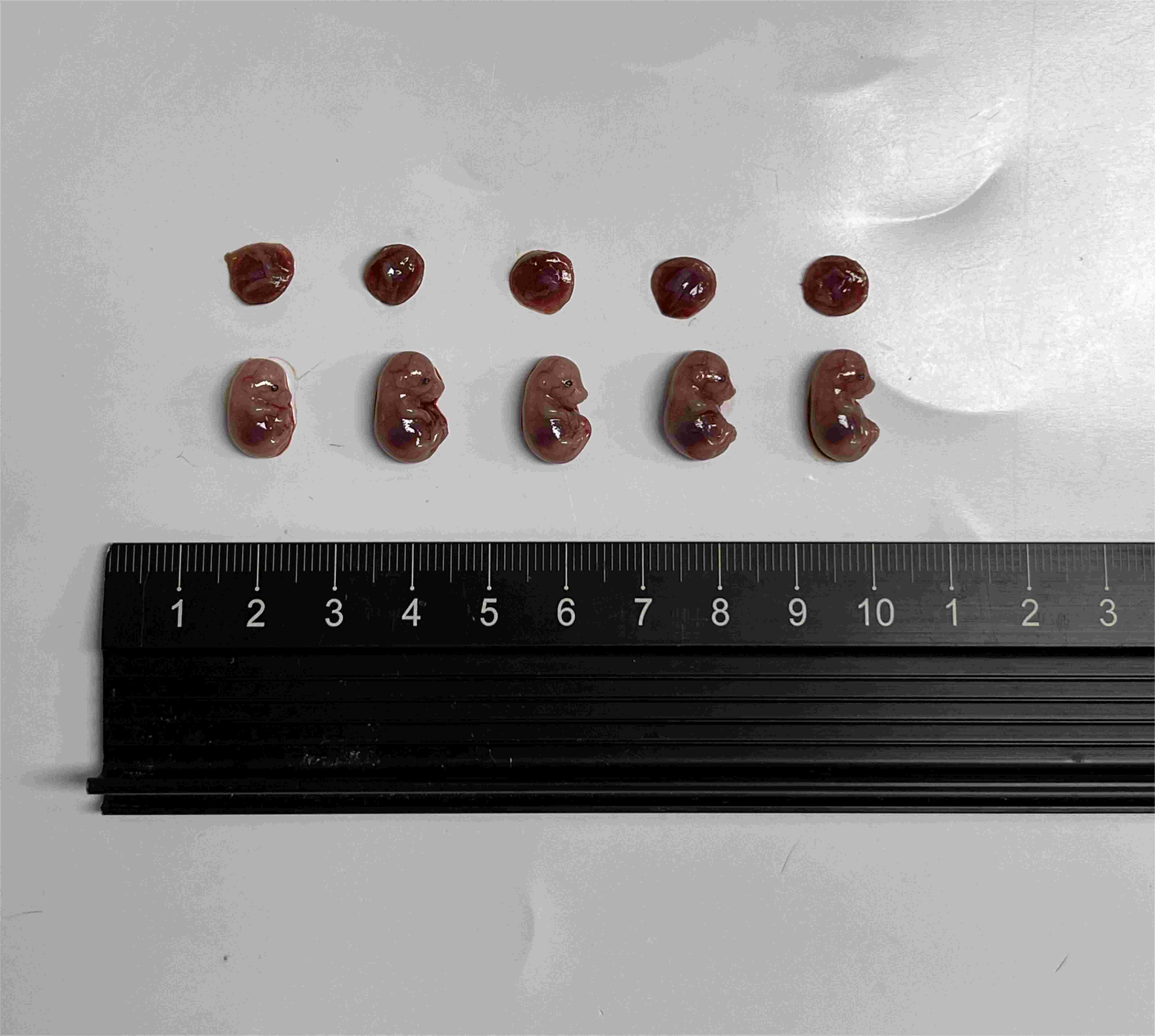

Supplement: Supplemental Information 8 [file peerj-13-19568-s008.zip › Figure 5A/abortion+shmiR-391-3p/AVV-mirna-5.jpg]

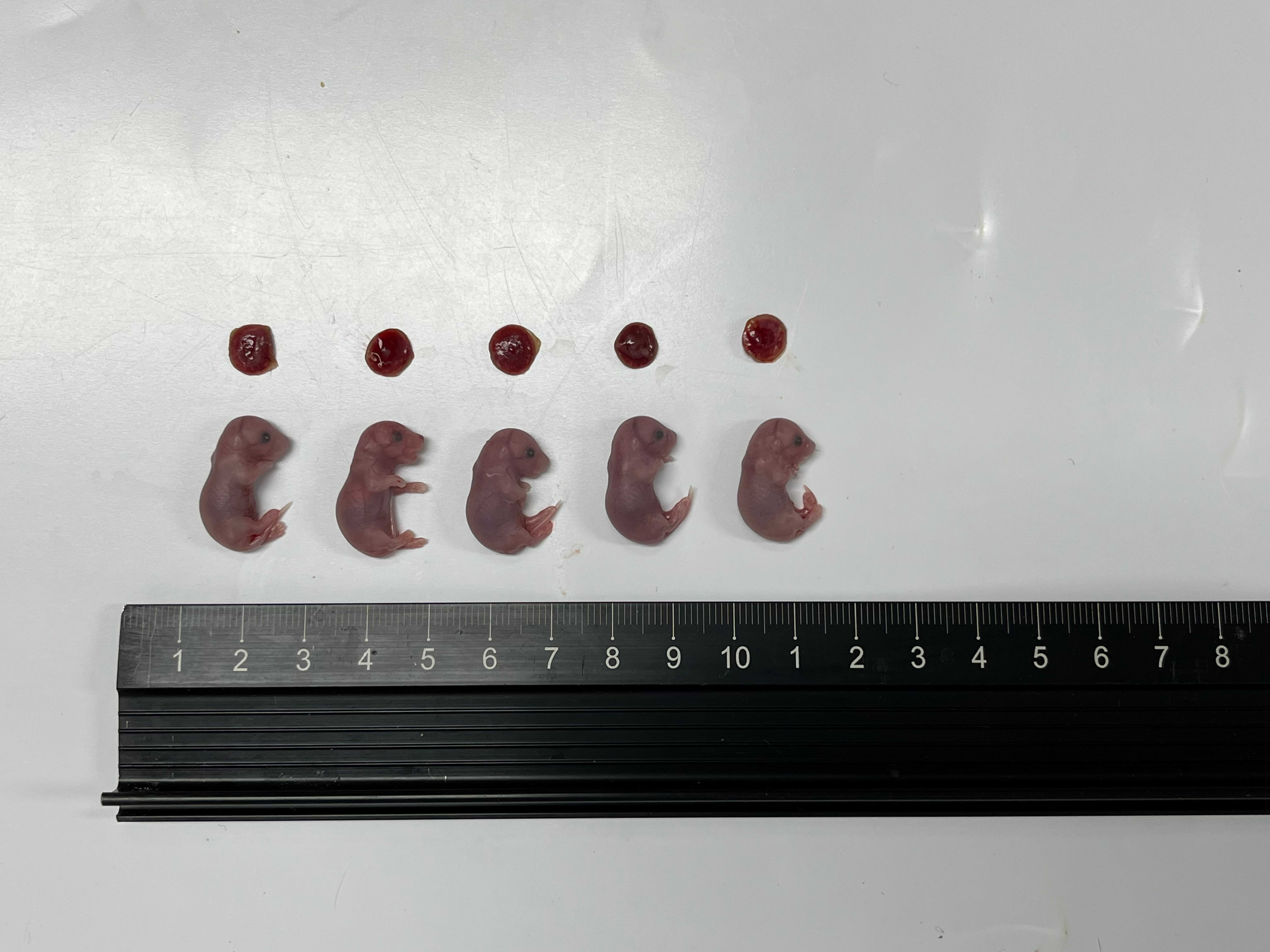

Supplement: Supplemental Information 8 [file peerj-13-19568-s008.zip › Figure 5A/control/con-1.jpg]

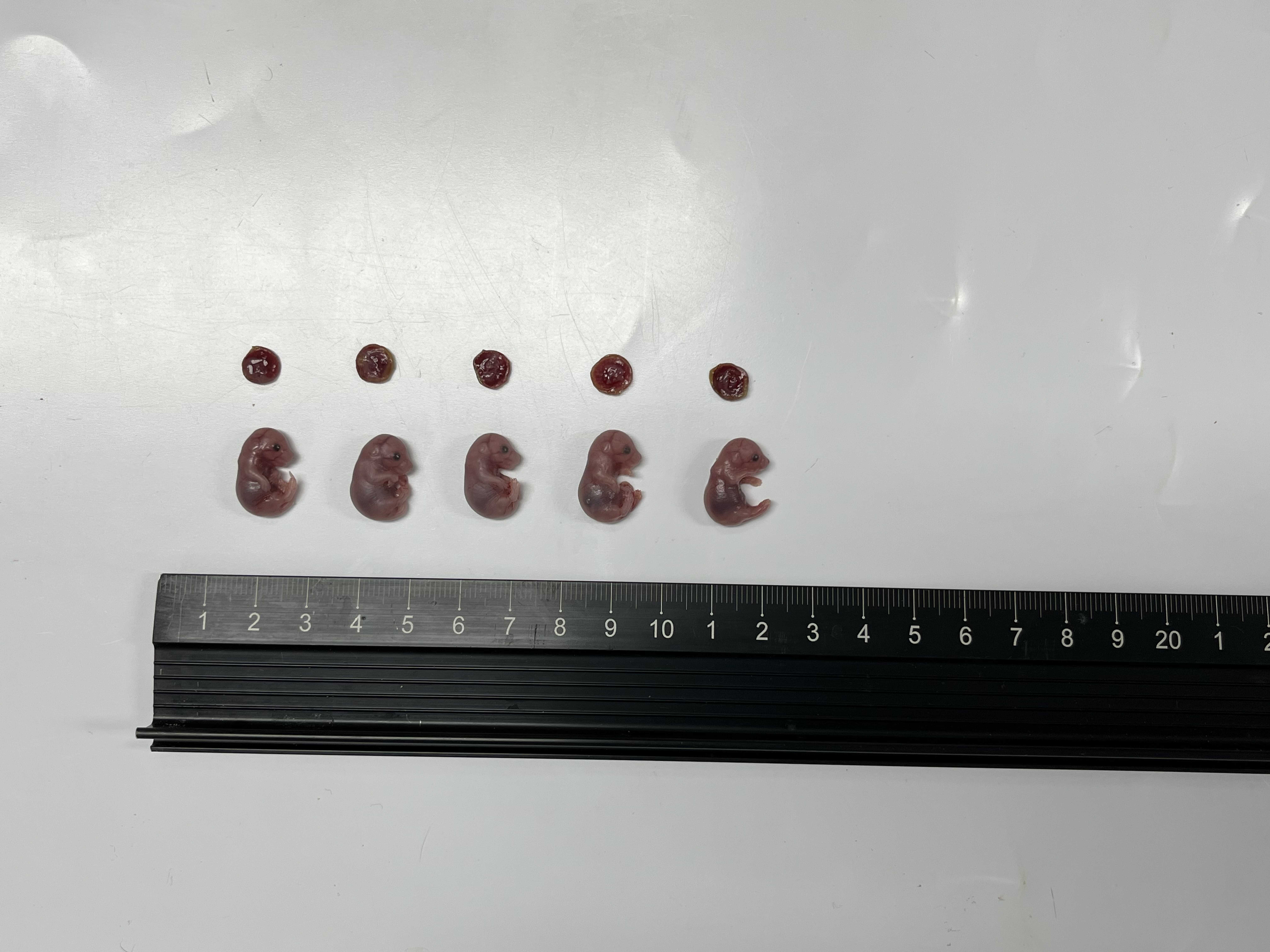

Supplement: Supplemental Information 8 [file peerj-13-19568-s008.zip › Figure 5A/control/con-2.jpg]

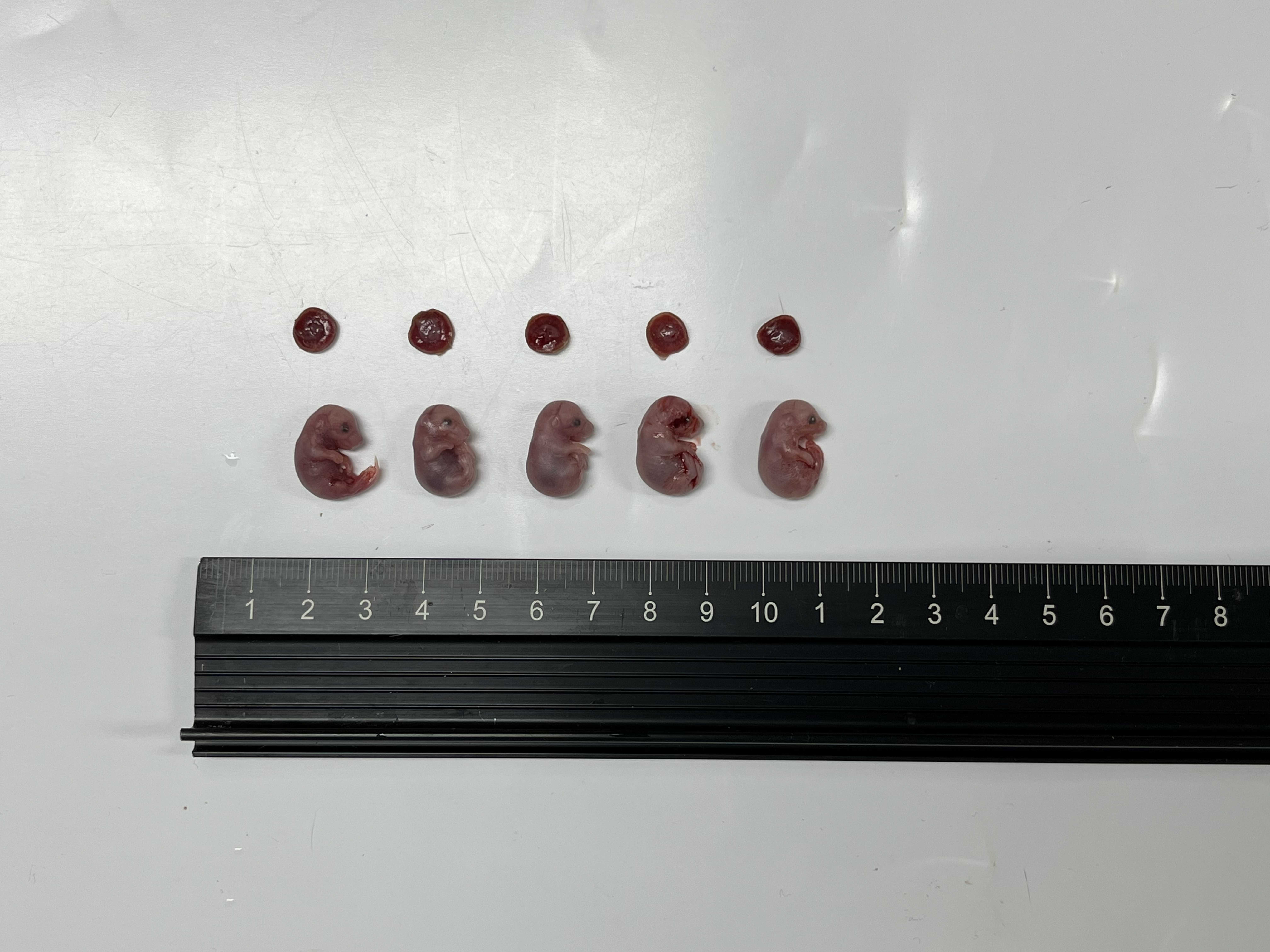

Supplement: Supplemental Information 8 [file peerj-13-19568-s008.zip › Figure 5A/control/con-3.jpg]

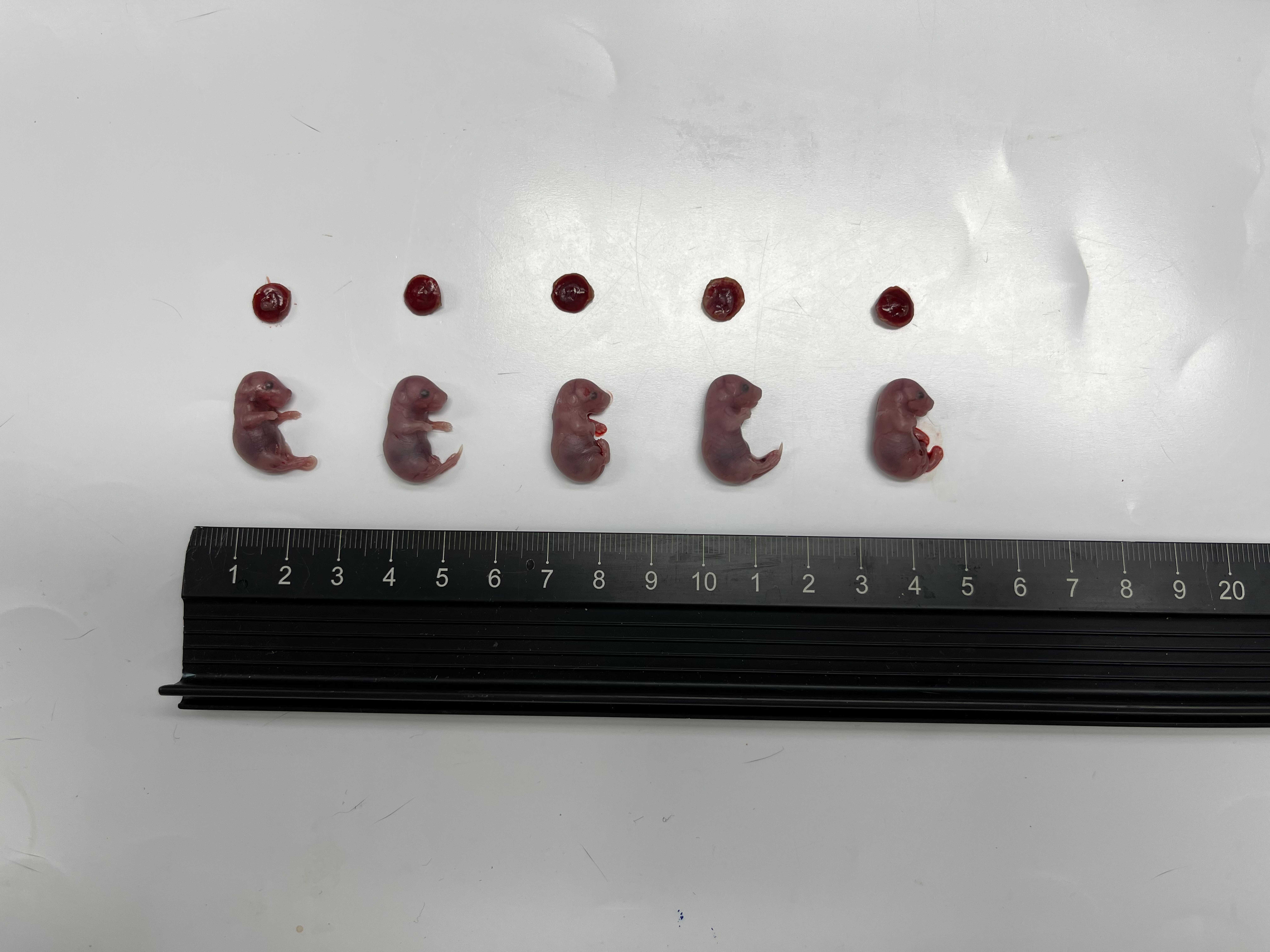

Supplement: Supplemental Information 8 [file peerj-13-19568-s008.zip › Figure 5A/control/con-4.jpg]

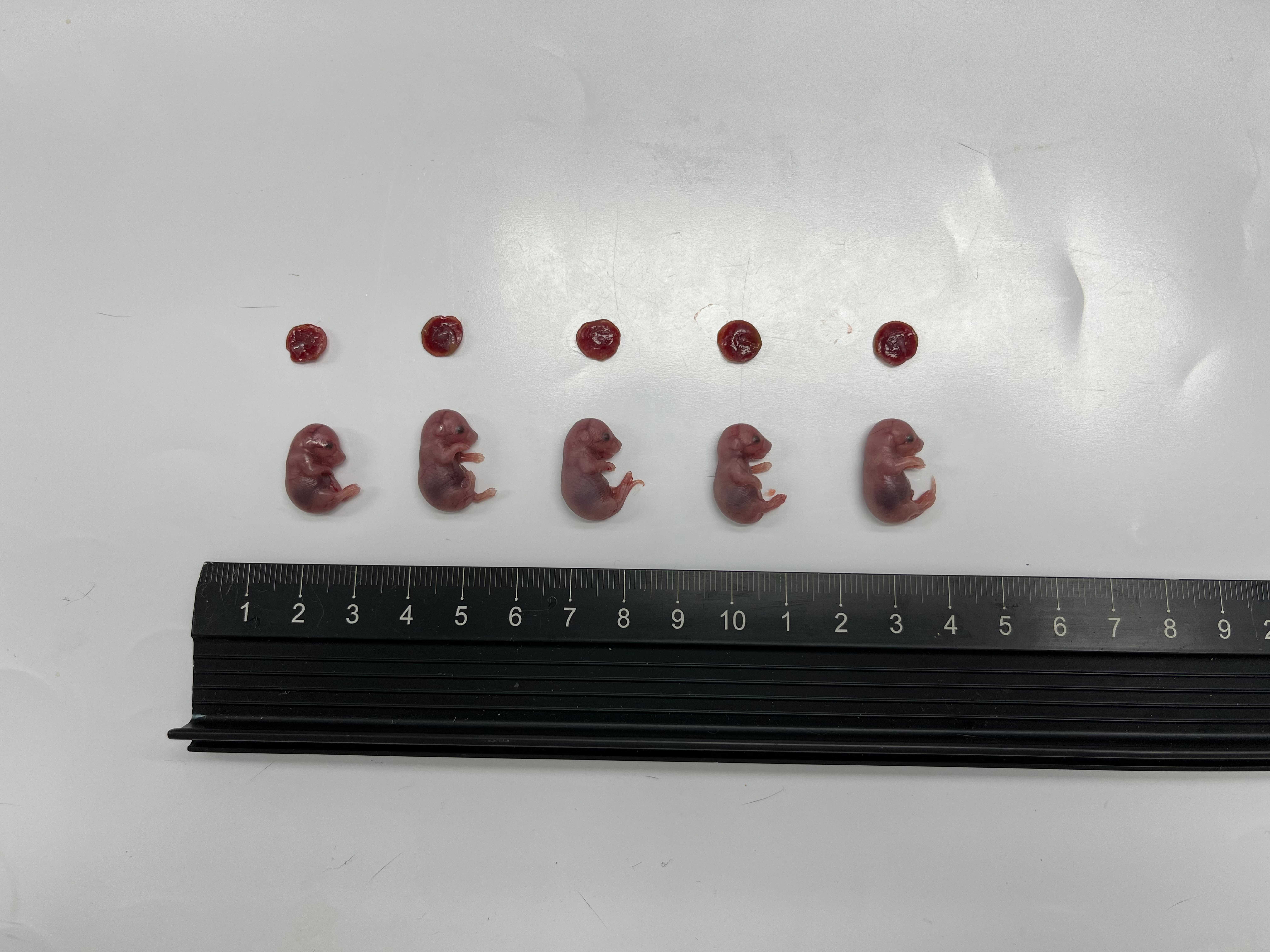

Supplement: Supplemental Information 8 [file peerj-13-19568-s008.zip › Figure 5A/control/con-5.jpg]

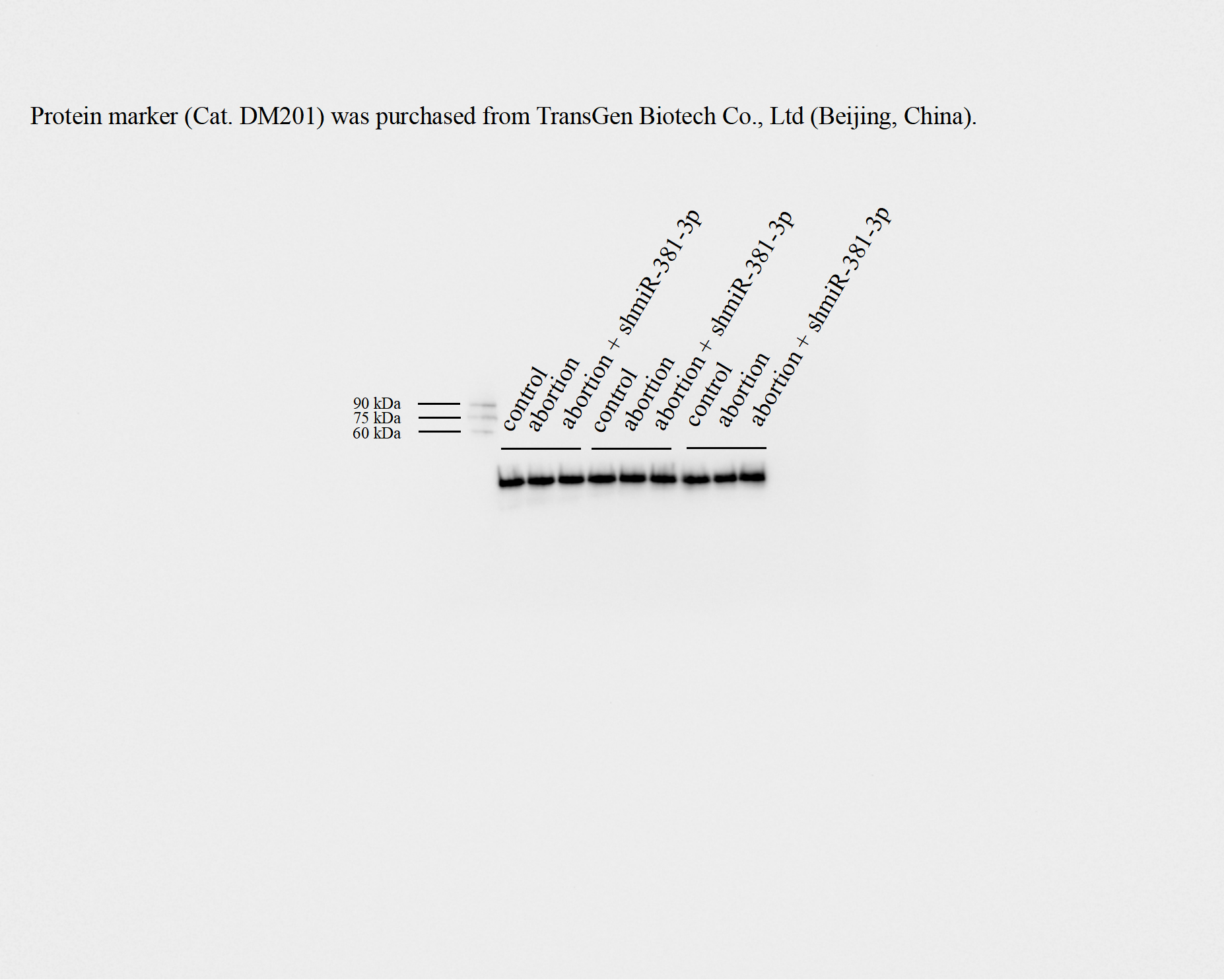

Supplement: Supplemental Information 10 [file peerj-13-19568-s010.zip › Figure 6B/Description.png]

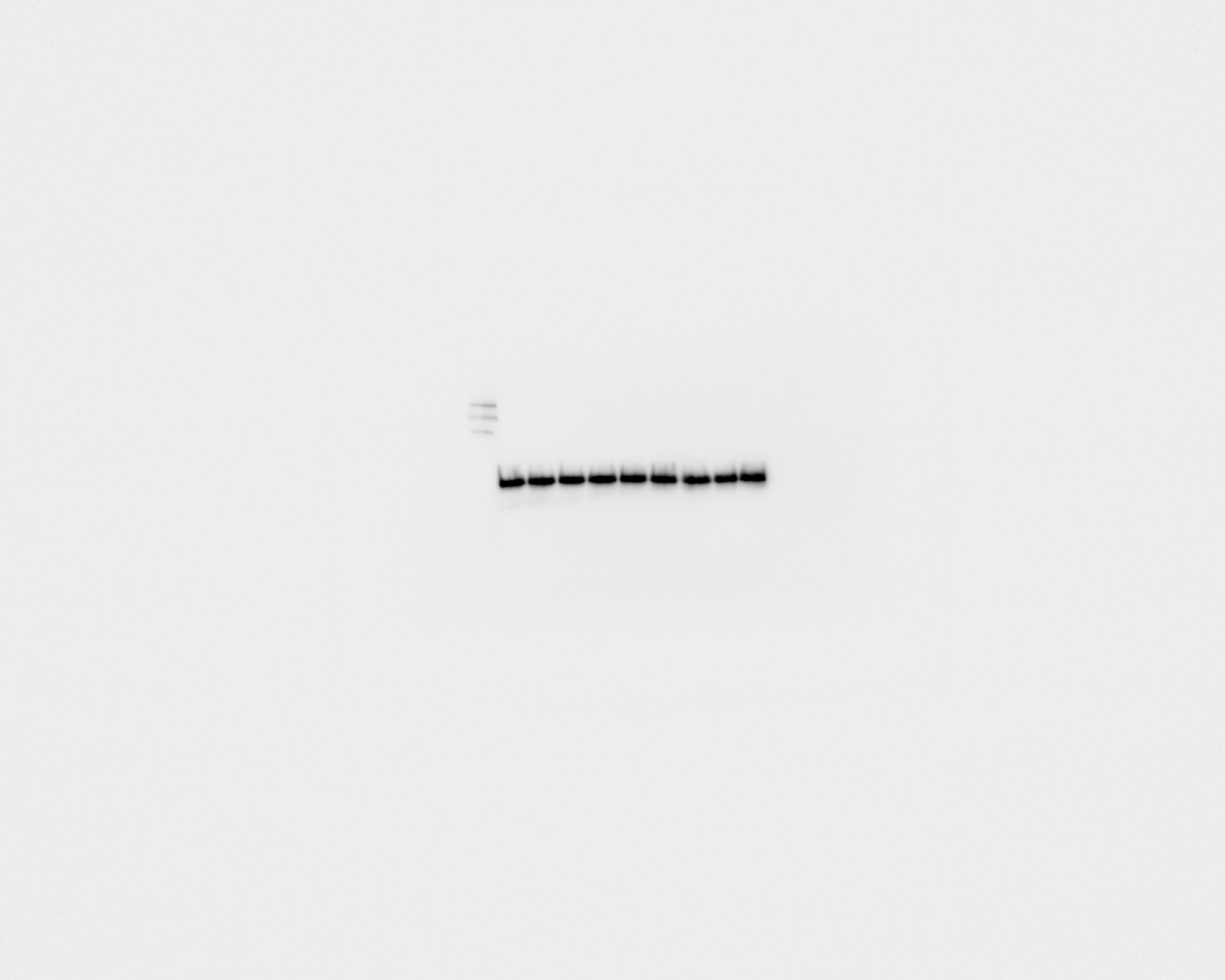

Supplement: Supplemental Information 10 [file peerj-13-19568-s010.zip › Figure 6B/GAPDH.tif]

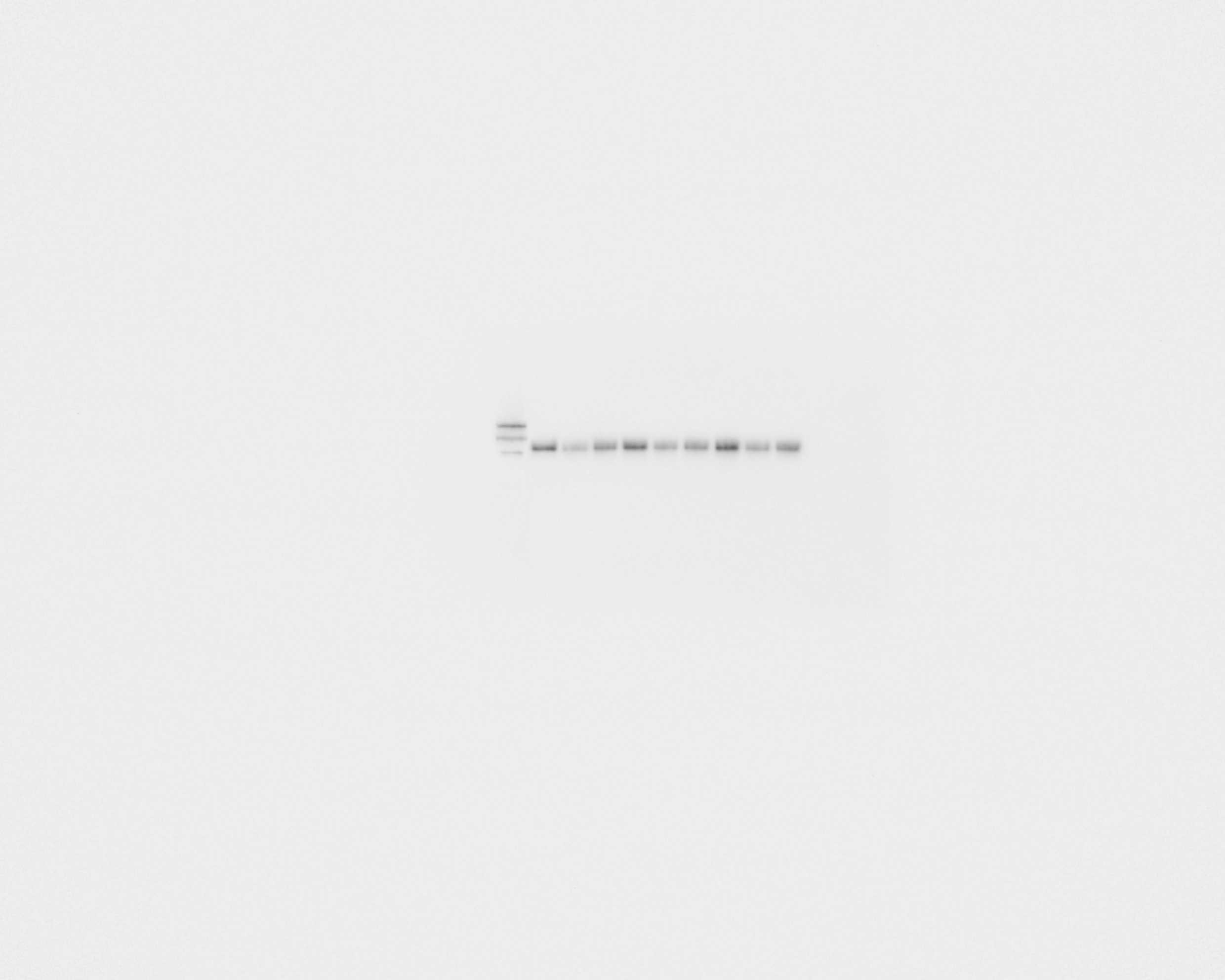

Supplement: Supplemental Information 10 [file peerj-13-19568-s010.zip › Figure 6B/P-P65.tif]

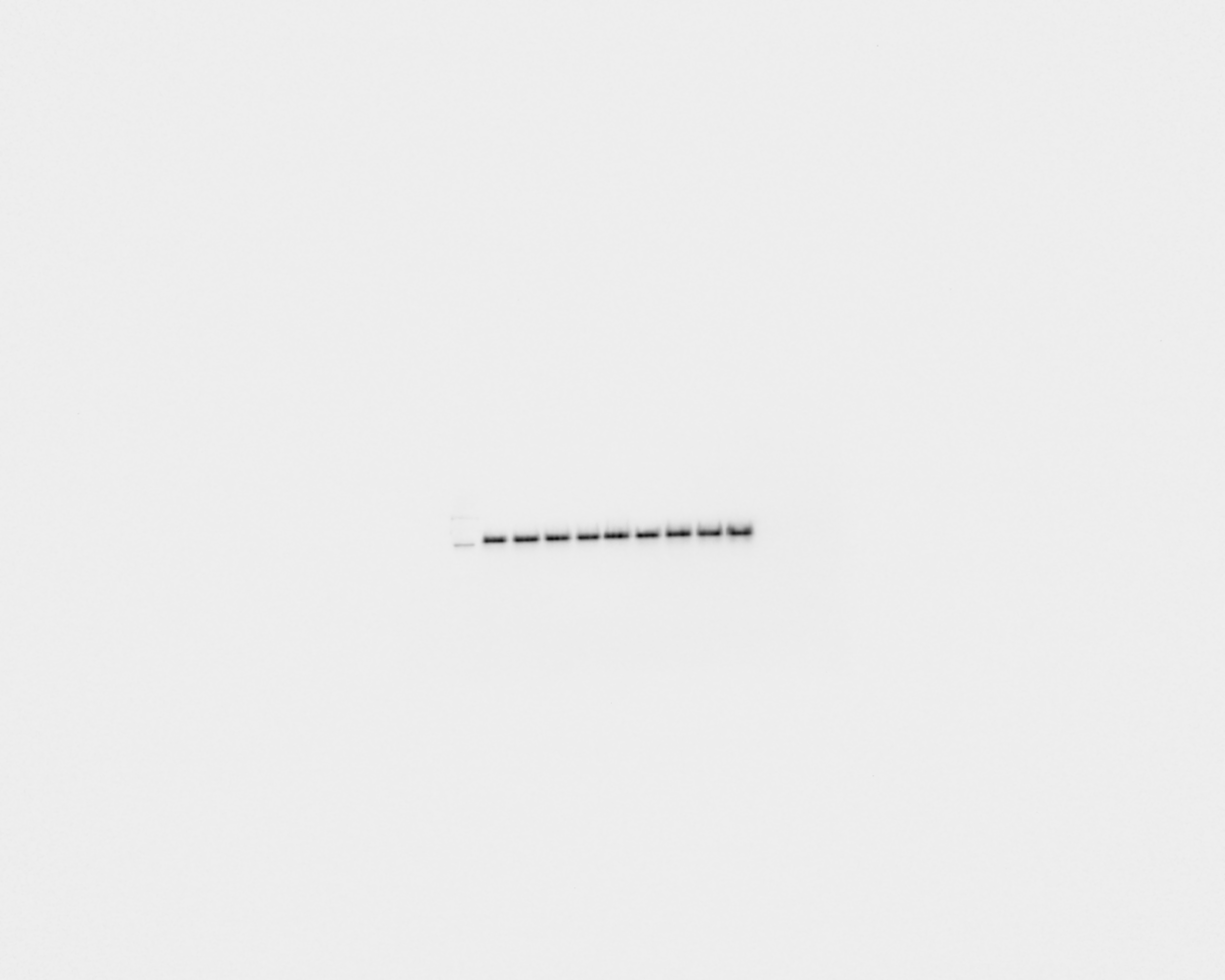

Supplement: Supplemental Information 10 [file peerj-13-19568-s010.zip › Figure 6B/P65.tif]

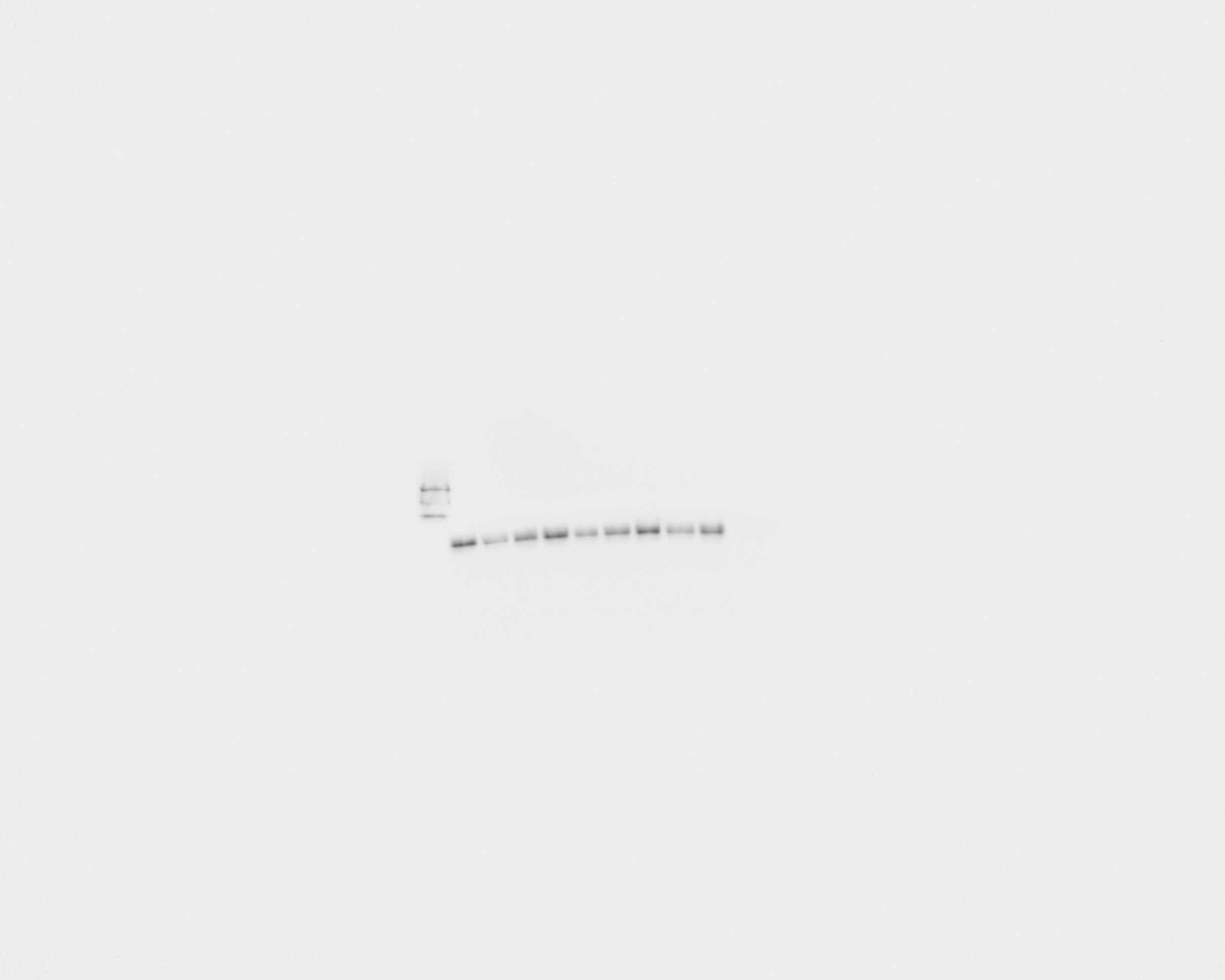

Supplement: Supplemental Information 10 [file peerj-13-19568-s010.zip › Figure 6B/VEGF.tif]
