# Supplementary material for: miR-381-3p contribution in mouse spontaneous abortion via targeting VEGFA
Source: PeerJ. 2025 Jun 24;13:e19568. doi: 10.7717/peerj.19568 (PMC12204090; doi:10.7717/peerj.19568)
Supplement: Supplemental Information 13 [file peerj-13-19568-s013.docx]

**Table S1.** Differential miRNA in the placentas of aborted mice based on high-throughput sequencing

| Name | log2(abortion/control) | 1. value | Regulation |
| --- | --- | --- | --- |
| mmu-miR-12182-3p | 2.033091938 | 0.000399745 | up |
| mmu-miR-12184-5p | 2.228061008 | 0.039760678 | up |
| mmu-miR-125b-1-3p | 1.332332132 | 0.008287529 | up |
| mmu-miR-134-3p | 1.035112605 | 0.025219776 | up |
| mmu-miR-136-5p | 1.229674589 | 0.002942896 | up |
| mmu-miR-145a-5p | 1.125435786 | 0.018437961 | up |
| mmu-miR-181a-2-3p | 1.116836336 | 0.039048003 | up |
| mmu-miR-184-3p | 1.310448034 | 0.005428622 | up |
| mmu-miR-192-3p | 1.433741021 | 0.016379073 | up |
| mmu-miR-192-5p | 2.732874009 | 0.000349525 | up |
| mmu-miR-194-1-3p | 2.481666432 | 1.05221E-05 | up |
| mmu-miR-194-2-3p | 1.803981696 | 0.02630282 | up |
| mmu-miR-194-5p | 1.916463611 | 0.003946105 | up |
| mmu-miR-200a-3p | 1.703147791 | 0.004821426 | up |
| mmu-miR-200a-5p | 2.547090493 | 8.27064E-05 | up |
| mmu-miR-200b-3p | 2.000628323 | 0.000512064 | up |
| mmu-miR-200b-5p | 1.95164506 | 0.000947004 | up |
| mmu-miR-204-5p | 1.218936804 | 0.009727781 | up |
| mmu-miR-215-3p | 2.128133719 | 1.44113E-05 | up |
| mmu-miR-215-5p | 2.581435094 | 7.10838E-06 | up |
| mmu-miR-302b-3p | 1.212796383 | 0.016337454 | up |
| mmu-miR-31-5p | 1.030092173 | 0.008408483 | up |
| mmu-miR-325-3p | 4.071905039 | 0.047494831 | up |
| mmu-miR-329-5p | 1.196008791 | 0.004088894 | up |
| mmu-miR-340-3p | 1.777328466 | 0.000252306 | up |
| mmu-miR-340-5p | 1.13023681 | 0.009208549 | up |
| mmu-miR-344-3p | 1.267571704 | 0.03797398 | up |
| mmu-miR-369-5p | 1.261212344 | 0.009229584 | up |
| mmu-miR-370-3p | 1.528452387 | 0.006376228 | up |
| mmu-miR-376b-5p | 1.189762637 | 0.01287094 | up |
| mmu-miR-376c-5p | 1.458347799 | 0.005553238 | up |
| mmu-miR-381-3p | 1.13533467 | 0.019245793 | up |
| mmu-miR-383-5p | 3.594163338 | 2.22097E-05 | up |
| mmu-miR-429-3p | 1.747805397 | 0.006441433 | up |
| mmu-miR-434-5p | 1.574645888 | 0.002457638 | up |
| mmu-miR-455-3p | 1.765832892 | 0.002662414 | up |
| mmu-miR-455-5p | 2.136002464 | 0.000373192 | up |
| mmu-miR-463-5p | 2.32651766 | 0.001002874 | up |
| mmu-miR-465a-3p | 2.587385149 | 0.000739588 | up |
| mmu-miR-465a-5p | 3.01948012 | 6.24774E-05 | up |
| mmu-miR-465b-3p | 2.587376657 | 0.00073392 | up |
| mmu-miR-465b-5p | 2.62983113 | 0.00043296 | up |
| mmu-miR-465c-3p | 2.604982161 | 0.000839652 | up |
| mmu-miR-465c-5p | 2.730999094 | 0.000148929 | up |
| mmu-miR-465d-3p | 2.010568132 | 0.023983078 | up |
| mmu-miR-470-5p | 2.526508192 | 0.000343181 | up |
| mmu-miR-471-3p | 3.134938006 | 4.16647E-06 | up |
| mmu-miR-471-5p | 2.747578597 | 0.000162398 | up |
| mmu-miR-485-5p | 1.194578456 | 0.004004993 | up |
| mmu-miR-541-5p | 1.03485379 | 0.013003299 | up |
| mmu-miR-543-3p | 1.224256605 | 0.007975652 | up |
| mmu-miR-615-3p | 1.88232404 | 0.003352695 | up |
| mmu-miR-615-5p | 2.525235028 | 0.002461765 | up |
| mmu-miR-668-3p | 1.214356434 | 0.008363008 | up |
| mmu-miR-668-5p | 4.474397081 | 0.014696729 | up |
| mmu-miR-6715-5p | 2.852885005 | 0.003097054 | up |
| mmu-miR-708-3p | 1.761214385 | 0.000372312 | up |
| mmu-miR-708-5p | 1.218660396 | 0.03954953 | up |
| mmu-miR-741-3p | 2.775002646 | 8.82604E-05 | up |
| mmu-miR-741-5p | 3.613665271 | 0.000128919 | up |
| mmu-miR-742-3p | 5.01714474 | 0.001679999 | up |
| mmu-miR-742-5p | 2.882079962 | 0.007744819 | up |
| mmu-miR-743a-3p | 2.54296961 | 0.000923349 | up |
| mmu-miR-743a-5p | 3.02642188 | 0.000680941 | up |
| mmu-miR-743b-3p | 2.78843059 | 8.55707E-05 | up |
| mmu-miR-743b-5p | 3.262173125 | 5.70812E-05 | up |
| mmu-miR-802-5p | 4.285092801 | 0.022781244 | up |
| mmu-miR-871-3p | 3.271331446 | 3.75746E-05 | up |
| mmu-miR-871-5p | 2.406463303 | 0.001474427 | up |
| mmu-miR-878-3p | 1.817315848 | 0.014609603 | up |
| mmu-miR-878-5p | 2.90676579 | 6.05969E-05 | up |
| mmu-miR-880-3p | 2.403508292 | 0.000868012 | up |
| mmu-miR-881-3p | 3.257532041 | 3.02249E-05 | up |
| mmu-miR-883a-3p | 2.374371236 | 0.00198504 | up |
| mmu-miR-883b-5p | 4.071895507 | 0.04738399 | up |
| mmu-miR-92b-5p | 1.331657379 | 0.014498786 | up |
| 5_14543-5p(cel-miR-354-3p) | 2.51010399 | 0.014202193 | up |
| X_47486-5p(rno-miR-465-5p) | 3.223225474 | 2.26133E-05 | up |
| X_47469-5p | 3.112422912 | 2.14005E-05 | up |
| X_47464-5p | 3.812743099 | 6.50754E-06 | up |
| X_47481-3p | 2.820407743 | 0.000345054 | up |
| X_47474-5p | 2.619194404 | 0.000602285 | up |
| X_47483-5p(rno-miR-465-5p) | 2.840158483 | 0.000208302 | up |
| 12_32402-3p | 1.139717103 | 0.013531195 | up |
| 5_12772-5p(cel-miR-87-5p) | 1.926115471 | 0.036225176 | up |
| X_47486-3p | 2.802292858 | 0.000298835 | up |
| X_47475-5p(mdo-miR-7386h-5p) | 2.739022847 | 0.000162958 | up |
| X_47464-3p(cel-miR-1018) | 2.980419892 | 4.20376E-05 | up |
| 8_21411-3p(bta-miR-2439-5p) | 1.892395318 | 0.002001449 | up |
| 5_13647-5p(hsa-miR-574-5p) | 1.111559994 | 0.03974332 | up |
| X_47468-3p(rno-miR-880-3p) | 2.61910081 | 0.000341324 | up |
| 8_22469-5p(rno-miR-1199-5p) | 1.568569685 | 0.025672875 | up |
| X_47483-3p | 2.802285375 | 0.00029657 | up |
| X_47477-5p(rno-miR-465-5p) | 2.936817084 | 6.62076E-05 | up |
| X_47477-3p | 2.82040146 | 0.000342851 | up |
| 4_9530-5p(hsa-miR-518c-5p) | 2.867401096 | 0.008224729 | up |
| X_47474-3p(rno-miR-871-3p) | 3.460850275 | 1.73121E-05 | up |
| 11_30660-3p(cfa-miR-8908a-3p) | 2.274510563 | 6.26976E-05 | up |
| 5_12771-3p(cel-miR-87-5p) | 1.925794846 | 0.035463468 | up |
| X_47469-3p | 2.047531428 | 0.004785961 | up |
| X_47481-5p(rno-miR-465-5p) | 2.9368203 | 6.69559E-05 | up |
| X_46563-5p | 1.021837417 | 0.026930628 | up |
| X_47472-5p | 3.80839453 | 0.001535228 | up |
| X_47479-3p | 2.802287075 | 0.000297083 | up |
| 4_9451-3p(bta-miR-2471-5p) | 1.473317729 | 0.028165058 | up |
| 1_3102-3p(cte-miR-2708) | 1.732552529 | 0.043286399 | up |
| X_47479-5p(rno-miR-465-5p) | 2.840159825 | 0.000209328 | up |
| 7_20028-3p(dqu-miR-263b-3p) | 1.548524933 | 0.023267657 | up |
| 6_15945-3p(cin-miR-4001e-3p) | 1.857036379 | 0.029433422 | up |
| 16_40271-3p(tca-miR-263a-3p) | 2.049298042 | 0.021381208 | up |
| 18_43880-5p(hsa-miR-149-5p) | 2.621058977 | 0.008319487 | up |
| X_47472-3p(hsa-miR-892a) | 3.440586377 | 1.64013E-05 | up |
| 8_21987-5p(cfa-miR-8850) | 1.19845364 | 0.007687354 | up |
| mmu-miR-124-5p | -1.377276361 | 0.011847566 | down |
| mmu-miR-135b-5p | -1.240803439 | 0.048918805 | down |
| mmu-miR-21b | -1.577130691 | 0.022068142 | down |
| mmu-miR-21c | -1.483522882 | 0.019285615 | down |
| mmu-miR-345-5p | -1.085923317 | 0.023397198 | down |
| mmu-miR-3547-3p | -2.293314104 | 0.01786563 | down |
| mmu-miR-3572-5p | -2.92530737 | 0.030274476 | down |
| mmu-miR-5121 | -1.940372581 | 0.002361879 | down |
| mmu-miR-6919-5p | -4.929394828 | 0.006875787 | down |
| mmu-miR-6938-3p | -4.094480723 | 0.040289915 | down |
| mmu-miR-6948-5p | -1.638894206 | 0.038015314 | down |
| mmu-miR-6966-3p | -1.989885238 | 0.003006676 | down |
| mmu-miR-6979-3p | -4.700108148 | 0.017797146 | down |
| mmu-miR-6995-3p | -4.5176202 | 0.013705372 | down |
| mmu-miR-7008-5p | -4.108866229 | 0.045638043 | down |
| mmu-miR-7018-3p | -1.251664905 | 0.045648445 | down |
| mmu-miR-702-3p | -1.132152061 | 0.046678611 | down |
| mmu-miR-7027-3p | -4.362618433 | 0.033470186 | down |
| mmu-miR-9-3p | -1.170420054 | 0.031317746 | down |
| 17_42720-3p | -1.413824416 | 0.019328753 | down |
| 11_29341-3p(hsa-miR-3151-5p) | -1.90324581 | 0.034872016 | down |
| 11_29342-5p(hsa-miR-3151-5p) | -1.903547232 | 0.034309911 | down |
| 9_22987-5p | -1.618728111 | 0.018844056 | down |
| 4_10064-5p(gga-miR-1768) | -1.560909978 | 0.010742583 | down |


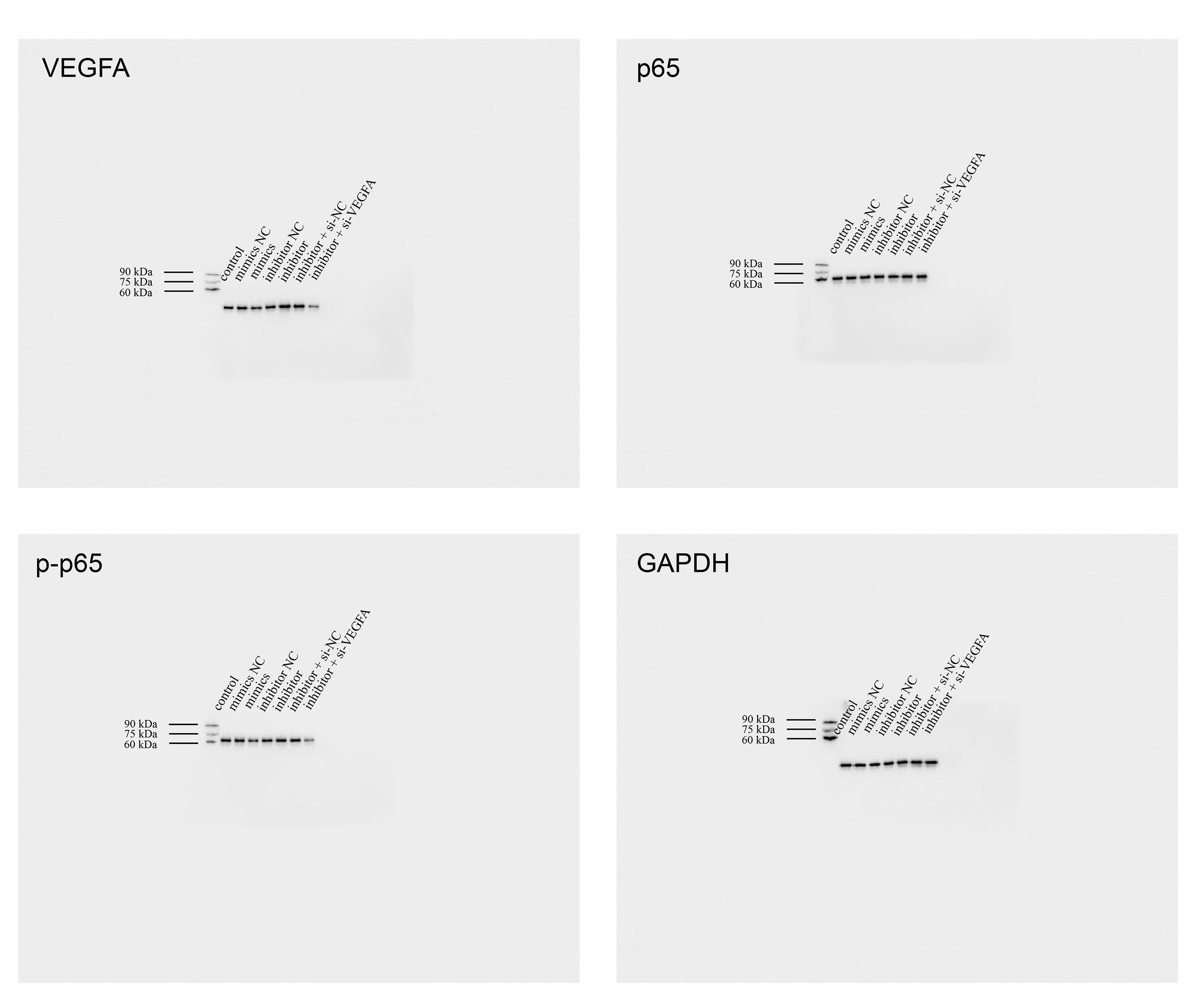


**Figure S1** The full-length gels and blots of the proteins in Figure 3C.


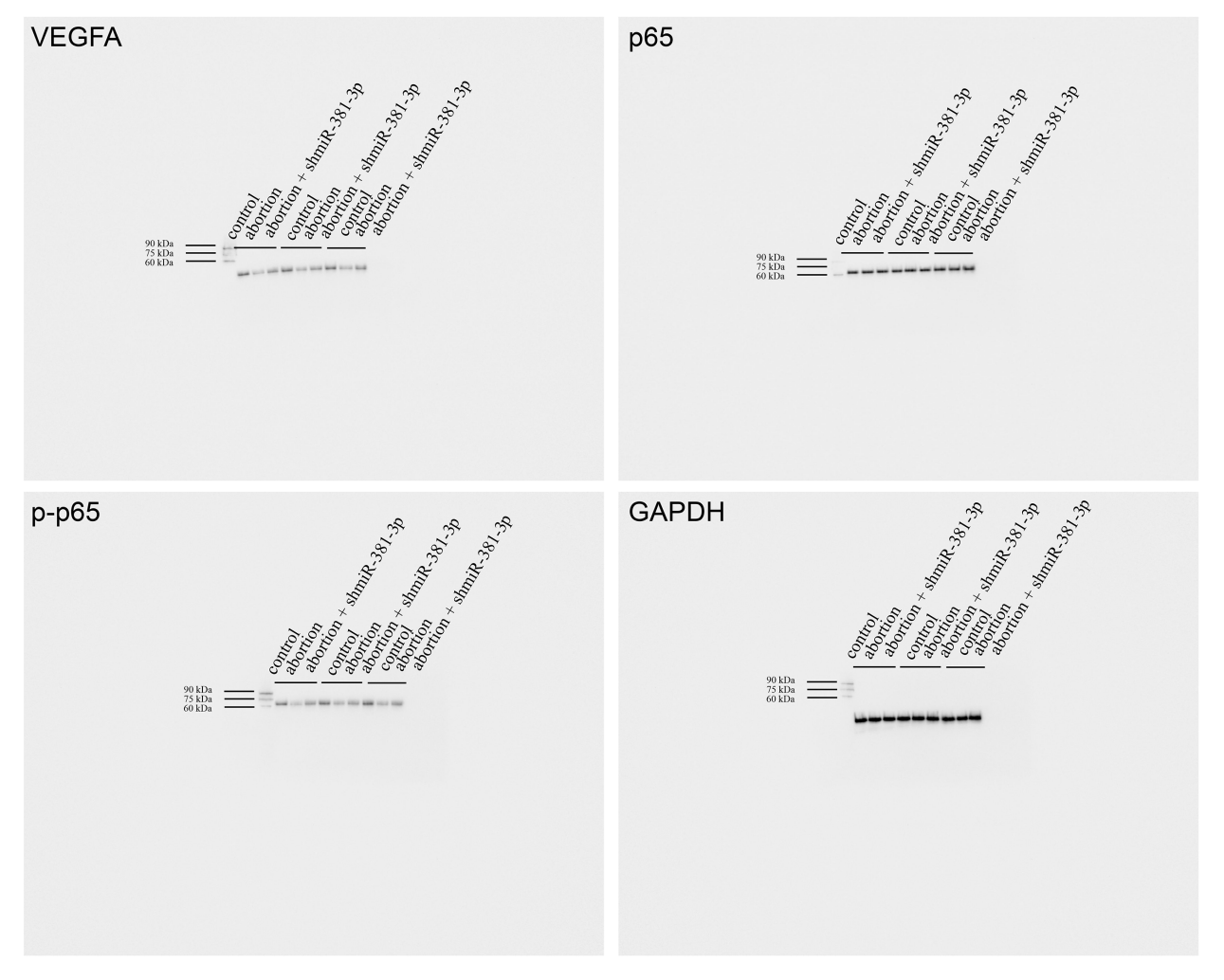


**Figure S2** The full-length gels and blots of the proteins in Figure 6B.
